# Interplay between antipredator behavior, parasitism, and gut microbiome in wild stickleback populations

**DOI:** 10.1038/s41522-025-00758-y

**Published:** 2025-07-19

**Authors:** Javier Edo Varg, Jaelle C. Brealey, David Benhaïm, Rafael Losada-Germain, Janette W. Boughman

**Affiliations:** 1 Department of Ecology and Genetics, section of Animal Ecology, Evolutionary Biology Centre, Uppsala University, Uppsala, Sweden; 2 Department of Aquatic Sciences and Assessment; Section for Ecology and Biodiversity, Swedish University of Agricultural Sciences, Uppsala, Sweden; 3 Department of Integrative Biology, Evolution, Ecology & Behavior program, Michigan State University, East Lansing, MI USA; 4 Department of Biological Sciences, Universidad De Los Andes, Bogota, Colombia; 5 Department of Natural History, NTNU University Museum, Norwegian University of Science and Technology (NTNU), Trondheim, Norway; 6 Land and Biodiversity, Norwegian Institute for Nature Research (NINA), Trondheim, Norway; 7 Department of Aquaculture and Fish Biology, Hólar University, Sauðárkrókur, Iceland

**Keywords:** Microbial communities, Environmental microbiology

## Abstract

The impact of microbial composition on stress-related behavior in aquatic organisms is poorly understood. This study explored the link between antipredator behavior, parasitism, and the gut microbiome in wild stickleback from two lakes: clear, spring-fed Galtaból and turbid, glacial-fed Þristikla. Behavioral analysis revealed differences between populations, with each exhibiting unique baseline behaviors. Microbiome analysis showed that a small proportion of its variation was explained by population, likely reflecting differences in lake environments. Only the marine genus *Pseudoalteromonas* abundance differed between populations. Our findings suggest that behavior and microbiome correlations may primarily reflect environmental adaptations and parasite status rather than direct gut-brain interactions. However, some tentative evidence suggests a potential innate connection between some antipredator behavior and microbiome composition. The study highlights the complexity of the gut-brain axis in wild populations and suggests future research directions, including experimental manipulations to uncover causal relationships between microbiome composition and behavior.

## Introduction

Stress is pervasive in natural environments, shaping ecosystems and their inhabitants. Habitats globally experience constant change and encounter a variety of stressors, which in turn influence animals and select individuals capable of responding to and tolerating environmental changes^1,2^. Stressors, originating from both anthropogenic and natural sources such as predation, can impact phenotypic variation, reduce structural complexity, and diminish diversity in ecosystems^3,4^. Changes in environments might affect the response of organisms to predation stress and other environmental stresses^2,5^. Predation stress is recognized to affect life-history traits and behavior, as well as interactions between organisms and their resident microbes^6–8^. However, the impact of the composition and dynamics of the microbes that inhabit aquatic organisms remains inadequately understood^3^. Similarly, our understanding of the relationship between stress-related changes in behavior and the microbiome is limited^9–11^.

Predation is a key selective force driving the evolution of various antipredator adaptations in prey, such as morphology^12,13^, behavior, physiology^14^ and life history traits^15^. These often co-vary^16^ and determine an individual’s fitness. Chemical and visual cues from predators have been shown to prompt changes in the spatial behavior of juveniles from fish species such as sticklebacks (*Gasterosteus aculeatus*), perch (*Perca fluviatilis*), and rainbow trout (*Oncorhynchus mykiss*), leading to decreased foraging and exploratory swimming^17–22^. A direct encounter with a predator usually includes a visual cue, which serves as a spatially and temporally reliable indicator of the presence of a risk, thereby indicating a high predation risk^23^. The antipredator behavior of fish depends on many factors, such as the distance to the predator, the behavior and hunting strategy of the predator, the species of predator, group size, and spatial dynamics of the prey fish, and various environmental factors^24–28^, as well as the evolutionary history of predation risk. Behavioral responses to predation have been extensively studied in stickleback fish. This species exhibits a range of antipredation behaviors, including freezing, adopting a defensive posture by swimming slowly with dorsal and pelvic fins or spines fully extended, and actively approaching the threat in a deliberate, slow manner to deter the predator or assess its intentions^29^. Alternatively, they may employ escape responses such as retreating, jumping, swimming away, or freezing^30^ and fast-start^31^.

Parasites are considered environmental stressors that impose fitness costs on their hosts, and play an important role in the evolution and ecology of many fish phenotypic traits, such as morphology, physiology, and behavior^32,33^. Behavioral changes, known as host manipulation, are extensively described in the literature^34,35^. They include changes in foraging efficiency, time budget, habitat selection, competitive ability, predator-prey relationships, swimming performance, sexual behavior, and mate choice^33,36^.

Parasites such as cestodes can impact fish swimming behavior in various ways, including quantifiable reductions in swimming performance, such as speed and stamina, increases in conspicuous behaviors like erratic movements, and escape responses of prey schools^36–39^. The underlying mechanisms may involve atrophy of musculature or pathology of the nervous system^40^. Additionally, parasites may increase the energetic cost of locomotion by affecting the hydrodynamic properties of fish, including buoyancy or buoyancy control^36,40,41^, although these effects appear to be mediated by physiological changes, rather than the mechanical effect of the parasite mass inside the fish^38^.

The antipredator response in parasitized fish can be affected by altering spatio-temporal overlap with their predators^36^. For example, threespine sticklebacks (*Gasterosteus aculeatu*s) infected by *Schistocephalus solidus* return to food patches more quickly than uninfected conspecifics following a simulated predator attack^42^. Predator avoidance can be undermined through different mechanisms, such as a decreased propensity for infected fish to join and remain with shoals^36^ or a reduction in the efficiency of the escape response. For example, the effects of parasite load on the fast-start performance of the threespine stickleback are associated with negative impacts on escape kinematics^43^, while also increasing cortisol levels, indicating an acute stress response^44^.

In addition to the above effects, the microbiome of the fish can also be affected by physiological changes in response to predation and parasite stress^6–8,45,46^. The microbiome plays key roles in the associated host, including involvement in the immune response, development, production of key metabolites, and even influencing the function of vital organs such as the brain^47–49^. Recently, there has been an increase in the literature on the effect of gut microorganisms on the host’s brain and behavior. For example, Collins and Bercik^50^, show that the nervous system, as well as the immune system, can play an important role in the regulation of the host microbial communities and vice versa. The main mechanism of microorganisms interacting with the brain is based on microorganism-derived neuroactive chemicals^11^. The coined name for this interaction is the microbiota-gut-brain-axis. Some studies have shown that the microbiome can affect host behavior, likely mediated through this microbiota-gut-brain-axis^51^. For example, in a zebrafish model, a probiotic supplement increased exploratory behavior and protected fish against stress-induced changes in the gut microbiome^52^. However, associations between microbiome changes and host behavior have been poorly studied in wild systems. Thus, while the importance of the microbiome in the host is unmistakable, more knowledge is needed regarding links between microbiome composition and host behavior in a variety of systems and under different stressors, such as predation or parasite infection.

The gut microbiome has been shown to play an important role during parasite infections. The gut microbiome can promote or inhibit parasite colonization and reproduction^53–55^, while parasite exposure and infection can disrupt the normal gut microbiome composition^45,46,56^. Parasites can interact directly with gut microbes as they traverse the gut, but they can also indirectly modify the gut microbiome through interactions with the host immune system^57^, even when residing in other body sites, e.g., the body cavity of intermediate hosts^45^. Parasites can also harbor their own microbes that may interact with the vertebrate host microbiome^58–61^ and act as vectors for introducing novel, potentially pathogenic microbes to the host animal^62–64^. Together, these studies highlight the importance of including parasite status in microbiome studies of wild populations, to disentangle interactions between parasite, host microbiome and the environment.

Substantial research shows that threespine sticklebacks exhibit extensive geographic variation among populations in a multitude of phenotypic traits and are often genetically differentiated^65^. This includes variation among populations in predation regimes and antipredator responses^66,67^, as well as the overall incidence of parasitism and which parasites typically infect them^68^. Recently, population variation in the microbiome and its role in adapting to distinct environments has received attention^69,70^, including adapting to varying parasite communities^71,72^. Exposure to predators and parasites varies for populations that are ecologically differentiated, and such differences are likely to affect both antipredator behavior and the microbiomes associated with hosts.

We studied two populations of threespine sticklebacks inhabiting ecologically differentiated lakes in Iceland. One lake, Galtaból, is fed by springs and is quite clear, while the other lake, Þristikla, is fed by glacial melt and is quite turbid, reducing visibility^67^. The sensory environment in these two lakes is thus quite distinct and expected to alter both the detection of predators and escape behavior^67,73^. Although we were unable to fully evaluate the predator community or predation intensity, other aspects of ecology are likely to differ between lakes, including the prey community^74^, the substrate, depth, and size of the lakes (Lucek et al.^75^), and their water chemistry (Young, Boughman et al. unpublished data). Thus, we reasoned that the two populations might differ in their antipredator behavior, parasitism, and their microbiome^76^.

We explored a set of questions to further understand the association between behavior and microbiomes in populations adapted to diverse environments. First, we asked whether glacial and spring-fed populations differ in their response to predatory attack, including their escape behavior, swimming activity, and boldness, and how parasitism affects these responses. Next, we asked whether the composition or diversity of host microbiome differed for glacial and spring-fed populations. We also asked whether the microbiome for parasitized and non-parasitized fish differ. We focused on a major parasite of threespine stickleback, the cestode *Schistocephalus solidus* because it is known to affect many aspects of stickleback behavior and physiology including antipredator behavior^39,77,78^ and the microbiome^57,72^. And last, given the important functional role of the microbiome for the host stress response, we explored whether aspects of antipredator behavior and the microbial community were related and if associations correlated with population or parasite status. To address these questions we simulated predatory attack on threespine sticklebacks from these two ecologically differentiated populations, observed their behavioral responses, recorded the presence of *S. solidus*, and characterized the gut microbial community for each fish in our experiment. We tested a set of predictions relating to the questions posed above. First, we predicted that Galtaból and Þristikla fish would differ in their antipredator behavior and in traits such as boldness and swimming activity, with Galtaból fish, inhabiting a clear-water environment, showing enhanced antipredator responses. Next, given the differences in lake ecology, we predicted that the two populations would differ in their microbial community, and also that microbiomes would differ for parasitized and non-parasitized fish. Lastly, we predicted that antipredator behavior would be related to the gut microbial community in some manner.

## Results

### Behavior: effect of robotic predator presence before simulated attack

**Fig. 1:**
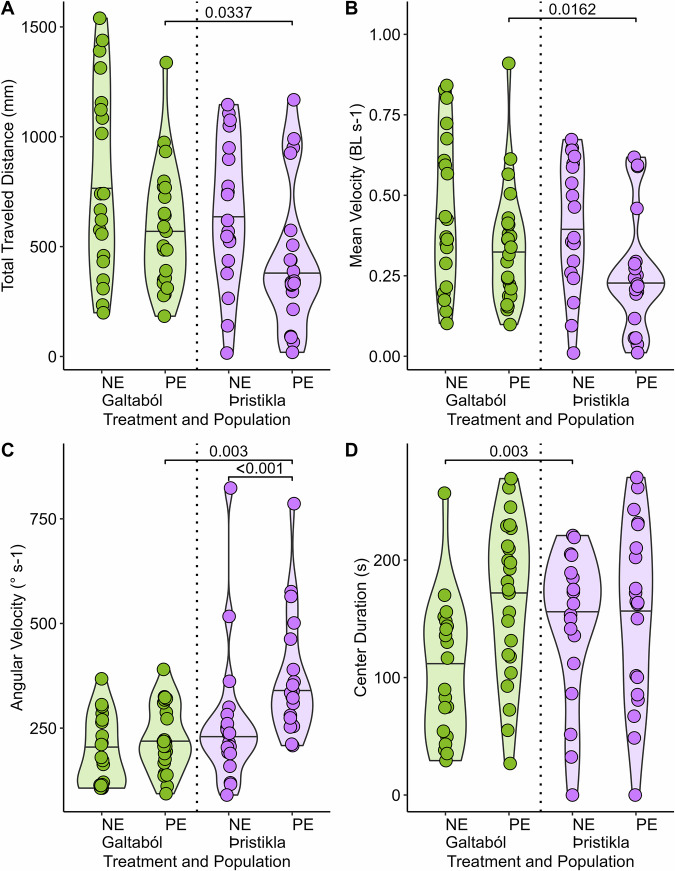
Effects of acclimation on the swimming activity behavior and tank use, before simulated predator attack period. **A** Total traveled distance in mm. **B** Mean velocity in body length (BL) per second. **C** Angular velocity in degrees per second. **D** Time (s) spent in the center zone, an indicator of boldness. Samples are grouped by population (glacial Þristikla and spring-fed Galtaból), and treatment (NE non-exposed fish, without presence of predator in tank, PE predator-exposed fish, with presence of predator in tank but before simulated attack). *P* values from significant (*p* < 0.05) post-hoc comparisons are shown above square brackets (full results are provided in Supplementary Table 1). Significance was assessed by post hoc contrasts focused on testing our stated hypotheses.

We compared our two ecologically different fish populations in the non-exposed fish to characterize baseline behavioral differences among populations. Those that differed among populations were aspects of activity including total distance traveled and angular velocity, with spring-fed Galtaból fish displaying higher distance traveled and glacial Þristikla fish showing higher angular velocity (Table 1, Fig. 1).

**Table 1:** Results of linear models, using data from control trials and the acclimation period before predator attack, to test population (glacial Þristikla and spring-fed Galtaból fish) and Treatment (predator-exposed and non-exposed) and their interaction on swimming activity (Total Traveled Distance, Angular Velocity, and Mean Velocity) and the tank use (Center duration) in stickleback fish

*Variable*	*Effect*	*Estimate*	*s.e*.	*df*	_*F*_	*p-value*
Total Traveled Distance	Intercept	27.181	1.628	1	870.39	**<0.0001**
	Population	−2.667	2.333	1	4.3918	**0.039314**
	Treatment	−3.649	2.205	1	7.3339	**0.008292**
	Population: Treatment	−1.394	3.21	1	0.1885	0.665356
Mean Velocity	Intercept	0.64736	0.03962	1	863.37	**<0.0001**
	Population	−0.03569	0.05676	1	2.6395	0.108222
	Treatment	−0.08393	0.05364	1	8.1798	**0.005417**
	Population: Treatment	−0.05551	0.0781	1	0.5051	0.479349
Angular Velocity	Intercept	2.248	0.04027	1	14243.2	**<0.0001**
	Population	0.11437	0.0577	1	17.1567	**<0.0001**
	Treatment	0.07676	0.05453	1	10.2051	**0.002013**
	Population: Treatment	0.10009	0.07939	1	1.5896	0.211098
Center Duration	Intercept	106.12	14.83	1	387.74	**<0.0001**
	Population	39.77	21.25	1	0.9282	0.33826
	Treatment	61.58	20.09	1	6.0293	**0.01627**
	Population: Treatment	−51.36	29.24	1	3.0848	0.0829

Significant effects are in bold.

We compared the behavior of non-exposed fish to that of predator-exposed fish during the acclimation period for two purposes. Firstly, we aimed to determine if there was any effect of the presence of the robotic predator on activity-related behaviors. We observed differences in activity, with all the variables being impacted by the presence of the robotic predator regardless of the populations (i.e., non-exposed versus predator-exposed treatment, significant effect in Fig. 1 and Table 1). The variables total distance traveled and mean velocity were negatively affected, whereas the angular velocity was positively affected in the presence of the predator (Fig. 1, Table 1).

Secondly, we assessed boldness by comparing tank use of the non-exposed fish to that of the predator-exposed fish. An increase in center duration was identified as bold behavior (Fig. 1D), while a decrease was indicative of shy behavior. We observed an effect of the robotic predator regardless of the population: center duration increased in the presence of the predator (Table 1, Fig. 1D).

### Effect of the simulated predatory attack

**Fig. 2:**
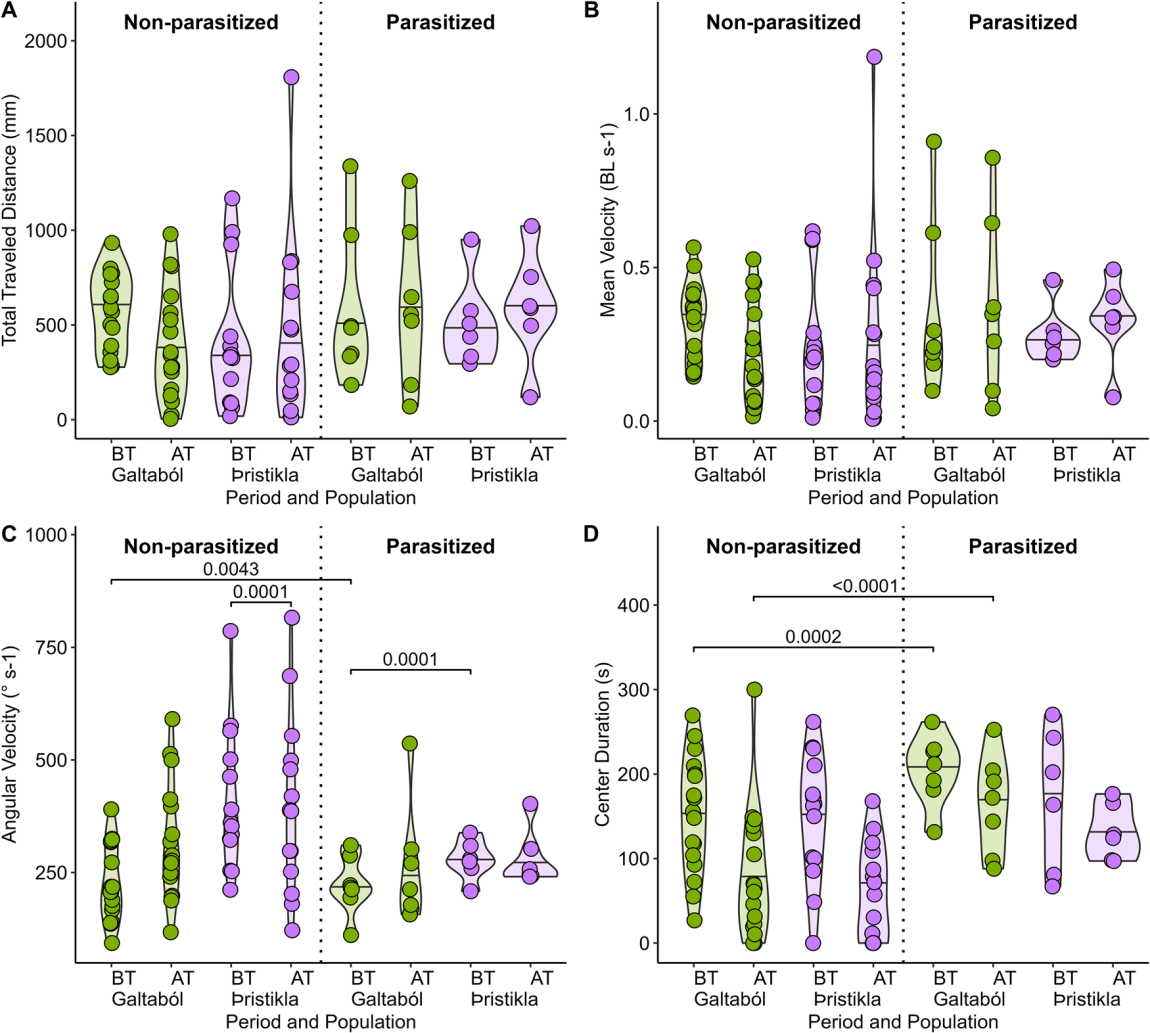
Comparison of swimming activity and tank use behavior before and after the simulated predatory attack on predator-exposed fish, in non-parasitized and parasitized groups. **A** Total traveled distance in mm. **B** Mean velocity in body length (BL) per second. **C** Angular velocity in degrees per second. **D** Time (s) spent in the center zone, an indicator of boldness. Samples are grouped by parasite status, population (glacial Þristikla and spring-fed Galtaból), and time period (before (BT) and after (AT) the simulated predatory attack). *P* values from significant (*p* < 0.05) post-hoc comparisons are shown above square brackets (full results are provided in Supplementary Table 2). Note: control fish which were never exposed to the predator are not shown in this figure. Significance was assessed by a post hoc of multiple comparison test on the population by Period by Parasite interaction.

In predator-exposed fish, we compared behavior during the acclimation period to that after the simulated predatory attack to examine behavioral responses to predation and to investigate potential differences in responses between glacial and spring-fed populations. We found a significant interaction between population and period for angular velocity, which increased after simulated attack in Galtaból (spring-fed fish), whereas it was the opposite in Þristikla (glacial fish, Table 2, Fig. 2C). Furthermore, the simulated predatory attack influenced center duration (Table 2), leading to a decrease in time spent in the tank center and an increase in time spent at the tank border after the attack for both glacial and spring-fed populations (Fig. 2D).

**Table 2:** Results of mixed-effects models (Fish ID as random effect) testing for the effects of robotic predator attack, period (before and after triggering the robotic predato), population (glacial Þristikla and spring-fed Galtaból fish), effect of parasites and all interactions on swimming activity (Total Traveled Distance, Angular Velocity, and Mean Velocity) and the tank use (Center Duration, and Distance to Predator) in stickleback fish

*Variable*	*Effect*	*Estimate*	*s.e*.	*df*	$\chi 2$	*p-value*
Total Traveled Distance	Intercept	18.2051	1.9748	1	342.415	**<0.0001**
	Population	0.1233	2.9387	1	0.3717	0.5421
	Period	5.4564	2.0611	1	0.6014	0.438
	Parasite	4.7571	3.6567	1	2.1608	0.1416
	Population:Period	−5.515	3.067	1	1.5642	0.2111
	Population:Parasite	0.4693	5.3997	1	0.2853	0.5933
	Period:Parasite	−5.2034	3.8164	1	1.2998	0.2542
	Population:Period:Parasite	3.9818	5.6356	1	0.4992	0.4798
Mean Velocity	Intercept	0.44038	0.04813	1	329.0625	**<0.0001**
	Population	0.01209	0.07162	1	0.4502	0.5022
	Period	0.12044	0.04865	1	0.4822	0.4874
	Parasite	0.12466	0.08912	1	1.9146	0.1665
	Population:Period	−0.12387	0.07239	1	1.4108	0.2349
	Population:Parasite	−0.02137	0.1316	1	0.0428	0.8361
	Period:Parasite	−0.11569	0.09008	1	1.134	0.2869
	Population:Period:Parasite	0.08973	0.13302	1	0.4551	0.4999
Angular Velocity	Intercept	2.4651	0.042	1	10449.17	**<0.0001**
	Population	0.08237	0.0625	1	7.3513	**0.0067**
	Period	−0.14152	0.04675	1	1.5614	0.211462
	Parasite	−0.08738	0.07777	1	3	0.083267
	Population:Period	0.17588	0.06957	1	3.0556	0.08046
	Population:Parasite	−0.01778	0.11484	1	0.7374	0.390509
	Period:Parasite	0.09143	0.08657	1	0.1823	0.6694
	Population:Period:Parasite	−0.12829	0.12784	1	1.007	0.31563
Center Duration	Intercept	80.14	16.18	1	222.4518	**<0.0001**
	Population	−18.16	24.08	1	1.3567	0.244105
	Period	72.19	17.26	1	25.8844	**<0.0001**
	Parasite	84.07	29.96	1	9.2842	**0.002311**
	Population:Period	15.46	25.69	1	0.0901	0.764109
	Population:Parasite	−14.34	44.24	1	0.3687	0.543731
	Period:Parasite	−31.4	31.96	1	2.8423	0.091814
	Population:Period:Parasite	−16.76	47.2	1	0.1261	0.722489
Distance To Predator	Intercept	5.32292	0.42097	1	159.8779	**<0.0001**
	Population	0.08641	0.62643	1	0.019	0.8903
	Period	0.12406	0.49038	1	0.064	0.8003
	Parasite	−0.32829	0.77949	1	0.1774	0.6736
	Population:Period	0.46214	0.72971	1	0.4011	0.5265
	Population:Parasite	0.301	1.15105	1	0.0684	0.7937
	Period:Parasite	−0.07437	0.90801	1	0.0067	0.9347
	Population:Period:Parasite	−0.55779	1.34084	1	0.1731	0.6774

Significant effects are in bold.

### Effect of parasites on antipredator behavior

We did not observe any significant interaction between parasite, population, and period, nor any significant interaction between parasite and population for any behavior (Table 2). The parasite effect overall was significant for center duration only (Table 2). Center duration (proxy for boldness) significantly increased in each population when parasitized (Table 2, Fig. 2D).

### Microbiome

All samples for microbiome analysis were taken after the predatory attack (one per individual for non-exposed and predator-exposed fish). After sequencing, sample and taxa quality filtering and taxa contamination filtering (see methods), 1673 microbial taxa at the species-level were recovered from 62 stickleback gut samples, representing 652 genera. We observed some batch effects of extraction date on microbiome composition (Supplementary Figs. 2, 3, Supplementary Table 3), thus we took extraction date into account in all analyses (see methods). Due to low sample numbers for the parasitized Þristikla group, these individuals were not included in the microbiome analysis. Instead, we focused on comparisons between (i) non-parasitized Galtaból (*n* = 24) and Þristikla (*n* = 25) individuals, and (ii) non-parasitized (*n* = 24) and parasitized (*n* = 13) Galtaból individuals.

**Fig. 3:**
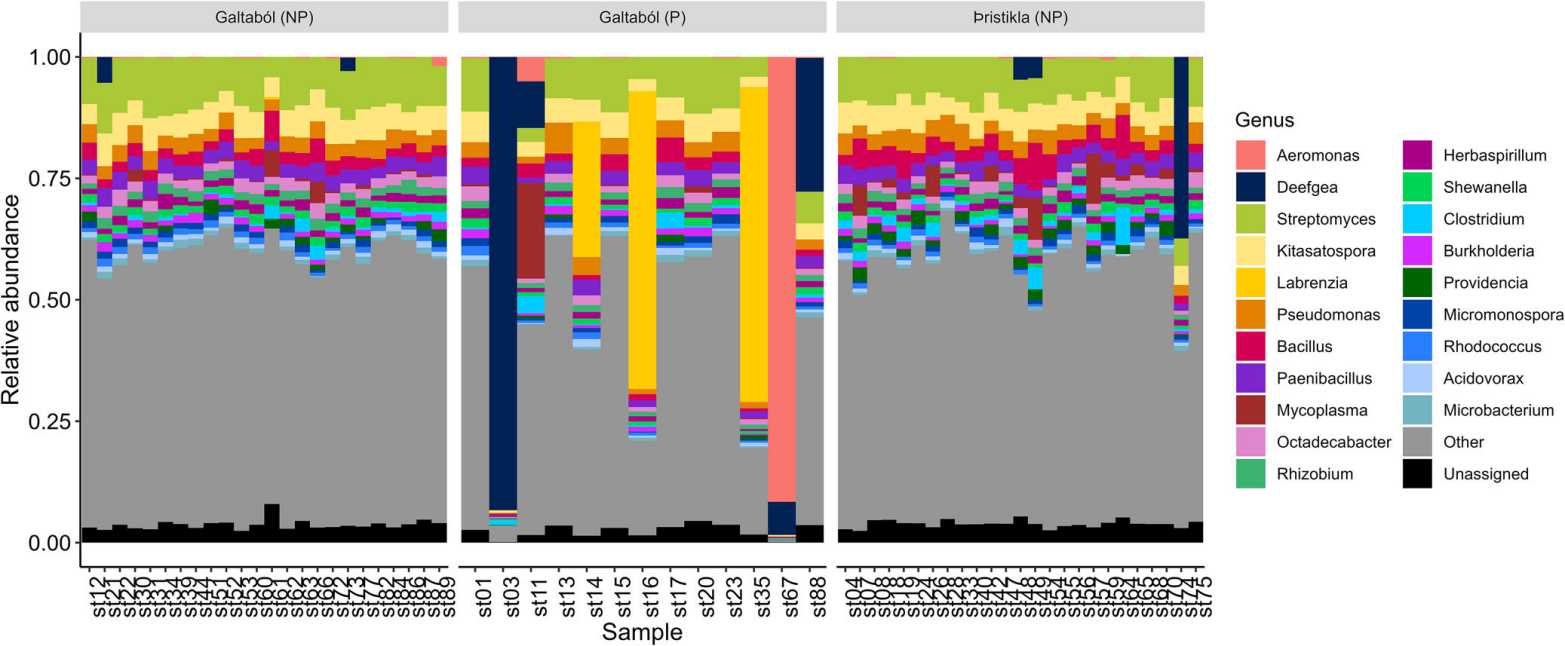
Gut microbial composition of non-parasitized (NP) and parasitized (P) individuals from the Galtaból and Þristikla populations. The 20 most abundant genera are shown, the rest are grouped as “Other”. Species-level taxa that could not be assigned at the genus level are grouped as ‘Unassigned’.

**Fig. 4:**
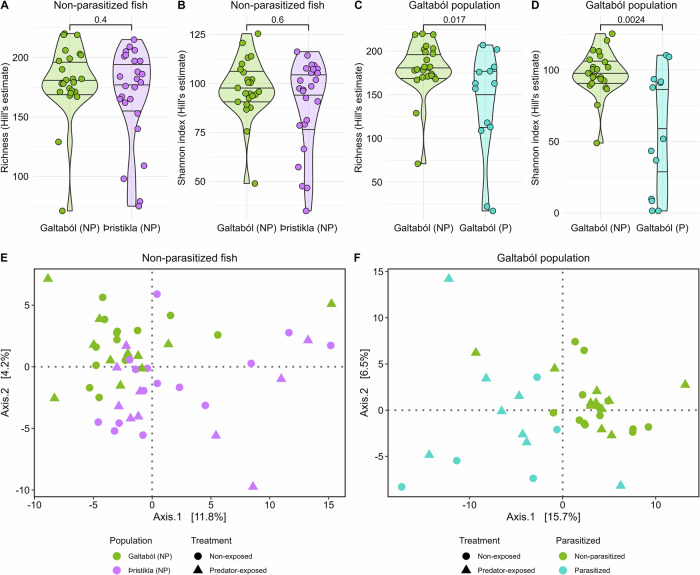
Alpha and beta diversity in the gut microbiome of non-parasitized (NP) and parasitized (P) populations. **A**, **B** Alpha diversity metrics between non-parasitized Galtaból (*n* = 24) and Þristikla (*n* = 25) populations. **C**, **D** Alpha diversity metrics between non-parasitized (*n* = 24) and parasitized (*n* = 13) Galtaból individuals. Alpha diversity metrics were estimated at the genus-level using a Hill number framework for richness (**A**, **C**) and Shannon index (**B**, **D**). Violin plots with all samples as colored points show the distribution of the data, while the quartiles (0.25, 0.5, and 0.75) of the metric in each group are shown as black lines. Metrics were compared between populations using a Wilcox test and the resulting *p* values are shown above the brackets. PCoA of gut microbiome composition of non-parasitized (NP) Galtaból and Þristikla populations (**E**) and non-parasitized and parasitized Galtaból individuals (**F**). Shape (“Treatment”) indicates whether the fish were in the non-exposed (circles) or the predator-exposed treatment (triangles). Taxa abundances were summed to the genus-level and normalized using the centered-log ratio transformation. Variations due to extraction batch effects were regressed out (see Supplementary Fig. 3 for before-correction PCoAs). Euclidean distances were then calculated and PERMANOVAs performed. Population in (**E**) explained 4.2% of the variation in microbiome composition (*F* = 1.93, *p* = 0.003), while parasite status in (**F**) explained 7.2% of the variation (*F* = 2.42, *p* = 0.001). In both analyses, sequencing depth explained 5.0–6.4% of the variation in microbiome composition (*p* < 0.002) Full PERMANOVA results, including PERMANOVAs before batch correction, are reported in Supplementary Table 3.

The sticklebacks generally had quite diverse gut microbiomes at the genus level, with the most abundant identified genera including *Streptomyces* and *Kitasatospora* (Fig. 3). While there were no differences in alpha diversity between non-parasitized Galtaból and Þristikla individuals (Fig. 4A, B), the populations had significantly different gut microbiome compositions after adjusting for batch effects (Fig. 4E; Supplementary Table 3). Population explained 4.2% of the variation in microbiome composition (*F* = 1.93, *p* = 0.003). However, sequencing depth contributed to 5.0% of the variation in the data (*F* = 2.30, *p* = 0.001) and thus could be confounding the results. *Pseudoalteromonas* had significantly higher relative abundance in the non-parasitized, spring-fed Galtaból individuals compared to Þristikla individuals (adjusted *p* < 0.001, Supplementary Table 4); however, no other taxa were associated with differential abundance between populations.

In comparison to non-parasitized individuals in the spring-fed Galtaból population, several parasitized Galtaból individuals were dominated by one bacterial genus, which varied between *Aeromonas*, *Deefgea* and *Labrenzia* depending on the individual (Fig. 3). Consequently, parasitized individuals had significantly lower alpha diversity compared to non-parasitized individuals (Fig. 4C, D). Parasite status explained 7.2% of the variation in gut microbiome composition (*F* = 2.42, *p* = 0.001, Fig. 4F, Supplementary Table 3). Again, sequencing depth explained 6.4% of the variation (*F* = 2.16, *p* = 0.002). In the differential abundance tests, *Kitasatospora* and *Shewanella* had significantly higher relative abundance in non-parasitized Galtaból individuals compared to parasitized ones (adjusted *p* < 0.05, Supplementary Table 4). No other taxa were associated with differential abundance between parasite status.

### Links between fish behaviors and microbiomes

The microbiome composition did not differ significantly between non-exposed and predator-exposed fish, based on the PCoA (Fig. 4E, F, Supplementary Table 3). Likewise, no microbial genera were associated with predator exposure in a differential abundance analysis.

As an alternative approach to explore associations between the microbiome and behavioral datasets, we used MOFA, an unsupervised, multivariate approach. With MOFA, we aimed to identify coordinated changes between behavioral traits and microbe abundances and correlated these changes with fish population and parasite status. Since the predator-exposed fish included behavioral variables not relevant for the non-exposed fish (distance to and duration around the predator stimulus), non-exposed fish and predator-exposed fish were analysed separately.

The MOFAs revealed some evidence for associations between behavioral traits and microbe abundances. Generally each factor in the MOFAs explained substantial variation in only one dataset (Fig. 5). However, the MOFA on predator-exposed fish identified three factors explaining >0.75% of variation in both datasets (Factor 1, 3 and 4 in Fig. 5C). We therefore focus on these three factors (see Supplementary Figs. 4–14 for summary figures from the other factors).

**Fig. 5:**
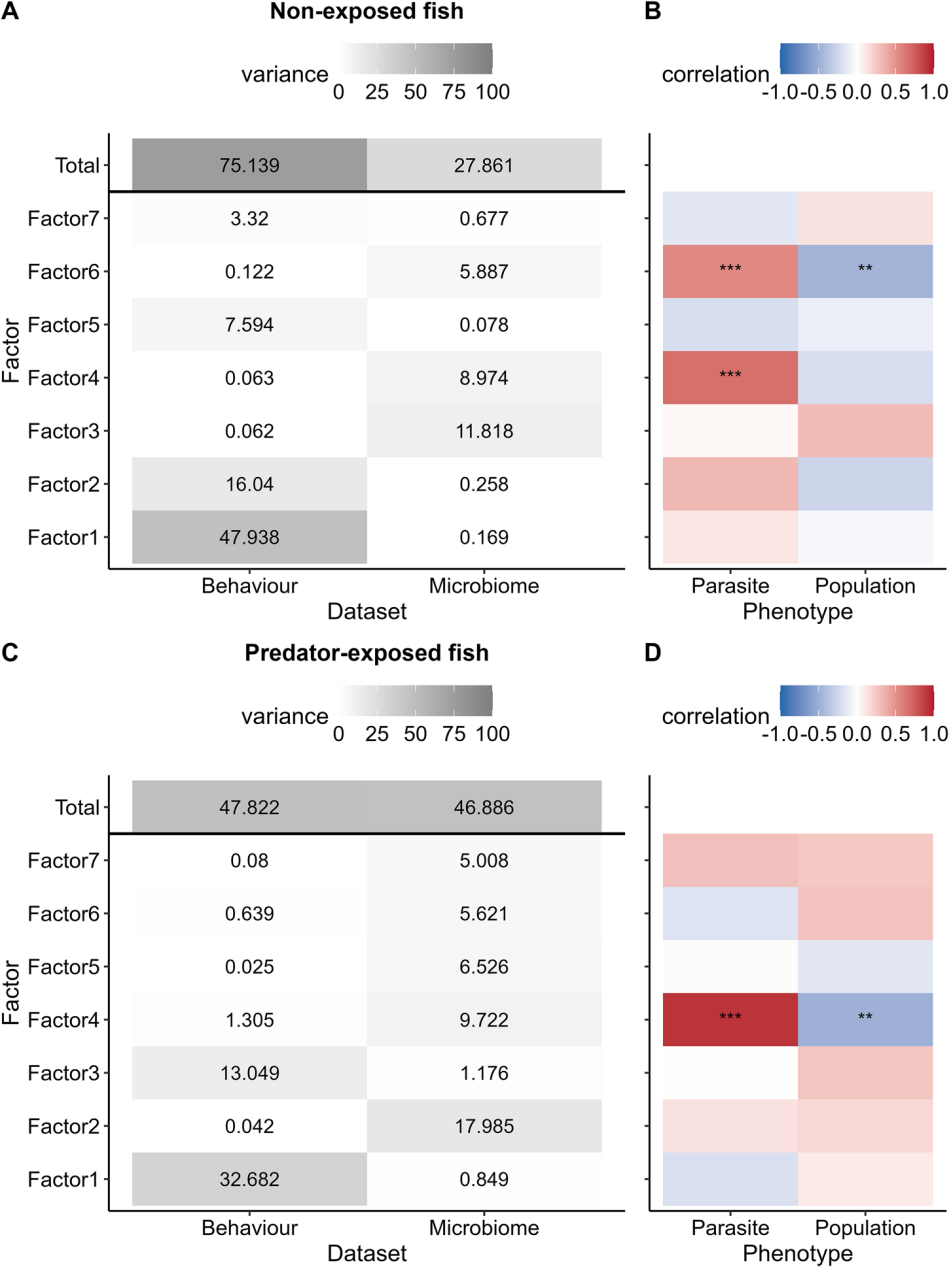
Summary results from the MOFA2 model on non-exposed and predator-exposed fish. **A**, **C** Percentage of variance explained by each factor across each dataset in non-exposed fish (**A**) and predator-exposed fish (**C**). The total variance explained by each model in each dataset is also included at the top of the heatmaps. **B**, **D** Correlation of factors with fish parasite status and population in non-exposed fish (**B**) and predator-exposed fish (**D**). Significant correlations are indicated by * (*p* < 0.05), ** (*p* < 0.01) and *** (*p* < 0.001). Red–blue heatmap indicates whether population or parasite was positively or negatively correlated with the factor.

**Fig. 6:**
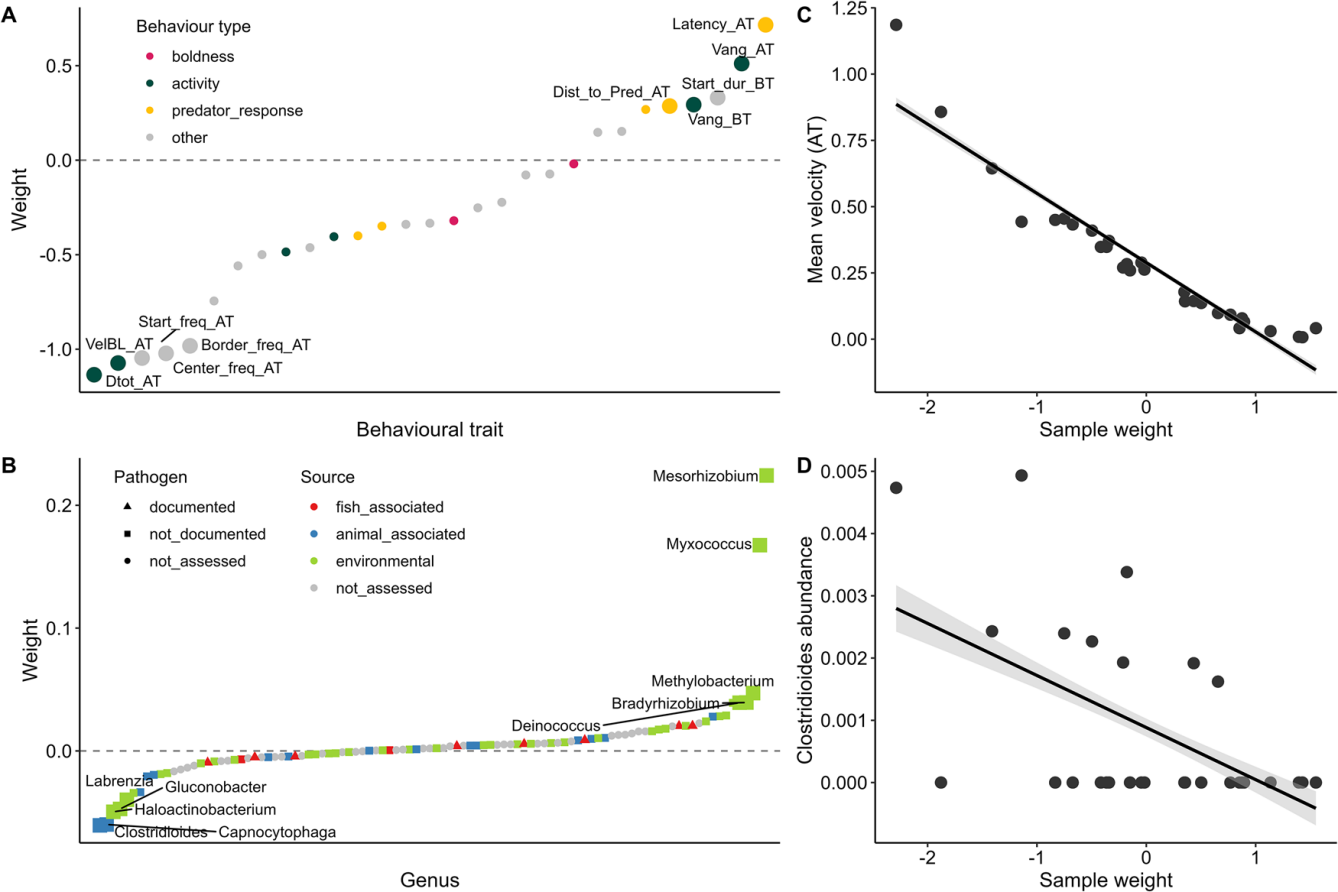
Associations identified by MOFA in predator-exposed fish among behavioral traits and microbial abundances, for factor 1. **Factor 1** explained 33% of the variance in the behavioral dataset and 0.85% of variance in the microbiome dataset in predator-exposed fish. Factor 1 was not significantly correlated with population or parasite status (*p* > 0.05). **A** Contribution (weight) of behavioral traits to factor 1. Traits are ranked according to their weight. The higher the absolute weight, the more strongly associated a trait is with the factor. A positive weight indicates the trait has higher levels in samples with positive factor values, while a negative weight indicates the opposite. Behavioral traits are colored by their broader behavioral category. The top five traits contributing to the factor in each direction are labeled. Behavioral trait abbreviations: AT measurement after simulated predator attack, BT measurement before simulated predator attack, dist distance, Dtot total distance traveled, dur duration of time spent in the respective tank zone, freq number of times fish visited the respective tank zone, pred measurement relating to the position of the predator, Vang angular velocity, VelBL mean velocity in body length per second. **B** Contribution (weight) of microbial genera to factor 1, displayed in the same way as for (**A**). Microbial genera are colored by their putative source and shaped on their potential as a fish pathogen, based on a literature search (Supplementary Table 5). **C** Example association between factor 1 sample weights vs a top behavioral trait, mean velocity after attack (AT). A smoothed linear conditional means line is shown in black with confidence intervals in gray. **D** Example association between factor 1 sample weights vs a top microbial genus, *Clostridioides*, displayed in the same way as for (**C**).

For predator-exposed fish, in Factor 1, traits relating to swimming activity and response to the predator stimulus contributed the most in the behavioral dataset (Fig. 6A), while environmental genera like *Mesorhizobium* and animal-associated bacteria like *Clostridioides* contributed the most in the microbial dataset (Fig. 6B). In particular, mean velocity and the genus *Clostridioides* were associated with this factor (Fig. 6C, D).

**Fig. 7:**
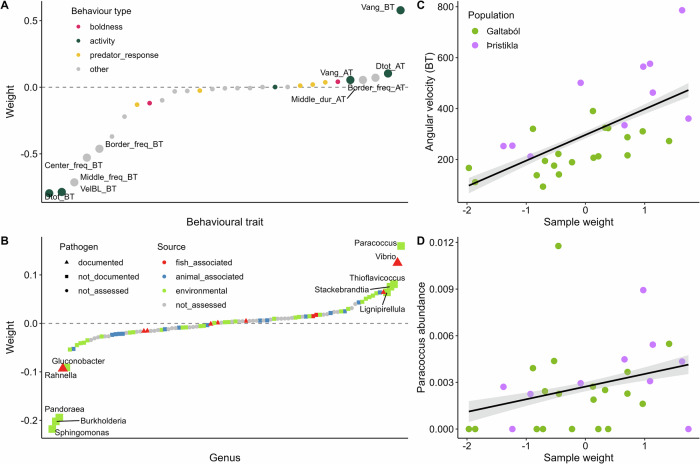
Associations identified by MOFA in predator-exposed fish among behavioral traits and microbial abundances, for factor 3. **Factor 3** explained 13% of the variance in the behavioral dataset and 1% of variance in the microbiome dataset in predator-exposed fish. Factor 3 was not significantly correlated with population or parasite status, although there was a non-significant weak correlation with population (*p* > 0.05). **A** Contribution (weight) of behavioral traits to factor 3. Traits are ranked according to their weight. The higher the absolute weight, the more strongly associated a trait is with the factor. A positive weight indicates the trait has higher levels in samples with positive factor values, while a negative weight indicates the opposite. Behavioral traits are colored by their broader behavioral category. The top five traits contributing to the factor in each direction are labeled. Behavioral trait abbreviations: AT measurement after predator trigger, BT measurement before predator trigger, dist distance, Dtot total distance traveled, dur duration of time spent in the respective tank zone, freq number of times fish visited the respective tank zone, pred measurement relating to the position of the predator, Vang angular velocity, VelBL mean velocity in body length per second. **B** Contribution (weight) of microbial genera to factor 3, displayed in the same way as for (**A**). Microbial genera are colored by their putative source and shaped on their potential as a fish pathogen, based on a literature search (Supplementary Table 5). **C** Example association between factor 3 sample weights vs a top behavioral trait, angular velocity before simulated predator attack (BT). Samples are colored by fish population. A smoothed linear conditional means line is shown in black with confidence intervals in gray. **D** Example association between factor 3 sample weights vs a top microbial genus, *Paracoccus*, displayed in the same way as for (**C**).

In Factor 3, swimming activity variables, particularly before the triggered predator attack, were among the strongest contributors in the behavioral dataset (Fig. 7A, C), while environmental genera like *Paracoccus* and *Sphingomonas* contributed the most to the microbiome dataset (Fig. 7B, D). Factor 3 was also weakly correlated with population (Fig. 5D).

**Fig. 8:**
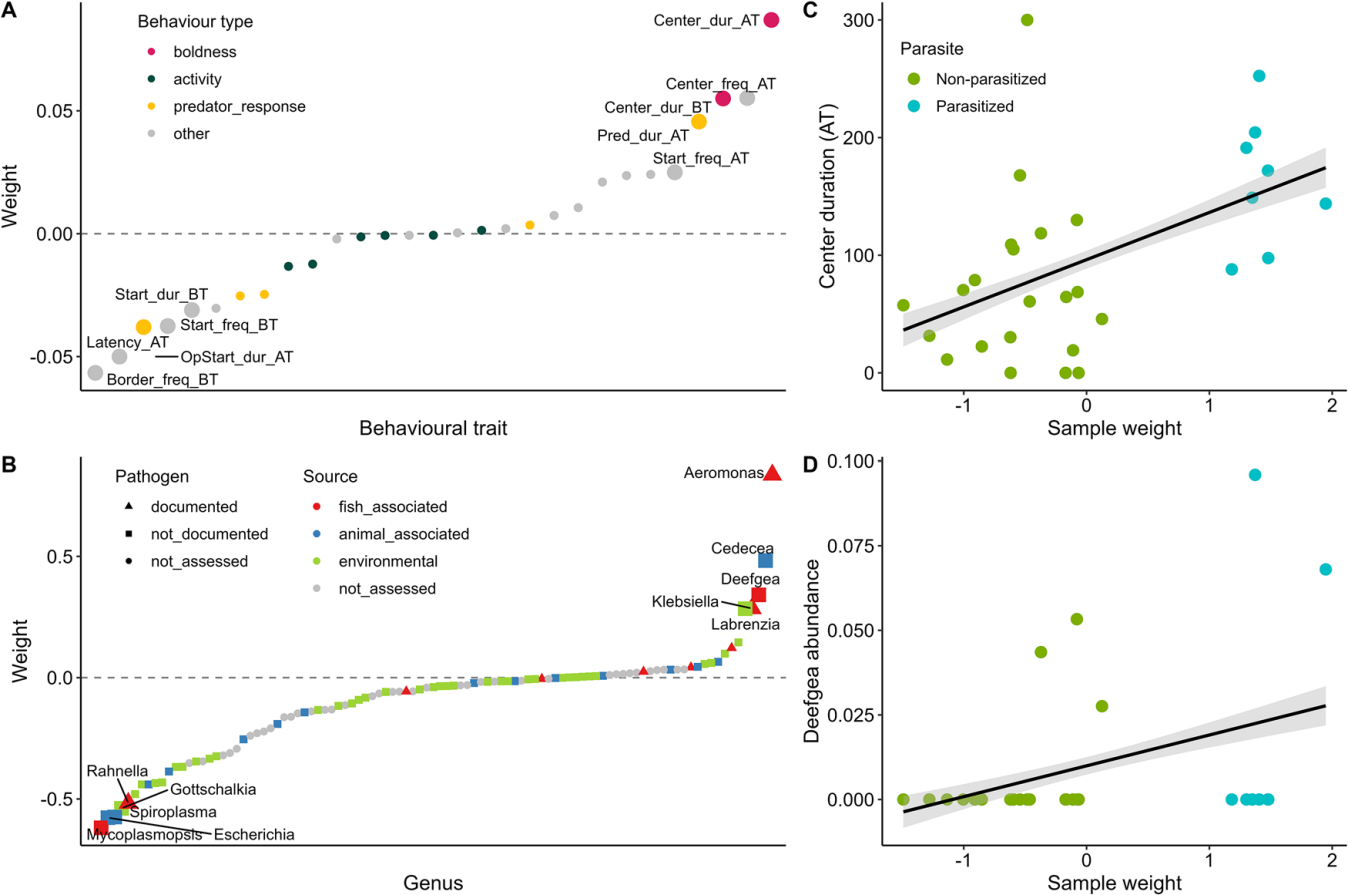
Associations identified by MOFA in predator-exposed fish among behavioral traits and microbial abundances, for factor 4. **Factor 4** explained 10% of the variance in the behavioral dataset and 1% of variance in the microbiome dataset in predator-exposed fish. Factor 4 was significantly correlated with the parasite status and population of the individual (*p* < 0.05). **A** Contribution (weight) of behavioral traits to factor 4. Traits are ranked according to their weight. The higher the absolute weight, the more strongly associated a trait is with the factor. A positive weight indicates the trait has higher levels in samples with positive factor values, while a negative weight indicates the opposite. Behavioral traits are colored by their broader behavioral category. The top five traits contributing to the factor in each direction are labeled. Behavioral trait abbreviations: AT measurement after predator trigger, BT measurement before predator trigger, dist distance, dur duration of time spent in the respective tank zone, freq number of times fish visited the respective tank zone, pred measurement relating to the position of the predator. **B** Contribution (weight) of microbial genera to factor 4, displayed in the same way as for (**A**). Microbial genera are colored by their putative source and shaped on their potential as a fish pathogen, based on a literature search (Supplementary Table 5). **C** Example association between factor 4 sample weights vs a top behavioral trait, center duration after simulated predator attack (AT). Samples are colored by parasite status (note non-parasitized includes both Galtaból and Þristikla individuals). A smoothed linear conditional means line is shown in black with confidence intervals in gray. **D** Example association between factor 4 sample weights vs a top microbial genus, *Deefgea*, displayed in the same way as for (**C**).

Factor 4 was correlated with parasite status and population (Fig. 5D). Moreover, the behavioral trait relating to boldness, center duration, contributed strongly to this factor (Fig. 8A). In the microbiome dataset, putative animal- and fish-associated microbial genera were among the strongest contributors, including the documented fish pathogen *Aeromonas* and the fish microbe *Deefgea* (Fig. 8B). For example, center duration and *Deefgea* abundance was higher in parasitized compared to non-parasitized individuals (Fig. 8C, D).

## Discussion

Our main objective in analyzing behavior and responses to simulated predation is to uncover possible links between parasitism, antipredator behavior, and the microbiome. We begin by discussing behavioral findings, followed by comparing the microbiome between populations and parasite status, and finish by discussing potential links between behavior and the microbiome suggested by our findings.

The glacial and spring-fed populations differed in baseline behavior and some responses to predation. Baseline behavior revealed that fish from the two populations were active in different ways: glacial Þristikla fish showed higher angular velocity while spring-fed Galtaból fish traveled further. Predator responses were strong for both populations as well. Comparing fish activity during the acclimation period in the predator-exposed treatment (i.e., before the predator was triggered) to the same time interval in the non-exposed treatment confirmed that the mere presence of the robotic predator influenced behavior and did so differently for the two populations, increasing time spent in the tank center (and therefore boldness) for spring-fed fish from Galtaból and increasing activity measures for glacial fish from Þristikla. Differences in activity and boldness may relate to the ability to detect predators in the distinct sensory environments of each lake. The clear waters in spring-fed Galtaból allow sticklebacks to detect predators from a distance, and unobstructed vision may foster high exploration and boldness in those open waters^24^. In contrast, the turbid waters in glacial Þristikla make visual detection of predators difficult except at very close distances^67^, perhaps fostering more erratic movements and shyness, both of which may help fish escape capture by predators^73,79^.

In spite of these population differences in both baseline behavior and in the mere presence of the robotic predator, both populations responded strongly and similarly to simulated predatory attack. This similarity is perhaps surprising given that the lakes differ substantially in ecology and visibility, and that a prior study showed different escape behaviors for glacial and spring-fed fish^67^. That study analyzed a larger set of behaviors, including first response to predator attack, and some of the differences seen were in those first responses, which we did not measure. Our findings suggest that a robust and cautious response to predator attacks is favored in both types of populations.

Both populations showed similar responses when parasitized, although there was higher overall parasitism in spring-fed Galtaból fish. Parasitized fish appeared to be bolder compared with their unparasitized counterparts in response to the robotic predator attack. This finding is in accordance with the results of previous studies on the same species, showing that parasitized sticklebacks returned more quickly to food patches following disturbance by a model predator^42^, or spent more time in the open water and reduced their flight responses to a predator attack from above^17,30,42,77^. Both of these responses would be likely to enhance transmission of the parasite by increasing the likelihood of predation, as parasites act as strong selective agents on their host by altering behavior and habitat use, ultimately decreasing the hosts’ fitness^43,80^. However, we cannot exclude undetectable effects due to the low sample size of tested parasitized fish.

Consistent with previous studies of wild-caught sticklebacks, a small but significant proportion of variation in the gut microbiome was explained by population, likely reflecting differences in lake environment and genetic background between the two populations^71,81^. However, only one microbial taxon, the typically marine genus *Pseudoalteromonas*, was significantly differentially abundant between populations. Thus, the differences in gut microbiome in our populations appear to be driven by small changes in the abundance of many taxa, rather than large differences in a few taxa. To minimize the confounding effects of transient microbes derived from food or water sources^81^, fish were co-housed by population and fed the same diet for several weeks before the experiments were performed. We therefore cannot rule out that this set-up had a homogenizing effect on the microbiome of both populations. However, this set-up did not mask the large microbiome variation observed in parasitized fish, which were co-housed with non-parasitized fish. Previous research has shown that transitioning fish from a wild to a laboratory setting, including a laboratory diet, does affect gut microbiome composition within 14 days^82^. However, a study in wild sticklebacks found that host genotype explains more variation in the gut microbiome than environmental effects like diet and habitat, suggesting that microbiome composition may depend more on host selection of microbes, rather than transient colonization from environmental sources^71^.

Our findings align with recent work by Viver et al.^83^, who showed that the transient microbiome in fish is highly dependent on the supplied food, and that fasting the fish for 80 h effectively eliminated most transient microbes, leaving the resident microbiome. In our study, despite many days of a standardized diet, we observed significant differences between ecotypes, which may reflect the persistence of the resident-core microbiome. These findings suggest that both ecotype and environment shape the microbiome as the host adapts to its surroundings. Furthermore, Viver et al.^83^, found that the bacteria present in the supplied diet were unable to successfully colonize the gut and replace the resident bacteria naturally found in the wild. These findings indicate that signals from the resident microbiome, possibly influenced by feeding habits in the wild, may still persist even after an extended period on a laboratory diet.

The effect of parasitism on the microbiome was only investigated in the spring-fed Galtaból population, where it was associated with decreased alpha diversity and an increase in abundance of specific microbial taxa, such as the potential pathogen *Aeromonas*^84^, the marine genus *Labrenzia*^85^ and the fish gut microbe *Deefgea*^86^, and the decrease of putatively commensal microbes such as *Shewanella* and *Kitasatospora*^46^. Some *Kitasatospora* species even produce secondary metabolites with anthelmintic properties^87,88^. Previous studies of fish microbiomes during parasite exposure or infection suggest that parasite infection promotes the growth of taxa that are likely not beneficial to the host, while inhibiting the growth of commensal microbes^45,46,60,72^. For example, in zebrafish, *Shewanella* was negatively associated with nematode parasite load^46^, while in sticklebacks, bacteria related to *Labrenzia* (of the Rhodobacteraceae family or Rhodobacterales order) were associated with exposure or infection with the cestode *S. solidus*^45,72^.

To our knowledge, this is the first report on the relationship between the microbiome and behavior in these wild populations. We observed some correlations between behavioral traits and the microbiome in predator-exposed fish. However, these interactions were generally weak, perhaps due to the inherent complexity of the gut-brain axis and its causal relationships, which have yet to be fully understood^10^. We acknowledge that limitations in our study, including the sample size, reduced our ability to detect small effects. Nonetheless, our results suggest some intriguing links between the microbiome and behavior in wild populations, which we discuss in more detail below.

Some of the links we observed between behavioral traits and the microbiome can be explained by fish population or parasite status, and we cannot be certain whether these are true interactions along the gut-brain axis influencing behavior, or instead if they are indirect correlations driven primarily by these factors acting in a non-behavioral context. For example, in predator-exposed fish, we observed correlations between parasite status, boldness and potential pathogens (Fig. 8), reflecting the results identified in the separate analyses discussed above.

We also observed correlations between swimming activity and putative environmental microbes in predator-exposed fish, which could not be associated with either population or parasite status. While most of the associated microbes are not well-documented, abundance of the bacterium *Clostridioides* was correlated with increased mean velocity, which can be an indicator of bolder and more active fish^89,90^. In a selection experiment of red junglefowl (the ancestors of domestic chickens), the abundances of related Clostridiales bacteria were enriched in birds selected for a low-fear of humans, compared to high-fear animals^91^. Other studies have also found links between specific microbes and their products and how these affect metabolism and locomotion of the host^92,93^. While tentative, our results are an intriguing suggestion that there could be some innate link between swimming activity, stress levels, bold/shy traits, and the microbiome.

A number of other studies have found links between the gut microbiota and host behaviors, including stress-related behavior, social behavior, locomotion, and personality, and also point out that microbes potentially act as neuromodulators or affect neural development, all of which are relevant to our study^93,94^. The reciprocal relationships we found between the microbiome and antipredator behavior are relatively weak, as evidenced by the factor correlations and factor weightings, and are seen for only a subset of microbes and behaviors in predator-exposed fish. However, this is not surprising, considering that any mechanistic connections are likely to be indirect and mediated through many pathways. These include host physiology and neural systems^94,95^ including the hypothalamus-pituitary-interrenal (HPI) axis that regulates many aspects of the stress response^96,97^, the immune system including the major-histocompatibility (MHC) locus^45,57,98^, host-microbiota crosstalk^99^, as well as coevolution between microbiota and the host^94^. These are complex systems, and thus, the connections are also likely to be multifaceted, given the many potential mechanisms influencing the microbiota-gut-brain axis^100^. This complexity means that each connection might be of small effect, yet perhaps combine for a larger effect on the host and their behavior, and the microbial community. Large effects like those seen in lab-based manipulations on germ-free animals would be exciting but seem unlikely in our wild fish^95^. We pick up significant, albeit weak relationships only in predator-exposed fish, which we argue seems to be the most likely outcome if it is the case that host antipredator behavior, population, parasite status, and the microbiome have reciprocal effects on each other. We would expect only a subset of microbes and behaviors to respond to each other, given that the microbiome plays multiple roles within hosts, and only some of these are likely to be functionally connected to host antipredator behavior and responses to stress (the variables we measured), and these connections are probably mediated through various physiological mechanisms^94^.

Our study is one of the first to investigate microbiome–behavioral associations in a wild population. Most previous studies have relied on laboratory animals, where germ-free or reduced microbiome animals and/or probiotic supplements have been used to manipulate the microbiome, resulting in large effects on behavior (reviewed in ref. ^51^). For example, in a study on zebrafish, a probiotic supplement was found to protect conventionally-raised laboratory animals against stress-induced alteration of the gut microbiome^52^. Antibiotic treatment in *Daphnia* was found to alter microbiome structure and reverse stress-mediated changes to life history traits during predator stress^8^. Similar results have been observed in mice and rats^49,101,102^. However, laboratory-reared animals often have different, potentially maladapted microbiomes compared to wild populations, possibly contributing to the large effects seen in those studies^82,95,103^. We hypothesize that our wild populations, with their “natural” microbiomes, may have adapted to the stresses of a natural environment, like predator attacks. Thus, their microbiomes might already be optimized to protect the fish against stress-related microbiome changes, perhaps helping to explain why we see few associations between behavioral traits and microbiome composition.

In the future, performing stress experiments in wild populations while manipulating the microbiome, e.g., through probiotic, antibiotic or phage therapy, might reveal stronger evidence for behavior–microbe interactions. Teasing apart causality will also require experimental manipulations. As a first step, a holo-omics approach, where multi-omics techniques like genomics, transcriptomics and metabolomics are used to simultaneously investigate both the host and the microbiome^104^ could identify gene expression networks and key metabolites involved in modulating the coordinated microbial–host response to stress. Selection experiments could compare microbiome changes over generations selected for stress-resilience or stress-susceptibility^91,95^. Potential microbial and/or host effects could then be followed up in validation experiments, to confirm causality and work out the mechanisms. For example, identifying the *Clostridioides* strain associated with bolder fish in our study and administering it as a “probiotic” before exposure to predator stress in a follow-up experiment could be used to confirm or reject its association with antipredator response behavior. To determine causal effects of a microbial community, fecal microbiome transplants could be performed by switching microbiomes between bold and shy animals, to see if the behavioral traits also switch based on microbiome composition, similarly as the experiment carried out by Collins, Kassam and Bercik^105^, on mice. Such microbiome transplant methods are well-established in zebrafish models and could be adapted to sticklebacks^106^. Such future work, building on studies like ours, will improve our understanding of the complexity of the gut-brain axis across the animal clade.

## Methods

### Sampling and fieldwork

We collected adult stickleback with minnow traps from two high elevation lakes, spring-fed Galtaból and glacial Þristikla, on 14 June 2019, and transported them to the fish facility at Verið aquaculture station of Hólar University (Saudárkrókur, Iceland). We housed fish separately by population, with approximately 30 fish in each of three 19 l aerated buckets per population, at 12 °C, which is close to the temperature of the lakes. Fish were fed ad libitum once per day with frozen bloodworms. The experiment started on 21 June 2019 and ended on 13 July 2019. We randomly selected subjects in the mornings before feeding and moved them from their home tanks to ensure their guts were free of digesting food before behavioral trials and gut dissection. Only female fish were used for all experiments. The animal treatments were conducted in accordance with the Icelandic Food and Veterinary Authority (Matvælastofnum, MAST), and approved by Michigan State University Institutional Animal Care and Use Committee (IACUC, protocol numbers 05/18-077-99, 05/16-064-00, and 201900128). Permits to collect stickleback fish were granted by Fjállabak Nature Reserve and the Vantajökulsþjóðgarður National Park.

### Behavioral experiments

Adult sticklebacks were food deprived for one day and then individually recorded for behavior in an arena of 150 × 50 cm with a water depth of 21 cm and a distance from the camera of 155 cm. Water in the arena was clear, and so, similar to conditions for the spring-fed population of Galtaból. This was done primarily to allow us to see behaviors clearly and because the limited numbers of wild-caught fish available did not allow us to also explore the potential effects of turbidity on antipredator behavior. Prior work by our team on these and other Icelandic populations shows that glacial fish exhibit some plasticity in antipredator response to visual environment, becoming more similar to spring-fed fish^67^; therefore any differences we find in the present study suggest the population effect is robust. To minimize the potential accumulation of stress-related and other chemical cues, the arena was thoroughly cleaned between each individual trial. After each trial, the arena was drained and rinsed with spring water, drained and cleaned with ethanol, and rinsed again. Then, in preparation for a behavioral trial, the experimental arena was re-filled from the same water source as was used to fill holding tanks. This ensured that any residual cues were removed before testing the next fish.

The experiment had two predation treatments: predator-exposed and non-exposed. We simulated predator attack with a robotic trout that was programmed to attack at a constant speed and strike distance (see video & full description in Fig. 10). We also had a control group with no robotic predator exposure which we term the non-exposed group. For both groups, we started with 2 min of acclimation time, to allow the fish to adjust to the arena. We then recorded behaviors for 4 min to establish a baseline for behavior. Next, for the predator-exposed group, a simulated attack by the robotic predator was triggered when the stickleback fish passed in front of the robotic predator. After triggering the robotic predator, stickleback behavior was recorded for 8 min. For the non-exposed group, fish behavior was recorded for 8 min in the same way as the predator-exposed group but there was no robotic predator in the arena, therefore no predator cue to be triggered. For both treatments, the stickleback fish was measured and euthanized by decapitation following the EU Directive 2010/63/EU^107^. Immediately after being euthanized, the gut was extracted, stored in RNALater and frozen in liquid nitrogen for further analyses.

### Parasite identification

Parasites were identified by visual inspection during the dissection for gut extraction, focusing on a major stickleback parasite in the body cavity, *S. solidus*. Parasite prevalence was recorded as a binary variable (present/absent) and information about parasite abundance or diversity was not recorded. Parasitism prevalence was higher in Galtaból compared to Þristikla (27.3% vs 15.4%), and we could only determine the parasitism status of an individual after the behavioral trial was completed, resulting in an unbalanced study design when testing for associations with parasite status. Final sample numbers for the behavioral analyses were as follows: 40 Galtaból fish in the non-exposed group (30 non-parasitized, 10 parasitized), 48 Galtaból fish in the predator-exposed group (34 non-parasitized, 14 parasitized), 38 Þristikla fish in the non-exposed group (38 non-parasitized, 0 parasitized) and 40 Þristikla fish in the predator-exposed group (28 non-parasitized, 12 parasitized). Galtaból and Þristikla fish were similar in size, and measured 5.9 ± 0.6 cm and 5.5 ± 0.5 cm in length, respectively, with corresponding weights of 1.4 ± 0.7 g and 1.1 ± 0.5 g.

### Characterization of behavior: arena for non-exposed condition

**Fig. 9:**
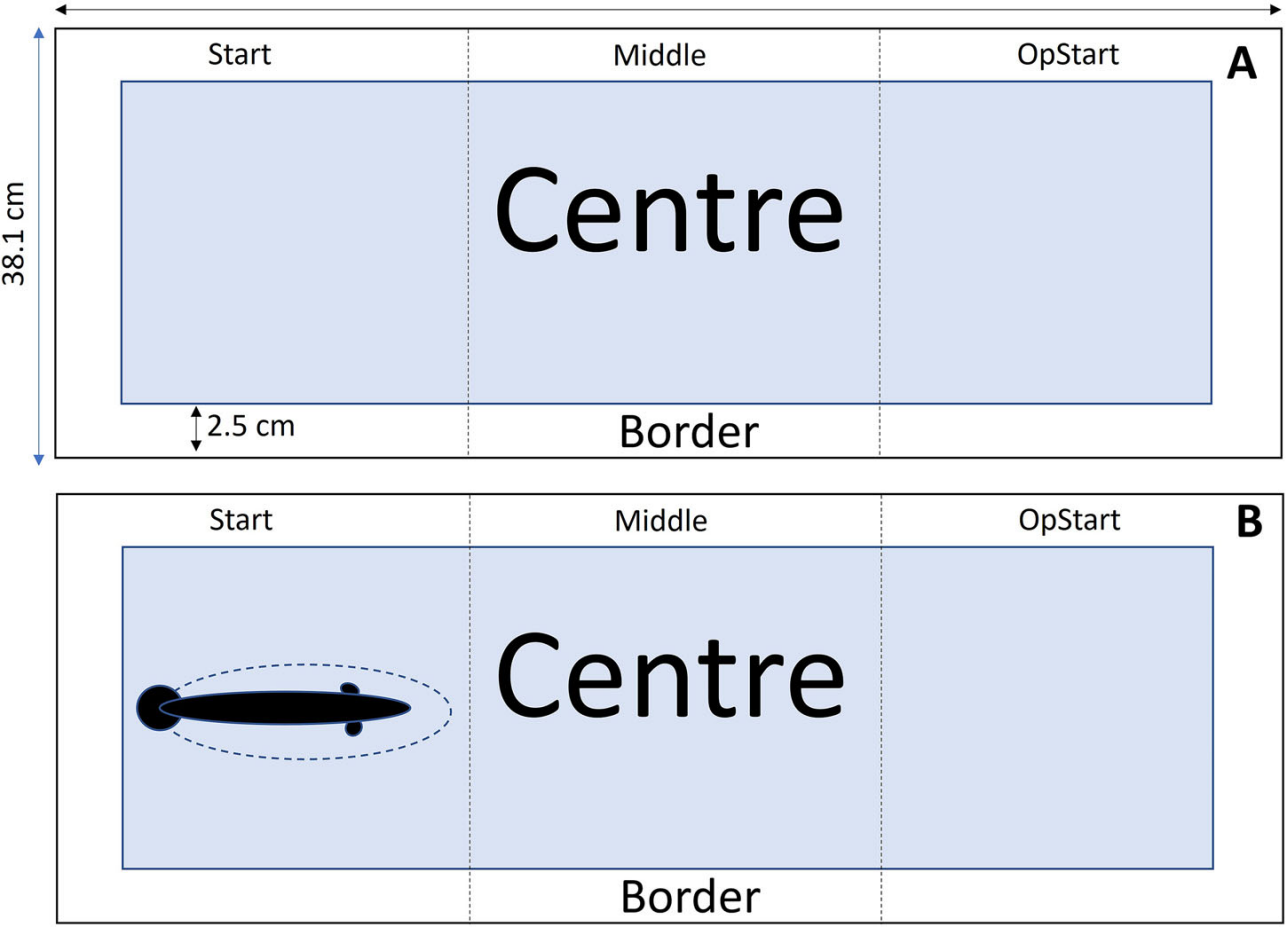
Delimitation of the virtual zones on the bottom of the arena for video analysis under two conditions. In the control condition (**A**), dotted lines define three equal-sized zones (Start, Middle, Opstart), with the blue rectangle indicating the Center zone and the Border representing the entire arena minus the Center zone. In the predator-exposed condition (**B**) (shown here before the robotic predator is triggered), the same zone layout is used. The robotic predator is positioned in the Start zone, with an additional hidden zone beneath it and an Entry zone marked by a dotted ellipse around the predator.

The arena was divided into five virtual zones (Fig. 9A) using the software EthoVision XT 15 (Noldus, The Netherlands), comprised of three equal-sized zones: Start (left part of the arena where the tested fish is placed at the beginning of experiment), Opstart (right part of the arena at the opposite side of start) and Middle (zone located between Start and OpStart); and two additional zones: Center (the center zone was considered a risky area, a common measure indicative of a high degree of boldness in such an apparatus) and Border (this zone is associated with thigmotaxis that is staying close to the walls of an arena, a common measure indicative of a high degree of shyness in such an apparatus)^108^. This zone width was 2.5 cm corresponding to approximately half of the total length of the mean value for both populations. The variables of interest extracted with EthoVision XT 15 were as follows: (i) the time spent in each zone previously described, (ii) the distance traveled by each fish in the device (Dtot in mm), and (iii) the absolute angular velocity of the fish expressed in degrees per second (Vang in ° s − 1) and its mean velocity expressed in body length per second (Vel in BL s − 1).

### Characterization of behavior: arena for treatment condition

**Fig. 10:**
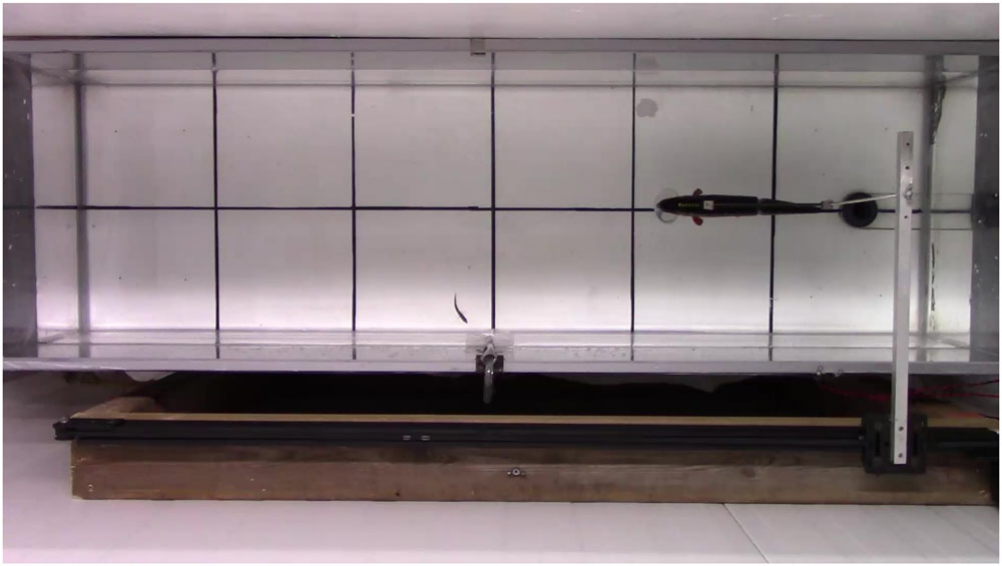
Video 59 of the predator-exposed treatment of the Þristikla population, non-parasitized. The video was trimmed to show the moment when the robotic predator was triggered. The camera was set to 160p recording in P mode, with stabilization deactivated. The file format is AVCHD, and the accepted SD card format is exFAT. The robotic predator is constructed using a motor and a belt system, designed so that the tail of the silicone model aligns with the tank’s siphon and stops before reaching the end of the rail.

Two reversed arena settings were designed in Ethovision for the situations before triggering the robotic predator, when the robotic predator is on the left, and after triggering the robotic predator, when it has moved to the right (see video in Fig. 10). So, we only describe the situation before triggering the predator (Fig. 9B).

The same five zones as described for the non-exposed treatment were also used here. In addition to these zones, we defined Pred (hidden zone under the robotic predator) after we observed that the tested fish could hide under the robotic predator once it was triggered and Entry (zone located all around the robotic predator). The variables of interest extracted with EthoVision XT 15 were as follows: (i) the time spent in each zone previously described, (ii) the time spent in Pred and Entry, (iii) the distance traveled by each fish in the device (Dtot in mm), and (iv) the absolute angular velocity of the fish expressed in degrees per second (Vang in ° s − 1) and its mean velocity expressed in body length per second (Vel in BL s − 1).

### Nucleic acids extraction, library preparation and sequencing

Due to limitations in funding, a subset of individuals were randomly selected for microbiota analysis, aiming for similar numbers of individuals per population and treatment. All parasitized Þristikla individuals were excluded, due to the low parasite prevalence in this population. For nucleic acid extraction, whole guts previously stored in RNALater were homogenized and lysed with a TissueLyser for 1.5 min at 25–30 Hz in 1.6 ml Buffer RLT (Qiagen). DNA and RNA were then simultaneously extracted from 450 µl of lysate per sample using the Allprep® DNA/RNA Mini kit (Qiagen, ID/No:80204) following the manufacturer’s protocol. The extractions were performed in six batches and each batch included a blank negative control containing only Buffer RLT, which were taken through all stages of library preparation and sequencing.

A total of 71 DNA gut extracts and all six extraction blank controls were submitted to SciLifeLab Uppsala for quality control, library preparation and sequencing. During quality control, nine gut extracts were flagged as having evidence of DNA degradation (low DNA concentrations and/or skewed DNA fragment distributions). These samples were excluded from further processing. TruSeq Nano DNA libraries (Illumina) were prepared and sequenced by SciLifeLab Uppsala on two lanes of a Illumina NovaSeq 6000 SP flowcell using paired-end 150 bp read length v1 sequencing chemistry. Thus, we obtained high quality sequencing data from 62 individuals: 18 Galtaból fish in the non-exposed group (13 non-parasitized, 5 parasitized), 19 Galtaból fish in the predator-exposed group (11 non-parasitized, 8 parasitized), 15 Þristikla fish in the non-exposed group (all non-parasitized) and 10 Þristikla fish in the predator-exposed group (all non-parasitized).

### DNA Sequence data processing, taxonomic assignment and taxa filtering

Adapter and quality trimming of sequenced DNA reads was performed using AdapterRemoval v.2.2.4^109^, trimming consecutive bases with quality scores of <30 and removing reads <50 bp after trimming. Paired reads from both lanes were concatenated into a single fasta file per sample. Reads with mean base quality <30 and exact PCR duplicates (original or reverse complement) were filtered out with PrinSeq-Lite v0.20.4^110^. Reads resulting from the phage PhiX, a positive control spiked in during Illumina sequencing, were removed by mapping to the PhiX reference genome (GCF_000819615.1) with bwa mem v0.7.17^111^. The unmapped reads were retained with SAMTools v1.9 (Li et al. 2009) and BEDTools v2.27.1^112^ downstream analyses. In the same manner, reads from human contamination were removed by mapping to the *Homo sapiens* reference genome (GCF_000001405.38)^113^ and reads from the host stickleback were removed by mapping to the *G. aculeatus* reference genome (GCF_016920845.1)^114^.

Microbial taxonomic assignment was then performed on the unmapped, non-host reads, using the *k*-mer based classifier Kraken2 v2.0.8^115^ with the standard Kraken2 database (all archaea, bacteria, viruses and the human genome in RefSeq; built 2020-10-01) and default parameters. Bracken v2.0^116^ was used to estimate taxa abundances from the Kraken read assignments at the species level (-l S) using a read length of 150 bp (-r 150), a k-mer length of 35 bp (-k 35) and without an abundance threshold (-t 0). Kraken-biom (https://github.com/smdabdoub/kraken-biom) was used to extract the summarized abundances assigned at the species levels.

Quality control was then performed on the taxa abundances in RStudio v402 using R v4.3.1. To reduce noise, taxa present at <0.05% relative abundance (Bracken abundance divided by sum of Bracken abundance in a sample) were filtered out. The community compositions of the blank control samples were then compared to the stickleback gut samples, with the aim to identify and remove putative laboratory contaminants. All six negative blank controls included in the DNA extractions had low numbers of microbial reads (mean: 403, range: 113–722). One blank had a microbial community more similar to the fish gut samples than the other blanks (Supplementary Fig. 1). Since there was no clustering by extraction batch (Supplementary Fig. 2), we determined that low-level cross-contamination between samples and the blank had occurred during this extraction batch. We therefore excluded this blank from contamination identification. Using the other five blanks, contaminants were identified using the decontam ‘prevalence’ function^117^ with the default threshold of 0.1, resulting in 134 taxa identified as contaminants and removed from the dataset. Using the decontam ‘frequency’ function^117^ with DNA extraction concentration, an additional 38 taxa were identified and removed. The final taxa abundances were summarized at the genus-level for subsequent statistical analysis.

### Statistical analysis of Behavior

We first investigated (1) whether behavior was different between the two populations among non-exposed fish and (2) whether the presence of a predator affects behavior differently between stickleback populations, using data from the non-exposed treatment and the fish’s activity during the acclimation period in the predator exposure (i.e., before the robotic predator was triggered). This comparison was analyzed using a linear model. First, to test the swimming activity, we used Total Distance Swum, Velocity, and Angular Velocity as response variables. Second, to test tank (arena) use, Center Duration was the response variable, given that it is inherently opposite to Border Duration, making separate analysis unnecessary. For both swimming activity and tank use, the fixed factors included Population (spring-fed Galtaból and glacial Þristikla), Treatment (non-exposed and predator-exposed) and their interaction (Population by Treatment). The interaction term tests one of our hypotheses: whether behavioral responses to predators vary by population, so we retain it in our models even when nonsignificant. Posthoc Tukey tests were used using the emmeans R package^118^ to test for differences between treatments. This analysis is a post hoc of multiple comparison test on the Population by treatment interaction^119^ to assess only biologically meaningful pairwise differences between populations, i.e., Galtaból non-exposed vs Þristikla non-exposed; Galtaból predator-exposed vs Þristikla predator-exposed; Galtaból predator-exposed vs Galtaból predator-exposed; Þristikla predator-exposed vs Þristikla predator-exposed.

To further test the differences in behavior under the predator exposure, a linear mixed effect model was performed in R with lme4 package^120^. Total Distance swum, Velocity, and Angular Velocity were used as response variables of swimming activity. Center duration and Distance to predator were also used as response variables to test fish boldness. Population (Galtaból and Þristikla), Period (before and after triggering the robotic predator), and Parasite (presence vs absence) and their interactions were used as fixed factors. Again, we retain interaction terms because they test important hypotheses of whether predator response varies with either population or parasitism status. The individual fish ID was used as a random effect factor because of our repeated measures design. Diagnostics based on residuals of the model were performed to assess compliance with the underlying assumptions. A contrast list post hoc test was performed as previously described by using the R package emmeans^118^. This analysis is a multiple comparison post hoc test on the population by Period by Parasite interaction^119^ to assess only biologically meaningful pairwise differences between populations, Period and Parasite (full results reported in Supplementary Tables 1-2).

The dependent variables were transformed whenever necessary to ensure that the residuals followed the assumed error distribution. Transformed variables can be observed in Supplementary Data 1, 2 and 3. Finally, the effects of the independent variables were estimated from the models and their significance was tested by likelihood ratio tests (LRT) between models, respecting the marginality of the effects that are supposed to follow a chi-2 distribution under the null hypothesis (type III tests; car R package^121^). All behavioral analysis were run in R version 4.4.2^122^.

### Statistical analysis of microbiome

All microbiome statistical analyses were performed in RStudio v2024.12.0 using R v4.4.1^122^. Alpha diversity metrics were calculated from Braken relative abundances using the Hill numbers framework with the R package hillR^123^, using *q* = 0 to estimate richness and *q* = 1 to estimate the Shannon index. Statistically significant differences (*p* < 0.05) in alpha diversity metrics between groups were evaluated using Wilcoxon rank-sum tests implemented in the R package ggsignif^124^. For unsupervised analyses, Bracken abundance counts were normalized by the center-log ratio (CLR) transformation, using a pseudocount of 1 added to all taxa in all samples to resolve the problem of zero values. Euclidean distance matrices were calculated from the CLR-transformed data with the phyloseq^125^ function distance. Principal Coordinates Analysis (PCoA) was performed using the phyloseq function ordinate, using Euclidean distances. Permutational multivariate analysis of variance (PERMANOVA) was performed on the Euclidean distances using the adonis2 function in the R package vegan v.2.6–2 (https://github.com/vegandevs/vegan), including the following as covariates: sequencing depth (total Braken abundance counts in a sample), extraction date, length of individual fish, Population or Parasite (depending on the comparison), and treatment (non-exposed or predator-exposed). As there was still an effect of extraction batch on our community composition data (Supplementary Fig. 3, Supplementary Table 3), we used limma’s function removeBatchEffect^126^ to regress out the DNA extraction batch effect from the normalized data and repeated the ordination and PERMANOVA as described.

General linear models implemented through MaAsLin2^127^ were used to identify genera with significantly different relative abundance (calculated via total sum scaling) between (1) non-parasitized Galtaból and Þristikla individuals and (2) non-parasitized and parasitized Galtaból individuals. In both models, Treatment (non-exposed vs predator-exposed) was included as a fixed effect while extraction batch was included as a random effect. MaAsLin2 was run without additional normalization or transformation steps and otherwise default parameters. Genera with adjusted p-values (MaAsLin2 q-value) <0.05 were classed as significantly differentially abundant.

To integrate the behavioral and microbiome datasets, multi-omic factor analysis (MOFA) was performed using the R package MOFA2^128^. MOFA is similar to PCA, where matrices of different types of data generated from the same individuals are reduced to a small number of latent factors representing the key contributors of variation across the datasets^128^. In this study, MOFA was performed on the non-exposed individuals and predator-exposed individuals separately, since the predator-exposed fish included behavioral variables not relevant for the non-exposed control fish (distance to and duration around the predator stimulus). For the behavioral dataset, all variables were included, both before and after the simulated predator attack. The behavioral variables were converted to approximate a normal distribution using the inverse normal transformation. For the microbiome dataset, the 100 most variable genera in each MOFA run (non-exposed and predator-exposed) were included, using CLR-transformed abundances after regressing out the extraction batch effect (as described for PCoA). Both MOFA models were trained with 7 factors, using Gaussian distributions for both datasets, scaling each dataset to have similar variances and otherwise using default values. After the MOFA models were trained, the function correlate_factors_with_covariates in the MOFA2 package was used to identify factors that were significantly correlated (alpha = 0.05) with metadata variables (fish population, parasite status, length of individual and sample extraction batch). Factors that explained >0.75% of the variation in both datasets were investigated further. The top 10 features (behavioral variables and microbial genera) contributing to the variation captured by each factor (5 with positive weights and 5 with negative weights) were extracted and classified.

## Supplementary information

Supplementary information

Supplementary data

## Data Availability

All data supporting this manuscript is available. Raw metagenomic sequencing data is available at the European Nucleotide Archive (ENA) under project PRJEB52754 [link: https://www.ebi.ac.uk/ena/browser/view/PRJEB52754]. ENA accessions for all samples are provided in Supplementary Data 4. Sample metadata, including behavioral traits extracted from the videos, are also provided in Supplementary Data 4. Behavioral traits extracted from the videos for behavior statistical analysis are provided in Supplementary Data 2 and 3.

## Code availability

All data supporting this manuscript is available. All R code for behavior statistical analysis is provided in Supplementary Data 1. All R code for figures, microbiome, and microbiome–behavior analysis is provided in Supplementary Data 5.

## References

[1] Schulte, PM. What is environmental stress? Insights from fish living in a variable environment.*J Exp Biol* **217**, 23–34, 10.1242/jeb.089722 (2014) 24353201 10.1242/jeb.089722

[2] Ning, D. et al. Environmental stress mediates groundwater microbial community assembly. *Nat. Microbiol.* **9**, 490–501 (2024). 38212658 10.1038/s41564-023-01573-x

[3] Fakan, E. P., Allan, B. J. M., Illing, B., Hoey, A. S. & McCormick, M. I. Habitat complexity and predator odours impact on the stress response and antipredation behaviour in coral reef fish. *PLoS ONE* **18**, e0286570 (2023). 10.1371/journal.pone.0286570 PMC10306203 37379294

[4] Jenkins, M. R. et al. Natural and anthropogenic sources of habitat variation influence exploration behaviour, stress response, and brain morphology in a coastal fish. *J. Anim. Ecol.* **90**, 2446–2461 (2021). 34143892 10.1111/1365-2656.13557

[5] Abrahams, M. V., Mangel, M. & Hedges, K. Predator-prey interactions and changing environments: Who benefits?. *Philos. Trans. R. Soc. B: Biol. Sci.* **362**, 2095–2104 (2007). 10.1098/rstb.2007.2102 PMC2442855 17472922

[6] Liu, Q. et al. Fish predation risk alters the microbiota of Daphnia in the process of inducing its life-history defence traits. *Freshw. Biol.* **69**, 591–605 (2024).

[7] Zha, Y., Eiler, A., Johansson, F. & Svanbäck, R. Effects of predation stress and food ration on perch gut microbiota. *Microbiome* **6**, 28 (2018). 29409543 10.1186/s40168-018-0400-0 PMC5801810

[8] Sadeq, S. A., Mills, R. I. L. & Beckerman, A. P. The microbiome mediates the interaction between predation and heavy metals. *Sci. Total Environ.* **775**, 145144 (2021). 33631565 10.1016/j.scitotenv.2021.145144

[9] Foster, K. R., Schluter, J., Coyte, K. Z. & Rakoff-Nahoum, S. The evolution of the host microbiome as an ecosystem on a leash. *Nature* **548**, 43–51 (2017). 28770836 10.1038/nature23292 PMC5749636

[10] Foster, J. A., Rinaman, L. & Cryan, J. F. Stress & the gut-brain axis: Regulation by the microbiome.*Neurobiol Stress* **7**, 124–136, 10.1016/j.ynstr.2017.03.001 (2017). 29276734 10.1016/j.ynstr.2017.03.001 PMC5736941

[11] Johnson, K. V. A. & Foster, K. R. Why does the microbiome affect behaviour?. *Nat. Rev. Microbiol.* **16**, 647–655 (2018). 29691482 10.1038/s41579-018-0014-3

[12] Eklöv, P. & Svanbäck, R. Predation risk influences adaptive morphological variation in fish populations. *Am. Naturalist* **167**, 440–452 (2006). 16673351 10.1086/499544

[13] Lapiedra, O., Schoener, T. W., Leal, M., Losos, J. B. & Kolbe, J. J. Predator-driven natural selection on risk-taking behavior in anole lizards. *Science* **360**, 1017–1020 (2018). 29853685 10.1126/science.aap9289

[14] Rödl, T., Berger, S., Romero, L. M. & Wikelski, M. Tameness and stress physiology in a predator-naive island species confronted with novel predation threat. *Proc. R. Soc. B: Biol. Sci.* **274**, 577–582 (2007). 10.1098/rspb.2006.3755 PMC1766385 17476779

[15] Reznick, D., Butler Iv, M. J. & Rodd, H. Life-history evolution in guppies. VII The comparative ecology of high- and low-predationenvironments. *Am Nat* **157**, 126–40, 10.1086/318627 (2001). 18707267 10.1086/318627

[16] Endler, J. A. Multiple-trait coevolution and environmental gradients in guppies. *Trends Ecol. Evol.* **10**, 22–29 (1983). 10.1016/s0169-5347(00)88956-9 21236940

[17] Milinski, M. & Heller, R. Influence of a predator on optimal foraging behaviour of sticklebacks. *Nature* **275**, 642–644 (1978).

[18] Fraser, D. F. & Gilliam, J. F. Nonlethal Impacts of Predator Invasion : Facultative Suppression of Growth and Reproduction. *Ecology* **73**, 959–970 (1992).

[19] Diehl, S., Eklöv, P., Diehl, S. & Eklov, P. Effects of Piscivore-mediated habitat use on resources, diet, and growth of perch. *Ecology.* **76**, 1712–1726 (1995).

[20] Brown, G. E. & Smith, R. J. F. Acquired predator recognition in juvenile rainbow trout (Oncorhynchus mykiss): conditioning hatchery-reared fish to recognize chemical cues of a predator. *Can. J. Fish. Aquat. Sci.* **55**, 611–617 (1998).

[21] Lehtiniemi, M. Swim or hide: predator cues cause species specific reactions in young fish larvae. *J. Fish. Biol.* **66**, 1285–1299 (2005).

[22] Kopack, C. J., Dale Broder, E., Lepak, J. M., Fetherman, E. R. & Angeloni, L. M. Behavioral responses of a highly domesticated, predator naïve rainbow trout to chemical cues of predation. *Fish. Res.* **169**, 1–7 (2015).

[23] Hall, A. E. & Clark, T. D. Seeing is believing: metabolism provides insight into threat perception for a prey species of coral reef fish. *Anim. Behav.* **115**, 117–126 (2016).

[24] Cerri, R. D. The effect of light intensity on predator and prey behaviour in cyprinid fish: factors that influence prey risk. *Anlm. Behav.* **31**, 736–742 (1983).

[25] Lima, S. L. & Dill, L. M. Behavioral decisions made under the risk of predation: a review and prospectus. *Can. J. Zool.* **68**, 619–640 (1980).

[26] Godin, J. G. J. & Valdron Clark, K. A. Risk-taking in stickleback fishes faced with different predatory threats 1. *Ecoscience* **4**, 246–251 (1997).

[27] Jolles, J. W. et al. Both prey and predator features predict the individual predation risk and survival of schooling prey. *Elife* **11**, e76344 (2022). 35852826 10.7554/eLife.76344 PMC9348852

[28] Lambert, P. J., Herbert-Read, J. E. & Ioannou, C. C. The measure of spatial position within groups that best predicts predation risk depends on group movement. *Proc. R. Soc. B* **288**, 20211286 (2021). 34521249 10.1098/rspb.2021.1286 PMC8441135

[29] Näslund, J., Pettersson, L. & Johnsson, J. I. Behavioural reactions of three-spined sticklebacks to simulated risk of predation—Effects of predator distance and movement. *FACETS* **1**, 55–66 (2017).

[30] Huntingford, F. & Giles, N. Individual variation in anti-predator responses in the three-spined stickleback (Gasterosteus aculeatus L.). *Ethology* **74**, 205–210 (1987).

[31] Bergstrom, C. A. Fast-start swimming performance and reduction in lateral plate number in threespine stickleback. *Can. J. Zool.* **80**, 207–213 (2002).

[32] Bush, A. O., Fernández, J. C., Esch, G. W. & Seed, J. R. *Parasitism: The Diversity and Ecology of Animal Parasites* (Cambridge University Press, 2001).

[33] Tiddy, I. C., Schneider, K. & Elmer, K. R. Environmental correlates of adaptive diversification in postglacial freshwater fishes. *J Fish Biol.* **104**, 517–535, 10.1111/jfb.15621 (2024). 37984834 10.1111/jfb.15621

[34] Poulin, R. Adaptive’ changes in the behaviour of parasitized animals: a critical review. *Int. J. Parasitol.* **25**, 1371–1383 (1995). 8719948 10.1016/0020-7519(95)00100-x

[35] Thomas, F., Adamo, S. & Moore, J. Parasitic manipulation: where are we and where should we go?. *Behav. Process.* **68**, 185–199 (2005). 10.1016/j.beproc.2004.06.010 15792688

[36] Barber, I., Hoare, D. & Krause, J. Effects of parasites on fish behaviour: a review and evolutionary perspective. *Rev. Fish. Biol. Fish.* **10**, 131–165 (2000).

[37] Jolles, J. W., Mazué, G. P. F., Davidson, J., Behrmann-Godel, J. & Couzin, I. D. Schistocephalus parasite infection alters sticklebacks’ movement ability and thereby shapes social interactions. *Sci. Rep.* **10**, 12282 (2020). 32703965 10.1038/s41598-020-69057-0 PMC7378215

[38] Grécias, L., Valentin, J. & Aubin-Horth, N. Testing the parasite mass burden effect on alteration of host behaviour in the Schistocephalus-stickleback system. *J. Exp. Biol.* **221**, jeb174748 (2018). 29444843 10.1242/jeb.174748

[39] Demandt, N. et al. Parasite infection disrupts escape behaviours in fish shoals: parasite infection disrupts escape waves. *Proc. R. Soc. B: Biol. Sci.* **287**, 20201158 (2020). 10.1098/rspb.2020.1158 PMC7735259 33143588

[40] Barber, I. Parasites, behaviour and welfare in fish. *Appl. Anim. Behav. Sci.* **104**, 251–264 (2007).

[41] LoBue, C. P. & Bell, M. A. Phenotypic manipulation by the cestode parasite schistocephalus solidus of its intermediate host, gasterosteus aculeatus, the threespine stickleback. *Am. Nat.* **142**, 725–735 (1993). 19425968 10.1086/285568

[42] Giles, N. Behavioural effects of the parasite Schistocephalus solidus (Cestoda) on an intermediate host, the three-spined stickleback, Gasterosteus aculeatus L. *Anim. Behav.* **31**, 1192–1194 (1983).

[43] Blake, R. W., Kwok, P. Y. L. & Chan, K. H. S. Effects of two parasites, Schistocephalus solidus (Cestoda) and Bunodera spp. (Trematoda), on the escape fast-start performance of three-spined sticklebacks. *J. Fish. Biol.* **69**, 1345–1355 (2006).

[44] Allan, B. J. M. et al. Parasite infection directly impacts escape response and stress levels in fish. *J. Exp. Biol.* **223**, jeb230904 (2020). 10.1242/jeb.230904 32611788

[45] Hahn, M. A. et al. Host phenotype and microbiome vary with infection status, parasite genotype, and parasite microbiome composition. *Mol. Ecol.* **31**, 1577–1594 (2022). 35000227 10.1111/mec.16344

[46] Gaulke, C. A. et al. A longitudinal assessment of host-microbe-parasite interactions resolves the zebrafish gut microbiome’s link to Pseudocapillaria tomentosa infection and pathology. *Microbiome* **7**, 10 (2019). 30678738 10.1186/s40168-019-0622-9 PMC6346533

[47] Marchesi, J. R. et al. The gut microbiota and host health: a new clinical frontier. *Gut* 1–10 10.1136/gutjnl-2015-309990 (2015). 10.1136/gutjnl-2015-309990 PMC4752653 26338727

[48] Varg, J. E., Bergvall, C. & Svanbäck, R. The stressful effects of microplastics associated with Chromium (VI) on the microbiota of daphnia magna. *Front. Environ. Sci.* **10**, 1–13 (2022).

[49] Bailey, M. T. et al. Exposure to a social stressor alters the structure of the intestinal microbiota: implications for stressor-induced immunomodulation. *Brain Behav. Immun.* **25**, 397–407 (2011). 21040780 10.1016/j.bbi.2010.10.023 PMC3039072

[50] Collins, S. M. & Bercik, P. The relationship between intestinal microbiota and the central nervous system in normal gastrointestinal function and disease. *Gastroenterology* **136**, 2003–2014 (2009). 19457424 10.1053/j.gastro.2009.01.075

[51] Vuong, H. E., Yano, J. M., Fung, T. C. & Hsiao, E. Y. The microbiome and host behavior. 10.1146/annurev-neuro-072116 (2017). 10.1146/annurev-neuro-072116-031347 PMC6661159 28301775

[52] Davis, D. J. et al. Lactobacillus plantarum attenuates anxiety-related behavior and protects against stress-induced dysbiosis in adult zebrafish. *Sci. Rep.* **6**, 1–11 (2016). 27641717 10.1038/srep33726 PMC5027381

[53] White, E. C. et al. Manipulation of host and parasite microbiotas: survival strategies during chronic nematode infection. *Sci. Adv.* **4**, 1–10 (2018). 10.1126/sciadv.aap7399 PMC5851687 29546242

[54] Plieskatt, J. L. et al. Infection with the carcinogenic liver fluke Opisthorchis viverrini modifies intestinal and biliary microbiome. *FASEB J.* **27**, 4572–4584 (2013). 23925654 10.1096/fj.13-232751 PMC3804743

[55] Jaenike, J., Unckless, R., Cockburn, S. N., Boelio, L. M. & Perlman, S. J. Adaptation via symbiosis: recent spread of a drosophila defensive symbiont. *Science* **329**, 212–215 (2010). 20616278 10.1126/science.1188235

[56] Williams, A. R., Myhill, L. J. & Stolzenbach, S. Emerging interactions between diet, gastrointestinal helminth infection, and the gutmicrobiota in livestock. *BMC Veterinary Research.* **1**, 62, 10.1186/s12917-021-02752-w (2021). 10.1186/s12917-021-02752-w PMC7845040 33514383

[57] Reynolds, L. A., Finlay, B. B. & Maizels, R. M. Cohabitation in the intestine: interactions among helminth parasites, bacterial microbiota, and host immunity. *J. Immunol.* **195**, 4059–4066 (2015). 26477048 10.4049/jimmunol.1501432 PMC4617609

[58] Landmann, F., Voronin, D., Sullivan, W. & Taylor, M. J. Anti-filarial activity of antibiotic therapy is due to extensive apoptosis after Wolbachia depletion from filarial nematodes. *PLoS Pathog.* **7**, e1002351 (2011). 22072969 10.1371/journal.ppat.1002351 PMC3207916

[59] Jorge, F., Dheilly, N. M. & Poulin, R. Persistence of a core microbiome through the ontogeny of a multi-host parasite. *Front. Microbiol.* **11**, 954 (2020). 32508779 10.3389/fmicb.2020.00954 PMC7248275

[60] Brealey, J. C. et al. Microbiome “Inception”: an intestinal cestode shapes a hierarchy of microbial communities nested within the host. *mBio* **13**, e00679–22 (2022). 10.1128/mbio.00679-22 PMC9239044 35502903

[61] Kashinskaya, E. N. et al. Trophic diversification and parasitic invasion as ecological niche modulators for gut microbiota of whitefish. *Front. Microbiol.* **14**, 1090899 (2023). 36998403 10.3389/fmicb.2023.1090899 PMC10043260

[62] Vaughan, J. A., Tkach, V. V. & Greiman, S. E. Neorickettsial endosymbionts of the digenea: diversity, transmission and distribution. In *Advances in Parasitology*253–297 (Academic Press, 2012). 10.1016/B978-0-12-398457-9.00003-2 22726644

[63] Dessì, D., Rappelli, P., Diaz, N., Cappuccinelli, P. & Fiori, P. L. Mycoplasma hominis and trichomonas vaginalis: a unique case of symbiotic relationship between two obligate human parasites. *Front. Biosci*. **11**, 2028 (2006). 10.2741/1944 16720288

[64] Deenonpoe, R. et al. The carcinogenic liver fluke Opisthorchis viverrini is a reservoir for species of Helicobacter. *Asian Pac. J. Cancer Prev.* **16**, 1751–1758 (2015). 25773821 10.7314/apjcp.2015.16.5.1751 PMC4945248

[65] Bell, M. A. & Foster, S. A. The evolutionary biology of the threespine stickleback. *J. Anim. Ecol.* **64**, 418 (1994).

[66] Giles, N. & Huntingford, F. A. Predation risk and inter-population variation in anti-predator behaviour in the three-spined stickleback, Gasterosteus aculeatus L. *Anim. Behav*. **32**, 264–275 (1984).

[67] Ålund, M. et al. Sensory environment affects Icelandic threespine stickleback’s anti-predator escape behaviour. *Proc. R. Soc. B: Biol. Sci.* **289**, 20220044 (2022). 10.1098/rspb.2022.0044 PMC8984813 35382599

[68] Young, R. E. & MacColl, A. D. C. Spatial and temporal variation in macroparasite communities of three-spined stickleback. *Parasitology* **144**, 436–449 (2016). 27762183 10.1017/S0031182016001815

[69] Rennison, D. J., Rudman, S. M. & Schluter, D. Parallel changes in gut microbiome composition and function during colonization, local adaptation and ecological speciation. *Proc. R. Soc. B: Biol. Sci.* **286**, 20191911 (2019). 10.1098/rspb.2019.1911 PMC6939261 31795865

[70] Härer, A., Mauro, A. A., Laurentino, T. G., Rosenblum, E. B. & Rennison, D. J. Gut microbiota parallelism and divergence associated with colonisation of novel habitats. *Mol. Ecol.* **32**, 5661–5672 (2023). 37715531 10.1111/mec.17135

[71] Smith, C. C. R., Snowberg, L. K., Gregory Caporaso, J., Knight, R. & Bolnick, D. I. Dietary input of microbes and host genetic variation shape among-population differences in stickleback gut microbiota. *ISME J.* **9**, 2515–2526 (2015). 25909977 10.1038/ismej.2015.64 PMC4611514

[72] Ling, F. et al. The gut microbiota response to helminth infection depends on host sex and genotype. *ISME J.* **14**, 1141–1153 (2020). 32005978 10.1038/s41396-020-0589-3 PMC7174316

[73] Ajemian, M. J., Sohel, S. & Mattila, J. Effects of turbidity and habitat complexity on antipredator behavior of three-spined sticklebacks (Gasterosteus aculeatus): antipredator behavior in sticklebacks. *Environ. Biol. Fishes* **98**, 45–55 (2015).

[74] Laspoumaderes, C. et al. Glacier melting and stoichiometric implications for lake community structure: Zooplankton species distributions across a natural light gradient. *Glob. Chang Biol.* **19**, 316–326 (2013). 23504742 10.1111/gcb.12040

[75] Lucek, K., Kristjánsson, B. K., Skúlason, S. & Seehausen, O. Ecosystem size matters: the dimensionality of intralacustrine diversification in Icelandic stickleback is predicted by lake size. *Ecol Evol* **6**, 5256–5272 (2016). 10.1002/ece3.2239 PMC4984502 27551381

[76] Shankregowda, A. M. et al. Host habitat rather than evolutionary history explains gut microbiome diversity in sympatric stickleback species. *Front. Microbiol.* **14**, 1232358 (2023). 10.3389/fmicb.2023.1232358 PMC10601471 37901806

[77] Barber, I., Walker, P. & Svensson, P. A. Behavioural responses to simulated avian predation in female three spined sticklebacks: the effect of experimental Schistocephalus solidus infections. *Behaviour* **141**, 1425–1440 (2004).

[78] Barber, I., Mora, A. B., Payne, E. M., Weinersmith, K. L. & Sih, A. Parasitism, personality and cognition in fish. *Behav. Process*. **141**, 205–219 Preprint at 10.1016/j.beproc.2016.11.012 (2017). 10.1016/j.beproc.2016.11.012 27894933

[79] Leahy, S. M., McCormick, M. I., Mitchell, M. D. & Ferrari, M. C. O. To fear or to feed: the effects of turbidity on perception of risk by a marine fish. *Biol. Lett.* **7**, 811–813 (2011). 21849308 10.1098/rsbl.2011.0645 PMC3210690

[80] Thorburn, D. M. J. et al. Context-dependent parasite infection affects trophic niche in populations of sympatric stickleback species. *Parasitology* **149**, 1164–1172 (2022). 35570701 10.1017/S0031182022000531 PMC10090597

[81] Bolnick, D. I. et al. Individuals’ diet diversity influences gut microbial diversity in two freshwater fish (threespine stickleback and Eurasian perch). *Ecol. Lett.* **17**, 979 (2014). 24847735 10.1111/ele.12301 PMC4084827

[82] Restivo, V. E., Kidd, K. A., Surette, M. G., Bucking, C. & Wilson, J. Y. The gut content microbiome of wild-caught rainbow darter is altered during laboratory acclimation. *Comp. Biochem Physiol. Part D. Genom. Proteom.* **39**, 100835 (2021). 10.1016/j.cbd.2021.100835 33894530

[83] Viver, T. et al. Food determines ephemerous and non-stable gut microbiome communities in juvenile wild and farmed Mediterranean fish. *Sci. Total Environ.* **889**, 164080 (2023). 10.1016/j.scitotenv.2023.164080 37201821

[84] Beaz-Hidalgo, R. & Figueras, M. J. Aeromonas spp. whole genomes and virulence factors implicated in fish disease. *J. Fish. Dis.* **36**, 371–388 (2013). 23305319 10.1111/jfd.12025

[85] Biebl, H. et al. Description of Labrenzia alexandrii gen. nov., sp. nov., a novel alphaproteobacterium containing bacteriochlorophyll a, and a proposal reclassification of Stappia aggregata as Labrenzia aggregata comb. nov., and of Stappia alba as Labrenzia alba comb. nov., and emended descriptions of the genera Pannonibacter, Stappia and Roseibium, and of the species Roseibium denhamense and Roseibium hamelinense. *Int. J. Syst. Evol. Microbiol.* **57**, 1095–1107 (2007). 17473266 10.1099/ijs.0.64821-0

[86] Gim, D. H. et al. Description of Deefgea piscis sp. nov., and Deefgea tanakiae sp. nov., isolated from the gut of Korean indigenous fish. *J. Microbiol.* **60**, 1061 (2022). 36048329 10.1007/s12275-022-2250-5 PMC9433522

[87] Ōmura, S., Otoguro, K., Nishikiori, T., Ōiwa, R. & Iwai, Y. Setamycin, A New Antibiotic. *J. Antibiot.* **34**, 1253–1256 (1981). 10.7164/antibiotics.34.1253 7309621

[88] Takahashi, Y. Genus Kitasatospora, taxonomic features and diversity of secondary metabolites. *J. Antibiotics* **70**, 506–513 Preprint at 10.1038/ja.2017.8 (2017). 10.1038/ja.2017.8 28196972

[89] Sih, A., Bell, A. & Johnson, J. C. Behavioral syndromes: an ecological and evolutionary overview. *Trends Ecol. Evol*. **19**, 372–378 Preprint at 10.1016/j.tree.2004.04.009 (2004). 10.1016/j.tree.2004.04.009 16701288

[90] Biro, P. A. & Stamps, J. A. Are animal personality traits linked to life-history productivity?. *Trends Ecol. Evol.* **23**, 361–368 (2008). 18501468 10.1016/j.tree.2008.04.003

[91] Puetz, L. C. et al. Gut microbiota linked with reduced fear of humans in red Junglefowl has implications for early domestication. *Adv. Genet.* **2**, 2100018 (2021). 36619855 10.1002/ggn2.202100018 PMC9744516

[92] Schretter, C. E. et al. A gut microbial factor modulates locomotor behaviour in Drosophila. *Nature* **563**, 402–406 (2018). 30356215 10.1038/s41586-018-0634-9 PMC6237646

[93] Schretter, C. E. Links between the gut microbiota, metabolism, and host behavior. *Gut Microbes* 11, 245–248 Preprint at 10.1080/19490976.2019.1643674 (2020). 10.1080/19490976.2019.1643674 PMC7053934 31345081

[94] Archie, E. A. & Tung, J. Social behavior and the microbiome. *Curr. Opin. Behav. Sci*. **6**, 28–34 Preprint at 10.1016/j.cobeha.2015.07.008 (2015).

[95] Ayayee, P. A. & Wong, R. Y. Zebrafish (Danio rerio) behavioral phenotypes not underscored by different gut microbiota. *BioRxiv*10.1101/2024.05.29.596447 (2024). 10.1002/ece3.70237 PMC11362613 39219576

[96] Barton, B. A. Stress in fishes: a diversity of responses with particular reference to changes in circulating corticosteroids. *Integrat. Comparative Biol*. **42** http://icb.oxfordjournals.org/ (2002). 10.1093/icb/42.3.517 21708747

[97] Harris, B. N. & Carr, J. A. The role of the hypothalamus-pituitary-adrenal/interrenal axis in mediating predator-avoidance trade-offs. *Gen. Compar. Endocrinol.* **230**, 110–142 Preprint at 10.1016/j.ygcen.2016.04.006 (2016). 10.1016/j.ygcen.2016.04.006 27080550

[98] Fuess, L. E. et al. Immune gene expression covaries with gut microbiome composition in stickleback. *mBio* **12**, 10–1128 (2021). 10.1128/mBio.00145-21 PMC8262870 33947750

[99] Lyte, J. M., Koester, L. R., Daniels, K. M. & Lyte, M. Distinct cecal and fecal microbiome responses to stress are accompanied by sex- and diet-dependent changes in behavior and gut serotonin. *Front. Neurosci.* **16**, 827343 (2022). 10.3389/fnins.2022.827343 PMC9039258 35495029

[100] Awe, T. et al. The modulatory role of gut microbiota on host behavior: exploring the interaction between the brain-gut axis and the neuroendocrine system. *AIMS Neurosci*. **11**, 49–62 Preprint at 10.3934/NEUROSCIENCE.2024004 (2024). 10.3934/Neuroscience.2024004 PMC11007408 38617041

[101] Liang, S. et al. Administration of Lactobacillus helveticus NS8 improves behavioral, cognitive, and biochemical aberrations caused by chronic restraint stress. *Neuroscience* **310**, 561–577 (2015). 26408987 10.1016/j.neuroscience.2015.09.033

[102] Smith, C. J. et al. Probiotics normalize the gut-brain-microbiota axis in immunodeficient mice. *Am. J. Physiol. Gastrointest. Liver Physiol.* **307**, G793–G802 (2014). 25190473 10.1152/ajpgi.00238.2014 PMC4200314

[103] Rosshart, S. P. et al. Wild mouse gut microbiota promotes host fitness and improves disease resistance. *Cell* **171**, 1015–1028.e13 (2017). 29056339 10.1016/j.cell.2017.09.016 PMC6887100

[104] Nyholm, L. et al. Holo-Omics: integrated host-microbiota multi-omics for basic and applied biological research. *iScience* **23**, 101414 (2020). 32777774 10.1016/j.isci.2020.101414 PMC7416341

[105] Collins, S. M., Kassam, Z. & Bercik, P. The adoptive transfer of behavioral phenotype via the intestinal microbiota: experimental evidence and clinical implications. *Curr. Opin. Microbiol*. **16**, 240–245, Preprint at 10.1016/j.mib.2013.06.004 (2013). 10.1016/j.mib.2013.06.004 23845749

[106] Cornuault, J. K., Byatt, G., Paquet, M. E., De Koninck, P. & Moineau, S. Zebrafish: a big fish in the study of the gut microbiota. *Curr. Opin. Biotechnol.* **73**, 308–313 (2022). 34653834 10.1016/j.copbio.2021.09.007

[107] Valentim, A. M., van Eeden, F. J., Strähle, U. & Olsson, I. A. S. Euthanizing zebrafish legally in Europe. *EMBO Rep.* **17**, 1688–1689 (2016). 27797854 10.15252/embr.201643153 PMC5283582

[108] Dahlbom, S. J., Lagman, D., Lundstedt-Enkel, K., Sundström, L. F. & Winberg, S. Boldness predicts social status in zebrafish (Danio rerio). *PLoS ONE* **6**, e23565 (2011). 10.1371/journal.pone.0023565 PMC3157393 21858168

[109] Schubert, M., Lindgreen, S. & Orlando, L. AdapterRemoval v2: rapid adapter trimming, identification, and read merging. *BMC Res. Notes* **9**, 88 (2016). 26868221 10.1186/s13104-016-1900-2 PMC4751634

[110] Schmieder, R. & Edwards, R. Quality control and preprocessing of metagenomic datasets. *Bioinformatics* **27**, 863–864 (2011). 21278185 10.1093/bioinformatics/btr026 PMC3051327

[111] Li, H. Aligning sequence reads, clone sequences and assembly contigs with BWA-MEM. *arXiv preprint* arXiv:1303.3997 10.1186/s13756-018-0352-y (2013).

[112] Quinlan, A. R. & Hall, I. M. BEDTools: a flexible suite of utilities for comparing genomic features. *Bioinformatics* **26**, 841–842 (2010). 20110278 10.1093/bioinformatics/btq033 PMC2832824

[113] Schneider, V. A. et al. Evaluation of GRCh38 and de novo haploid genome assemblies demonstrates the enduring quality of the reference assembly. *Genome Res.* **27**, 849–864 (2017). 28396521 10.1101/gr.213611.116 PMC5411779

[114] Nath, S., Shaw, D. E. & White, M. A. Improved contiguity of the threespine stickleback genome using long-read sequencing. *G3 Genes|Genomes|Genetics* **11**, jkab007 (2021). 10.1093/g3journal/jkab007 PMC8022941 33598708

[115] Wood, D. E., Lu, J. & Langmead, B. Improved metagenomic analysis with Kraken 2. *Genome Biol.* **20**, 1–13 (2019). 31779668 10.1186/s13059-019-1891-0 PMC6883579

[116] Lu, J., Breitwieser, F. P., Thielen, P. & Salzberg, S. L. Bracken: estimating species abundance in metagenomics data. *PeerJ Comput. Sci.* **3**, e104 (2017). 40271438 10.7717/peerj-cs.104 PMC12016282

[117] Davis, N. M., Proctor, D. M., Holmes, S. P., Relman, D. A. & Callahan, B. J. Simple statistical identification and removal of contaminant sequences in marker-gene and metagenomics data. *Microbiome* **6**, 226 (2018). 30558668 10.1186/s40168-018-0605-2 PMC6298009

[118] Lenth, R. V. et al. emmeans: estimated marginal means, aka Least-Squares Means. *R package version 1.4.6.* https://cran.r-project.org/package=emmeans (2020).

[119] Hothorn, T., Hornik, K., Van De Wiel, M. A. & Zeileis, A. A lego system for conditional inference. *Am. Stat.* **60**, 257–263 (2006).

[120] Bates, D., Mächler, M., Bolker, B. M. & Walker, S. C. Fitting linear mixed-effects models using lme4. *J Stat. Softw* **67**, 1–48 (2015).

[121] Fox, J. & Weisberg, S. *An R Companion to Applied Regression* (Sage, Thousand Oaks, 2019).

[122] R Core Team. A language and environment for statistical computing. R Foundation for Statistical Computing, Vienna, Austria. (2023).

[123] Alberdi, A. & Gilbert, M. T. P. A guide to the application of Hill numbers to DNA-based diversity analyses. *Mol. Ecol. Resour.* **19**, 804–817 (2019). 30947383 10.1111/1755-0998.13014

[124] Ahlmann-Eltze, C. & Patil, I. ggsignif: R Package for Displaying Significance Brackets for ‘ggplot2’. *PsyArxiv*10.31234/osf.io/7awm6 (2021)

[125] McMurdie, P. J. & Holmes, S. phyloseq: an R Package for reproducible interactive analysis and graphics of microbiome census data. *PLoS ONE* **8**, e61217 (2013). 23630581 10.1371/journal.pone.0061217 PMC3632530

[126] Ritchie, M. E. et al. limma powers differential expression analyses for RNA-sequencing and microarray studies. *Nucleic Acids Res.* **43**, e47 (2015). 25605792 10.1093/nar/gkv007 PMC4402510

[127] Mallick, H. et al. Multivariable association discovery in population-scale meta-omics studies. *PLoS Comput. Biol.* **17**, e1009442 (2021). 34784344 10.1371/journal.pcbi.1009442 PMC8714082

[128] Argelaguet, R. et al. Multi-Omics factor analysis—a framework for unsupervised integration of multi-omics data sets. *Mol. Syst. Biol.* **14**, 8124 (2018). 10.15252/msb.20178124 PMC6010767 29925568

