# Supplementary material for: Interplay between antipredator behavior, parasitism, and gut microbiome in wild stickleback populations
Source: NPJ Biofilms Microbiomes. 2025 Jul 19;11:138. doi: 10.1038/s41522-025-00758-y (PMC12276351; doi:10.1038/s41522-025-00758-y)
Supplement: Supplementary file 1 — Supplementary information [file 41522_2025_758_MOESM1_ESM.pdf]

Supplementary Information

Interplay between antipredator behavior, parasitism, and gut microbiome in wild stickleback populations.

Javier Edo Varg [*], Jaelle C. Brealey, David Benhaïm, Rafael Losada-Germain, Janette W. Boughman

*Corresponding author: Javier Edo Varg
Email: jedovarg@gmail.com
Telephone: +46760791049

Supplementary Figures

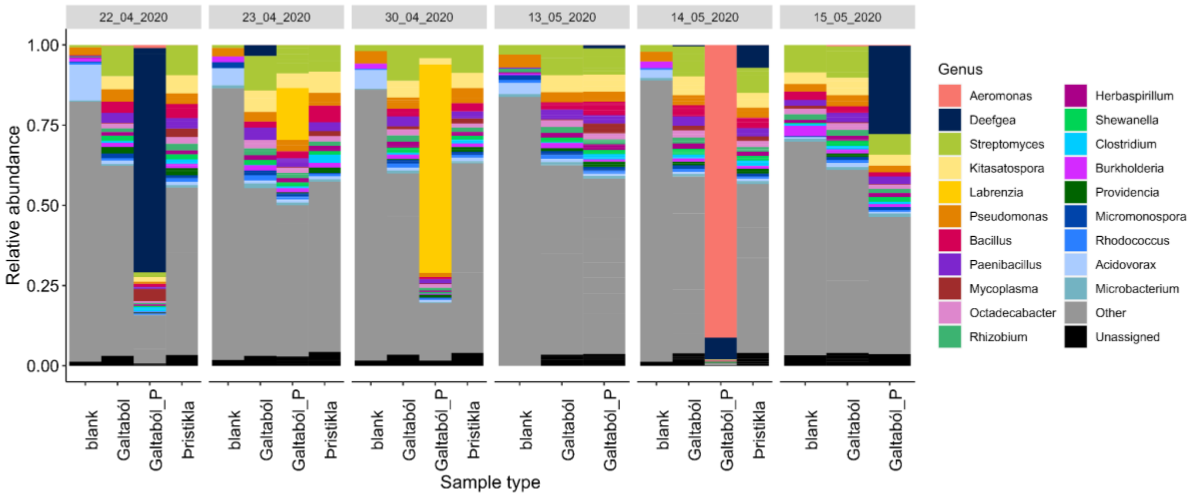

Supplementary Figure 1. Microbial composition of blanks and gut samples before filtering of contaminating taxa. Samples are pooled by population and parasite status within each extraction batch date (shown as the facet). The composition of the blank negative control sample for each extraction batch is also shown. Galtaból_P indicates parasitized Galtaból individuals. The 20 most abundant genera are shown, the rest are grouped as “Other”. Species-level taxa that could not be assigned at the genus level are grouped as ‘Unassigned’.

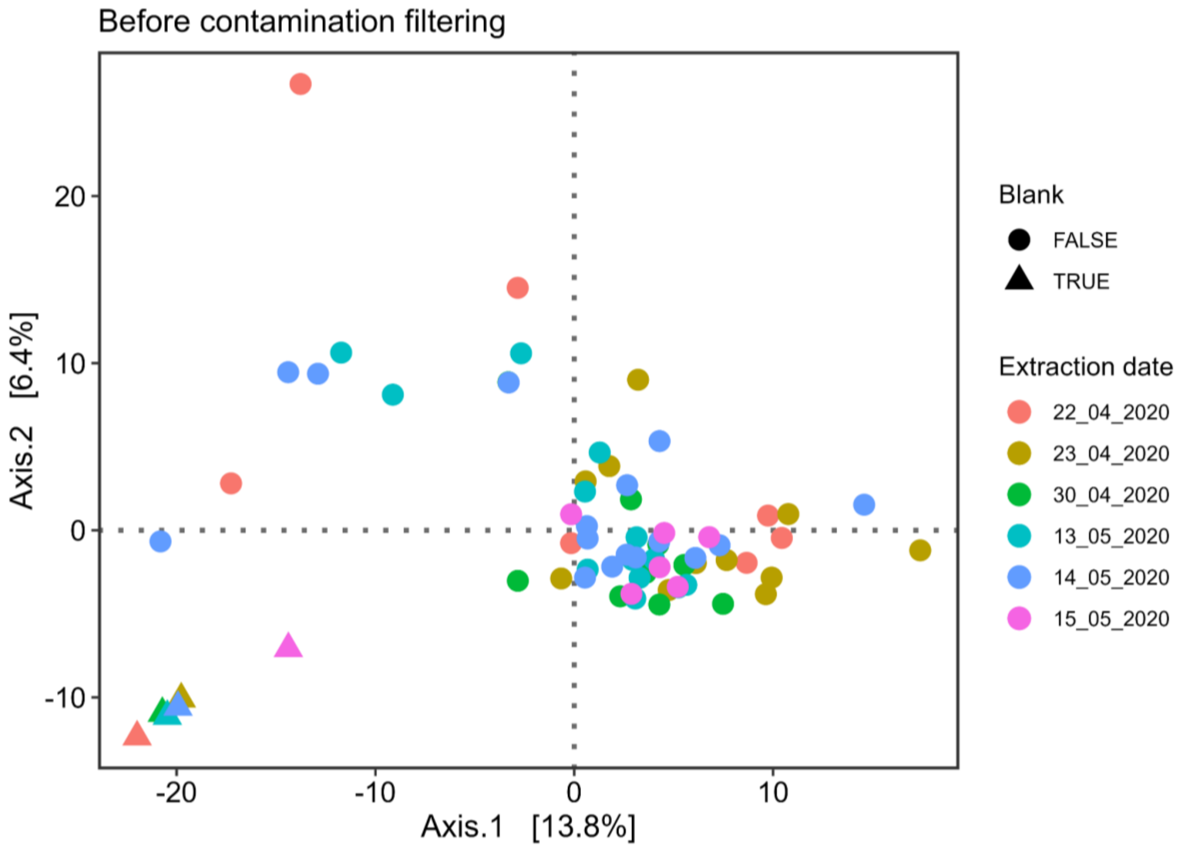

Supplementary Figure 2. PCoA of microbiome composition of blanks and gut samples before filtering of contaminating taxa. Samples are colored by extraction batch date. Blank samples are indicated by triangles and fish gut samples by circles. Taxa abundances at the species-level were normalized using the centered-log ratio transformation. Euclidean distances were then calculated, from which the PCoA was generated.

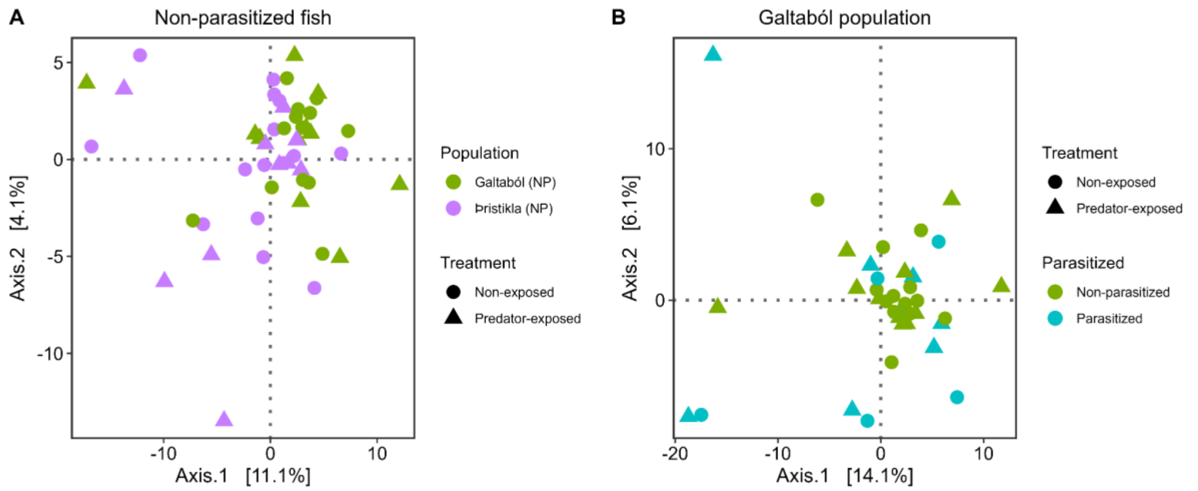

Supplementary Figure 3. PCoA of gut microbiome composition of non-parasitized (NP) spring-fed Galtaból and glacial Þristikla populations (**A**) and non-parasitized and parasitized Galtaból individuals (**B**). Shape ("treatment") indicates whether the fish were used in the control non-exposed experiment (circles) or the predator-exposed experiment (triangles). Taxa abundances were summed to the genus-level and normalized using the centered-log ratio transformation. Euclidean distances were then calculated and PERMANOVAs performed. Population in A explained 3.2% of the variation in microbiome composition ($F = 1.66$, $p = 0.004$), while parasite status in B explained 2.9% of the variation ($F = 1.09$, $p = 0.226$). In both analyses, extraction batch explained a greater proportion of variation (in A: 11.8%, $F = 1.22$, $p = 0.005$; in B: 14.3%, $F = 1.10$, $p = 0.150$), while sequencing depth explained 5.2–7.6% of the variation in microbiome composition ($p = 0.001$ in both) Full PERMANOVA results are reported in Supplementary Table S3.

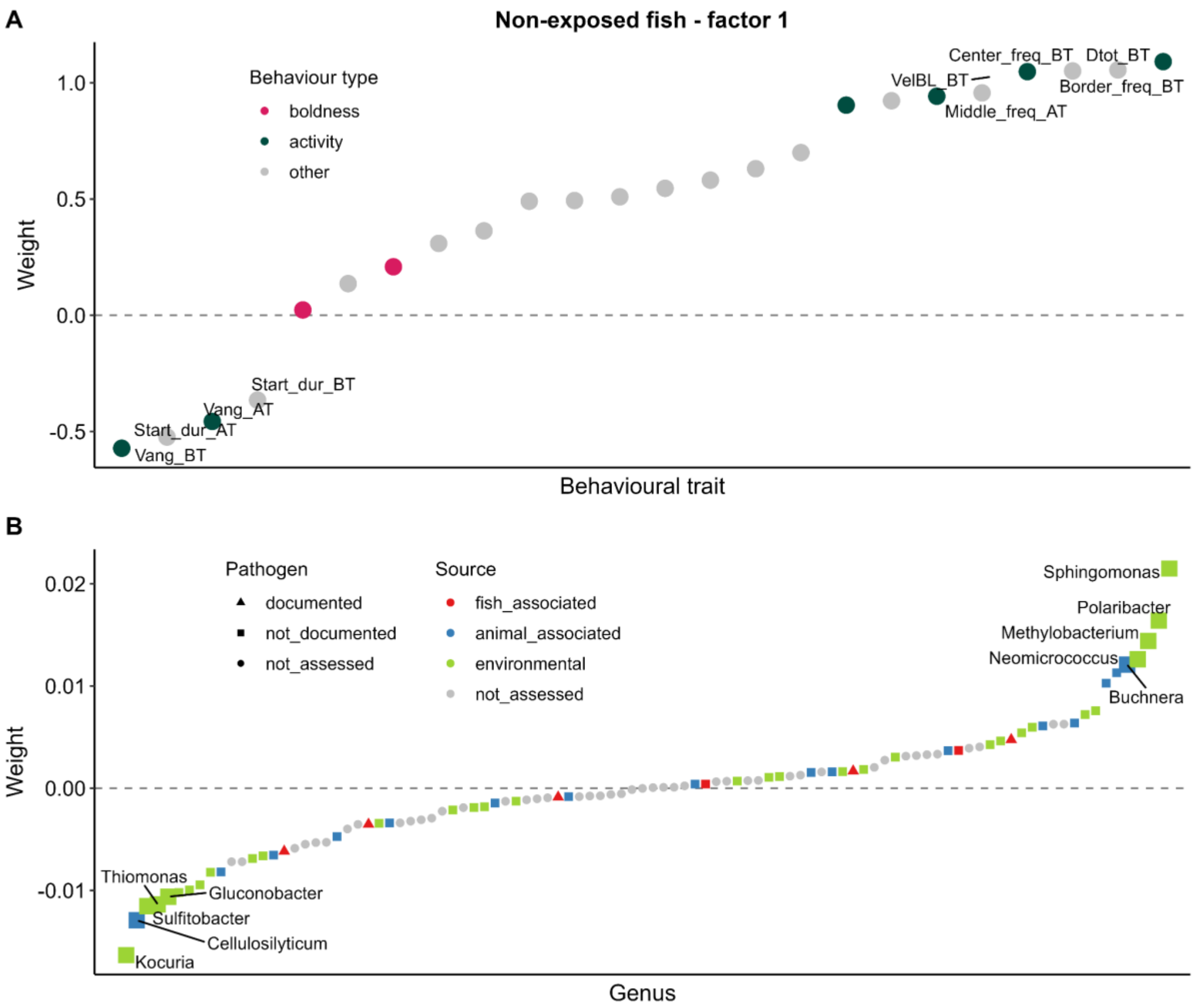

Supplementary Figure 4. Associations identified by MOFA in **non-exposed control fish** among behavioral traits and microbial abundances, for **factor 1**, which explained 48% of the variance in the behavioral dataset and 0.2% of variance in the microbiome dataset. **A)** Contribution (weight) of behavioral traits to the factor. Traits are ranked according to their weight. The higher the absolute weight, the more strongly associated a trait is with the factor. A positive weight indicates the trait has higher levels in samples with positive factor values, while a negative weight indicates the opposite. Behavioral traits are coloured by their broader behavioral category. The top five traits contributing to the factor in each direction are labeled. **B)** Contribution (weight) of microbial genera to the factor, displayed in the same way as for A. Microbial genera are coloured by their putative source and shaped on their potential as a fish pathogen, based on a literature search.

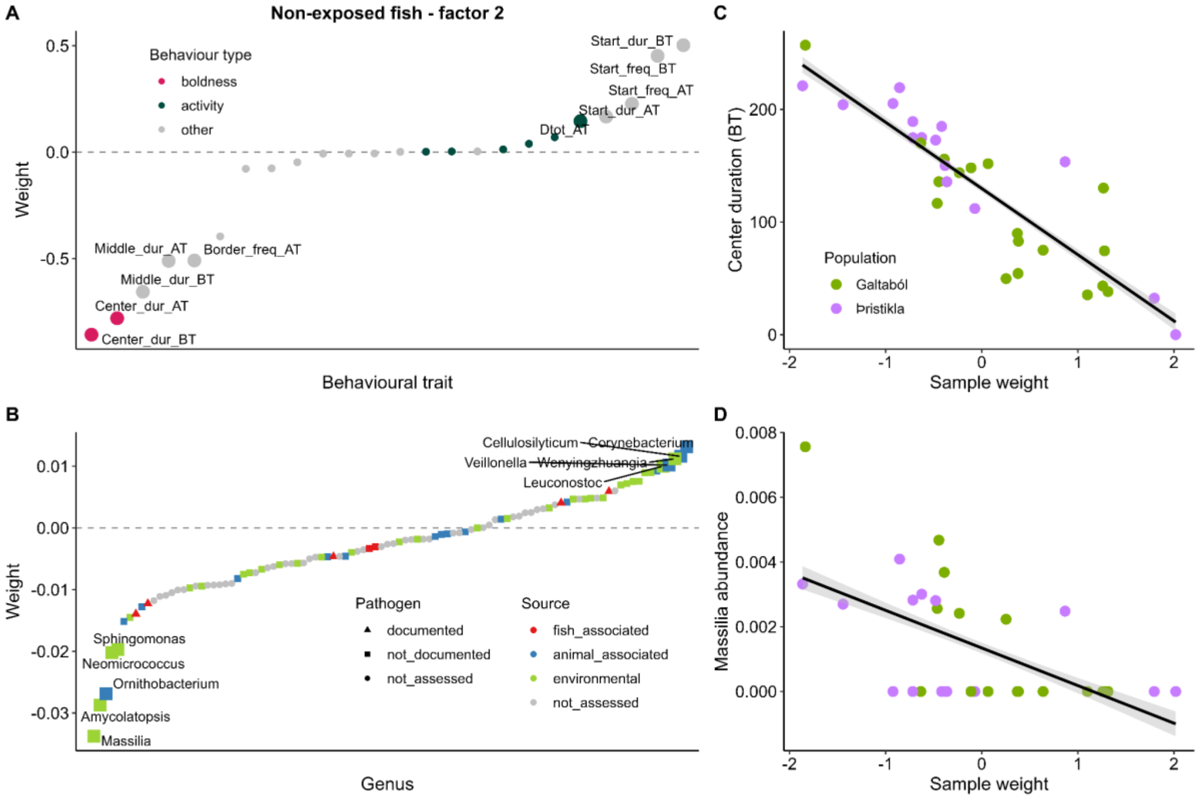

Supplementary Figure 5. Associations identified by MOFA in **non-exposed control fish** among behavioral traits and microbial abundances, for **factor 2**, which explained 16% of the variance in the behavioral dataset and 0.26% of variance in the microbiome dataset. **A)** Contribution (weight) of behavioral traits to the factor. Traits are ranked according to their weight. The higher the absolute weight, the more strongly associated a trait is with the factor. A positive weight indicates the trait has higher levels in samples with positive factor values, while a negative weight indicates the opposite. Behavioral traits are coloured by their broader behavioral category. The top five traits contributing to the factor in each direction are labeled. **B)** Contribution (weight) of microbial genera to the factor, displayed in the same way as for A. Microbial genera are coloured by their putative source and shaped on their potential as a fish pathogen, based on a literature search. **C)** Example association between factor 2 sample weights vs a top behavioral trait, center duration (BT). Samples are colored by population. A smoothed linear conditional means line is shown in black with confidence intervals in grey. **D)** Example association between factor 2 sample weights vs a top environmental microbial genus, *Massilia*, displayed in the same way as for C.

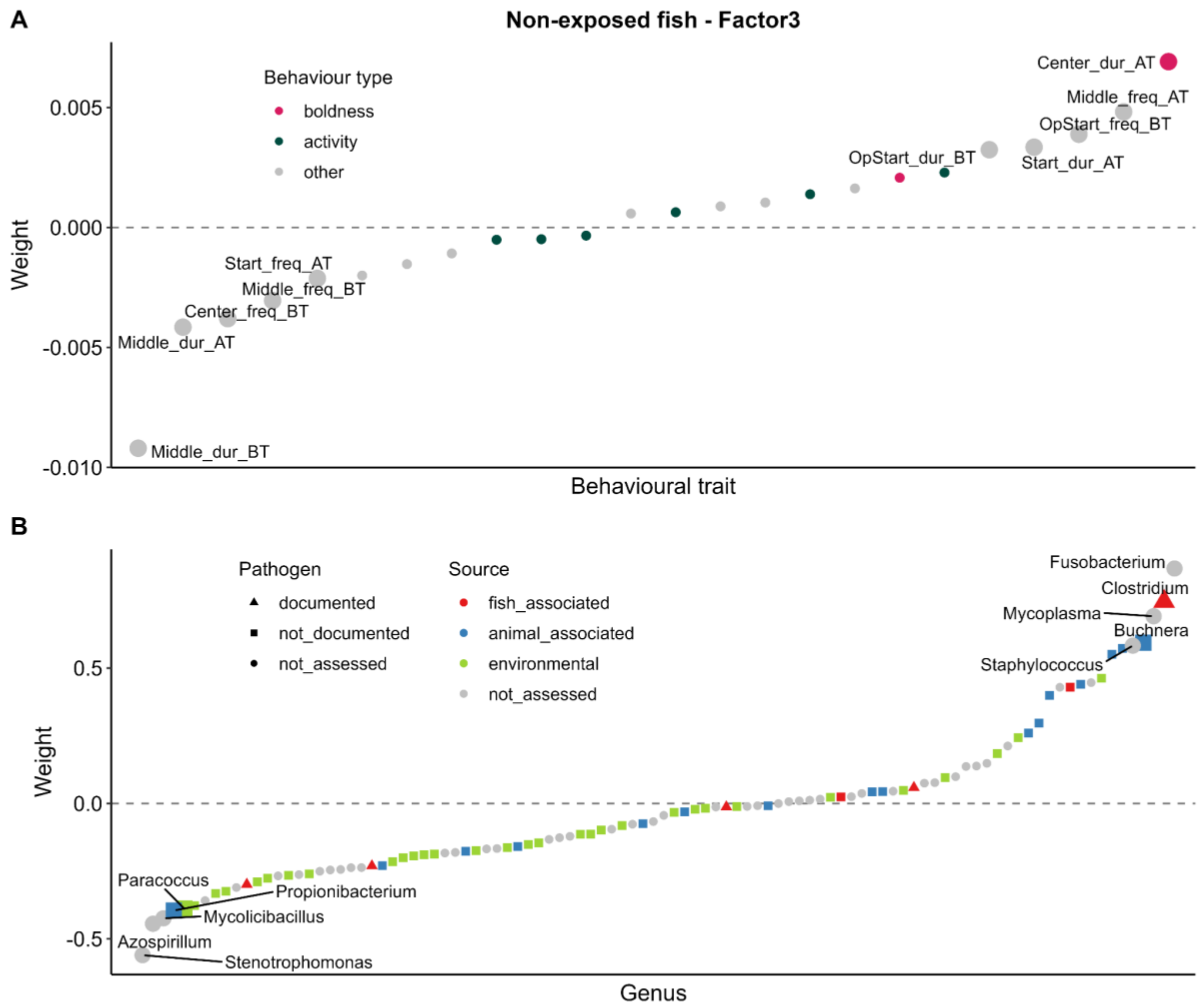

Supplementary Figure 6. Associations identified by MOFA in **non-exposed control fish** among behavioral traits and microbial abundances, for **factor 3**, which explained 0.06% of the variance in the behavioral dataset and 12% of variance in the microbiome dataset. **A)** Contribution (weight) of behavioral traits to the factor. Traits are ranked according to their weight. The higher the absolute weight, the more strongly associated a trait is with the factor. A positive weight indicates the trait has higher levels in samples with positive factor values, while a negative weight indicates the opposite. Behavioral traits are coloured by their broader behavioral category. The top five traits contributing to the factor in each direction are labeled. **B)** Contribution (weight) of microbial genera to the factor, displayed in the same way as for A. Microbial genera are coloured by their putative source and shaped on their potential as a fish pathogen, based on a literature search.

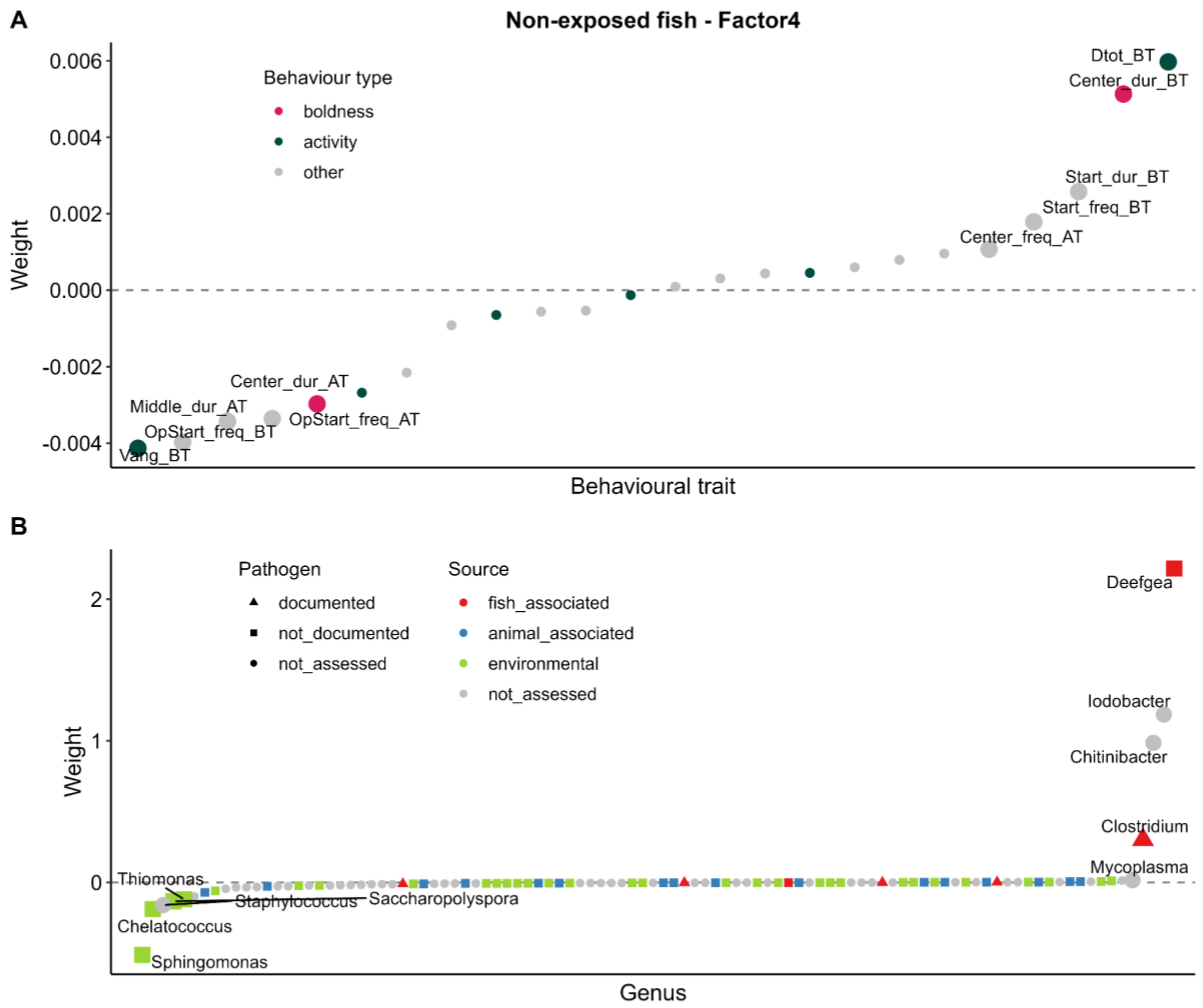

Supplementary Figure 7. Associations identified by MOFA in **non-exposed control fish** among behavioral traits and microbial abundances, for **factor 4**, which explained 0.06% of the variance in the behavioral dataset and 9% of variance in the microbiome dataset. **A)** Contribution (weight) of behavioral traits to the factor. Traits are ranked according to their weight. The higher the absolute weight, the more strongly associated a trait is with the factor. A positive weight indicates the trait has higher levels in samples with positive factor values, while a negative weight indicates the opposite. Behavioral traits are coloured by their broader behavioral category. The top five traits contributing to the factor in each direction are labeled. **B)** Contribution (weight) of microbial genera to the factor, displayed in the same way as for A. Microbial genera are coloured by their putative source and shaped on their potential as a fish pathogen, based on a literature search.

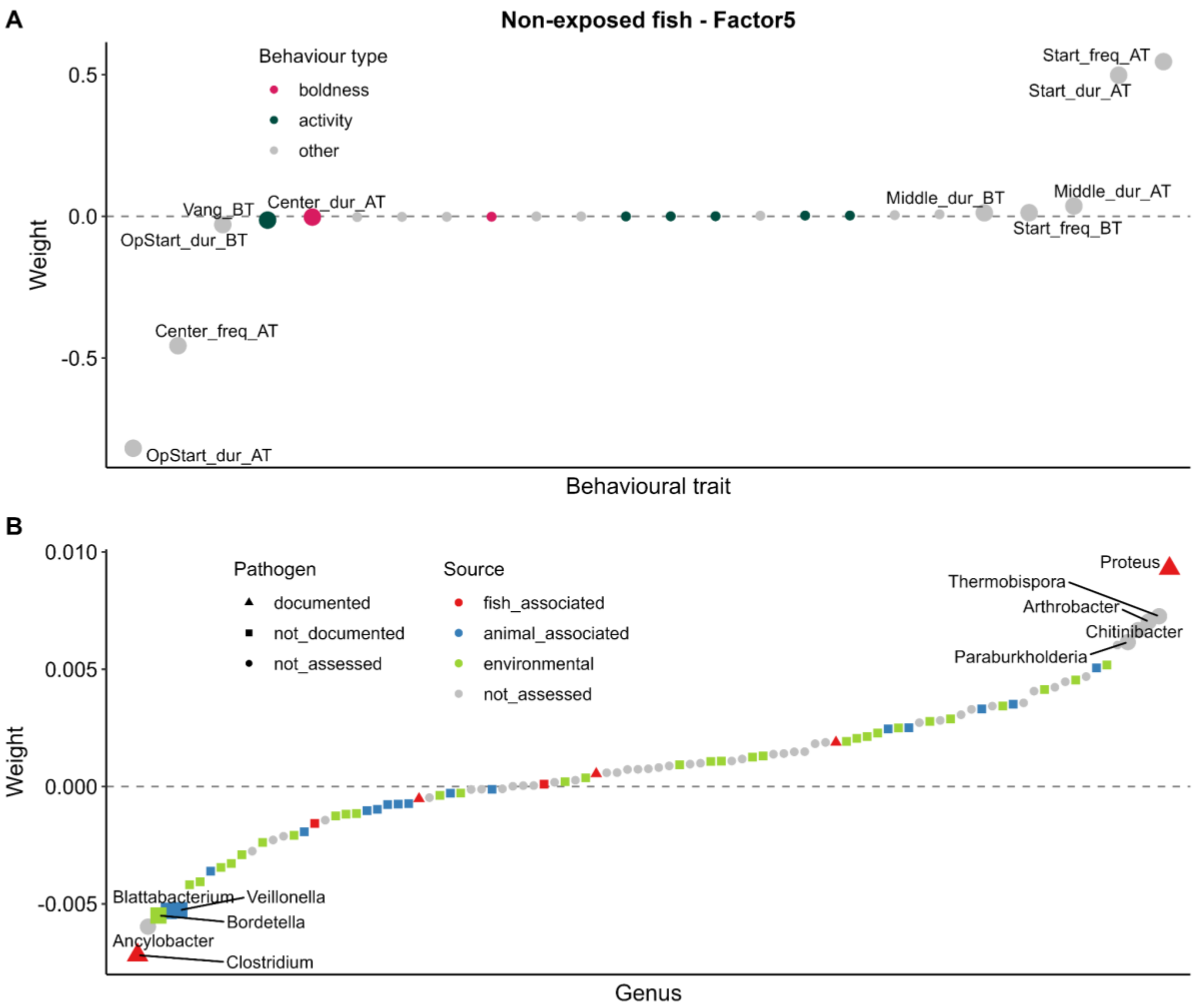

Supplementary Figure 8. Associations identified by MOFA in **non-exposed control fish** among behavioral traits and microbial abundances, for **factor 5**, which explained 8% of the variance in the behavioral dataset and 0.08% of variance in the microbiome dataset. **A)** Contribution (weight) of behavioral traits to the factor. Traits are ranked according to their weight. The higher the absolute weight, the more strongly associated a trait is with the factor. A positive weight indicates the trait has higher levels in samples with positive factor values, while a negative weight indicates the opposite. Behavioral traits are coloured by their broader behavioral category. The top five traits contributing to the factor in each direction are labeled. **B)** Contribution (weight) of microbial genera to the factor, displayed in the same way as for A. Microbial genera are coloured by their putative source and shaped on their potential as a fish pathogen, based on a literature search.

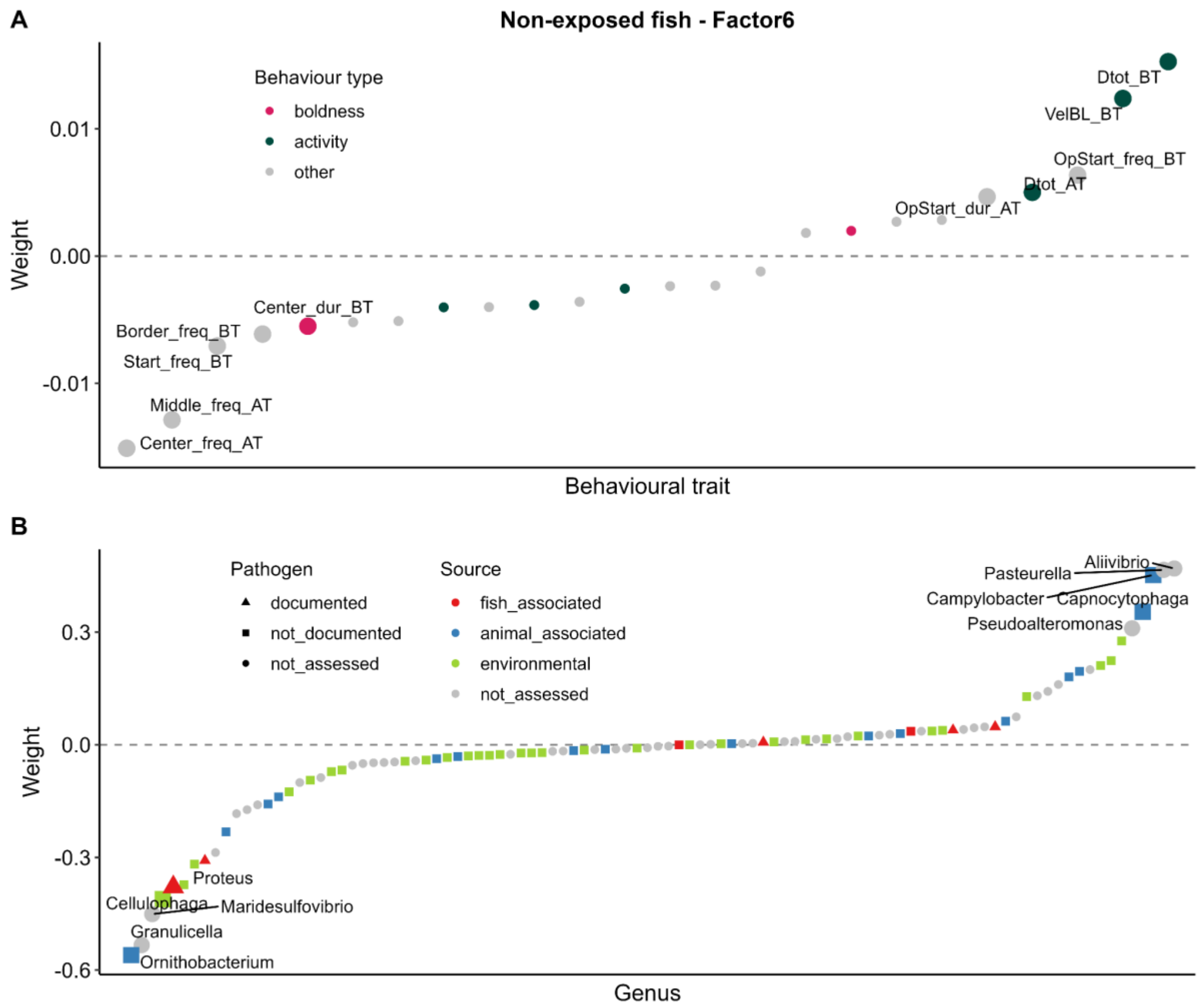

Supplementary Figure 9. Associations identified by MOFA in **non-exposed control fish** among behavioral traits and microbial abundances, for **factor 6**, which explained 0.1% of the variance in the behavioral dataset and 6% of variance in the microbiome dataset. **A)** Contribution (weight) of behavioral traits to the factor. Traits are ranked according to their weight. The higher the absolute weight, the more strongly associated a trait is with the factor. A positive weight indicates the trait has higher levels in samples with positive factor values, while a negative weight indicates the opposite. Behavioral traits are coloured by their broader behavioral category. The top five traits contributing to the factor in each direction are labeled. **B)** Contribution (weight) of microbial genera to the factor, displayed in the same way as for A. Microbial genera are coloured by their putative source and shaped on their potential as a fish pathogen, based on a literature search.

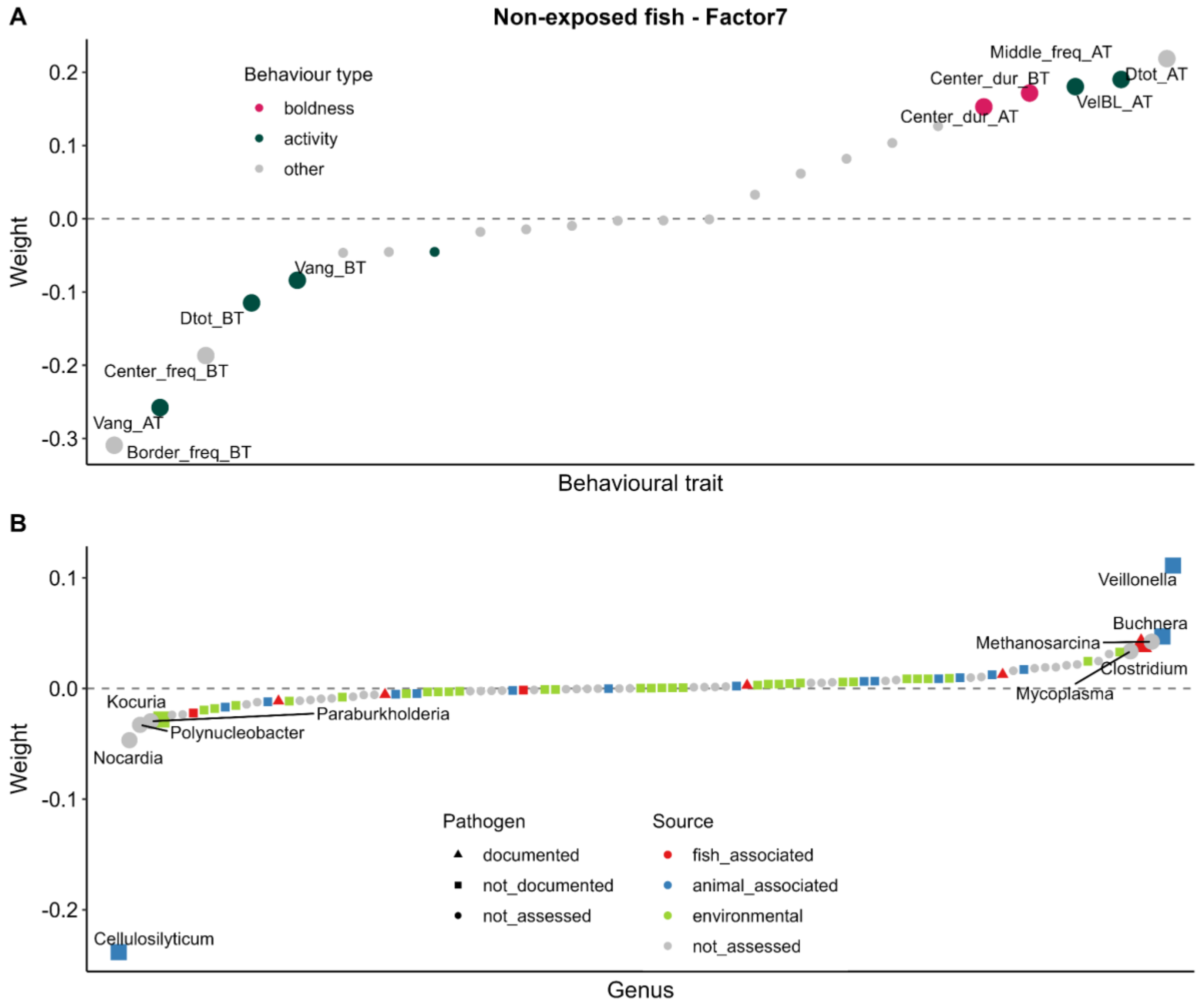

Supplementary Figure 10. Associations identified by MOFA in **non-exposed control fish** among behavioral traits and microbial abundances, for **factor 7**, which explained 3% of the variance in the behavioral dataset and 0.7% of variance in the microbiome dataset. **A)** Contribution (weight) of behavioral traits to the factor. Traits are ranked according to their weight. The higher the absolute weight, the more strongly associated a trait is with the factor. A positive weight indicates the trait has higher levels in samples with positive factor values, while a negative weight indicates the opposite. Behavioral traits are coloured by their broader behavioral category. The top five traits contributing to the factor in each direction are labeled. **B)** Contribution (weight) of microbial genera to the factor, displayed in the same way as for A. Microbial genera are coloured by their putative source and shaped on their potential as a fish pathogen, based on a literature search.

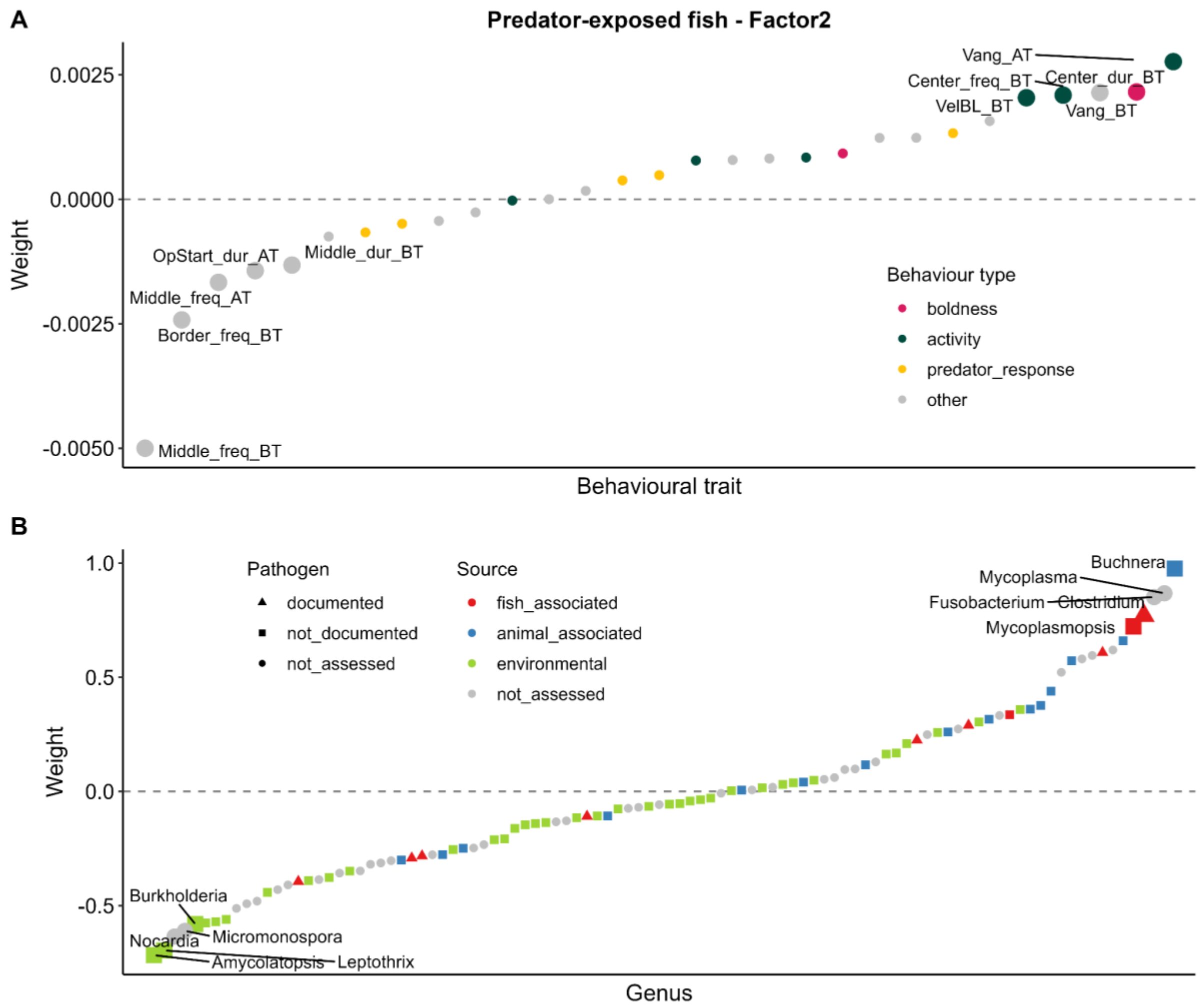

Supplementary Figure 11. Associations identified by MOFA in **predator-exposed fish** among behavioral traits and microbial abundances, for **factor 2**, which explained 0.04% of the variance in the behavioral dataset and 18% of variance in the microbiome dataset. **A)** Contribution (weight) of behavioral traits to the factor. Traits are ranked according to their weight. The higher the absolute weight, the more strongly associated a trait is with the factor. A positive weight indicates the trait has higher levels in samples with positive factor values, while a negative weight indicates the opposite. Behavioral traits are coloured by their broader behavioral category. The top five traits contributing to the factor in each direction are labeled. **B)** Contribution (weight) of microbial genera to the factor, displayed in the same way as for A. Microbial genera are coloured by their putative source and shaped on their potential as a fish pathogen, based on a literature search.

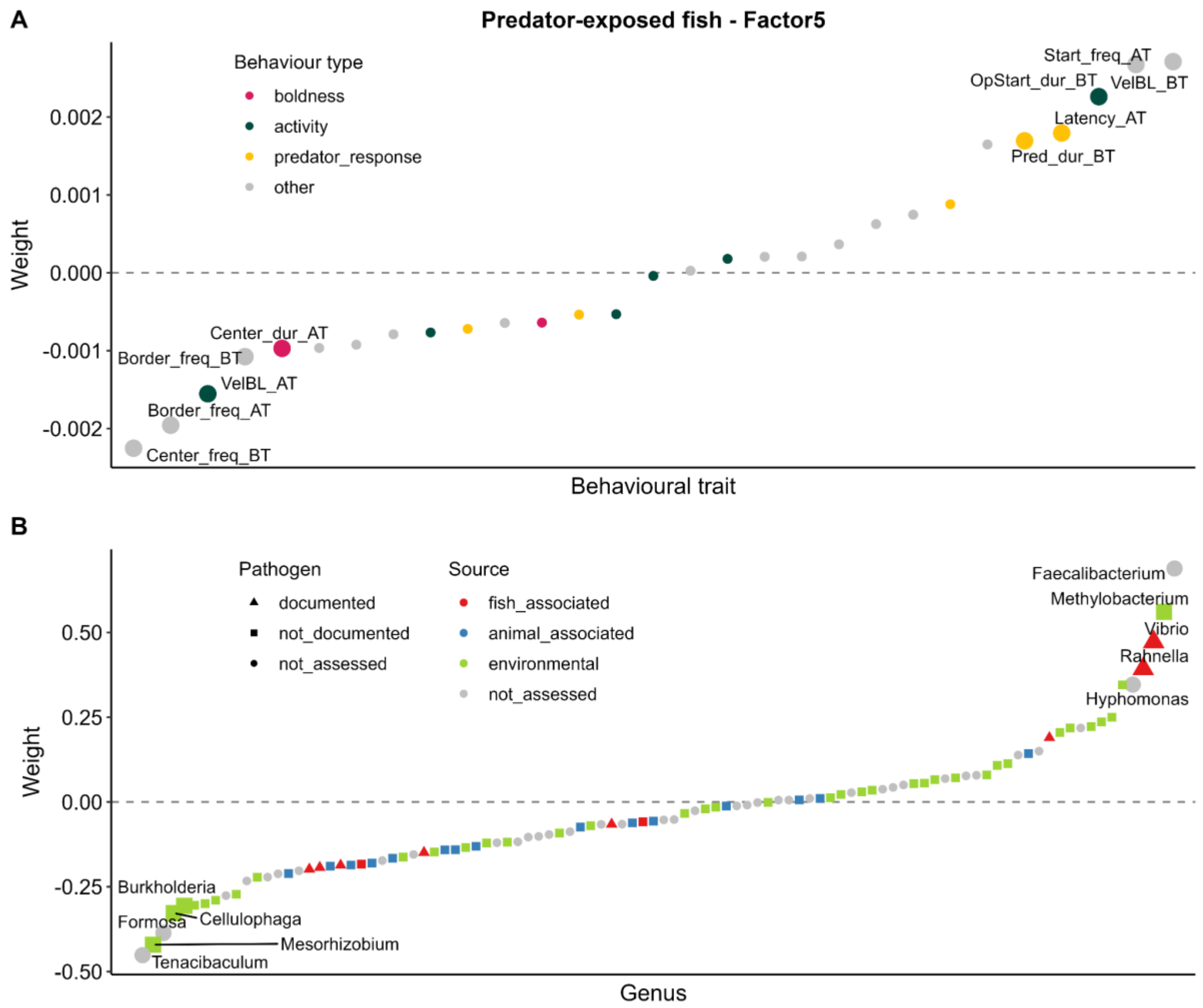

Supplementary Figure 12. Associations identified by MOFA in **predator-exposed fish** among behavioral traits and microbial abundances, for **factor 5**, which explained 0.03% of the variance in the behavioral dataset and 7% of variance in the microbiome dataset. **A)** Contribution (weight) of behavioral traits to the factor. Traits are ranked according to their weight. The higher the absolute weight, the more strongly associated a trait is with the factor. A positive weight indicates the trait has higher levels in samples with positive factor values, while a negative weight indicates the opposite. Behavioral traits are coloured by their broader behavioral category. The top five traits contributing to the factor in each direction are labeled. **B)** Contribution (weight) of microbial genera to the factor, displayed in the same way as for A. Microbial genera are coloured by their putative source and shaped on their potential as a fish pathogen, based on a literature search.

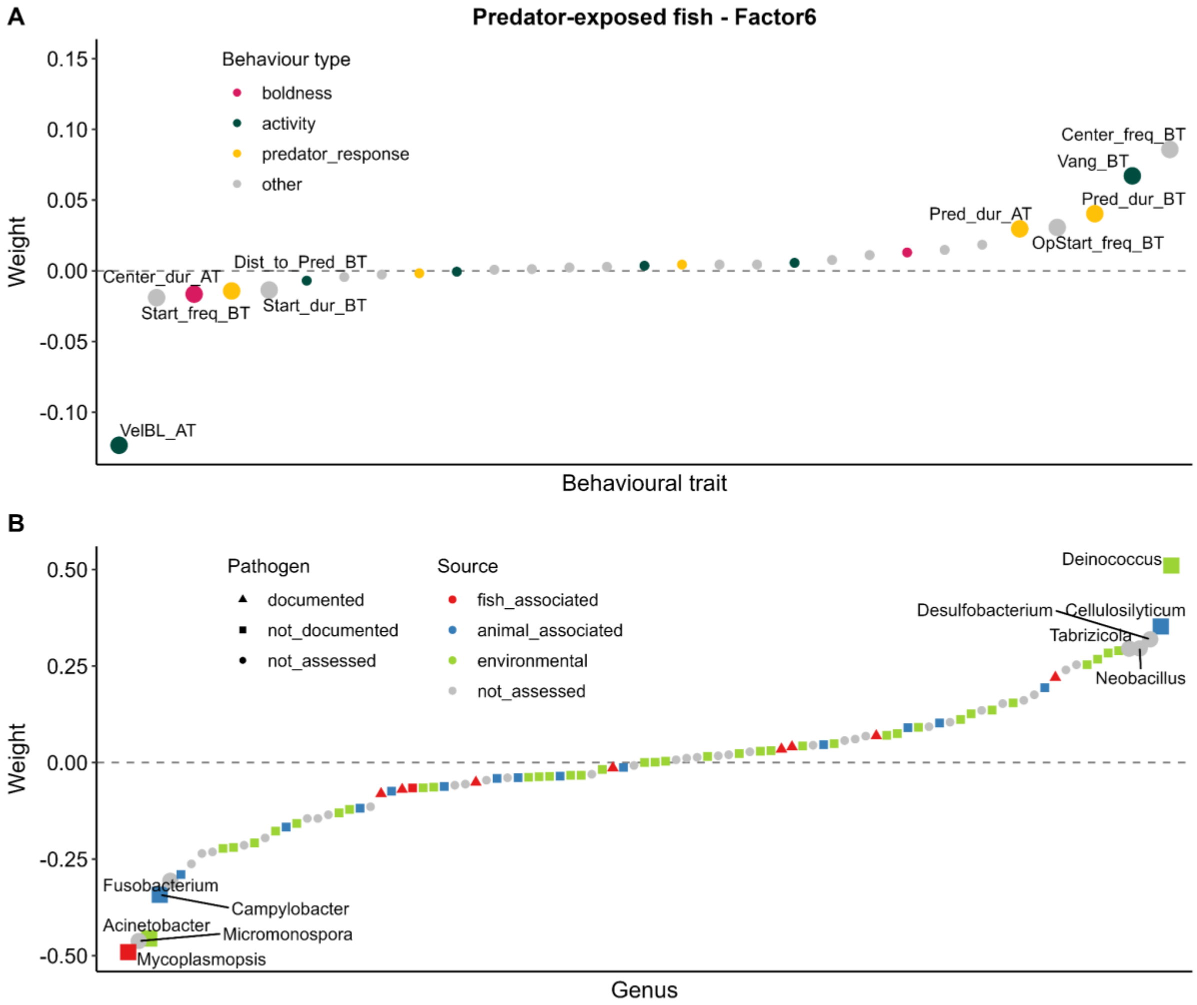

Supplementary Figure 13. Associations identified by MOFA in **predator-exposed fish** among behavioral traits and microbial abundances, for **factor 6**, which explained 0.6% of the variance in the behavioral dataset and 6% of variance in the microbiome dataset. **A)** Contribution (weight) of behavioral traits to the factor. Traits are ranked according to their weight. The higher the absolute weight, the more strongly associated a trait is with the factor. A positive weight indicates the trait has higher levels in samples with positive factor values, while a negative weight indicates the opposite. Behavioral traits are coloured by their broader behavioral category. The top five traits contributing to the factor in each direction are labeled. **B)** Contribution (weight) of microbial genera to the factor, displayed in the same way as for A. Microbial genera are coloured by their putative source and shaped on their potential as a fish pathogen, based on a literature search.

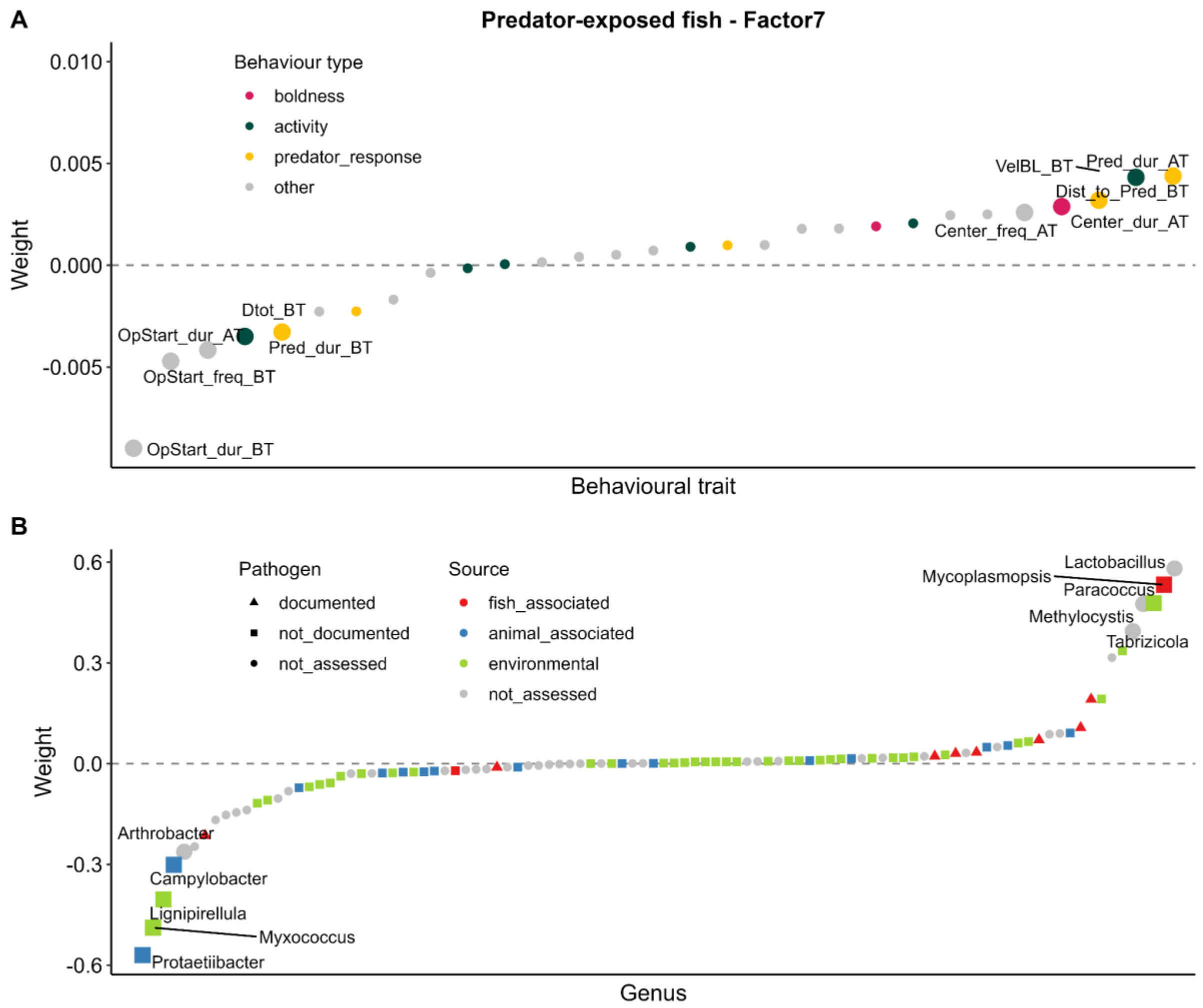

Supplementary Figure 14. Associations identified by MOFA in **predator-exposed fish** among behavioral traits and microbial abundances, for **factor 7**, which explained 0.08% of the variance in the behavioral dataset and 5% of variance in the microbiome dataset. **A)** Contribution (weight) of behavioral traits to the factor. Traits are ranked according to their weight. The higher the absolute weight, the more strongly associated a trait is with the factor. A positive weight indicates the trait has higher levels in samples with positive factor values, while a negative weight indicates the opposite. Behavioral traits are coloured by their broader behavioral category. The top five traits contributing to the factor in each direction are labeled. **B)** Contribution (weight) of microbial genera to the factor, displayed in the same way as for A. Microbial genera are coloured by their putative source and shaped on their potential as a fish pathogen, based on a literature search.

Supplementary Tables

Supplementary Table 1. Full results from the contrast post hoc tests for Figure 2.

Total Distance Traveled

contrast	estimate	SE	df	t.ratio	p.value
Galta_Control_vs_Galta_Predator	2.67	2.33	79	1.143	0.2565
Galta_Control_vs_Pristi_Control	3.65	2.2	79	1.655	0.1018
Galta_Predator_vs_Pristi_Predator	5.04	2.33	79	2.162	0.0337
Pristi_Control_vs_Pristi_Predator	4.06	2.2	79	1.842	0.0693

Mean Velocity

contrast	estimate	SE	df	t.ratio	p.value
Galta_Control_vs_Galta_Predator	0.0357	0.0568	79	0.629	0.5313
Galta_Control_vs_Pristi_Control	0.0839	0.0536	79	1.565	0.1217
Galta_Predator_vs_Pristi_Predator	0.1394	0.0568	79	2.457	0.0162
Pristi_Control_vs_Pristi_Predator	0.0912	0.0536	79	1.7	0.0931

Angular Velocity

contrast	estimate	SE	df	t.ratio	p.value
Galta_Control_vs_Galta_Predator	-0.1144	0.0577	79	-1.982	0.0509
Galta_Control_vs_Pristi_Control	-0.0768	0.0545	79	-1.408	0.1632
Galta_Predator_vs_Pristi_Predator	-0.1768	0.0577	79	-3.065	0.003
Pristi_Control_vs_Pristi_Predator	-0.2145	0.0545	79	-3.933	0.0002

Center Duration

contrast	estimate	SE	df	t.ratio	p.value
Galta_Control_vs_Galta_Predator	-39.8	21.3	79	-1.871	0.065
Galta_Control_vs_Pristi_Control	-61.6	20.1	79	-3.066	0.003
Galta_Predator_vs_Pristi_Predator	-10.2	21.3	79	-0.481	0.6319
Pristi_Control_vs_Pristi_Predator	11.6	20.1	79	0.577	0.5654

Supplementary Table 2. Full results from the contrast post hoc tests for Figure 3.

Angular Velocity

contrast	estimate	SE	df	t.ratio	p.value
Galta_BT_Np_vs_Galta_AT_Np	-0.08237	0.0625	69.9	-1.318	0.1918
Pristi_BT_Np_vs_Pristi_AT_Np	-0.25824	0.0625	69.9	-4.132	0.0001
Galta_BT_Np_vs_Galta_BT_P	0.14152	0.0468	40	3.027	0.0043
Galta_AT_Np_vs_Galta_AT_P	-0.03436	0.0515	40	-0.667	0.5087
Pristi_BT_Np_vs_Pristi_BT_P	0.05009	0.0729	40	0.687	0.4958
Pristi_AT_Np_vs_Pristi_AT_P	0.00249	0.0787	40	0.032	0.9749
Galta_BT_Np_vs_Pristi_BT_Np	-0.08237	0.0625	69.9	-1.318	0.1918
Galta_BT_P_vs_Pristi_BT_P	-0.25824	0.0625	69.9	-4.132	0.0001
Galta_AT_Np_vs_Pristi_AT_Np	-0.06458	0.0963	69.9	-0.67	0.5049
Galta_AT_P_vs_Pristi_AT_P	-0.11217	0.0963	69.9	-1.164	0.2483

Center Duration

contrast	estimate	SE	df	t.ratio	p.value
Galta_BT_Np_vs_Galta_AT_Np	18.2	24.1	67.5	0.754	0.4532
Pristi_BT_Np_vs_Pristi_AT_Np	2.7	24.1	67.5	0.112	0.911
Galta_BT_Np_vs_Galta_BT_P	-72.2	17.3	40	-4.182	0.0002
Galta_AT_Np_vs_Galta_AT_P	-87.7	19	40	-4.608	<.0001
Pristi_BT_Np_vs_Pristi_BT_P	-40.8	26.9	40	-1.516	0.1373
Pristi_AT_Np_vs_Pristi_AT_P	-39.5	29.1	40	-1.359	0.1817
Galta_BT_Np_vs_Pristi_BT_Np	18.2	24.1	67.5	0.754	0.4532
Galta_BT_P_vs_Pristi_BT_P	2.7	24.1	67.5	0.112	0.911
Galta_AT_Np_vs_Pristi_AT_Np	32.5	37.1	67.5	0.876	0.3843
Galta_AT_P_vs_Pristi_AT_P	33.8	37.1	67.5	0.911	0.3657

Supplementary Table 3. PERMANOVAs of variation in stickleback gut microbiomes.

Test	Variable	Degrees of freedom	Sum of squares	R2	F	P value
Non-parasitized Galtaból vs Þristikla (**after** batch correction)	Sequencing depth	1	673	5.0%	2.30	**0.001**
	Population	1	565	4.2%	1.93	**0.003**
	Treatment	1	280	2.1%	0.96	0.550
	length (cm)	1	188	1.4%	0.64	0.998
	Extraction date	5	300	2.2%	0.21	1.000
	Residual	39	11406	85.0%	NA	NA
	Total	48	13413	100.0%	NA	NA
Galtaból non-parasitized vs parasitized (**after** batch correction)	Sequencing depth	1	683	6.4%	2.16	**0.002**
	Parasite	1	767	7.2%	2.42	**0.001**
	Treatment	1	325	3.0%	1.03	0.350
	length (cm)	1	192	1.8%	0.61	0.990
	Extraction date	5	204	1.9%	0.13	1.000
	Residual	27	8539	79.7%	NA	NA
	Total	36	10710	100.0%	NA	NA
Non-parasitized Galtaból vs Þristikla (**before** batch correction)	Sequencing depth	1	776	5.2%	2.65	**0.001**
	Population	1	485	3.2%	1.66	**0.004**
	Treatment	1	275	1.8%	0.94	0.618
	length (cm)	1	297	2.0%	1.02	0.372
	Extraction date	5	1777	11.8%	1.22	**0.005**
	Residual	39	11406	76.0%	NA	NA
	Total	48	15015	100.0%	NA	NA
Galtaból non-parasitized vs parasitized	Sequencing depth	1	914	7.6%	2.89	**0.001**
	Parasite	1	345	2.9%	1.09	0.226
	Treatment	1	299	2.5%	0.94	0.566

(**before** batch correction)	length (cm)	1	260	2.2%	0.82	0.889
	Extraction date	5	1734	14.3%	1.10	0.150
	Residual	27	8539	70.6%	NA	NA
	Total	36	12091	100.0%	NA	NA

Supplementary Table 4. Significant associations (adjusted $p < 0.05$) between population (non-parasitized) and parasite status (within Galtaból), based on genus-level relative abundances. Treatment(non-exposed vs predator-exposed fish) was also included in the models; however, no significant associations were found.

Test	Microbe (genus)	Direction	Co-efficient	SE	N (all)	N (not 0)	P value	Adjusted p value
Non-parasitized Galtaból vs Þristikla	Pseudo-alteromonas	Increased in Galta	-0.002	0.0004	49	36	4.66E-07	0.0003
Galtaból non-parasitized vs parasitized	Kitasatospora	Increased in NP	-0.023	0.0054	37	37	1.45E-04	0.0487
	Shewanella	Increased in NP	-0.006	0.0014	37	37	1.38E-04	0.0487

Supplementary Table 5. Literature search results for annotating microbial genera of interest in MOFA result figures. Putative sources were assigned using the following: if the genus has been documented as a fish pathogen, source = fish associated (even if the genus is also found in the environment); if the genus has been documented as present in a fish, source = fish associated (even if the genus is also found in the environment); if the genus has been documented as present in an animal (insect, bird, mammal, etc), source = animal associated (even if the genus is also found in the environment; exception: opportunistic human pathogens where disease is only documented in immunocompromised/hospital environments, and the known reservoir is the environment); if no documentation exists for the genus's ability to survive in an animal, source = environmental.

Genus	Species in our dataset	Comments	Literature	Putative source	Documented as a fish pathogen
Acineto-bacter	top: Acinetobacter baumannii; Acinetobacter sp. WCHA55	genus of soil bacteria, but Ab found almost always in hospitals/sewage etc (natural habitat not known), opportunistic pathogen of humans and mammals (vet clinics), has been found in aquaculture facilities	review: 10.1016/j.ijid.2013.03.021	environmental	No
Aeromonas	top: Aeromonas sp. CU5; Aeromonas veronii; Aeromonas hydrophila; Aeromonas salmonicida	water & animal-associated; can be pathogenic in humans; can be pathogenic in fish	10.3389/fmicb.2019.02742; 10.1111/1751-7915.12091	fish associated	Yes
Agro-bacterium	Agrobacterium tumefaciens	soil bacteria, pathogen of various plants; biotech vector	10.1128/MMBR.67.1.16-37.2003	environmental	No
Amyco-latopsis	top: Amycolatopsis keratiniphila; Amycolatopsis sp. YIM 10	marsh soil; Chinese mine soil; producer of secondary metabolites	10.1099/ijs.0.02515-0; review: 10.3390/antibiotics10101254	environmental	No
Arseno-phonus	Arsenophonus nasoniae	associated with parasitic-wasps ("son-killer microbe")	10.1016/j.jip.2023.107947	animal associated	No
Blatta-bacterium	Blattabacterium cuenoti	cockroach endosymbiont, important for nitrogen recycling in insects	10.1016/S1055-7903(02)00330-5	animal associated	No

Bordetella	top: Bordetella trematum	rare opportunistic pathogen in healthcare; various spp of genus found in environment and are likely the ancestor to the disease-associated spp in animals	10.1099/00207713-46-4-849; 10.3389/fmicb.2017.00028	environmental	No
Brady-rhizobium	top: Bradyrhizobium sp.; Bradyrhizobium diazoefficiens	soil and plant-associated rhizosphere microbes (mostly)	10.1016/j.syapm.2019.03.006	environmental	No
Brevibacillus	Brevibacillus formosus; Brevibacillus laterosporus	environmental, especially soil; B. laterosporus can also inhabit insects & may be pathogenic to invertebrates	10.1128/genomeA.00753-15; 10.3390/insects4030476	animal associated	No
Buchnera	Buchnera aphidicola	endosymbiont of aphids (insect)	10.1146/annurev.ento.43.1.17	animal associated	No
Burkholderia	top: Burkholderia multivorans; Burkholderia vietnamiensis	soil microbes, can be found in marine environments, can be plant-associated, can be plant pathogens, can be opportunistic pathogens in humans	10.1111/j.1574-6976.2008.00113.x	environmental	No
Caldicellulo-siruptor	Caldicellulosiruptor changbaiensis	thermophilic aquatic bacteria from a hot spring	10.1099/ijs.0.065441-0	environmental	No
Campylo-bacter	top: Campylobacter jejuni; Campylobacter sp. RM6914	guts of birds and cattle; can be a food-borne pathogen in humans	10.1128/cmr.00006-15	animal associated	No
Capno-cytophaga	top: Capnocytophaga endodontalis	spp of genus (including Ce) found in oropharyngeal tract of mammals	review: 10.1016/j.ijantimicag.2006.10.005	animal associated	No
Cedecea	Cedecea neteri	found in insects and aquatic environments; rare human pathogen	10.1017/S1742758400012868; 10.1186/s40793-017-0255-1	animal associated	No
Cellulophaga	top: Cellulophaga lytica	marine water & macroalgae, coastal habitats	10.1099/00207713-49-3-1231	environmental	No
Cellulosi-lyticum	Cellulosilyticum sp. WCF-2	cow faeces; various spp isolated from rumens, river sediment; degrade cellulose	10.1099/ijs.0.014712-0	animal associated	No
Chelato-coccus	Chelatococcus sp. CO-6	environmental	10.1016/j.jbiotec.2015.12.021	environmental	No
Clostridioides	Clostridioides difficile	soil & water microbe; pathobiont of human & mammalian gut	rivers: 10.1016/j.anaerobe.2010.06.001; review:	animal associated	No

			10.1111/j.1863-2378.2010.01352.x		
Clostridium	top: Clostridium botulinum	soil, aquatic sediments; toxic producing; fish can be asymptomatic carriers, but outbreaks can occur with high mortality in fish populations	10.3389/fmicb.2014.00287	fish associated	Yes
Coryne-bacterium	top: Corynebacterium sp. NML98-0116; Corynebacterium urealyticum	opportunistic nosocomial pathogen, often isolated from urine; colonises human skin and urinary tract, probably transmitted from skin to infectious sites, medical devices, etc; various spp of genus can be environmental	review: 10.2147/IDR.S74795	animal associated	No
Deefgea	Deefgea sp. D17	gut of a freshwater fish	10.1007/s12275-022-2250-5	fish associated	No
Deinococcus	top: Deinococcus sp. D7000; Deinococcus proteolyticus	various environmental sources, but also mammal faeces (unclear if growth after defecation however)	10.1016/j.margen.2020.100832; 10.4056/sigs.2756060	environmental	No
Ensifer	Ensifer sojae	plant-associated rhizosphere	10.1099/ijs.0.025049-0	environmental	No
Escherichia	top: Escherichia coli	guts of mammals; some can cause disease; can survive in water and can be found in and on fish, likely from contaminated waters (i.e. not a natural resident of fish guts)	fish: 10.1016/j.watres.2004.02.016	animal associated	No
Finegoldia	Finegoldia magna	mammalian skin & mucosa associated; Fm is a human opportunistic pathogen and can be found in clinical environments	10.1038/s41598-017-18661-8	animal associated	No
Flavo-bacterium	top: Flavobacterium nitrogenifigens; Flavobacterium magnum	soil and water environments, including rhizosphere; can cause disease in fish, depending on species	10.1007/s40588-018-0086-x	fish associated	Yes
Geitlerinema	Geitlerinema sp. PCC 7407	cyanobacteria, soil & water associated in biofilms	10.1073/pnas.1217107110	environmental	No
Gemma-timonas	Gemmatimonas sp. TET16	high arctic stream in Greenland; photosynthetic	10.3389/fmicb.2020.606612	environmental	No

Gloeobacter	Gloeobacter violaceus	rock-dwelling cyanobacteria	10.1093/dnares/10.4.137 ; 10.1371/journal.pone.0066323	environmental	No
Glucono-bacter	Gluconobacter oxydans	highly studied due to biotech applications, grows in sugary environments like flowers, fruit, beer, garden soil; can cause rot in some fruits	review: https://pubmed.ncbi.nlm.nih.gov/11361077/	environmental	No
Gottschalkia	Gottschalkia acidurici	environmental, both terrestrial and aquatic	10.1099/ijsem.0.002008	environmental	No
Haloactino-bacterium	Haloactinobacterium sp. HY164	bat faeces; taxonomy seems a bit of a mess, also possible soil or sediment bacteria	10.1099/ijsem.0.004953	environmental	No
Klebsiella	top: Klebsiella oxytoca; Klebsiella pneumoniae	genus of environmental (soil & water) bacteria, some of which can cause infections in humans, especially immuno-compromised, one main infection route is the gut; Ko has mutualistic relationship with housefly eggs, also researched for biofuel production; zebrafish are a model for Klebsiella infections, so can colonize the gut of fish; Kp can also cause disease in fish	flies: 10.1007/s00114-009-0574-1; soil/water: 10.1007/s00203-018-1532-0; disease in fish: 10.1016/j.aquaculture.2021.737376	fish associated	Yes
Kocuria	top: Kocuria turfanensis; Kocuria palustris	air in China; rhizosphere of wetland plant	10.1099/ijs.0.65323-0; 10.1099/00207713-49-1-167	environmental	No
Labrenzia	top: Labrenzia alexandrii	marine protist	10.1099/ijs.0.64821-0	environmental	No
Lactococcus	Lactococcus garvieae; Lactococcus lactis	Lg is a fish pathogen, also found in the environment; Ll is associated with dairy products & plants, frequently used in cheeses	10.1016/j.rvsc.2016.09.010	fish associated	Yes
Leptothrix	Leptothrix cholodnii	iron-rich freshwater streams, wetlands, sediments	old book chapter: 10.1128/mr.42.2.329-356.1978	environmental	No
Leuconostoc	Leuconostoc gelidum	stored meat & dairy products; plant material	10.1099/00207713-39-3-217	environmental	No
Lignipirellula	Lignipirellula cremea	decomposing wood in water	10.1007/s10482-020-01407-4	environmental	No

Massilia	top: Massilia sp. WG5; Massilia armeniaca; Massilia albidiflava; Massilia violaceinigra	soil, especially Chinese environments; freshwater from glaciers	10.1099/ijs.0.64083-0; 10.1099/ijsem.0.002836; 10.3390/microorganisms 10040704	environmental	No
Meso-rhizobium	top: Mesorhizobium opportunistum	plant-associated rhizosphere	10.1099/ijs.0.005728-0	environmental	No
Methylo-bacterium	top: Methylobacterium aquaticum	freshwater, soil, aquatic sediments; some from aquatic plants etc too	10.1099/ijs.0.63319-0	environmental	No
Myco-bacterium	top: Mycobacterium mantenii	water, rare human infections	10.1099/ijs.0.010405-0	environmental	No
Myco-plasmopsis	top: Mycoplasma pulmonis; Mycoplasma maculosum	note some taxonomy overlap with Mycoplasma & Mycoplasmopsis; whole family is a member of fish gut microbiota; Mycoplasma mobile can cause disease in fish when present in other places e.g. skin; can also be found in other environments	10.1111/jfd.13283; 10.1038/ismej.2015.189	fish associated	No
Myxococcus	top: Myxococcus stipitatus	soil bacteria, saprophytic & predatory, "social" bacteria, form fruiting bodies	10.1046/j.1462-2920.1999.00016.x; 10.1111/j.1758-2229.2012.00373.x	environmental	No
Neo-micrococcus	Neomicrococcus aestuarii	tidal flat sediment (genus reassigned)	10.1099/ijs.0.026245-0; 10.1099/ijsem.0.000490	environmental	No
Nostoc	top: Nostoc sp. 'Lobaria pulmonaria (5183) cyanobiont'; Nostoc sp. PCC 7524	cyanobacteria, possibly lichen-associated, possibly free-living	10.1073/pnas.1217107110; 10.1186/s12864-018-4743-5	environmental	No
Olleya	Olleya aquimaris	marine environments	10.1099/ijs.0.014563-0	environmental	No
Ornitho-bacterium	Ornithobacterium rhinotracheale	respiratory disease in poultry, also isolated in wild birds (both with & without signs of disease), can be passed through close-contact and water	10.3390/vetsci7010003; 10.1186/s12866-019-1395-9	animal associated	No
Pandoraea	top: Pandoraea sp. XY-2; Pandoraea apista	degrading environments, e.g. rotten wood, wastewater, sediment sludge; Pa found in immunocompromised patients	10.3389/fmicb.2019.00033	environmental	No

Pantoea	Pantoea alhagi; Pantoea dispersa	soil or plant-associated rhizosphere (rare opportunistic pathogens)	10.1016/j.syapm.2008.09.004	environmental	No
Paracoccus	top: Paracoccus sanguinis	majority of spp from soil, mud, groundwater, etc, some Ps and Py found in human clinical samples;	clinical: 10.1099/ijs.0.000193; book chapter (can't access): 10.1007/0-387-30745-1_12	environmental	No
Polaribacter	top: Polaribacter haliotis; Polaribacter sp. SA4-12	gut of marine gastropod; sea ice; members of genus from seawater/ice, polar soil, algae, polar sediment	10.1099/ijsem.0.001557	environmental	No
Propioni-bacterium	top: Propionibacterium australiense	mammalian skin genus; species in lesions in cattle	10.1128/MRA.01445-18	animal associated	No
Protaetii-bacter	Protaetiibacter intestinalis	gut of an insect; spp of genus also found in soil	10.1099/ijsem.0.003444	animal associated	No
Proteus	top: Proteus cibarius	environmental and mammalian gut, also found in marine fishes; may be pathogenic OR beneficial in fish, depending on bacteria species and fish species; Pc found in fermented seafood; some species are human opportunistic pathogens	10.1007/s00248-015-0720-6	fish associated	Yes
Providencia	top: Providencia rettgeri	water & soil, marine sponge, oral cavity of sharks; pathogen of some insects; emerging nosocomial pathogen	sponge: 10.1016/j.aquaculture.2021.737156; insect pathogen: 10.1016/j.micinf.2011.02.005	animal associated	No
Rahnella	Rahnella aquatilis	water, soil, plant rhizosphere; can cause disease in some fish	10.1111/j.1574-6968.1997.tb12585.x; 10.3354/dao03099	fish associated	Yes
Rhodoluna	Candidatus Rhodoluna planktonica	freshwater	10.1099/ijs.0.001743-0	environmental	No
Saccharo-polyspora	Saccharopolyspora erythraea	soil, antibiotic-producer	10.1038/nbt1297	environmental	No

Salmonella	Salmonella enterica	human foodborne pathogen, primary reservoirs are in poultry, also resides in human gut; can survive well in the environment; not a native in aquatic environments, but is found in sea & river waters, and in seafood & various fish species (usually due to pollution)	10.1007/s13213-015-1102-5	environmental	No
Sanguibacter	Sanguibacter keddieii	cultured from blood samples of healthy cows	10.4056/sigs.16197	animal associated	No
Sphingo-monas	top: Sphingomonas lutea; Sphingomonas alpina	freshwater; alpine soil	10.1099/ijsem.0.001546; 10.1099/ijs.0.035964-0	environmental	No
Spiroplasma	top: Spiroplasma tabanidicola; Spiroplasma chinense	insect-associated; can cause disease in plants and opportunistic (rare) disease in humans	10.5604/12321966.1185758	animal associated	No
Stacke-brandtia	Stackebrandtia nassauensis	soil	10.1099/ijs.0.63496-0	environmental	No
Sulfitobacter	top: Sulfitobacter pseudonitzschiae	isolated from diatom (i.e. algae-associated)	10.1099/ijs.0.064972-0	environmental	No
Thioflavi-coccus	Thioflavicoccus mobilis	marine microbial mat in a salt marsh	10.1099/00207713-51-1-105	environmental	No
Thiomonas	Thiomonas intermedia	freshwater water-associated soil (mud, sediment)	10.1128/AEM.01424-20	environmental	No
Tricho-desmium	Trichodesmium erythraeum	marine cyanobacteria, more frequent in the tropics	10.1111/j.1574-6976.2012.00352.x	environmental	No
Veillonella	Veillonella dispar; Veillonella parvula	gut & oral cavity of mammals, can sometimes cause disease in mammals	10.1146/annurev.mi.39.100185.001135	animal associated	No
Vibrio	top: Vibrio taketomensis	sp from seawater; genus of marine bacteria; can causes disease in fish (often skin lesions, then septicaemia)	seawater: 10.1016/j.syapm.2019.126048; fish disease: 10.1016/j.aqrep.2022.101459	fish associated	Yes
Wenying-zhuangia	Wenyingzhuangia fucanilytica	coastal seawater; can break down sulfated fucans	10.1099/ijsem.0.001184	environmental	No

List of Supplementary Files

Supplementary Data 1. R code for statistical analysis of behavior dataset.

Supplementary Data 2. Sample metadata, with subset of the behavioral traits extracted from the videos including data from the non-exposed treatment and the fish's activity during the acclimation period in the predator exposure (i.e., before the robotic predator was triggered).

Supplementary Data 3. Sample metadata, with the predator-exposed behavioral traits extracted from the videos, including the Period Before and after triggering the robotic predator

Supplementary Data 4. Sample metadata, including behavioral traits extracted from the videos, and sample ENA accessions.

Supplementary Data 5. HTML R markdown file containing statistical analysis of microbiome dataset, statistical analysis for associations between the microbiome and behavior datasets and generation of all figures.
