# Supplementary material for: Interplay between antipredator behavior, parasitism, and gut microbiome in wild stickleback populations
Source: NPJ Biofilms Microbiomes. 2025 Jul 19;11:138. doi: 10.1038/s41522-025-00758-y (PMC12276351; doi:10.1038/s41522-025-00758-y)
Supplement: Supplementary file 2 — Supplementary data [file 41522_2025_758_MOESM2_ESM.zip › Supplementary Data 1_behavior_statistical_analysis.html]

Stk behavior


# Stk behavior

#### 2025-02-12

## R Markdown

This is an R Markdown document. Markdown is a simple formatting
syntax for authoring HTML, PDF, and MS Word documents. For more details
on using R Markdown see http://rmarkdown.rstudio.com.

When you click the **Knit** button a document will be
generated that includes both content as well as the output of any
embedded R code chunks within the document. You can embed an R code
chunk like this:

Note that the `echo = FALSE` parameter was added to the
code chunk to prevent printing of the R code that generated the
plot.

rm(list=ls()) ls()

Packages

```
rm(list=ls())
ls()
```

```
## character(0)
```

```
#load the following libraries in R 
library(car)
```

```
## Loading required package: carData
```

```
library(lme4)
```

```
## Loading required package: Matrix
```

```
library(emmeans)
```

```
## Welcome to emmeans.
## Caution: You lose important information if you filter this package's results.
## See '? untidy'
```

```
library(ggplot2)
library(Rmisc)
```

```
## Loading required package: lattice
```

```
## Loading required package: plyr
```

```
library(lattice)
library(MASS)
#####to transform the data####
library(ggpubr)
```

```
## 
## Attaching package: 'ggpubr'
```

```
## The following object is masked from 'package:plyr':
## 
##     mutate
```

```
library(Rmisc)
library(moments)
```

tranwforming the data for ## Treatment Before VS Control Before
########### Activity and Tank use

```
####tranwforming the data for ## Treatment Before VS Control Before ########### Activity and Tank use 
rm(list=ls())
ls()
```

```
## character(0)
```

```
convstreat <- read.csv("~/Documents/Iceland project/Dta/No6TreatmentBeforevsControlBefore.csv") 

names(convstreat)
```

```
##  [1] "Video_nbb"        "Type"             "Population"       "TypePopulation"  
##  [5] "Parasite"         "Period"           "Sex"              "length_cm"       
##  [9] "Weight"           "Vang"             "Middle_Duration"  "Start_Duration"  
## [13] "Border_duration"  "Dtot"             "Dist_to_Pred"     "Pred_duration"   
## [17] "Vel"              "VelBL"            "Center_Duration"  "OpStart_Duration"
## [21] "Middle_Freq"      "Start_Freq"       "Center_Freq"      "Border_Freq"     
## [25] "OpStart_freq"     "first_reaction"   "X5sec_after"
```

```
attach(convstreat)

######### swiming activity###

shapiro.test(convstreat $Dtot)#no normal
```

```
## 
##  Shapiro-Wilk normality test
## 
## data:  convstreat$Dtot
## W = 0.96135, p-value = 0.0136
```

```
shapiro.test(convstreat $VelBL)#no normal
```

```
## 
##  Shapiro-Wilk normality test
## 
## data:  convstreat$VelBL
## W = 0.95995, p-value = 0.0111
```

```
shapiro.test(convstreat $Vang)#no normal
```

```
## 
##  Shapiro-Wilk normality test
## 
## data:  convstreat$Vang
## W = 0.84479, p-value = 7.162e-08
```

```
shapiro.test(convstreat$Center_Duration) #normal
```

```
## 
##  Shapiro-Wilk normality test
## 
## data:  convstreat$Center_Duration
## W = 0.97473, p-value = 0.101
```

```
shapiro.test(convstreat$Border_duration) #normal
```

```
## 
##  Shapiro-Wilk normality test
## 
## data:  convstreat$Border_duration
## W = 0.9744, p-value = 0.09613
```

```
skewness(convstreat$Dtot, na.rm = TRUE)
```

```
## [1] 0.5591598
```

```
skewness(convstreat$VelBL, na.rm = TRUE)
```

```
## [1] 0.5453326
```

```
NorDtot <- sqrt(convstreat$Dtot)
NorVelBL <- sqrt(convstreat$VelBL)
NorVang <- log10(convstreat$Vang)


shapiro.test(NorDtot)
```

```
## 
##  Shapiro-Wilk normality test
## 
## data:  NorDtot
## W = 0.98563, p-value = 0.4856
```

```
shapiro.test(NorVelBL)
```

```
## 
##  Shapiro-Wilk normality test
## 
## data:  NorVelBL
## W = 0.98449, p-value = 0.4192
```

```
shapiro.test(NorVang)
```

```
## 
##  Shapiro-Wilk normality test
## 
## data:  NorVang
## W = 0.97519, p-value = 0.1083
```

```
###add the data transformed into the data set
convstreat <- cbind(convstreat, NorDtot)
convstreat <- cbind(convstreat, NorVelBL)
convstreat <- cbind(convstreat, NorVang)


write.table(convstreat, file="~/Documents/Iceland project/Before_Control-Before_TreatmentNor.csv", col.names=NA,row.names=TRUE, sep=",", quote=FALSE)

rm(list=ls())
ls()
```

```
## character(0)
```

```
convstreat <- read.csv("~/Documents/Iceland project/Before_Control-Before_TreatmentNor.csv")
```

Treatment Before VS Control Before ########### Activity and Tank
use

```
############# Treatment Before VS Control Before ########### Activity and Tank use 
#Table 1 
#########

#NorDtot

convstreat <- read.csv("~/Documents/Iceland project/Before_Control-Before_TreatmentNor.csv") 

convstreat$Population <- as.factor(convstreat$Population) 
convstreat$Type <- as.factor(convstreat$Type) 

fit13 <- lm(NorDtot ~ Population* Type,  data= convstreat)
summary(fit13)
```

```
## 
## Call:
## lm(formula = NorDtot ~ Population * Type, data = convstreat)
## 
## Residuals:
##      Min       1Q   Median       3Q      Max 
## -20.5639  -4.7317  -0.6579   4.6907  14.7058 
## 
## Coefficients:
##                               Estimate Std. Error t value Pr(>|t|)    
## (Intercept)                     27.181      1.628  16.693   <2e-16 ***
## PopulationPristi                -2.667      2.333  -1.143    0.256    
## TypePredator                    -3.649      2.205  -1.655    0.102    
## PopulationPristi:TypePredator   -1.394      3.210  -0.434    0.665    
## ---
## Signif. codes:  0 '***' 0.001 '**' 0.01 '*' 0.05 '.' 0.1 ' ' 1
## 
## Residual standard error: 7.282 on 79 degrees of freedom
## Multiple R-squared:  0.1277, Adjusted R-squared:  0.09455 
## F-statistic: 3.854 on 3 and 79 DF,  p-value: 0.01252
```

```
contrasts(convstreat$Population) <- contr.sum 
contrasts(convstreat$Type) <- contr.sum

fit13 <- lm(NorDtot ~ Population* Type,  data= convstreat)

Anova(fit13, type="III")
```

```
## Anova Table (Type III tests)
## 
## Response: NorDtot
##                 Sum Sq Df  F value    Pr(>F)    
## (Intercept)      46154  1 870.3874 < 2.2e-16 ***
## Population         233  1   4.3918  0.039314 *  
## Type               389  1   7.3339  0.008292 ** 
## Population:Type     10  1   0.1885  0.665356    
## Residuals         4189 79                       
## ---
## Signif. codes:  0 '***' 0.001 '**' 0.01 '*' 0.05 '.' 0.1 ' ' 1
```

```
emm <- emmeans(fit13, ~ Population * Type) # Specify the relevant pairwise comparisons
summary(emm)
```

```
##  Population Type     emmean   SE df lower.CL upper.CL
##  Galta      Control    27.2 1.63 79     23.9     30.4
##  Pristi     Control    24.5 1.67 79     21.2     27.8
##  Galta      Predator   23.5 1.49 79     20.6     26.5
##  Pristi     Predator   19.5 1.63 79     16.2     22.7
## 
## Confidence level used: 0.95
```

```
contrast_list <- list( "Galta_Control_vs_Galta_Predator" = c(1, -1, 0, 0), "Galta_Control_vs_Pristi_Control" = c(1, 0, -1, 0), "Galta_Predator_vs_Pristi_Predator" = c(0, 1, 0, -1), "Pristi_Control_vs_Pristi_Predator" = c(0, 0, 1, -1) ) 
# Perform the contrasts 
contrast_results <- contrast(emm, contrast_list) 
summary(contrast_results)
```

```
##  contrast                          estimate   SE df t.ratio p.value
##  Galta_Control_vs_Galta_Predator       2.67 2.33 79   1.143  0.2565
##  Galta_Control_vs_Pristi_Control       3.65 2.20 79   1.655  0.1018
##  Galta_Predator_vs_Pristi_Predator     5.04 2.33 79   2.162  0.0337
##  Pristi_Control_vs_Pristi_Predator     4.06 2.20 79   1.842  0.0693
```

```
## 
## Call:
## lm(formula = NorVelBL ~ Population * Type, data = convstreat)
## 
## Residuals:
##      Min       1Q   Median       3Q      Max 
## -0.51166 -0.10779 -0.00132  0.12234  0.39051 
## 
## Coefficients:
##                               Estimate Std. Error t value Pr(>|t|)    
## (Intercept)                    0.64736    0.03962  16.341   <2e-16 ***
## PopulationPristi              -0.03569    0.05676  -0.629    0.531    
## TypePredator                  -0.08393    0.05364  -1.565    0.122    
## PopulationPristi:TypePredator -0.05551    0.07810  -0.711    0.479    
## ---
## Signif. codes:  0 '***' 0.001 '**' 0.01 '*' 0.05 '.' 0.1 ' ' 1
## 
## Residual standard error: 0.1772 on 79 degrees of freedom
## Multiple R-squared:  0.1219, Adjusted R-squared:  0.08855 
## F-statistic: 3.656 on 3 and 79 DF,  p-value: 0.01594
```

```
## Anova Table (Type III tests)
## 
## Response: NorVelBL
##                  Sum Sq Df  F value    Pr(>F)    
## (Intercept)     27.1015  1 863.3698 < 2.2e-16 ***
## Population       0.0829  1   2.6395  0.108222    
## Type             0.2568  1   8.1798  0.005417 ** 
## Population:Type  0.0159  1   0.5051  0.479349    
## Residuals        2.4798 79                       
## ---
## Signif. codes:  0 '***' 0.001 '**' 0.01 '*' 0.05 '.' 0.1 ' ' 1
```

```
##  contrast                          estimate     SE df t.ratio p.value
##  Galta_Control_vs_Galta_Predator     0.0357 0.0568 79   0.629  0.5313
##  Galta_Control_vs_Pristi_Control     0.0839 0.0536 79   1.565  0.1217
##  Galta_Predator_vs_Pristi_Predator   0.1394 0.0568 79   2.457  0.0162
##  Pristi_Control_vs_Pristi_Predator   0.0912 0.0536 79   1.700  0.0931
```

```
## 
## Call:
## lm(formula = NorVang ~ Population * Type, data = convstreat)
## 
## Residuals:
##      Min       1Q   Median       3Q      Max 
## -0.40729 -0.12943 -0.00938  0.12127  0.55335 
## 
## Coefficients:
##                               Estimate Std. Error t value Pr(>|t|)    
## (Intercept)                    2.24800    0.04027  55.821   <2e-16 ***
## PopulationPristi               0.11437    0.05770   1.982   0.0509 .  
## TypePredator                   0.07676    0.05453   1.408   0.1632    
## PopulationPristi:TypePredator  0.10009    0.07939   1.261   0.2111    
## ---
## Signif. codes:  0 '***' 0.001 '**' 0.01 '*' 0.05 '.' 0.1 ' ' 1
## 
## Residual standard error: 0.1801 on 79 degrees of freedom
## Multiple R-squared:  0.2641, Adjusted R-squared:  0.2362 
## F-statistic:  9.45 on 3 and 79 DF,  p-value: 2.086e-05
```

```
## Anova Table (Type III tests)
## 
## Response: NorVang
##                 Sum Sq Df    F value    Pr(>F)    
## (Intercept)     461.99  1 14243.2352 < 2.2e-16 ***
## Population        0.56  1    17.1567 8.564e-05 ***
## Type              0.33  1    10.2051  0.002013 ** 
## Population:Type   0.05  1     1.5896  0.211098    
## Residuals         2.56 79                         
## ---
## Signif. codes:  0 '***' 0.001 '**' 0.01 '*' 0.05 '.' 0.1 ' ' 1
```

```
##  contrast                          estimate     SE df t.ratio p.value
##  Galta_Control_vs_Galta_Predator    -0.1144 0.0577 79  -1.982  0.0509
##  Galta_Control_vs_Pristi_Control    -0.0768 0.0545 79  -1.408  0.1632
##  Galta_Predator_vs_Pristi_Predator  -0.1768 0.0577 79  -3.065  0.0030
##  Pristi_Control_vs_Pristi_Predator  -0.2145 0.0545 79  -3.933  0.0002
```

```
## 
## Call:
## lm(formula = Center_Duration ~ Population * Type, data = convstreat)
## 
## Residuals:
##      Min       1Q   Median       3Q      Max 
## -156.105  -53.227    8.613   44.891  150.840 
## 
## Coefficients:
##                               Estimate Std. Error t value Pr(>|t|)    
## (Intercept)                     106.12      14.83   7.154 3.79e-10 ***
## PopulationPristi                 39.77      21.25   1.871  0.06503 .  
## TypePredator                     61.58      20.09   3.066  0.00297 ** 
## PopulationPristi:TypePredator   -51.36      29.24  -1.756  0.08290 .  
## ---
## Signif. codes:  0 '***' 0.001 '**' 0.01 '*' 0.05 '.' 0.1 ' ' 1
## 
## Residual standard error: 66.34 on 79 degrees of freedom
## Multiple R-squared:  0.1148, Adjusted R-squared:  0.08117 
## F-statistic: 3.415 on 3 and 79 DF,  p-value: 0.02137
```

```
## Anova Table (Type III tests)
## 
## Response: Center_Duration
##                  Sum Sq Df  F value  Pr(>F)    
## (Intercept)     1706393  1 387.7372 < 2e-16 ***
## Population         4085  1   0.9282 0.33826    
## Type              26534  1   6.0293 0.01627 *  
## Population:Type   13576  1   3.0848 0.08290 .  
## Residuals        347671 79                     
## ---
## Signif. codes:  0 '***' 0.001 '**' 0.01 '*' 0.05 '.' 0.1 ' ' 1
```

```
##  contrast                          estimate   SE df t.ratio p.value
##  Galta_Control_vs_Galta_Predator      -39.8 21.3 79  -1.871  0.0650
##  Galta_Control_vs_Pristi_Control      -61.6 20.1 79  -3.066  0.0030
##  Galta_Predator_vs_Pristi_Predator    -10.2 21.3 79  -0.481  0.6319
##  Pristi_Control_vs_Pristi_Predator     11.6 20.1 79   0.577  0.5654
```

```
## 
## Call:
## lm(formula = Border_duration ~ Population * Type, data = convstreat)
## 
## Residuals:
##      Min       1Q   Median       3Q      Max 
## -150.837  -44.721   -7.555   52.983  158.898 
## 
## Coefficients:
##                               Estimate Std. Error t value Pr(>|t|)    
## (Intercept)                     193.88      14.96  12.962  < 2e-16 ***
## PopulationPristi                -39.76      21.43  -1.856  0.06725 .  
## TypePredator                    -62.21      20.25  -3.072  0.00292 ** 
## PopulationPristi:TypePredator    49.20      29.49   1.669  0.09915 .  
## ---
## Signif. codes:  0 '***' 0.001 '**' 0.01 '*' 0.05 '.' 0.1 ' ' 1
## 
## Residual standard error: 66.89 on 79 degrees of freedom
## Multiple R-squared:  0.1176, Adjusted R-squared:  0.08409 
## F-statistic: 3.509 on 3 and 79 DF,  p-value: 0.01904
```

```
## Anova Table (Type III tests)
## 
## Response: Border_duration
##                  Sum Sq Df  F value  Pr(>F)    
## (Intercept)     1983277  1 443.2495 < 2e-16 ***
## Population         4733  1   1.0579 0.30683    
## Type              29128  1   6.5099 0.01266 *  
## Population:Type   12458  1   2.7843 0.09915 .  
## Residuals        353478 79                     
## ---
## Signif. codes:  0 '***' 0.001 '**' 0.01 '*' 0.05 '.' 0.1 ' ' 1
```

```
## $emmeans
##  Population Type     emmean   SE df lower.CL upper.CL
##  Galta      Control     194 15.0 79      164      224
##  Pristi     Control     154 15.3 79      124      185
##  Galta      Predator    132 13.7 79      104      159
##  Pristi     Predator    141 15.0 79      111      171
## 
## Confidence level used: 0.95 
## 
## $contrasts
##  contrast                         estimate   SE df t.ratio p.value
##  Galta Control - Pristi Control      39.76 21.4 79   1.856  0.2556
##  Galta Control - Galta Predator      62.21 20.3 79   3.072  0.0152
##  Galta Control - Pristi Predator     52.78 21.2 79   2.495  0.0684
##  Pristi Control - Galta Predator     22.45 20.5 79   1.093  0.6948
##  Pristi Control - Pristi Predator    13.02 21.4 79   0.607  0.9295
##  Galta Predator - Pristi Predator    -9.44 20.3 79  -0.466  0.9663
## 
## P value adjustment: tukey method for comparing a family of 4 estimates
```

```
##  contrast                          estimate   SE df t.ratio p.value
##  Galta_Control_vs_Galta_Predator      39.76 21.4 79   1.856  0.0672
##  Galta_Control_vs_Pristi_Control      62.21 20.3 79   3.072  0.0029
##  Galta_Predator_vs_Pristi_Predator    13.02 21.4 79   0.607  0.5454
##  Pristi_Control_vs_Pristi_Predator    -9.44 20.3 79  -0.466  0.6425
```

##########################Treatment comparing before and after
trigger############################## #table 2 ########Treatment
comparing before and after############# ###########

```
##########################Treatment comparing before and after trigger##############################
#table 2
########Treatment comparing before and after#############
###########
rm(list=ls())
ls()
```

```
## character(0)
```

```
treatBandA <- read.csv("~/Documents/Iceland project/Dta/TreatmentBefore_After.csv")


######### swiming activity###
shapiro.test(treatBandA $Dtot) #no normal
```

```
## 
##  Shapiro-Wilk normality test
## 
## data:  treatBandA$Dtot
## W = 0.9417, p-value = 0.0006245
```

```
shapiro.test(treatBandA $VelBL) #no Normal
```

```
## 
##  Shapiro-Wilk normality test
## 
## data:  treatBandA$VelBL
## W = 0.90656, p-value = 9.916e-06
```

```
shapiro.test(treatBandA $Vang) # no normal
```

```
## 
##  Shapiro-Wilk normality test
## 
## data:  treatBandA$Vang
## W = 0.90006, p-value = 5.057e-06
```

```
shapiro.test(treatBandA $Center_Duration) # normal borderline
```

```
## 
##  Shapiro-Wilk normality test
## 
## data:  treatBandA$Center_Duration
## W = 0.97207, p-value = 0.05411
```

```
shapiro.test(treatBandA $Border_duration) #normal
```

```
## 
##  Shapiro-Wilk normality test
## 
## data:  treatBandA$Border_duration
## W = 0.97685, p-value = 0.1164
```

```
shapiro.test(treatBandA $Dist_to_Pred) #no Normal
```

```
## 
##  Shapiro-Wilk normality test
## 
## data:  treatBandA$Dist_to_Pred
## W = 0.94479, p-value = 0.0009434
```

```
skewness(treatBandA$Dtot, na.rm = TRUE)
```

```
## [1] 0.9480523
```

```
skewness(treatBandA$VelBL, na.rm = TRUE)
```

```
## [1] 1.37403
```

```
NorDtot <- sqrt(treatBandA $Dtot)
NorVelBL <- sqrt(treatBandA $VelBL)
NorVang <- log10(treatBandA $Vang)
NorDpred <- sqrt(treatBandA $Dist_to_Pred)

shapiro.test(NorDtot) #normal
```

```
## 
##  Shapiro-Wilk normality test
## 
## data:  NorDtot
## W = 0.98911, p-value = 0.6801
```

```
shapiro.test(NorVelBL) #normal
```

```
## 
##  Shapiro-Wilk normality test
## 
## data:  NorVelBL
## W = 0.98982, p-value = 0.7312
```

```
shapiro.test(NorVang) #normal
```

```
## 
##  Shapiro-Wilk normality test
## 
## data:  NorVang
## W = 0.99256, p-value = 0.9043
```

```
shapiro.test(NorDpred) #normal
```

```
## 
##  Shapiro-Wilk normality test
## 
## data:  NorDpred
## W = 0.98448, p-value = 0.3773
```

```
treatBandA <- cbind(treatBandA, NorDtot) 
treatBandA <- cbind(treatBandA, NorVelBL) 
treatBandA <- cbind(treatBandA, NorVang) 
treatBandA <- cbind(treatBandA, NorDpred) 


write.table(treatBandA, file="~/Documents/Iceland project/Before-After-predatorNor.csv", col.names=NA,row.names=TRUE, sep=",", quote=FALSE)
```

```
##############################

rm(list=ls())
ls()
```

```
## character(0)
```

```
treatBandA <- read.csv("~/Documents/Iceland project/Before-After-predatorNor.csv")


treatBandA$Population <- as.factor(treatBandA$Population) 
treatBandA$Period <- as.factor(treatBandA$Period) 
treatBandA$Parasite <- as.factor(treatBandA$Parasite) 

fit18.2 <- lmer(NorDtot ~ Population* Period *Parasite  +(1 | Video_nbb), data= treatBandA)
summary(fit18.2)
```

```
## Linear mixed model fit by REML ['lmerMod']
## Formula: NorDtot ~ Population * Period * Parasite + (1 | Video_nbb)
##    Data: treatBandA
## 
## REML criterion at convergence: 571.7
## 
## Scaled residuals: 
##      Min       1Q   Median       3Q      Max 
## -2.20093 -0.43427 -0.01398  0.45739  2.75237 
## 
## Random effects:
##  Groups    Name        Variance Std.Dev.
##  Video_nbb (Intercept) 30.19    5.495   
##  Residual              36.11    6.009   
## Number of obs: 88, groups:  Video_nbb, 44
## 
## Fixed effects:
##                                     Estimate Std. Error t value
## (Intercept)                          18.2051     1.9748   9.219
## PopulationPristi                      0.1233     2.9387   0.042
## PeriodBT                              5.4564     2.0611   2.647
## ParasiteP                             4.7571     3.6567   1.301
## PopulationPristi:PeriodBT            -5.5150     3.0670  -1.798
## PopulationPristi:ParasiteP            0.4693     5.3997   0.087
## PeriodBT:ParasiteP                   -5.2034     3.8164  -1.363
## PopulationPristi:PeriodBT:ParasiteP   3.9818     5.6356   0.707
## 
## Correlation of Fixed Effects:
##             (Intr) PpltnP PerdBT ParstP PpP:PBT PpP:PP PBT:PP
## PopultnPrst -0.672                                           
## PeriodBT    -0.522  0.351                                    
## ParasiteP   -0.540  0.363  0.282                             
## PpltnPr:PBT  0.351 -0.522 -0.672 -0.189                      
## PpltnPrs:PP  0.366 -0.544 -0.191 -0.677  0.284               
## PrdBT:PrstP  0.282 -0.189 -0.540 -0.522  0.363   0.353       
## PplP:PBT:PP -0.191  0.284  0.366  0.353 -0.544  -0.522 -0.677
```

```
contrasts(treatBandA$Population) <- contr.sum 
contrasts(treatBandA$Period) <- contr.sum
contrasts(treatBandA$Parasite) <- contr.sum

Anova(fit18.2, type="III")
```

```
## Analysis of Deviance Table (Type III Wald chisquare tests)
## 
## Response: NorDtot
##                              Chisq Df Pr(>Chisq)    
## (Intercept)                84.9808  1  < 2.2e-16 ***
## Population                  0.0018  1   0.966545    
## Period                      7.0084  1   0.008113 ** 
## Parasite                    1.6924  1   0.193285    
## Population:Period           3.2335  1   0.072148 .  
## Population:Parasite         0.0076  1   0.930740    
## Period:Parasite             1.8590  1   0.172744    
## Population:Period:Parasite  0.4992  1   0.479844    
## ---
## Signif. codes:  0 '***' 0.001 '**' 0.01 '*' 0.05 '.' 0.1 ' ' 1
```

```
######################swiming activity before and after trigger#####################
rm(list=ls())
ls()
```

```
## character(0)
```

```
treatBandA <- read.csv("~/Documents/Iceland project/Before-After-predatorNor.csv")


treatBandA$Population <- as.factor(treatBandA$Population) 
treatBandA$Period <- as.factor(treatBandA$Period) 
treatBandA$Parasite <- as.factor(treatBandA$Parasite) 

fit19.2 <- lmer(NorVelBL ~ Population*Period *Parasite  +(1| Video_nbb), data= treatBandA)
summary(fit19.2)
```

```
## Linear mixed model fit by REML ['lmerMod']
## Formula: NorVelBL ~ Population * Period * Parasite + (1 | Video_nbb)
##    Data: treatBandA
## 
## REML criterion at convergence: -24.3
## 
## Scaled residuals: 
##      Min       1Q   Median       3Q      Max 
## -1.71540 -0.41839 -0.06234  0.41176  2.95656 
## 
## Random effects:
##  Groups    Name        Variance Std.Dev.
##  Video_nbb (Intercept) 0.01926  0.1388  
##  Residual              0.02012  0.1418  
## Number of obs: 88, groups:  Video_nbb, 44
## 
## Fixed effects:
##                                     Estimate Std. Error t value
## (Intercept)                          0.44038    0.04813   9.150
## PopulationPristi                     0.01209    0.07162   0.169
## PeriodBT                             0.12044    0.04865   2.476
## ParasiteP                            0.12466    0.08912   1.399
## PopulationPristi:PeriodBT           -0.12387    0.07239  -1.711
## PopulationPristi:ParasiteP          -0.02137    0.13160  -0.162
## PeriodBT:ParasiteP                  -0.11569    0.09008  -1.284
## PopulationPristi:PeriodBT:ParasiteP  0.08973    0.13302   0.675
## 
## Correlation of Fixed Effects:
##             (Intr) PpltnP PerdBT ParstP PpP:PBT PpP:PP PBT:PP
## PopultnPrst -0.672                                           
## PeriodBT    -0.505  0.340                                    
## ParasiteP   -0.540  0.363  0.273                             
## PpltnPr:PBT  0.340 -0.505 -0.672 -0.183                      
## PpltnPrs:PP  0.366 -0.544 -0.185 -0.677  0.275               
## PrdBT:PrstP  0.273 -0.183 -0.540 -0.505  0.363   0.342       
## PplP:PBT:PP -0.185  0.275  0.366  0.342 -0.544  -0.505 -0.677
```

```
contrasts(treatBandA$Population) <- contr.sum 
contrasts(treatBandA$Period) <- contr.sum
contrasts(treatBandA$Parasite) <- contr.sum

Anova(fit19.2, type="III")
```

```
## Analysis of Deviance Table (Type III Wald chisquare tests)
## 
## Response: NorVelBL
##                              Chisq Df Pr(>Chisq)    
## (Intercept)                83.7242  1    < 2e-16 ***
## Population                  0.0285  1    0.86592    
## Period                      6.1288  1    0.01330 *  
## Parasite                    1.9568  1    0.16186    
## Population:Period           2.9276  1    0.08708 .  
## Population:Parasite         0.0264  1    0.87097    
## Period:Parasite             1.6495  1    0.19903    
## Population:Period:Parasite  0.4551  1    0.49995    
## ---
## Signif. codes:  0 '***' 0.001 '**' 0.01 '*' 0.05 '.' 0.1 ' ' 1
```

```
rm(list=ls())
ls()
```

```
## character(0)
```

```
treatBandA <- read.csv("~/Documents/Iceland project/Before-After-predatorNor.csv")


treatBandA$Population <- as.factor(treatBandA$Population) 
treatBandA$Period <- as.factor(treatBandA$Period) 
treatBandA$Parasite <- as.factor(treatBandA$Parasite) 

fit20 <- lmer(NorVang ~ Population* Period *Parasite  +(1| Video_nbb), data= treatBandA)
summary(fit20)
```

```
## Linear mixed model fit by REML ['lmerMod']
## Formula: NorVang ~ Population * Period * Parasite + (1 | Video_nbb)
##    Data: treatBandA
## 
## REML criterion at convergence: -41.4
## 
## Scaled residuals: 
##      Min       1Q   Median       3Q      Max 
## -2.31215 -0.52716 -0.06751  0.47487  1.83215 
## 
## Random effects:
##  Groups    Name        Variance Std.Dev.
##  Video_nbb (Intercept) 0.01141  0.1068  
##  Residual              0.01858  0.1363  
## Number of obs: 88, groups:  Video_nbb, 44
## 
## Fixed effects:
##                                     Estimate Std. Error t value
## (Intercept)                          2.46510    0.04200  58.692
## PopulationPristi                     0.08237    0.06250   1.318
## PeriodBT                            -0.14152    0.04675  -3.027
## ParasiteP                           -0.08738    0.07777  -1.124
## PopulationPristi:PeriodBT            0.17588    0.06957   2.528
## PopulationPristi:ParasiteP          -0.01778    0.11484  -0.155
## PeriodBT:ParasiteP                   0.09143    0.08657   1.056
## PopulationPristi:PeriodBT:ParasiteP -0.12829    0.12784  -1.003
## 
## Correlation of Fixed Effects:
##             (Intr) PpltnP PerdBT ParstP PpP:PBT PpP:PP PBT:PP
## PopultnPrst -0.672                                           
## PeriodBT    -0.557  0.374                                    
## ParasiteP   -0.540  0.363  0.301                             
## PpltnPr:PBT  0.374 -0.557 -0.672 -0.202                      
## PpltnPrs:PP  0.366 -0.544 -0.204 -0.677  0.303               
## PrdBT:PrstP  0.301 -0.202 -0.540 -0.557  0.363   0.377       
## PplP:PBT:PP -0.204  0.303  0.366  0.377 -0.544  -0.557 -0.677
```

```
contrasts(treatBandA$Population) <- contr.sum 
contrasts(treatBandA$Period) <- contr.sum
contrasts(treatBandA$Parasite) <- contr.sum

Anova(fit20, type="III")
```

```
## Analysis of Deviance Table (Type III Wald chisquare tests)
## 
## Response: NorVang
##                                Chisq Df Pr(>Chisq)    
## (Intercept)                3444.7259  1  < 2.2e-16 ***
## Population                    1.7368  1   0.187545    
## Period                        9.1618  1   0.002471 ** 
## Parasite                      1.2624  1   0.261201    
## Population:Period             6.3904  1   0.011474 *  
## Population:Parasite           0.0240  1   0.876935    
## Period:Parasite               1.1155  1   0.290899    
## Population:Period:Parasite    1.0070  1   0.315630    
## ---
## Signif. codes:  0 '***' 0.001 '**' 0.01 '*' 0.05 '.' 0.1 ' ' 1
```

```
# Obtain the estimated marginal means (EMMs) 
emm <- emmeans(fit20, ~ Population * Period * Parasite) 
contrast_list <- list( "Galta_BT_Np_vs_Galta_AT_Np" = c(1, -1, 0, 0, 0, 0, 0, 0), "Pristi_BT_Np_vs_Pristi_AT_Np" = c(0, 0, 1, -1, 0, 0, 0, 0), "Galta_BT_P_vs_Galta_AT_P" = c(0, 0, 0, 0, 1, -1, 0, 0), "Pristi_BT_P_vs_Pristi_AT_P" = c(0, 0, 0, 0, 0, 0, 1, -1), "Galta_BT_Np_vs_Galta_BT_P" = c(1, 0, -1, 0, 0, 0, 0, 0), "Galta_AT_Np_vs_Galta_AT_P" = c(0, 1, 0, -1, 0, 0, 0, 0), "Pristi_BT_Np_vs_Pristi_BT_P" = c(0, 0, 0, 0, 1, 0, -1, 0), "Pristi_AT_Np_vs_Pristi_AT_P" = c(0, 0, 0, 0, 0, 1, 0, -1) )
# Perform the contrasts 
contrast_results <- contrast(emm, contrast_list) 
summary(contrast_results)
```

```
##  contrast                     estimate     SE   df t.ratio p.value
##  Galta_BT_Np_vs_Galta_AT_Np   -0.08237 0.0625 69.9  -1.318  0.1918
##  Pristi_BT_Np_vs_Pristi_AT_Np -0.25824 0.0625 69.9  -4.132  0.0001
##  Galta_BT_P_vs_Galta_AT_P     -0.06458 0.0963 69.9  -0.670  0.5049
##  Pristi_BT_P_vs_Pristi_AT_P   -0.11217 0.0963 69.9  -1.164  0.2483
##  Galta_BT_Np_vs_Galta_BT_P     0.14152 0.0468 40.0   3.027  0.0043
##  Galta_AT_Np_vs_Galta_AT_P    -0.03436 0.0515 40.0  -0.667  0.5087
##  Pristi_BT_Np_vs_Pristi_BT_P   0.05009 0.0729 40.0   0.687  0.4958
##  Pristi_AT_Np_vs_Pristi_AT_P   0.00249 0.0787 40.0   0.032  0.9749
## 
## Degrees-of-freedom method: kenward-roger
```

```
#########
#####################Tank use swiming activity before and after trigger####################
#########

rm(list=ls())
ls()
```

```
## character(0)
```

```
treatBandA <- read.csv("~/Documents/Iceland project/Before-After-predatorNor.csv")


treatBandA$Population <- as.factor(treatBandA$Population) 
treatBandA$Period <- as.factor(treatBandA$Period) 
treatBandA$Parasite <- as.factor(treatBandA$Parasite) 

fit21.2 <- lmer(Center_Duration ~ Population* Period *Parasite  +(1| Video_nbb), data= treatBandA)

summary(fit21.2)
```

```
## Linear mixed model fit by REML ['lmerMod']
## Formula: Center_Duration ~ Population * Period * Parasite + (1 | Video_nbb)
##    Data: treatBandA
## 
## REML criterion at convergence: 909.3
## 
## Scaled residuals: 
##      Min       1Q   Median       3Q      Max 
## -1.77494 -0.47720 -0.01313  0.44218  2.71116 
## 
## Random effects:
##  Groups    Name        Variance Std.Dev.
##  Video_nbb (Intercept) 1918     43.79   
##  Residual              2533     50.33   
## Number of obs: 88, groups:  Video_nbb, 44
## 
## Fixed effects:
##                                     Estimate Std. Error t value
## (Intercept)                            80.14      16.18   4.953
## PopulationPristi                      -18.16      24.08  -0.754
## PeriodBT                               72.19      17.26   4.182
## ParasiteP                              84.07      29.96   2.806
## PopulationPristi:PeriodBT              15.46      25.69   0.602
## PopulationPristi:ParasiteP            -14.34      44.24  -0.324
## PeriodBT:ParasiteP                    -31.40      31.96  -0.983
## PopulationPristi:PeriodBT:ParasiteP   -16.76      47.20  -0.355
## 
## Correlation of Fixed Effects:
##             (Intr) PpltnP PerdBT ParstP PpP:PBT PpP:PP PBT:PP
## PopultnPrst -0.672                                           
## PeriodBT    -0.533  0.358                                    
## ParasiteP   -0.540  0.363  0.288                             
## PpltnPr:PBT  0.358 -0.533 -0.672 -0.194                      
## PpltnPrs:PP  0.366 -0.544 -0.195 -0.677  0.290               
## PrdBT:PrstP  0.288 -0.194 -0.540 -0.533  0.363   0.361       
## PplP:PBT:PP -0.195  0.290  0.366  0.361 -0.544  -0.533 -0.677
```

```
contrasts(treatBandA$Population) <- contr.sum 
contrasts(treatBandA$Period) <- contr.sum
contrasts(treatBandA$Parasite) <- contr.sum

Anova(fit21.2, type="III")
```

```
## Analysis of Deviance Table (Type III Wald chisquare tests)
## 
## Response: Center_Duration
##                              Chisq Df Pr(>Chisq)    
## (Intercept)                24.5355  1  7.295e-07 ***
## Population                  0.5692  1   0.450587    
## Period                     17.4916  1  2.886e-05 ***
## Parasite                    7.8749  1   0.005013 ** 
## Population:Period           0.3624  1   0.547187    
## Population:Parasite         0.1051  1   0.745830    
## Period:Parasite             0.9654  1   0.325827    
## Population:Period:Parasite  0.1261  1   0.722489    
## ---
## Signif. codes:  0 '***' 0.001 '**' 0.01 '*' 0.05 '.' 0.1 ' ' 1
```

```
# Obtain the estimated marginal means (EMMs) 
emm <- emmeans(fit21.2, ~ Population * Period * Parasite) # Specify the relevant pairwise comparisons 

contrast_list <- list( "Galta_BT_Np_vs_Galta_AT_Np" = c(1, -1, 0, 0, 0, 0, 0, 0), "Pristi_BT_Np_vs_Pristi_AT_Np" = c(0, 0, 1, -1, 0, 0, 0, 0), "Galta_BT_P_vs_Galta_AT_P" = c(0, 0, 0, 0, 1, -1, 0, 0), "Pristi_BT_P_vs_Pristi_AT_P" = c(0, 0, 0, 0, 0, 0, 1, -1), "Galta_BT_Np_vs_Galta_BT_P" = c(1, 0, -1, 0, 0, 0, 0, 0), "Galta_AT_Np_vs_Galta_AT_P" = c(0, 1, 0, -1, 0, 0, 0, 0), "Pristi_BT_Np_vs_Pristi_BT_P" = c(0, 0, 0, 0, 1, 0, -1, 0), "Pristi_AT_Np_vs_Pristi_AT_P" = c(0, 0, 0, 0, 0, 1, 0, -1) )
```

```
#########

rm(list=ls())
ls()
```

```
## character(0)
```

```
treatBandA <- read.csv("~/Documents/Iceland project/Before-After-predatorNor.csv")


treatBandA$Population <- as.factor(treatBandA$Population) 
treatBandA$Period <- as.factor(treatBandA$Period) 
treatBandA$Parasite <- as.factor(treatBandA$Parasite) 

fit22.2 <- lmer(Border_duration ~ Population* Period*Parasite  +(1| Video_nbb), data= treatBandA)
summary(fit22.2)
```

```
## Linear mixed model fit by REML ['lmerMod']
## Formula: Border_duration ~ Population * Period * Parasite + (1 | Video_nbb)
##    Data: treatBandA
## 
## REML criterion at convergence: 914.4
## 
## Scaled residuals: 
##      Min       1Q   Median       3Q      Max 
## -2.20702 -0.40060  0.05073  0.42543  2.00728 
## 
## Random effects:
##  Groups    Name        Variance Std.Dev.
##  Video_nbb (Intercept) 2841     53.30   
##  Residual              2296     47.91   
## Number of obs: 88, groups:  Video_nbb, 44
## 
## Fixed effects:
##                                     Estimate Std. Error t value
## (Intercept)                          195.509     17.383  11.247
## PopulationPristi                      30.693     25.867   1.187
## PeriodBT                             -48.446     16.435  -2.948
## ParasiteP                            -68.786     32.188  -2.137
## PopulationPristi:PeriodBT            -30.252     24.456  -1.237
## PopulationPristi:ParasiteP             7.766     47.531   0.163
## PeriodBT:ParasiteP                    15.995     30.431   0.526
## PopulationPristi:PeriodBT:ParasiteP   23.685     44.937   0.527
## 
## Correlation of Fixed Effects:
##             (Intr) PpltnP PerdBT ParstP PpP:PBT PpP:PP PBT:PP
## PopultnPrst -0.672                                           
## PeriodBT    -0.473  0.318                                    
## ParasiteP   -0.540  0.363  0.255                             
## PpltnPr:PBT  0.318 -0.473 -0.672 -0.172                      
## PpltnPrs:PP  0.366 -0.544 -0.173 -0.677  0.257               
## PrdBT:PrstP  0.255 -0.172 -0.540 -0.473  0.363   0.320       
## PplP:PBT:PP -0.173  0.257  0.366  0.320 -0.544  -0.473 -0.677
```

```
contrasts(treatBandA$Population) <- contr.sum 
contrasts(treatBandA$Period) <- contr.sum
contrasts(treatBandA$Parasite) <- contr.sum

Anova(fit22.2, type="III")
```

```
## Analysis of Deviance Table (Type III Wald chisquare tests)
## 
## Response: Border_duration
##                               Chisq Df Pr(>Chisq)    
## (Intercept)                126.4922  1     <2e-16 ***
## Population                   1.4079  1     0.2354    
## Period                       8.6895  1     0.0032 ** 
## Parasite                     4.5669  1     0.0326 *  
## Population:Period            1.5302  1     0.2161    
## Population:Parasite          0.0267  1     0.8702    
## Period:Parasite              0.2763  1     0.5992    
## Population:Period:Parasite   0.2778  1     0.5981    
## ---
## Signif. codes:  0 '***' 0.001 '**' 0.01 '*' 0.05 '.' 0.1 ' ' 1
```

```
emm <- emmeans(fit22.2, ~ Population * Period * Parasite) # Specify the relevant pairwise comparisons 

contrast_list <- list( "Galta_BT_Np_vs_Galta_AT_Np" = c(1, -1, 0, 0, 0, 0, 0, 0), "Pristi_BT_Np_vs_Pristi_AT_Np" = c(0, 0, 1, -1, 0, 0, 0, 0), "Galta_BT_P_vs_Galta_AT_P" = c(0, 0, 0, 0, 1, -1, 0, 0), "Pristi_BT_P_vs_Pristi_AT_P" = c(0, 0, 0, 0, 0, 0, 1, -1), "Galta_BT_Np_vs_Galta_BT_P" = c(1, 0, -1, 0, 0, 0, 0, 0), "Galta_AT_Np_vs_Galta_AT_P" = c(0, 1, 0, -1, 0, 0, 0, 0), "Pristi_BT_Np_vs_Pristi_BT_P" = c(0, 0, 0, 0, 1, 0, -1, 0), "Pristi_AT_Np_vs_Pristi_AT_P" = c(0, 0, 0, 0, 0, 1, 0, -1) )
# Perform the contrasts 
contrast_results <- contrast(emm, contrast_list) 
summary(contrast_results)
```

```
##  contrast                     estimate   SE   df t.ratio p.value
##  Galta_BT_Np_vs_Galta_AT_Np    -30.693 25.9 61.3  -1.187  0.2400
##  Pristi_BT_Np_vs_Pristi_AT_Np   -0.441 25.9 61.3  -0.017  0.9864
##  Galta_BT_P_vs_Galta_AT_P      -38.459 39.9 61.3  -0.964  0.3386
##  Pristi_BT_P_vs_Pristi_AT_P    -31.892 39.9 61.3  -0.800  0.4269
##  Galta_BT_Np_vs_Galta_BT_P      48.446 16.4 40.0   2.948  0.0053
##  Galta_AT_Np_vs_Galta_AT_P      78.698 18.1 40.0   4.346  0.0001
##  Pristi_BT_Np_vs_Pristi_BT_P    32.451 25.6 40.0   1.267  0.2125
##  Pristi_AT_Np_vs_Pristi_AT_P    39.019 27.7 40.0   1.410  0.1661
## 
## Degrees-of-freedom method: kenward-roger
```

```
#########

rm(list=ls())
ls()
```

```
## character(0)
```

```
treatBandA <- read.csv("~/Documents/Iceland project/Before-After-predatorNor.csv")


treatBandA$Population <- as.factor(treatBandA$Population) 
treatBandA$Period <- as.factor(treatBandA$Period) 
treatBandA$Parasite <- as.factor(treatBandA$Parasite) 

fit23 <- lmer(NorDpred ~ Population* Period*Parasite  +(1| Video_nbb), data= treatBandA)
summary(fit23)
```

```
## Linear mixed model fit by REML ['lmerMod']
## Formula: NorDpred ~ Population * Period * Parasite + (1 | Video_nbb)
##    Data: treatBandA
## 
## REML criterion at convergence: 329.3
## 
## Scaled residuals: 
##      Min       1Q   Median       3Q      Max 
## -1.97172 -0.61247 -0.09845  0.59929  1.79182 
## 
## Random effects:
##  Groups    Name        Variance Std.Dev.
##  Video_nbb (Intercept) 0.9687   0.9842  
##  Residual              2.0440   1.4297  
## Number of obs: 88, groups:  Video_nbb, 44
## 
## Fixed effects:
##                                     Estimate Std. Error t value
## (Intercept)                          5.32292    0.42097  12.644
## PopulationPristi                     0.08641    0.62643   0.138
## PeriodBT                             0.12406    0.49038   0.253
## ParasiteP                           -0.32829    0.77949  -0.421
## PopulationPristi:PeriodBT            0.46214    0.72971   0.633
## PopulationPristi:ParasiteP           0.30100    1.15105   0.261
## PeriodBT:ParasiteP                  -0.07437    0.90801  -0.082
## PopulationPristi:PeriodBT:ParasiteP -0.55779    1.34084  -0.416
## 
## Correlation of Fixed Effects:
##             (Intr) PpltnP PerdBT ParstP PpP:PBT PpP:PP PBT:PP
## PopultnPrst -0.672                                           
## PeriodBT    -0.582  0.391                                    
## ParasiteP   -0.540  0.363  0.315                             
## PpltnPr:PBT  0.391 -0.582 -0.672 -0.211                      
## PpltnPrs:PP  0.366 -0.544 -0.213 -0.677  0.317               
## PrdBT:PrstP  0.315 -0.211 -0.540 -0.582  0.363   0.394       
## PplP:PBT:PP -0.213  0.317  0.366  0.394 -0.544  -0.582 -0.677
```

```
contrasts(treatBandA$Population) <- contr.sum 
contrasts(treatBandA$Period) <- contr.sum
contrasts(treatBandA$Parasite) <- contr.sum

Anova(fit23, type="III")
```

```
## Analysis of Deviance Table (Type III Wald chisquare tests)
## 
## Response: NorDpred
##                               Chisq Df Pr(>Chisq)    
## (Intercept)                159.8779  1     <2e-16 ***
## Population                   0.0190  1     0.8903    
## Period                       0.0640  1     0.8003    
## Parasite                     0.1774  1     0.6736    
## Population:Period            0.4011  1     0.5265    
## Population:Parasite          0.0684  1     0.7937    
## Period:Parasite              0.0067  1     0.9347    
## Population:Period:Parasite   0.1731  1     0.6774    
## ---
## Signif. codes:  0 '***' 0.001 '**' 0.01 '*' 0.05 '.' 0.1 ' ' 1
```
