# Supplementary material for: Interplay between antipredator behavior, parasitism, and gut microbiome in wild stickleback populations
Source: NPJ Biofilms Microbiomes. 2025 Jul 19;11:138. doi: 10.1038/s41522-025-00758-y (PMC12276351; doi:10.1038/s41522-025-00758-y)
Supplement: Supplementary file 2 — Supplementary data [file 41522_2025_758_MOESM2_ESM.zip › Supplementary Data 5_Rmd_sticklebacks_kraken.html]

Stickleback microbiome & behavior analysis


# Stickleback microbiome & behavior analysis

#### Jaelle Brealey

#### February 18, 2025

- Processing
  steps
  - Read counts
  - Taxa QC
  - Contam identification
  - Final
    filtering
- Dataset
  summaries
- Community
  analyses
  - Top taxa
    summary
    - Figure 4
  - Alpha
    diversity
  - Ordination
    - Limma for batch effects
    - Figure 5
- Behaviour data
  - Figure 2
  - Figure 3
- Association testing with
  Maaslin2
- MOFA
  - controls
  - predator-exposed
  - Main figures
    - Figure 6
    - Figure 7
      TypeP F1
    - Figure 8
      TypeP F3
    - Figure 9
      TypeP F4
  - Supplementary figures
    - TypeC F1
    - TypeC F2
    - TypeC F3
    - TypeC F4
    - TypeC F5
    - TypeC F6
    - TypeC F7
    - TypeP F2
    - TypeP F5
    - TypeP F6
    - TypeP F7
- Taxa
  investigations
- Supp File S1
- Versions

```
set.seed(6854)

### Useful functions ###
# abundance filtering function
abundance_filter_f <- function(count.mat, cutoff) {
  #count.mat must be in format of samples = rows and taxa = columns
  #row.names must be sample IDs
  #cutoff must be proportion (e.g. 0.001 = 0.1% relative abundance)
  count.filt <- count.mat
  prop.mat <- as.data.frame(prop.table(as.matrix(count.filt), margin = 1))
  prop.mat$Sample <- row.names(prop.mat)
  prop.mat.m <- melt(prop.mat, by="Sample", value.name = "Proportion", variable.name = "Taxon")
  samples <- as.character(prop.mat$Sample)
  for (s in samples) {
    exclude.taxa <- as.vector(subset(prop.mat.m, Sample==s & Proportion < cutoff)$Taxon)
    count.filt[s,exclude.taxa] = 0
  }
  return(count.filt)
}

rank_summary <- function(ps, rank, NArm=FALSE){
  # summarises at specified rank
  # takes phyloseq object, returns dataframe
  ps.rank <- tax_glom(ps, taxrank = rank, NArm = NArm)
  df.rank <- as.data.frame(otu_table(ps.rank))
  names(df.rank) <- as.character(tax_table(ps.rank)[,rank])
  names(df.rank)[is.na(names(df.rank))] <- "Unknown"
  df.rank$Total.ct <- rowSums(df.rank)
  return(df.rank)
}

top_taxa <- function(ct.df, sample_var = "SampleID.R", unknown_var = "Unassigned", top = 9) {
  # calculates the top x taxa for taxa summarised at a taxonomic level
  # ct.df = dataframe: samples ~ sum_rank, value = count
  sums <- colSums(ct.df[,which(!names(ct.df) %in% c(sample_var,unknown_var))])
  sums.desc <- names(sums[order(sums, decreasing = T)])
  sums.1 <- ct.df[,c(sample_var,sums.desc[1:top])]
  sums.2 <- data.frame(Other=rowSums(ct.df[,sums.desc[(top+1):length(sums.desc)]]))
  if(unknown_var %in% names(ct.df)){
    top.df <- cbind.data.frame(sums.1, sums.2, Unassigned=ct.df[,unknown_var])
  } else {
    top.df <- cbind.data.frame(sums.1, sums.2)
  }
  return(top.df)
}

ps_ordination_f <- function(df, method, dist, k.range, rds.name){
  # v1 performs ordination using phyloseq command for given k range
  # v2 performs ordination using vegan command for given k range
  # returns the lowest k for which the model converged
  # only tested with NMDS so far
  # only run once - if file exists, save and import
  if(file.exists(rds.name)){
    ord.out <- readRDS(rds.name)
    return(ord.out)
  } else {
    if(is.na(k.range) & class(df) == "phyloseq"){
      # PCoA - less complicated, easier to run
      ord <- ordinate(df, method = method, distance = dist)
      saveRDS(ord, file = rds.name)
      return(ord)
    } else {
      for (k in k.range){
        if(class(df) == "dist"){
        # assume vegan
        ord <- metaMDS(df, k = k, try = 20, trymax = 100, maxit = 2000)
        } else if(class(df) == "phyloseq") {
          # phyloseq
          ord <- ordinate(df, method = method, distance = dist, k = k, try = 20, trymax = 100, maxit = 2000)
        } else {
          stop("Incorrect input format.")
        }
        if(ord$converged){
          ord.out <- list(ord = ord, k = k)
          saveRDS(ord.out, file = rds.name)
          return(ord.out)
        }
      }
      stop("No convergence for provided k values.")
    }
  }
}

run_maaslin2 <- function(ps, out_name, tax.tab, f_eff, r_eff = NA, n = "none", t = "none", taxa_level = "g"){
  # run Maaslin2
  if(file.exists(paste0(out_name,"/all_results.tsv"))){
    maas.out <- read.delim(paste0(out_name,"/all_results.tsv"))
  } else {
    if(is.na(r_eff)){
      maas.out <- Maaslin2(
        input_data = data.frame(otu_table(ps)),
        input_metadata = data.frame(sample_data(ps)),
        fixed_effects = f_eff,
        output = out_name,
        normalization = n, transform = t,
      )
    } else {
      maas.out <- Maaslin2(
        input_data = data.frame(otu_table(ps)),
        input_metadata = data.frame(sample_data(ps)),
        fixed_effects = f_eff,
        random_effects = r_eff,
        output = out_name,
        normalization = n, transform = t,
      )
    }
    maas.out <- maas.out$results
  }
  maas.out$taxonomy <- sapply(maas.out$feature, function(x){
    tax.tab[which(tax.tab$taxon == x),taxa_level]
  })
  maas.out <- maas.out[order(maas.out$metadata, maas.out$feature, maas.out$qval),]
  return(maas.out)
}

run_mofa_default <- function(df.input, outfile, metadata, covar, view.distrib, num_factors = 10){
  if(file.exists(paste0(outfile,".hdf5"))){
    # Load existing MOFA model
    mofa.obj.trained <- load_model(paste0(outfile,".hdf5"))
    mofa.corr <- read.csv(paste0(outfile,"_covariates_corr.csv"))
    mofa.weights <- read.csv(paste0(outfile,"_feature_weights.csv"))
    mofa.samples <- read.csv(paste0(outfile,"_sample_weights.csv"))
    
  } else {
    # run MOFA model
    # Set up
    mofa.obj <- create_mofa(df.input)
    
    # Define parameters
    opts.data <- get_default_data_options(mofa.obj)
    opts.data$scale_views <- TRUE # scale to make sure different views have similar variances
    opts.model <- get_default_model_options(mofa.obj)
    opts.model$num_factors <- num_factors
    # correct view model distributions
    # note views are sorted in alphabetic order by name, so distributions need to match
    if(length(view.distrib) != length(names(opts.model$likelihoods))){
      stop("No. of view distributions do not match number of views")
    }
    opts.model$likelihoods <- sapply(seq(1,length(view.distrib)), function(i) { 
      opts.model$likelihoods[i] <- view.distrib[i]
    })
    opts.train <- get_default_training_options(mofa.obj) #note for final model, switch convergence mode from 'fast' to 'medium' or 'slow'
    
    # Build and train model
    
    mofa.obj <- prepare_mofa(
      object = mofa.obj,
      data_options = opts.data,
      model_options = opts.model,
      training_options = opts.train
    )
    
    mofa.obj.trained <- run_mofa(mofa.obj, paste0(outfile,".hdf5"), use_basilisk = T)
    
    # Add metadata to MOFA object
    samples_metadata(mofa.obj.trained) <- metadata[samples_names(mofa.obj.trained)[[1]],]
    
    # save variances
    write.csv(get_variance_explained(mofa.obj.trained)$r2_total$single_group,
              paste0(outfile,"_variance_total.csv"),
              row.names = T, quote = F)
    write.csv(get_variance_explained(mofa.obj.trained)$r2_per_factor$single_group,
              paste0(outfile,"_variance_per_factor.csv"),
              row.names = T, quote = F)
    
    # save correlations with covariates
    mofa.corr <- merge(
      x = melt(correlate_factors_with_covariates(mofa.obj.trained,
                                                 covariates = covar,
                                                 plot="r", return_data = T), 
               varnames = c("Factor","Phenotype"), value.name = "cor.r"),
      y = melt(correlate_factors_with_covariates(mofa.obj.trained,
                                                 covariates = covar,
                                                 plot="log_pval", return_data = T), 
               varnames = c("Factor","Phenotype"), value.name = "cor.logp"),
      by = c("Factor","Phenotype")
    )
    mofa.corr$cor.p <- 10^(-mofa.corr$cor.logp)
    
    write.csv(mofa.corr, paste0(outfile,"_covariates_corr.csv"),
              row.names = F, quote = F)
    
    # save feature weights
    mofa.weights <- get_weights(
      mofa.obj.trained,
      views = "all",
      factors = "all",
      as.data.frame = TRUE,
      scale = F
    )
    write.csv(mofa.weights, paste0(outfile,"_feature_weights.csv"),
              row.names = F, quote = F)
    
    # save factor values for samples
    mofa.samples <- get_factors(mofa.obj.trained, as.data.frame = T)
    write.csv(mofa.samples, paste0(outfile,"_sample_weights.csv"),
              row.names = F, quote = F)    
  }
  
  # return list of output
  return(list(trained.obj=mofa.obj.trained, corr=mofa.corr, feature.weights=mofa.weights, sample.weights=mofa.samples))
}

get_feature_abundance_meta <- function(abund.mat, feature.ids, meta, sample.var = "Sample", sample.ids = NULL){
  # extract out abundance values for a specific set of genes/samples from an abundance matrix and return with supplied metadata in long format for plots, etc
  # abundance matrix should be features as rows, samples as columns
  # if sample.ids is NULL, use all
  if(is.null(sample.ids)){
    sample.ids <- colnames(abund.mat)
  }
  if(is.matrix(abund.mat)){
    if(length(feature.ids) == 1) {
      fid <- feature.ids
      df <- data.frame(Sample=names(abund.mat[fid,sample.ids]),
                       feature=fid,
                       abundance=abund.mat[fid,sample.ids])
      names(df)[1] <- sample.var
      df.long <- merge(df, meta, by = sample.var, all.x = T, all.y = F)
    } else {
      df <- abund.mat[feature.ids,sample.ids]
      df.long <- reshape2::melt(df, varnames = c("feature",sample.var), value.name = "abundance")
      df.long <- merge(df.long, meta, by = sample.var, all.x = T, all.y = F)
    }
    return(df.long)
  } else {
    stop("abund.mat is not a matrix")
  }
}

# plot MOFA results figure
mofa_figure_ind_factor <- function(filename, factor.name, feature.num.pos, feature.num.neg){
  feature.weights <- read.csv(filename)
  
  feature.weights.beh <- subset(feature.weights, factor == factor.name & view == "Behaviour")
  feature.weights.micro <- subset(feature.weights, factor == factor.name & view == "Microbiome")
  
  feature.weights.beh$rank <- paste(feature.weights.beh$feature,feature.weights.beh$factor, sep = "_")
  feature.weights.beh$rank <- factor(feature.weights.beh$rank, 
                                     levels = feature.weights.beh[order(feature.weights.beh$value, decreasing = F),"rank"])
  
  feature.weights.micro$rank <- paste(feature.weights.micro$feature,feature.weights.micro$factor, sep = "_")
  feature.weights.micro$rank <- factor(feature.weights.micro$rank, 
                                       levels = feature.weights.micro[order(feature.weights.micro$value, decreasing = F),"rank"])
  
  # top contributing to variation in either direction - only works on a per-factor basis though
  feature.weights.beh.positive <- subset(feature.weights.beh, value > 0)
  feature.weights.beh.positive <- feature.weights.beh.positive[order(feature.weights.beh.positive$value, decreasing = T)[1:feature.num.pos],"feature"]
  
  feature.weights.beh.negative <- subset(feature.weights.beh, value < 0)
  feature.weights.beh.negative <- feature.weights.beh.negative[order(feature.weights.beh.negative$value, decreasing = F)[1:feature.num.neg],"feature"]
  
  feature.weights.beh$anno <- sapply(feature.weights.beh$feature, function(x){
    if(x %in% c(feature.weights.beh.positive, feature.weights.beh.negative)){
      x
    } else {
      ""
    }
  })
  feature.weights.beh$anno <- factor(
    feature.weights.beh$anno, 
    levels = sort(unique(feature.weights.beh$anno))[c(2:sum(feature.num.pos,feature.num.neg,1),1)])
  
  # top contributing to variation in either direction
  feature.weights.micro.positive <- subset(feature.weights.micro, value > 0)
  feature.weights.micro.positive <- feature.weights.micro.positive[order(feature.weights.micro.positive$value, decreasing = T)[1:feature.num.pos],"feature"]
  
  feature.weights.micro.negative <- subset(feature.weights.micro, value < 0)
  feature.weights.micro.negative <- feature.weights.micro.negative[order(feature.weights.micro.negative$value, decreasing = F)[1:feature.num.neg],"feature"]
  
  feature.weights.micro$anno <- sapply(feature.weights.micro$feature, function(x){
    if(x %in% c(feature.weights.micro.positive, feature.weights.micro.negative)){
      x
    } else {
      ""
    }
  })
  feature.weights.micro$anno <- factor(
    feature.weights.micro$anno, 
    levels = sort(unique(feature.weights.micro$anno))[c(2:sum(feature.num.pos,feature.num.neg,1),1)])
  return(list(beh=feature.weights.beh, micro=feature.weights.micro,
              beh.top.pos=feature.weights.beh.positive, beh.top.neg=feature.weights.beh.negative,
              micro.top.pos=feature.weights.micro.positive, micro.top.neg=feature.weights.micro.negative))
}

# emulate ggplot2 default colors
gg_color_hue <- function(n) {
  hues = seq(15, 375, length = n + 1)
  hcl(h = hues, l = 65, c = 100)[1:n]
}

# emulate ggplot2 default ordered factor colors
gg_color_ordered <- function(n) {
  viridis::viridis(n)
}
```

# Processing steps

- Adapter removal, duplicate removal, mapping to reference
  genomes
- Taxonomy assignment with Kraken2

```
# Load and process data

meta <- read.csv("metadata_sticklebacks_2021-12-14.csv")
# add in additional behavioral variable
beh.lat <- read.delim("Latencytimetoresumeswimming.txt")
meta <- merge(meta, beh.lat, by = "Video_nb", all.x = T)
row.names(meta) <- meta$SampleID

# Kraken counts
kraken.counts <- read.delim("kraken2_otu_table_sticklebacks_GS_201207.txt", sep = '\t', skip = 1)
names(kraken.counts) <- append("taxon",gsub("\\_.*","",colnames(kraken.counts[,2:ncol(kraken.counts)])))
row.names(kraken.counts) <- kraken.counts$taxon
kraken.counts <- kraken.counts[,2:ncol(kraken.counts)] #remove taxon column from dataframe

# Kraken taxonomy (post-filtering steps)
filt.taxa <- read.csv("taxa_table_kraken2_otu_table_sticklebacks_GS_201207_filt.txt")
row.names(filt.taxa) <- filt.taxa$taxon

# Bracken pre-filtering totals
meta$Bracken.readct.total <- sapply(meta$SampleID, function(x){ 
  sum(kraken.counts[,as.character(x)])
})
meta$Bracken.richness.unfilt <- sapply(as.character(meta$SampleID), function(x) {
  specnumber(kraken.counts[,x])
})
meta$Blank <- meta$Type == "blank neg"
meta$Population2 <- as.character(meta$Population)
meta[meta$Parasite == "P","Population2"] <- "Galta_P"

# assign blanks DNA concentration value for decontam (all were below detection of Nanodrop)
meta[which(meta$Blank),"DNA.ng.ul"] <- c(rep(0.01,5),0.1)
```

## Read counts

```
plot.reads.1 <- ggplot(meta, aes(Population2, Reads.sequenced, color = Population2))+
  geom_boxplot(outlier.colour = NA)+
  geom_point(size = 2, position = position_jitterdodge())+
  scale_y_continuous(limits = c(0,2.5e7))+
  labs(title = "Total sequenced", y = "No. of reads")+
  theme_classic()+
  theme(axis.text = element_text(colour = "black"),
        legend.position = "none")

plot.reads.2 <- ggplot(meta, aes(Population2, Reads.P5.host, color = Population2))+
  geom_boxplot(outlier.colour = NA)+
  geom_point(size = 2, position = position_jitterdodge())+
  scale_y_continuous(limits = c(0,2.5e7))+
  labs(title = "Host reads", y = "No. of reads")+
  theme_classic()+
  theme(axis.text = element_text(colour = "black"),
        legend.position = "none")

plot.reads.3 <- ggplot(meta, aes(Population2, Reads.P5.unmapped, color = Population2))+
  geom_boxplot(outlier.colour = NA)+
  geom_point(size = 2, position = position_jitterdodge())+
  labs(title = "Non-host reads", y = "No. of reads")+
  theme_classic()+
  theme(axis.text = element_text(colour = "black"),
        legend.position = "none")

plot.reads.4 <- ggplot(meta, aes(Population2, Bracken.readct.total, color = Population2))+
  geom_boxplot(outlier.colour = NA)+
  geom_point(size = 2, position = position_jitterdodge())+
  labs(title = "Microbial reads", y = "No. of reads")+
  theme_classic()+
  theme(axis.text = element_text(colour = "black"),
        legend.position = "")

cowplot::plot_grid(plot.reads.1, plot.reads.2, plot.reads.3, plot.reads.4, nrow = 1)
```

```
print("Summary: Total reads sequenced")
```

```
## [1] "Summary: Total reads sequenced"
```

```
summary(meta[!meta$Blank,"Reads.sequenced"])
```

```
##     Min.  1st Qu.   Median     Mean  3rd Qu.     Max. 
##  7315750  9394428 10258260 11416501 12416039 24198056
```

```
print("Summary: Host reads")
```

```
## [1] "Summary: Host reads"
```

```
summary(meta[!meta$Blank,"Reads.P5.host"])
```

```
##     Min.  1st Qu.   Median     Mean  3rd Qu.     Max. 
##  5721068  7209721  7945694  8769828  9383678 18803322
```

```
print("Summary: Non-host reads")
```

```
## [1] "Summary: Non-host reads"
```

```
summary(meta[!meta$Blank,"Reads.P5.unmapped"])
```

```
##    Min. 1st Qu.  Median    Mean 3rd Qu.    Max. 
##   68799   93061  111825  130854  152945  276597
```

```
print("Summary: Microbial reads")
```

```
## [1] "Summary: Microbial reads"
```

```
summary(meta[!meta$Blank,"Bracken.readct.total"])
```

```
##    Min. 1st Qu.  Median    Mean 3rd Qu.    Max. 
##    5455    7490    9169   11686   12149   66166
```

## Taxa QC

```
### Taxa QC ### 
# apply abundance filter of 0.05% to remove false positives 
abund.thresh <- 0.0005
counts.filt <- abundance_filter_f(as.data.frame(t(kraken.counts)), abund.thresh)
# output: samples = rows, taxa = columns 
counts.filt <- counts.filt[,colSums(counts.filt) > 0] # remove taxa now not present in dataframe

ps.filt.taxa <- tax_table(as.matrix(filt.taxa[,2:8]))
filt.ps <- phyloseq(otu_table(as.matrix(counts.filt), taxa_are_rows = FALSE),
                    sample_data(meta),
                    tax_table(ps.filt.taxa))
sample_data(filt.ps)$Bracken.readct.filt <- sample_sums(filt.ps)
sample_data(filt.ps)$Bracken.richness.filt <- specnumber(otu_table(filt.ps))
```

Pre-contam filtering community composition

```
filt.df.genus <- rank_summary(filt.ps, "g")
filt.df.genus$SampleID <- row.names(filt.df.genus)
# exclude human
filt.df.genus <- filt.df.genus[,which(!names(filt.df.genus) == "Homo")]
filt.df.genus <- filt.df.genus[,c(ncol(filt.df.genus),1:(ncol(filt.df.genus)-2))]
filt.df.genus$Unassigned <- rowSums(filt.df.genus[,grep("Unknown",names(filt.df.genus))])
filt.df.genus <- filt.df.genus[,grep("Unknown", names(filt.df.genus), invert = T)]
filt.df.genus.top <- top_taxa(filt.df.genus, sample_var = "SampleID", top = 20)
filt.df.genus.top <- melt(filt.df.genus.top, id.vars = "SampleID", value.name = "count", variable.name = "taxon")
filt.df.genus.top <- merge(filt.df.genus.top, meta, by = "SampleID", all.x = T, all.y = F)

filt.df.genus.top$Ext.date <- factor(filt.df.genus.top$Ext.date, levels = c("22_04_2020","23_04_2020","30_04_2020",
                                                                            "13_05_2020","14_05_2020","15_05_2020"))
```

```
ggplot(filt.df.genus.top, aes(Population2, count, fill=taxon))+
  geom_col(position="fill", width = 1)+
  labs(x="Sample type", y="Relative abundance", fill="Genus")+
  scale_fill_manual(values=c("#F8766D", "#002255ff", "#abc837ff", "#ffe680ff", "#ffcc00ff", "#e38200ff", "#d40055ff", 
            "purple3", "#a02c2cff", "#de87cdff", "mediumseagreen", "#aa0088ff", "#00d455ff", "#00ccffff", 
            "#d42affff", "darkgreen", "#0044aaff",  "#2a7fffff", "#aaccffff", "#73b3c2ff","#969696","#000000"))+
  scale_x_discrete(labels = c("blank","Galtaból","Galtaból_P","Þristikla"))+
  theme(panel.grid.major = element_blank(), panel.grid.minor = element_blank(), panel.background = element_blank(), axis.line = element_line(colour = "black"),
        axis.title = element_text(size = 13), axis.text = element_text(color = "black", size = 12),
        axis.text.x = element_text(angle = 90),
        legend.position = "right")+
  facet_wrap(~Ext.date, scales = "free_x", nrow = 1)
```

```
ggsave("figures_2024/Supp_figure_S1_microbiome_composition_before_contam.png",units = "in", dpi = 600, width = 12, height = 5)
```

```
filt.ps.clr <- transform_sample_counts(filt.ps, function(OTU) OTU+1) #pseudo-count
filt.ps.clr <- microbiome::transform(filt.ps.clr, 'clr')
sample_data(filt.ps.clr)$Ext.date <- factor(sample_data(filt.ps.clr)$Ext.date, 
                                              levels = c("22_04_2020","23_04_2020","30_04_2020",
                                                         "13_05_2020","14_05_2020","15_05_2020"))
ord.filt.sp.clr <- ps_ordination_f(filt.ps.clr, "PCoA", "euclidean", k.range = NA, "ordinations/PCoA_euc_pre_decontam_clr_genus.rds")

plot_ordination(filt.ps.clr, ord.filt.sp.clr, type="samples", axes=c(1,2), color="Ext.date", shape = "Blank")+
  geom_vline(xintercept = 0, linetype = 3, size = 1, color = "grey42")+
  geom_hline(yintercept = 0, linetype = 3, size = 1, color = "grey42")+
  geom_point(size=4)+
  # geom_text(aes(label = Population2), color="black")+
  labs(title = "Before contamination filtering", color = "Extraction date")+
  theme_bw()+
  theme(panel.grid = element_blank(), legend.position = "right",
        axis.text = element_text(size = 12, colour = "black"), axis.title = element_text(size = 13),
        panel.border = element_rect(size = 1.2))
```

```
## Warning: Using `size` aesthetic for lines was deprecated in ggplot2 3.4.0.
## ℹ Please use `linewidth` instead.
## This warning is displayed once every 8 hours.
## Call `lifecycle::last_lifecycle_warnings()` to see where this warning was
## generated.
```

```
## Warning: The `size` argument of `element_rect()` is deprecated as of ggplot2 3.4.0.
## ℹ Please use the `linewidth` argument instead.
## This warning is displayed once every 8 hours.
## Call `lifecycle::last_lifecycle_warnings()` to see where this warning was
## generated.
```

```
ggsave("figures_2024/Supp_figure_S2_ordination_before_contam_v2.png",units = "in", dpi = 600, width = 7, height = 5)
```

## Contam identification

```
## identify potential contaminants with decontam using raw counts
kraken.ps <- phyloseq(otu_table(as.matrix(kraken.counts), taxa_are_rows = TRUE),
                      sample_data(meta))
# v1 using abundance
contam.freq <- isContaminant(kraken.ps, method = "frequency", conc = "DNA.ng.ul")
table(contam.freq$contaminant)
```

```
## 
## FALSE  TRUE 
##  6123    36
```

```
contams.freq <- rownames(contam.freq[which(contam.freq$contaminant),])

# v2 using blanks (exclude bk06)
contam.prev <- isContaminant(subset_samples(kraken.ps, SampleID != "bk06"), method="prevalence", neg="Blank")
table(contam.prev$contaminant)
```

```
## 
## FALSE  TRUE 
##  6025   134
```

```
contams.prev <- rownames(contam.prev[which(contam.prev$contaminant),])

keep.taxa.ids <- as.character(filt.taxa[which(!filt.taxa$taxon %in% c(contams.prev, contams.freq, "9606")),"taxon"])

contam.sums <- data.frame(
  SampleID=names(kraken.counts),
  contams.freq=colSums(prop.table(as.matrix(kraken.counts), margin = 2)[contams.freq,]),
  contams.prev=colSums(prop.table(as.matrix(kraken.counts), margin = 2)[contams.prev,]),
  human=prop.table(as.matrix(kraken.counts), margin = 2)["9606",],
  kept.taxa=colSums(prop.table(as.matrix(kraken.counts), margin = 2)[keep.taxa.ids,])
)
contam.sums <- merge(contam.sums, meta, by = "SampleID")
# see next chunk for plot 

# remove from ps
clean.ps <- prune_taxa(filt.ps, taxa = keep.taxa.ids)
sample_data(clean.ps)$Bracken.readct.clean <- sample_sums(clean.ps)
sample_data(clean.ps)$Bracken.richness.clean <- specnumber(otu_table(clean.ps))
sample_data(clean.ps)$Bracken.shannon.clean <- hillR::hill_taxa(otu_table(clean.ps), q = 1)

# write.table(counts.filt, "kraken2_otu_table_sticklebacks_GS_201207_filt.txt",
#             sep = "\t", row.names = T, quote = F)
```

## Final filtering

Filtering taxa based on:

- relative abundance > 0.05%
- `decontam` frequency: 36 contaminating taxa
  identified
- `decontam` prevalence (5 blanks - excluding bk06 due to
  probable low-level sample cross-contam): 134 taxa
- no filtering by absolute microbial read count as all samples
  >> blanks

```
ggplot(contam.sums, aes(Population2, kept.taxa, color = Population2))+
  geom_boxplot(outlier.colour = NA)+
  geom_point(size = 3, position = position_jitterdodge())+
  labs(title = "Taxa passing QC", x = "Population", y = "Relative abundance")+
  theme_classic()+
  theme(axis.text = element_text(size = 10, colour = "black"), axis.title = element_text(size = 11),
        legend.position = "none")
```

```
# looks reasonable - most losses come from excluding human DNA

ggplot(sample_data(subset_samples(clean.ps, Population2 != "blank")), aes(Population2, Bracken.readct.clean))+
  geom_boxplot(outlier.colour = NA)+
  geom_point(aes(color = Population2), size = 2, position = position_jitterdodge())+
  scale_y_continuous(trans = "log10")+
  labs(title = "Microbial reads", y = "No. of reads")+
  geom_hline(yintercept = 2000, linetype = 2)+
  theme_classic()+
  theme(axis.text = element_text(colour = "black"),
        legend.position = "none")
```

```
print("Summary: Microbial reads (no blanks)")
```

```
## [1] "Summary: Microbial reads (no blanks)"
```

```
summary(sample_data(subset_samples(clean.ps, Population2 != "blank"))$Bracken.readct.clean)
```

```
##    Min. 1st Qu.  Median    Mean 3rd Qu.    Max. 
##    1664    2220    2474    3999    3338   51211
```

```
print("Summary: Microbial reads (blanks)")
```

```
## [1] "Summary: Microbial reads (blanks)"
```

```
summary(sample_data(subset_samples(clean.ps, Population2 == "blank"))$Bracken.readct.clean)
```

```
##    Min. 1st Qu.  Median    Mean 3rd Qu.    Max. 
##    46.0   140.8   176.0   198.5   290.0   336.0
```

```
# doesn't make sense to filter by read count as all samples well above blanks
```

# Dataset summaries

```
ggplot(meta, aes(length_cm, weight_g, color = Population2))+
  geom_point(size = 3)+
  labs(x = "length of fish (cm)", y = "weight of fish (g)")+
  theme_classic()+
  theme(axis.text = element_text(color = "black", size = 12), legend.position = "right", plot.title = element_text(size = 13),
        axis.title = element_text(size = 12), strip.text.x = element_text(size = 12),
        panel.grid.major.y = element_line(colour = "grey70", linetype = 2, size = 1.1), panel.ontop = FALSE)
```

```
ggplot(meta, aes(Population2, weight_g))+
  geom_violin(aes(fill = Population2), alpha = 0.25)+
  geom_point(aes(fill = Population2), shape = 21, color = "black", size = 4, 
             position = position_jitterdodge())+
  labs(x = "Population", y = "weight of fish (g)")+
  theme_classic()+
  theme(axis.text = element_text(color = "black", size = 12), legend.position = "none", plot.title = element_text(size = 13),
        axis.title = element_text(size = 12), strip.text.x = element_text(size = 12),
        panel.grid.major.y = element_line(colour = "grey70", linetype = 2, size = 1.1), panel.ontop = FALSE)
```

```
ggplot(meta, aes(Population2, length_cm))+
  geom_violin(aes(fill = Population2), alpha = 0.25)+
  geom_point(aes(fill = Population2), shape = 21, color = "black", size = 4, 
             position = position_jitterdodge())+
  labs(x = "Population", y = "length of fish (cm)")+
  theme_classic()+
  theme(axis.text = element_text(color = "black", size = 12), legend.position = "none", plot.title = element_text(size = 13),
        axis.title = element_text(size = 12), strip.text.x = element_text(size = 12),
        panel.grid.major.y = element_line(colour = "grey70", linetype = 2, size = 1.1), panel.ontop = FALSE)
```

# Community analyses

Perform majority of analyses at the genus-level, due to uncertainty
in the assignment of species.

```
# clean.ps.relab <- transform_sample_counts(clean.ps, function(OTU) OTU/sum(OTU))

clean.df.genus <- rank_summary(clean.ps, "g")
clean.df.genus$SampleID <- row.names(clean.df.genus)
clean.df.genus <- clean.df.genus[,c(ncol(clean.df.genus),1:(ncol(clean.df.genus)-2))]
clean.df.genus$Unassigned <- rowSums(clean.df.genus[,grep("Unknown",names(clean.df.genus))])
clean.df.genus <- clean.df.genus[,grep("Unknown", names(clean.df.genus), invert = T)]

clean.ps.genus <- phyloseq(otu_table(clean.df.genus[,2:ncol(clean.df.genus)],
                                     taxa_are_rows = FALSE),
                           sample_data(clean.ps))
clean.ps.genus.relab <- transform_sample_counts(clean.ps.genus, function(OTU) OTU/sum(OTU))
 
clean.ps.genus.clr <- transform_sample_counts(clean.ps.genus, function(OTU) OTU+1) #pseudo-count
clean.ps.genus.clr <- microbiome::transform(clean.ps.genus.clr, 'clr')
```

## Top taxa summary

### Figure 4

```
# no blanks
clean.df.genus.top <- top_taxa(clean.df.genus[grep("^st",clean.df.genus$SampleID),], sample_var = "SampleID", top = 20)
clean.df.genus.top <- melt(clean.df.genus.top, id.vars = "SampleID", value.name = "count", variable.name = "taxon")
clean.df.genus.top <- merge(clean.df.genus.top, meta, by = "SampleID", all.x = T, all.y = F)

ggplot(clean.df.genus.top, aes(SampleID, count, fill=taxon))+
  geom_col(position="fill", width = 1)+
  labs(x="Sample", y="Relative abundance", fill="Genus")+
  scale_fill_manual(values=c("#F8766D", "#002255ff", "#abc837ff", "#ffe680ff", "#ffcc00ff", "#e38200ff", "#d40055ff", 
            "purple3", "#a02c2cff", "#de87cdff", "mediumseagreen", "#aa0088ff", "#00d455ff", "#00ccffff", 
            "#d42affff", "darkgreen", "#0044aaff",  "#2a7fffff", "#aaccffff", "#73b3c2ff","#969696","#000000"))+
  theme(panel.grid.major = element_blank(), panel.grid.minor = element_blank(), panel.background = element_blank(), 
        axis.line = element_line(colour = "black"),
        axis.title = element_text(size = 13), axis.text = element_text(color = "black", size = 12),
        axis.text.x = element_text(angle = 90),
        legend.position = "right")+
  facet_wrap(~Population2, scales = "free_x", nrow = 1,
             labeller=labeller(Population2 = c(Galta="Galtaból (NP)", Galta_P="Galtaból (P)", Pristi="Þristikla (NP)")))
```

```
ggsave("figures_2024/Figure_4_microbiome_composition_by_individual.png",units = "in", dpi = 800, width = 12, height = 5)
```

## Alpha diversity

```
sample_data(clean.ps.genus.relab)$Alpha.hill.q0 <- hillR::hill_taxa(otu_table(clean.ps.genus.relab), q = 0)
sample_data(clean.ps.genus.relab)$Alpha.hill.q1 <- hillR::hill_taxa(otu_table(clean.ps.genus.relab), q = 1)
```

```
plot.alpha.1a <-
ggplot(sample_data(subset_samples(clean.ps.genus.relab, Population2 %in% c("Galta","Pristi"))), aes(Population2, Alpha.hill.q0))+
  geom_violin(aes(fill = Population2), alpha = 0.25, draw_quantiles = c(0.25, 0.5, 0.75))+
  geom_point(aes(fill = Population2), shape = 21, color = "black", size = 3, 
             position = position_jitterdodge())+
  scale_fill_manual(values = c("#7CAE00", "#C77CFF"))+
  scale_x_discrete(labels = c("Galtaból (NP)","Þristikla (NP)"))+
  labs(x = "", y = "Richness (Hill's estimate)", title = "Non-parasitized fish")+
  geom_signif(comparisons = list(c("Galta","Pristi")), test = "wilcox.test",
                        step_increase = c(1.05))+
  theme_minimal()+
  theme(axis.text = element_text(color = "black", size = 12), legend.position = "none", plot.title = element_text(size = 13, hjust = 0.5),
        axis.title = element_text(size = 12))

plot.alpha.1b <-
ggplot(sample_data(subset_samples(clean.ps.genus.relab, Population2 %in% c("Galta","Pristi"))), aes(Population2, Alpha.hill.q1))+
  geom_violin(aes(fill = Population2), alpha = 0.25, draw_quantiles = c(0.25, 0.5, 0.75))+
  geom_point(aes(fill = Population2), shape = 21, color = "black", size = 3, 
             position = position_jitterdodge())+
  scale_fill_manual(values = c("#7CAE00", "#C77CFF"))+
  scale_x_discrete(labels = c("Galtaból (NP)","Þristikla (NP)"))+
  labs(x = "", y = "Shannon index (Hill's estimate)", title = "Non-parasitized fish")+
  geom_signif(comparisons = list(c("Galta","Pristi")), test = "wilcox.test",
                        step_increase = c(1.05))+
  theme_minimal()+
  theme(axis.text = element_text(color = "black", size = 12), legend.position = "none", plot.title = element_text(size = 13, hjust = 0.5),
        axis.title = element_text(size = 12))


plot.alpha.2a <-
ggplot(sample_data(subset_samples(clean.ps.genus.relab, Population2 %in% c("Galta","Galta_P"))), aes(Population2, Alpha.hill.q0))+
  geom_violin(aes(fill = Population2), alpha = 0.25, draw_quantiles = c(0.25, 0.5, 0.75))+
  geom_point(aes(fill = Population2), shape = 21, color = "black", size = 3, 
             position = position_jitterdodge())+
  scale_fill_manual(values = c("#7CAE00", "#00BFC4"))+
  scale_x_discrete(labels = c("Galtaból (NP)","Galtaból (P)"))+
  labs(x = "", y = "Richness (Hill's estimate)", title = "Galtaból population")+
  geom_signif(comparisons = list(c("Galta","Galta_P")), test = "wilcox.test",
                        step_increase = c(1.05))+
  theme_minimal()+
  theme(axis.text = element_text(color = "black", size = 12), legend.position = "none", plot.title = element_text(size = 13, hjust = 0.5),
        axis.title = element_text(size = 12))

plot.alpha.2b <-
ggplot(sample_data(subset_samples(clean.ps.genus.relab, Population2 %in% c("Galta","Galta_P"))), aes(Population2, Alpha.hill.q1))+
  geom_violin(aes(fill = Population2), alpha = 0.25, draw_quantiles = c(0.25, 0.5, 0.75))+
  geom_point(aes(fill = Population2), shape = 21, color = "black", size = 3, 
             position = position_jitterdodge())+
  scale_fill_manual(values = c("#7CAE00", "#00BFC4"))+
  scale_x_discrete(labels = c("Galtaból (NP)","Galtaból (P)"))+
  labs(x = "", y = "Shannon index (Hill's estimate)", title = "Galtaból population")+
  geom_signif(comparisons = list(c("Galta","Galta_P")), test = "wilcox.test",
                        step_increase = c(1.05))+
  theme_minimal()+
  theme(axis.text = element_text(color = "black", size = 12), legend.position = "none", plot.title = element_text(size = 13, hjust = 0.5),
        axis.title = element_text(size = 12))
```

```
plot.microbiome.1a <-
cowplot::plot_grid(plot.alpha.1a, plot.alpha.1b, plot.alpha.2a, plot.alpha.2b,
                   align = "hv", axis = "tblr", nrow = 1,
                   labels = c("A","B","C","D"))
```

```
## Warning in wilcox.test.default(c(220, 174, 171, 181, 182, 171, 178, 203, :
## cannot compute exact p-value with ties
```

```
## Warning in wilcox.test.default(c(220, 174, 171, 181, 182, 171, 178, 203, :
## cannot compute exact p-value with ties
```

```
plot.microbiome.1a
```

```
ggplot(sample_data(subset_samples(clean.ps.genus.relab, Population2 %in% c("Galta_P","Galta","Pristi"))), aes(Type, Alpha.hill.q0))+
  geom_violin(aes(fill = Type), alpha = 0.25, draw_quantiles = c(0.25, 0.5, 0.75))+
  geom_point(aes(fill = Type), shape = 21, color = "black", size = 3, 
             position = position_jitterdodge(jitter.width = 0.25))+
  scale_fill_manual(values = c("grey30", "grey70"))+
  scale_x_discrete(labels = c("Non-\nexposed","Predator-\nexposed"))+
  labs(x = "Treatment", y = "Richness (Hill's estimate)")+
  geom_signif(comparisons = list(c("Control","Predator")), test = "wilcox.test",
                        step_increase = c(1.05))+
  theme_minimal()+
  theme(axis.text = element_text(color = "black", size = 12), legend.position = "none", plot.title = element_text(size = 13, hjust = 0.5),
        axis.title = element_text(size = 12),
        strip.text = element_text(color = "black", size = 13))+
  facet_wrap(~ Population2, nrow = 1, scales = "free_x",
             labeller=labeller(Population2 = c(Galta="Galtaból (NP)", Galta_P="Galtaból (P)", Pristi="Þristikla (NP)")))
```

```
ggplot(sample_data(subset_samples(clean.ps.genus.relab, Population2 %in% c("Galta_P","Galta","Pristi"))), aes(Type, Alpha.hill.q1))+
  geom_violin(aes(fill = Type), alpha = 0.25, draw_quantiles = c(0.25, 0.5, 0.75))+
  geom_point(aes(fill = Type), shape = 21, color = "black", size = 3, 
             position = position_jitterdodge(jitter.width = 0.25))+
  scale_fill_manual(values = c("grey30", "grey70"))+
  scale_x_discrete(labels = c("Non-\nexposed","Predator-\nexposed"))+
  labs(x = "Treatment", y = "Shannon index (Hill's estimate)")+
  geom_signif(comparisons = list(c("Control","Predator")), test = "wilcox.test",
                        step_increase = c(1.05))+
  theme_minimal()+
  theme(axis.text = element_text(color = "black", size = 12), legend.position = "none", plot.title = element_text(size = 13, hjust = 0.5),
        axis.title = element_text(size = 12),
        strip.text = element_text(color = "black", size = 13))+
  facet_wrap(~ Population2, nrow = 1, scales = "free_x",
             labeller=labeller(Population2 = c(Galta="Galtaból (NP)", Galta_P="Galtaból (P)", Pristi="Þristikla (NP)")))
```

## Ordination

PCoA on CLR-normalised abundances, with euclidean distances.

All samples (including blanks):

```
ord.all.g.clr <- ps_ordination_f(clean.ps.genus.clr, "PCoA", "euclidean", k.range = NA, "ordinations/PCoA_euc_all_clr_genus.rds")
plot_ordination(clean.ps.genus.clr, ord.all.g.clr, type="samples", axes=c(1,2), color="Population2")+
  geom_vline(xintercept = 0, linetype = 3, size = 1, color = "grey42")+
  geom_hline(yintercept = 0, linetype = 3, size = 1, color = "grey42")+
  geom_point(size=4)+
#  geom_text(aes(label = SampleID), color="black")+
  labs(title = "Genus-level CLR: Fish + blanks")+
  theme_bw()+
  theme(panel.grid = element_blank(), legend.position = "right",
        axis.text = element_text(size = 12, colour = "black"), axis.title = element_text(size = 13),
        panel.border = element_rect(size = 1.2))
```

Galta vs Pristi (non-parasitized):

```
# NP
# no blanks
print("Non-parasitized fish - genus-level")
```

```
## [1] "Non-parasitized fish - genus-level"
```

```
dist.NP.fish.g.clr <- phyloseq::distance(subset_samples(clean.ps.genus.clr, Population2 %in% c("Galta","Pristi")),
                                 method = "euclidean")
meta.NP.fish.g.clr <- data.frame(sample_data(subset_samples(clean.ps.genus.clr, Population2 %in% c("Galta","Pristi"))))

perm.NP.fish.g.clr <- adonis2(dist.NP.fish.g.clr ~ Bracken.readct.total + Population + Type + length_cm + Ext.date, data = meta.NP.fish.g.clr,
                              method = "euclidean", permutations = 1000)

# format to print as table
perm.NP.fish.g.clr.df <- as.data.frame(perm.NP.fish.g.clr)
perm.NP.fish.g.clr.df$Variable <- row.names(perm.NP.fish.g.clr.df)
perm.NP.fish.g.clr.df$Test <- "NP.fish.genus.CLR"
row.names(perm.NP.fish.g.clr.df) <- NULL

ord.NP.fish.g.clr <- ps_ordination_f(subset_samples(clean.ps.genus.clr, Population2 %in% c("Galta","Pristi")), "PCoA", "euclidean", k.range = NA, "ordinations/PCoA_euc_fish_NP_clr_genus.rds")

plot.ord.1 <-
plot_ordination(clean.ps.genus.clr, ord.NP.fish.g.clr, type="samples", axes=c(1,2), color="Population2", shape="Type")+
  geom_vline(xintercept = 0, linetype = 3, size = 1, color = "grey42")+
  geom_hline(yintercept = 0, linetype = 3, size = 1, color = "grey42")+
  geom_point(size=4)+
  #  geom_text(aes(label = SampleID), color="black")+
  scale_color_manual(values = gg_color_hue(4)[c(2,4)], 
                     labels = c("Galtaból (NP)","Þristikla (NP)"))+
  scale_shape_manual(values = c(16,17), 
                     labels = c("Non-exposed","Predator-exposed"))+
  # labs(title = "Genus-level CLR: Fish (non-parasitized)")+
  labs(title = "Non-parasitized fish", color = "Population", shape = "Treatment")+
  theme_bw()+
  theme(panel.grid = element_blank(), legend.position = "right",
        axis.text = element_text(size = 12, colour = "black"), axis.title = element_text(size = 13),
        panel.border = element_rect(size = 1.2), plot.title = element_text(hjust = 0.5))
```

```
# Galta P vs NP
# no blanks
print("Galta fish - genus-level")
```

```
## [1] "Galta fish - genus-level"
```

```
dist.GP.fish.g.clr <- phyloseq::distance(subset_samples(clean.ps.genus.clr, Population2 %in% c("Galta","Galta_P")),
                                 method = "euclidean")
meta.GP.fish.g.clr <- data.frame(sample_data(subset_samples(clean.ps.genus.clr, Population2 %in% c("Galta","Galta_P"))))

perm.GP.fish.g.clr <- adonis2(dist.GP.fish.g.clr ~ Bracken.readct.total + Parasite + Type + length_cm + Ext.date, data = meta.GP.fish.g.clr,
                              method = "euclidean", permutations = 1000)

# format to print as table
perm.GP.fish.g.clr.df <- as.data.frame(perm.GP.fish.g.clr)
perm.GP.fish.g.clr.df$Variable <- row.names(perm.GP.fish.g.clr.df)
perm.GP.fish.g.clr.df$Test <- "Galta.fish.genus.CLR"
row.names(perm.GP.fish.g.clr.df) <- NULL

ord.GP.fish.g.clr <- ps_ordination_f(subset_samples(clean.ps.genus.clr, Population2 %in% c("Galta","Galta_P")), "PCoA", "euclidean", k.range = NA, "ordinations/PCoA_euc_fish_GP_clr_genus.rds")

plot.ord.2 <-
plot_ordination(clean.ps.genus.clr, ord.GP.fish.g.clr, type="samples", axes=c(1,2), color="Population2", shape="Type")+
  geom_vline(xintercept = 0, linetype = 3, size = 1, color = "grey42")+
  geom_hline(yintercept = 0, linetype = 3, size = 1, color = "grey42")+
  geom_point(size=4)+
  #  geom_text(aes(label = SampleID), color="black")+
  scale_color_manual(values = gg_color_hue(4)[c(2:3)], 
                     labels = c("Non-parasitized","Parasitized"))+
  scale_shape_manual(values = c(16,17), 
                     labels = c("Non-exposed","Predator-exposed"))+
  # labs(title = "Genus-level CLR: Fish (Galta population)")+
  labs(title = "Galtaból population", color = "Parasitized", shape = "Treatment")+
  theme_bw()+
  theme(panel.grid = element_blank(), legend.position = "right",
        axis.text = element_text(size = 12, colour = "black"), axis.title = element_text(size = 13),
        panel.border = element_rect(size = 1.2), plot.title = element_text(hjust = 0.5))
```

```
cowplot::plot_grid(plot.ord.1, plot.ord.2,
                   align = "hv", axis = "tblr", nrow = 1,
                   labels = c("A","B"))
```

```
ggsave("figures_2024/Supp_figure_S3_ordination_by_population.png",units = "in", dpi = 600, width = 11, height = 4.5)
```

### Limma for batch effects

```
limma.design.NP <- model.matrix(~ Population + Type, data = meta.NP.fish.g.clr)

NP.nobatch <- limma::removeBatchEffect(as.data.frame(t(otu_table(subset_samples(clean.ps.genus.clr, Population2 %in% c("Galta","Pristi"))))), 
                                         batch = meta.NP.fish.g.clr$Ext.date,
                                         design = limma.design.NP)

NP.nobatch.ps <- phyloseq(
  otu_table(NP.nobatch, taxa_are_rows = T),
  sample_data(meta.NP.fish.g.clr)
)

nobatch.dist.NP.fish.g.clr <- phyloseq::distance(NP.nobatch.ps,
                                 method = "euclidean")

nobatch.perm.NP.fish.g.clr <- adonis2(nobatch.dist.NP.fish.g.clr ~ Bracken.readct.total + Population + Type + length_cm + Ext.date, data = meta.NP.fish.g.clr,
                              method = "euclidean", permutations = 1000)

# # format to print as table
nobatch.perm.NP.fish.g.clr.df <- as.data.frame(nobatch.perm.NP.fish.g.clr)
nobatch.perm.NP.fish.g.clr.df$Variable <- row.names(nobatch.perm.NP.fish.g.clr.df)
nobatch.perm.NP.fish.g.clr.df$Test <- "NP.fish.genus.CLR.nobatch"
row.names(nobatch.perm.NP.fish.g.clr.df) <- NULL

nobatch.ord.NP.fish.g.clr <- ps_ordination_f(NP.nobatch.ps, "PCoA", "euclidean", k.range = NA, "ordinations/PCoA_euc_fish_NP_clr_genus_nobatch.rds")

plot.ord.3 <-
plot_ordination(NP.nobatch.ps, nobatch.ord.NP.fish.g.clr, type="samples", axes=c(1,2), color="Population2", shape="Type")+
  geom_vline(xintercept = 0, linetype = 3, size = 1, color = "grey42")+
  geom_hline(yintercept = 0, linetype = 3, size = 1, color = "grey42")+
  geom_point(size=4)+
  #  geom_text(aes(label = SampleID), color="black")+
  scale_color_manual(values = gg_color_hue(4)[c(2,4)], 
                     labels = c("Galtaból (NP)","Þristikla (NP)"))+
  scale_shape_manual(values = c(16,17), 
                     labels = c("Non-exposed","Predator-exposed"))+
  # labs(title = "Genus-level CLR: Fish (non-parasitized)")+
  labs(title = "Non-parasitized fish", color = "Population", shape = "Treatment")+
  theme_bw()+
  theme(panel.grid = element_blank(), legend.position = "bottom",
        axis.text = element_text(size = 12, colour = "black"), axis.title = element_text(size = 13),
        panel.border = element_rect(size = 1.2), plot.title = element_text(hjust = 0.5))+
  guides(color = guide_legend(nrow = 2, byrow = TRUE, direction = "vertical"),
         shape = guide_legend(nrow = 2, byrow = TRUE, direction = "vertical"))
```

```
limma.design.GP <- model.matrix(~ Parasite + Type, data = meta.GP.fish.g.clr)

GP.nobatch <- limma::removeBatchEffect(as.data.frame(t(otu_table(subset_samples(clean.ps.genus.clr, Population2 %in% c("Galta","Galta_P"))))), 
                                         batch = meta.GP.fish.g.clr$Ext.date,
                                         design = limma.design.GP)

GP.nobatch.ps <- phyloseq(
  otu_table(GP.nobatch, taxa_are_rows = T),
  sample_data(meta.GP.fish.g.clr)
)

nobatch.dist.GP.fish.g.clr <- phyloseq::distance(GP.nobatch.ps,
                                 method = "euclidean")

nobatch.perm.GP.fish.g.clr <- adonis2(nobatch.dist.GP.fish.g.clr ~ Bracken.readct.total + Parasite + Type + length_cm + Ext.date, data = meta.GP.fish.g.clr,
                              method = "euclidean", permutations = 1000)

# # format to print as table
nobatch.perm.GP.fish.g.clr.df <- as.data.frame(nobatch.perm.GP.fish.g.clr)
nobatch.perm.GP.fish.g.clr.df$Variable <- row.names(nobatch.perm.GP.fish.g.clr.df)
nobatch.perm.GP.fish.g.clr.df$Test <- "GP.fish.genus.CLR.nobatch"
row.names(nobatch.perm.GP.fish.g.clr.df) <- NULL

nobatch.ord.GP.fish.g.clr <- ps_ordination_f(GP.nobatch.ps, "PCoA", "euclidean", k.range = NA, "ordinations/PCoA_euc_fish_GP_clr_genus_nobatch.rds")

plot.ord.4 <-
plot_ordination(GP.nobatch.ps, nobatch.ord.GP.fish.g.clr, type="samples", axes=c(1,2), color="Population2", shape="Type")+
  geom_vline(xintercept = 0, linetype = 3, size = 1, color = "grey42")+
  geom_hline(yintercept = 0, linetype = 3, size = 1, color = "grey42")+
  geom_point(size=4)+
  #  geom_text(aes(label = SampleID), color="black")+
  scale_color_manual(values = gg_color_hue(4)[c(2:3)], 
                     labels = c("Non-parasitized","Parasitized"))+
  scale_shape_manual(values = c(16,17), 
                     labels = c("Non-exposed","Predator-exposed"))+
  # labs(title = "Genus-level CLR: Fish (Galta population)")+
  labs(title = "Galtaból population", color = "Parasitized", shape = "Treatment")+
  theme_bw()+
  theme(panel.grid = element_blank(), legend.position = "bottom",
        axis.text = element_text(size = 12, colour = "black"), axis.title = element_text(size = 13),
        panel.border = element_rect(size = 1.2), plot.title = element_text(hjust = 0.5))+
  guides(color = guide_legend(nrow = 2, byrow = TRUE, direction = "vertical"),
         shape = guide_legend(nrow = 2, byrow = TRUE, direction = "vertical"))
```

```
plot.microbiome.1b <-
cowplot::plot_grid(plot.ord.3, plot.ord.4,
                   align = "hv", axis = "tblr", nrow = 1,
                   labels = c("E","F"))
```

```
### write permanovas into one table
if(!file.exists("figures_2024/Supp_table_S3_permanovas.csv")){
  perm.all <- rbind.data.frame(
    perm.NP.fish.g.clr.df,
    perm.GP.fish.g.clr.df,
    nobatch.perm.NP.fish.g.clr.df,
    nobatch.perm.GP.fish.g.clr.df
  )
  write.csv(perm.all[,c(7,6,1:5)],
            "figures_2024/Supp_table_S3_permanovas.csv",
            row.names = F, quote = F)
  rm(perm.all)
}
```

### Figure 5

```
cowplot::plot_grid(plot.microbiome.1a, plot.microbiome.1b,
                   align = "hv", axis = "lr", nrow = 2,
                   rel_heights = c(0.75,1))
```

```
ggsave("figures_2024/Figure_5_microbiome_diversity_by_population_nobatch_2.png",units = "in", dpi = 800, width = 12, height = 10, bg = "white")
```

# Behaviour data

Needed for behaviour-microbiome integration (MOFA, see below) and to
generate behavioral plots in the same style as the microbiome plots.
Note that only a subset of individuals had DNA extracted for microbiome
analysis, so there are more individuals in the behavioral dataset than
the microbiome dataset.

```
meta.beh <- read.csv("behaviour/Before-After-control-pred-211122.csv")
meta.beh$SampleID <- sapply(meta.beh$Video_nbb, function(x){
  if(x %in% meta$Video_nb){
    meta[which(meta$Video_nb == x),"SampleID"]
  } else {
    NA
  }
})
meta.beh$Population2 <- as.character(meta.beh$Population)
meta.beh[meta.beh$Parasite == "P","Population2"] <- "Galta_P"
meta.beh$Population3 <- interaction(meta.beh$Population, meta.beh$Type)
meta.beh$Population4 <- interaction(meta.beh$Population, meta.beh$Period)

meta.beh$Period <- factor(meta.beh$Period, levels = c("BT","AT"), ordered = T)
meta.beh$Population4 <- factor(meta.beh$Population4, levels = c("Galta.BT","Galta.AT","Pristi.BT","Pristi.AT"))
meta.beh$Population3 <- factor(meta.beh$Population3, levels = c("Galta.Control", "Galta.Predator","Pristi.Control","Pristi.Predator"))
```

## Figure 2

```
# post-host contrasts for p-value brackets
fig.b1.sig <- read.delim("behaviour/posthoc_contrasts_table1.txt", sep = "\t")
fig.b1.sig$Population.Type1 <- paste(fig.b1.sig$Population1, fig.b1.sig$Type1, sep = ".")
fig.b1.sig$Population.Type2 <- paste(fig.b1.sig$Population2, fig.b1.sig$Type2, sep = ".")

# table(fig.b1.sig[,c("Variable","Significant")])
```

```
plot.beh.a <-
ggplot(subset(meta.beh, Period == "BT"), aes(Population3, Dtot))+
  geom_vline(xintercept = 2.5, linetype = 3, size = 0.75, colour = "black")+
  geom_violin(aes(fill = Population3), draw_quantiles = c(0.5), alpha = 0.25)+
  geom_point(aes(fill = Population3), shape = 21, color = "black", size = 4, 
             position = position_jitterdodge(jitter.width = 0.2))+
  labs(x = "Treatment and Population", y = "Total Traveled Distance (mm)")+
  scale_x_discrete(labels = c("NE\nGaltaból","PE\n",
                              "NE\nÞristikla","PE\n"))+
  scale_fill_manual(values = c("#7CAE00","#7CAE00","#C77CFF","#C77CFF"))+
  geom_signif(data = data.frame(
    subset(fig.b1.sig, Significant & Variable == "Dtot"),
    y = c(1525)),
                        aes(xmin = Population.Type1, xmax = Population.Type2, 
                            annotations = P.value, y_position = y),
                        textsize = 4, tip_length = 0.01,
                        manual = TRUE)+
  theme_classic()+
  theme(axis.text = element_text(color = "black", size = 12),
        axis.text.x = element_text(hjust = 0),
        legend.position = "none", 
        plot.title = element_text(size = 13),
        axis.title = element_text(size = 12), strip.text.x = element_text(size = 12))
```

```
## Warning in geom_signif(data = data.frame(subset(fig.b1.sig, Significant & :
## Ignoring unknown aesthetics: xmin, xmax, annotations, and y_position
```

```
plot.beh.b <-
ggplot(subset(meta.beh, Period == "BT"), aes(Population3, VelBL))+
  geom_vline(xintercept = 2.5, linetype = 3, size = 0.75, colour = "black")+
  geom_violin(aes(fill = Population3), draw_quantiles = c(0.5), alpha = 0.25)+
  geom_point(aes(fill = Population3), shape = 21, color = "black", size = 4, 
             position = position_jitterdodge(jitter.width = 0.2))+
  labs(x = "Treatment and Population", y = "Mean Velocity (BL s-1)")+
  scale_x_discrete(labels = c("NE\nGaltaból","PE\n",
                              "NE\nÞristikla","PE\n"))+
  scale_fill_manual(values = c("#7CAE00","#7CAE00","#C77CFF","#C77CFF"))+
  geom_signif(data = data.frame(
    subset(fig.b1.sig, Significant & Variable == "VelBL"),
    y = c(1.05)),
                        aes(xmin = Population.Type1, xmax = Population.Type2, 
                            annotations = P.value, y_position = y),
                        textsize = 4, tip_length = 0.01,
                        manual = TRUE)+
  theme_classic()+
  theme(axis.text = element_text(color = "black", size = 12),
        axis.text.x = element_text(hjust = 0),
        legend.position = "none", 
        plot.title = element_text(size = 13),
        axis.title = element_text(size = 12), strip.text.x = element_text(size = 12))
```

```
## Warning in geom_signif(data = data.frame(subset(fig.b1.sig, Significant & :
## Ignoring unknown aesthetics: xmin, xmax, annotations, and y_position
```

```
plot.beh.c <-
ggplot(subset(meta.beh, Period == "BT"), aes(Population3, Vang))+
  geom_vline(xintercept = 2.5, linetype = 3, size = 0.75, colour = "black")+
  geom_violin(aes(fill = Population3), draw_quantiles = c(0.5), alpha = 0.25)+
  geom_point(aes(fill = Population3), shape = 21, color = "black", size = 4, 
             position = position_jitterdodge(jitter.width = 0.2))+
  labs(x = "Treatment and Population", y = "Angular Velocity (\u00B0 s-1)")+
  scale_x_discrete(labels = c("NE\nGaltaból","PE\n",
                              "NE\nÞristikla","PE\n"))+
  scale_fill_manual(values = c("#7CAE00","#7CAE00","#C77CFF","#C77CFF"))+
  geom_signif(data = data.frame(
    subset(fig.b1.sig, Significant & Variable == "Vang"),
    y = c(890,850)),
                        aes(xmin = Population.Type1, xmax = Population.Type2, 
                            annotations = P.value, y_position = y),
                        textsize = 4, tip_length = 0.01,
                        manual = TRUE)+
  theme_classic()+
  theme(axis.text = element_text(color = "black", size = 12),
        axis.text.x = element_text(hjust = 0),
        legend.position = "none", 
        plot.title = element_text(size = 13),
        axis.title = element_text(size = 12), strip.text.x = element_text(size = 12))
```

```
## Warning in geom_signif(data = data.frame(subset(fig.b1.sig, Significant & :
## Ignoring unknown aesthetics: xmin, xmax, annotations, and y_position
```

```
plot.beh.d <-
ggplot(subset(meta.beh, Period == "BT"), aes(Population3, Center_Duration))+
  geom_vline(xintercept = 2.5, linetype = 3, size = 0.75, colour = "black")+
  geom_violin(aes(fill = Population3), draw_quantiles = c(0.5), alpha = 0.25)+
  geom_point(aes(fill = Population3), shape = 21, color = "black", size = 4, 
             position = position_jitterdodge(jitter.width = 0.2))+
  labs(x = "Treatment and Population", y = "Center Duration (s)")+
  scale_x_discrete(labels = c("NE\nGaltaból","PE\n",
                              "NE\nÞristikla","PE\n"))+
  scale_fill_manual(values = c("#7CAE00","#7CAE00","#C77CFF","#C77CFF"))+
  geom_signif(data = data.frame(
    subset(fig.b1.sig, Significant & Variable == "Center_duration"),
    y = c(285)),
                        aes(xmin = Population.Type1, xmax = Population.Type2, 
                            annotations = P.value, y_position = y),
                        textsize = 4, tip_length = 0.01,
                        manual = TRUE)+
  theme_classic()+
  theme(axis.text = element_text(color = "black", size = 12),
        axis.text.x = element_text(hjust = 0),
        legend.position = "none", 
        plot.title = element_text(size = 13),
        axis.title = element_text(size = 12), strip.text.x = element_text(size = 12))
```

```
## Warning in geom_signif(data = data.frame(subset(fig.b1.sig, Significant & :
## Ignoring unknown aesthetics: xmin, xmax, annotations, and y_position
```

```
cowplot::plot_grid(plot.beh.a, plot.beh.b, 
                   plot.beh.c, plot.beh.d,
                   align = "hv", axis = "tblr", ncol = 2,
                   labels = c("A","B","C","D"))
```

```
ggsave("figures_2024/Figure_2_behaviour_BT_by_population.png",units = "in", dpi = 800, width = 7, height = 9)
```

## Figure 3

```
meta.beh$Population4.Parasite <- interaction(meta.beh$Population4, meta.beh$Parasite)
meta.beh$Population4.Parasite <- factor(
  meta.beh$Population4.Parasite,
  levels = c("Galta.BT.NP", "Galta.AT.NP", "Pristi.BT.NP", "Pristi.AT.NP", 
             "Galta.BT.P", "Galta.AT.P", "Pristi.BT.P", "Pristi.AT.P")
)

# post-host contrasts for p-value brackets
fig.b2.sig <- read.delim("behaviour/posthoc_contrasts_table2.txt", sep = "\t",
                         header = T)

fig.b2.sig$Population.Period1 <- paste(fig.b2.sig$Population1, fig.b2.sig$Period1, sep = ".")
fig.b2.sig$Population.Period2 <- paste(fig.b2.sig$Population2, fig.b2.sig$Period2, sep = ".")

fig.b2.sig$Pop.Period.Para1 <- interaction(fig.b2.sig$Population.Period1,
                                           fig.b2.sig$Parasite1)
fig.b2.sig$Pop.Period.Para2 <- interaction(fig.b2.sig$Population.Period2,
                                           fig.b2.sig$Parasite2)
```

```
plot.beh.2.Dtot <-
ggplot(subset(meta.beh, Type == "Predator"), aes(Population4.Parasite, Dtot))+
  geom_vline(xintercept = 4.5, linetype = 3, size = 0.75, colour = "black")+
  annotate("text", x=2.5, y=2100, label= "Non-parasitized", fontface=2, size=4.5)+ 
  annotate("text", x=6.5, y=2100, label = "Parasitized", fontface=2, size=4.5)+
  geom_violin(aes(fill = Population4.Parasite), draw_quantiles = c(0.5), alpha = 0.25)+
  geom_point(aes(fill = Population4.Parasite), shape = 21, color = "black", size = 4, 
             position = position_jitterdodge(jitter.width = 0.2))+
  labs(x = "Period and Population", y = "Total Traveled Distance (mm)")+
  scale_x_discrete(labels = c("BT\nGaltaból","AT\n","BT\nÞristikla","AT\n",
                              "BT\nGaltaból","AT\n","BT\nÞristikla","AT\n"))+
  scale_fill_manual(values = c("#7CAE00","#7CAE00","#C77CFF","#C77CFF",
                               "#7CAE00","#7CAE00","#C77CFF","#C77CFF"))+
  theme_classic()+
  theme(axis.text = element_text(color = "black", size = 12),
        axis.text.x = element_text(hjust = 0),
        legend.position = "none", 
        plot.title = element_text(size = 13),
        axis.title = element_text(size = 12), strip.text.x = element_text(size = 12))

plot.beh.2.VelBL <-
ggplot(subset(meta.beh, Type == "Predator"), aes(Population4.Parasite, VelBL))+
  geom_vline(xintercept = 4.5, linetype = 3, size = 0.75, colour = "black")+
  annotate("text", x=2.5, y=1.3, label= "Non-parasitized", fontface=2, size=4.5)+ 
  annotate("text", x=6.5, y=1.3, label = "Parasitized", fontface=2, size=4.5)+
  geom_violin(aes(fill = Population4.Parasite), draw_quantiles = c(0.5), alpha = 0.25)+
  geom_point(aes(fill = Population4.Parasite), shape = 21, color = "black", size = 4, 
             position = position_jitterdodge(jitter.width = 0.2))+
  labs(x = "Period and Population", y = "Mean Velocity (BL s-1)")+
  scale_x_discrete(labels = c("BT\nGaltaból","AT\n","BT\nÞristikla","AT\n",
                              "BT\nGaltaból","AT\n","BT\nÞristikla","AT\n"))+
  scale_fill_manual(values = c("#7CAE00","#7CAE00","#C77CFF","#C77CFF",
                               "#7CAE00","#7CAE00","#C77CFF","#C77CFF"))+
  theme_classic()+
  theme(axis.text = element_text(color = "black", size = 12),
        axis.text.x = element_text(hjust = 0),
        legend.position = "none", 
        plot.title = element_text(size = 13),
        axis.title = element_text(size = 12), strip.text.x = element_text(size = 12))
```

```
plot.beh.2.Vang <-
ggplot(subset(meta.beh, Type == "Predator"), aes(Population4.Parasite, Vang))+
  geom_vline(xintercept = 4.5, linetype = 3, size = 0.75, colour = "black")+
  annotate("text", x=2.5, y=975, label= "Non-parasitized", fontface=2, size=4.5)+ 
  annotate("text", x=6.5, y=975, label = "Parasitized", fontface=2, size=4.5)+
  geom_violin(aes(fill = Population4.Parasite), draw_quantiles = c(0.5), alpha = 0.25)+
  geom_point(aes(fill = Population4.Parasite), shape = 21, color = "black", size = 4, 
             position = position_jitterdodge(jitter.width = 0.2))+
  labs(x = "Period and Population", y = "Angular Velocity (\u00B0 s-1)")+
  scale_x_discrete(labels = c("BT\nGaltaból","AT\n","BT\nÞristikla","AT\n",
                              "BT\nGaltaból","AT\n","BT\nÞristikla","AT\n"))+
  scale_fill_manual(values = c("#7CAE00","#7CAE00","#C77CFF","#C77CFF",
                               "#7CAE00","#7CAE00","#C77CFF","#C77CFF"))+
  geom_signif(data = data.frame(
    subset(fig.b2.sig, Significant & Variable == "Vang"),
    y=c(850,900,700)
  ),
              aes(xmin = Pop.Period.Para1, xmax = Pop.Period.Para2, 
                  annotations = P.value, y_position = y),
              textsize = 4, tip_length = 0.01,
              manual = TRUE)+
  theme_classic()+
  theme(axis.text = element_text(color = "black", size = 12),
        axis.text.x = element_text(hjust = 0),
        legend.position = "none", 
        plot.title = element_text(size = 13),
        axis.title = element_text(size = 12), strip.text.x = element_text(size = 12))
```

```
## Warning in geom_signif(data = data.frame(subset(fig.b2.sig, Significant & :
## Ignoring unknown aesthetics: xmin, xmax, annotations, and y_position
```

```
# add some spaces to <0.0001 label so it doesn't clash with the separation line
fig.b2.sig[which(fig.b2.sig$Variable == "Center_duration" & fig.b2.sig$Comparison == "Galta_AT_NP_vs_Galta_AT_P"),"P.value"] <- "<0.0001   "

plot.beh.2.Center <-
ggplot(subset(meta.beh, Type == "Predator"), aes(Population4.Parasite, Center_Duration))+
  geom_vline(xintercept = 4.5, linetype = 3, size = 0.75, colour = "black")+
  annotate("text", x=2.5, y=470, label= "Non-parasitized", fontface=2, size=4.5)+ 
  annotate("text", x=6.5, y=470, label = "Parasitized", fontface=2, size=4.5)+
  geom_violin(aes(fill = Population4.Parasite), draw_quantiles = c(0.5), alpha = 0.25)+
  geom_point(aes(fill = Population4.Parasite), shape = 21, color = "black", size = 4, 
             position = position_jitterdodge(jitter.width = 0.2))+
  labs(x = "Period and Population", y = "Center Duration (s)")+
  scale_x_discrete(labels = c("BT\nGaltaból","AT\n","BT\nÞristikla","AT\n",
                              "BT\nGaltaból","AT\n","BT\nÞristikla","AT\n"))+
  scale_fill_manual(values = c("#7CAE00","#7CAE00","#C77CFF","#C77CFF",
                               "#7CAE00","#7CAE00","#C77CFF","#C77CFF"))+
  geom_signif(data = data.frame(
    subset(fig.b2.sig, Significant & Variable == "Center_duration"),
    y=c(350,410)
  ),
              aes(xmin = Pop.Period.Para1, xmax = Pop.Period.Para2, 
                  annotations = P.value, y_position = y),
              textsize = 4, tip_length = 0.01, 
              manual = TRUE)+
  theme_classic()+
  theme(axis.text = element_text(color = "black", size = 12),
        axis.text.x = element_text(hjust = 0),
        legend.position = "none", 
        plot.title = element_text(size = 13),
        axis.title = element_text(size = 12), strip.text.x = element_text(size = 12))
```

```
## Warning in geom_signif(data = data.frame(subset(fig.b2.sig, Significant & :
## Ignoring unknown aesthetics: xmin, xmax, annotations, and y_position
```

```
cowplot::plot_grid(plot.beh.2.Dtot, 
                   plot.beh.2.VelBL,
                   plot.beh.2.Vang,
                   plot.beh.2.Center,
                   align = "hv", axis = "tblr", ncol = 2,
                   labels = c("A","B","C"))
```

```
ggsave("figures_2024/Figure_3_behaviour_Predator_by_Population_parasite.png",units = "in", dpi = 800, width = 10, height = 9)
```

# Association testing with Maaslin2

Variables to focus on:

- Population (non-parasitized Galta vs Pristi)
- Parasite (parasitized vs non-parasitized in Galta population)
- Treatment (“Type”, exposed to treatment or not, include in both
  tests)

```
# m1: population (no parasite)
maas.m1.pop.genus <- run_maaslin2(
  subset_samples(clean.ps.genus.relab, !Blank & Parasite != "P"),
  "maaslin2_out_211214/relab_genus_population", filt.taxa, 
  f_eff = c("Population","Type"), r_eff = c("Ext.date"),
  n = "none", t = "none", taxa_level = "g"
  
)
maas.m1.pop.genus$model <- "m1"

# m2: parasite (no Pristi)
maas.m2.para.genus <- run_maaslin2(
  subset_samples(clean.ps.genus.relab, !Blank & Population == "Galta"),
  "maaslin2_out_211214/relab_genus_parasite", filt.taxa, 
  f_eff = c("Parasite","Type"), r_eff = c("Ext.date"),
  n = "none", t = "none", taxa_level = "g"
  
)
maas.m2.para.genus$model <- "m2"

maas.all <- rbind.data.frame(maas.m1.pop.genus,maas.m2.para.genus)
```

**Significant results (FDR < 0.25)**

```
fdr.thresh <- 0.05
maas.all[which(maas.all$qval < fdr.thresh & maas.all$model %in% c("m1","m2")),c(11,1:9)] %>%
  kbl(row.names = FALSE) %>%
  kable_styling(bootstrap_options = c("striped","hover")) %>%
  column_spec(4, color = "black", 
              background = spec_color(maas.all[which(maas.all$qval < fdr.thresh & maas.all$model %in% c("m1","m2")),"coef"], 
                                      option = "viridis", scale_from = c(-0.05,0.05), alpha = 0.5))
```

| model | metadata | feature | value | coef | stderr | N | N.not.0 | pval | qval |
| --- | --- | --- | --- | --- | --- | --- | --- | --- | --- |
| m1 | Population | Pseudoalteromonas | Pristi | -0.0022146 | 0.0003778 | 49 | 36 | 0.0000005 | 0.0003217 |
| m2 | Parasite | Kitasatospora | P | -0.0233568 | 0.0053629 | 37 | 37 | 0.0001449 | 0.0487023 |
| m2 | Parasite | Shewanella | P | -0.0059858 | 0.0013938 | 37 | 37 | 0.0001381 | 0.0487023 |

```
# write significant results to file
write.table(maas.all[which(maas.all$qval < fdr.thresh & maas.all$model %in% c("m1","m2")),c(11,1:9)],
            "figures_2024/Supp_table_S4_maaslin_sig05_results.csv",
            row.names = F, quote = F, sep = ",")
```

# MOFA

Using MOFA to integrate behaviour and microbiome data. Since control
and predator-exposed fish contain different variables (distance to
predator, etc), need to run MOFA separately for both.

Behavourial variables are not normally distributed - use INT to
approximate a normal distribution. Use 100 most variable microbial
genera, after using limma to regress out extraction batch effects.

```
# Samples & metadata
samples.TypeC <- meta[which(meta$Type == "Control"),"SampleID"]
samples.TypeP <- meta[which(meta$Type == "Predator"),"SampleID"]

meta.typeC <- phyloseq::subset_samples(clean.ps.genus.clr, Type == "Control")
meta.typeC <- data.frame(meta.typeC@sam_data)
meta.typeC$sample <- meta.typeC$SampleID

meta.typeP <- phyloseq::subset_samples(clean.ps.genus.clr, Type == "Predator")
meta.typeP <- data.frame(meta.typeP@sam_data)
meta.typeP$sample <- meta.typeP$SampleID


# Behavioural variables
mofa.beh <- meta[which(!meta$Blank),grep("_[AB]T",names(meta), value = T)]
mofa.beh <- mofa.beh[,grep("Dim",names(mofa.beh), value = T, invert = T)]
mofa.beh <- mofa.beh[,grep("first_reaction",names(mofa.beh), value = T, invert = T)]
mofa.beh <- mofa.beh[,grep("X5sec_after",names(mofa.beh), value = T, invert = T)]
# 62 samples, 32 variables

qtrans <-
  function(x){
    k<-!is.na(x)
    ran<-rank(x[k])
    y<-qnorm((1:sum(k)-0.5)/sum(k))
    x[k]<-y[ran]
    x
  }

mofa.beh.int <- mapply(FUN = qtrans, mofa.beh, USE.NAMES = T)
rownames(mofa.beh.int) <- row.names(mofa.beh)

beh.variables.C <- colnames(mofa.beh.int[samples.TypeC,which(!is.na(colSums(mofa.beh.int[samples.TypeC,])))])
beh.variables.P <- colnames(mofa.beh.int[samples.TypeP,which(!is.na(colSums(mofa.beh.int[samples.TypeP,])))])
beh.variables.P <- c(beh.variables.P,"Latency.time_AT")

# limma on microbiome in controls
limma.design.typeC <- model.matrix(~ Population + Parasite, data = data.frame(sample_data(subset_samples(clean.ps.genus.clr, Type == "Control"))))

typeC.nobatch <- limma::removeBatchEffect(
  as.data.frame(t(otu_table(subset_samples(clean.ps.genus.clr, Type == "Control")))), 
  batch = sample_data(subset_samples(clean.ps.genus.clr, Type == "Control"))$Ext.date,
  design = limma.design.typeC)

micro.var.genes.C.nobatch <- rownames(typeC.nobatch)[order(apply(typeC.nobatch, 1, var),
                                                           decreasing = T)][1:100]


# limma on microbiome in predator-exposed
limma.design.typeP <- model.matrix(~ Population + Parasite, data = data.frame(sample_data(subset_samples(clean.ps.genus.clr, Type == "Predator"))))

typeP.nobatch <- limma::removeBatchEffect(
  as.data.frame(t(otu_table(subset_samples(clean.ps.genus.clr, Type == "Predator")))), 
  batch = sample_data(subset_samples(clean.ps.genus.clr, Type == "Predator"))$Ext.date,
  design = limma.design.typeP)

micro.var.genes.P.nobatch <- rownames(typeP.nobatch)[order(apply(typeP.nobatch, 1, var),
                                                           decreasing = T)][1:100]
# MOFA setups
nobatch.mofa.input.C <- rbind.data.frame(
  cbind.data.frame(
    melt(as.matrix(mofa.beh.int[samples.TypeC,beh.variables.C]),
         varnames = c("sample","feature")),
    data.frame(view = "Behaviour")),
  cbind.data.frame(
    melt(t(typeC.nobatch[micro.var.genes.C.nobatch,]),
         varnames = c("sample","feature")),
    data.frame(view = "Microbiome"))
)

nobatch.mofa.input.P <- rbind.data.frame(
  cbind.data.frame(
    melt(as.matrix(mofa.beh.int[samples.TypeP,beh.variables.P]),
         varnames = c("sample","feature")),
    data.frame(view = "Behaviour")),
  cbind.data.frame(
    melt(t(typeP.nobatch[micro.var.genes.P.nobatch,]),
         varnames = c("sample","feature")),
    data.frame(view = "Microbiome"))
)

# belatedly realised that Vel is just the raw form of Vel_BL (velocity normalised by body length) and should not be included
# also, Border & Center duration are inverse measures of the same parameter, thus should only use one (we are going with Center Duration)
nobatch.mofa.input.C <- nobatch.mofa.input.C[which(!nobatch.mofa.input.C$feature %in% c("Vel_AT","Vel_BT","Border_duration_BT","Border_duration_AT")),]
nobatch.mofa.input.P <- nobatch.mofa.input.P[which(!nobatch.mofa.input.P$feature %in% c("Vel_AT","Vel_BT","Border_duration_BT","Border_duration_AT")),]

# write.csv(nobatch.mofa.input.C, "mofa_runs_20250211/mofa_abundance_input_controls_250211.csv", row.names = F, quote = F)
# write.csv(nobatch.mofa.input.P, "mofa_runs_20250211/mofa_abundance_input_predator_250211.csv", row.names = F, quote = F)
# write.csv(meta.typeC, "mofa_runs_20250211/mofa_meta_typeC_250211.csv", row.names = F, quote = F)
# write.csv(meta.typeP, "mofa_runs_20250211/mofa_meta_typeP_250211.csv", row.names = F, quote = F)
```

## controls

```
## note MOFA clashes with phyloseq, should be run in an almost-empty environment
# library(MOFA2)
# library(reshape2)
# nobatch.mofa.trained.TypeC <- run_mofa_default(
#   df.input = nobatch.mofa.input.C,
#   outfile = "mofa_results_250211_nobatch_TypeC_incl_lat_f7_v3",
#   metadata = meta.typeC,
#   covar = c("Population","Parasite","Population2","Ext.date","length_cm"),
#   view.distrib = c("gaussian","gaussian"),
#   num_factors = 7
# )

# # factor1 strongly correlated with no. of features features
```

Using 100 most variable microbes, no factor explains variation at
> 0.75% in both datasets.

```
nobatch.mofa.TypeC.variance <- read.csv("mofa_runs_20250211/mofa_results_250211_nobatch_TypeC_incl_lat_f7_v3_variance_per_factor.csv", row.names = 1)

nobatch.mofa.TypeC.variance <- rbind.data.frame(
  nobatch.mofa.TypeC.variance,
  data.frame(t(colSums(nobatch.mofa.TypeC.variance)), row.names = "Total")
)

nobatch.mofa.TypeC.variance <- melt(
  cbind.data.frame(
    data.frame(Factor=row.names(nobatch.mofa.TypeC.variance)),
    nobatch.mofa.TypeC.variance), 
  id.vars = "Factor", value.name = "variance", variable.name = "Dataset")

nobatch.mofa.TypeC.variance$Factor <- factor(nobatch.mofa.TypeC.variance$Factor,
                                             levels = c("Factor1", "Factor2", "Factor3", "Factor4", "Factor5", "Factor6", 
                                                        "Factor7", "Total"))

nobatch.mofa.plot.TypeC.variance <-
ggplot(nobatch.mofa.TypeC.variance, aes(Dataset, Factor))+
  geom_tile(aes(fill = variance))+
  geom_text(aes(label = round(variance, 3)))+
  geom_hline(yintercept = 7.5, linewidth = 0.75, colour = "black", linetype = 1)+
  scale_fill_gradient2(low="white", high="grey50", limits = c(0, 100), na.value = "white")+
  labs(title = "Non-exposed fish")+
  theme_classic()+
  theme(plot.title = element_text(hjust = 0.5, face = "bold", size = 13),
        axis.text = element_text(color = "black", size = 12), axis.title = element_text(size = 13),
        legend.text = element_text(size = 12), legend.title = element_text(size = 13),
        legend.position = "top")
nobatch.mofa.plot.TypeC.variance
```

```
nobatch.mofa.TypeC.corr <- read.csv("mofa_runs_20250211/mofa_results_250211_nobatch_TypeC_incl_lat_f7_v3_covariates_corr.csv")

nobatch.mofa.TypeC.corr <- subset(nobatch.mofa.TypeC.corr, Phenotype %in% c("Parasite","Population"))
nobatch.mofa.TypeC.corr <- rbind.data.frame(
  nobatch.mofa.TypeC.corr,
  data.frame(Factor="", Phenotype="Parasite",
             cor.r=NA, cor.logp=NA, cor.p=NA)
)
nobatch.mofa.TypeC.corr$Factor <- factor(nobatch.mofa.TypeC.corr$Factor,
                                         levels = c("Factor1", "Factor2", "Factor3", "Factor4", "Factor5", "Factor6",
                                                    "Factor7", ""))
nobatch.mofa.TypeC.corr$sig.label <- ifelse(nobatch.mofa.TypeC.corr$cor.p > 0.1,NA,
                                            ifelse(nobatch.mofa.TypeC.corr$cor.p > 0.05,".",
                                                   ifelse(nobatch.mofa.TypeC.corr$cor.p > 0.01,"*",
                                                          ifelse(nobatch.mofa.TypeC.corr$cor.p > 0.001,"**","***"))))
nobatch.mofa.plot.TypeC.corr <-
ggplot(nobatch.mofa.TypeC.corr, aes(Phenotype, Factor))+
  geom_tile(aes(fill = cor.r))+
  geom_hline(yintercept = 10.5, linewidth = 0.75, colour = "black", linetype = 1)+
  scale_fill_gradient2(low="#2166AC", mid="white", high="#B2182B", limits = c(-1, 1), na.value = "white")+
  geom_text(aes(label = sig.label))+
  labs(title = "", fill = "correlation")+
  theme_classic()+
  theme(plot.title = element_text(hjust = 0.5, face = "bold", size = 13),
        axis.text = element_text(color = "black", size = 12), axis.title = element_text(size = 13),
        axis.text.y = element_blank(), axis.title.y = element_blank(),
        legend.text = element_text(size = 12), legend.title = element_text(size = 13),
        legend.position = "top")
nobatch.mofa.plot.TypeC.corr
```

```
## Warning: Removed 12 rows containing missing values or values outside the scale range
## (`geom_text()`).
```

```
nobatch.mofa.TypeC.sample.weights <- read.csv("mofa_runs_20250211/mofa_results_250211_nobatch_TypeC_incl_lat_f7_v3_sample_weights.csv")
```

## predator-exposed

```
# library(MOFA2)
# library(reshape2)
# nobatch.mofa.trained.TypeP <- run_mofa_default(
#   df.input = nobatch.mofa.input.P,
#   outfile = "mofa_results_250211_nobatch_TypeP_incl_lat_f7_v3",
#   metadata = meta.typeP,
#   covar = c("Population","Parasite","Population2","Ext.date","length_cm"),
#   view.distrib = c("gaussian","gaussian"),
#   num_factors = 7
# )
# # factor1,4 strongly correlated with no. of features
```

Using 100 most variable microbes, Factor 1, 3 and 4 explain variation
at > 0.75% in both datasets.

```
nobatch.mofa.TypeP.variance <- read.csv("mofa_runs_20250211/mofa_results_250211_nobatch_TypeP_incl_lat_f7_v3_variance_per_factor.csv", row.names = 1)

nobatch.mofa.TypeP.variance <- rbind.data.frame(
  nobatch.mofa.TypeP.variance,
  data.frame(t(colSums(nobatch.mofa.TypeP.variance)), row.names = "Total")
)

nobatch.mofa.TypeP.variance <- melt(
  cbind.data.frame(
    data.frame(Factor=row.names(nobatch.mofa.TypeP.variance)),
    nobatch.mofa.TypeP.variance), 
  id.vars = "Factor", value.name = "variance", variable.name = "Dataset")

nobatch.mofa.TypeP.variance$Factor <- factor(nobatch.mofa.TypeP.variance$Factor,
                                             levels = c("Factor1", "Factor2", "Factor3", "Factor4", "Factor5", "Factor6", 
                                                        "Factor7", "Total"))

nobatch.mofa.plot.TypeP.variance <-
ggplot(nobatch.mofa.TypeP.variance, aes(Dataset, Factor))+
  geom_tile(aes(fill = variance))+
  geom_text(aes(label = round(variance, 3)))+
  geom_hline(yintercept = 7.5, linewidth = 0.75, colour = "black", linetype = 1)+
  scale_fill_gradient2(low="white", high="grey50", limits = c(0, 100), na.value = "white")+
  labs(title = "Predator-exposed fish")+
  theme_classic()+
  theme(plot.title = element_text(hjust = 0.5, face = "bold", size = 13),
        axis.text = element_text(color = "black", size = 12), axis.title = element_text(size = 13),
        legend.text = element_text(size = 12), legend.title = element_text(size = 13),
        legend.position = "top")
nobatch.mofa.plot.TypeP.variance
```

```
nobatch.mofa.TypeP.corr <- read.csv("mofa_runs_20250211/mofa_results_250211_nobatch_TypeP_incl_lat_f7_v3_covariates_corr.csv")

nobatch.mofa.TypeP.corr <- subset(nobatch.mofa.TypeP.corr, Phenotype %in% c("Parasite","Population"))
nobatch.mofa.TypeP.corr <- rbind.data.frame(
  nobatch.mofa.TypeP.corr,
  data.frame(Factor="", Phenotype="Population",
             cor.r=NA, cor.logp=NA, cor.p=NA)
)
nobatch.mofa.TypeP.corr$Factor <- factor(nobatch.mofa.TypeP.corr$Factor,
                                         levels = c("Factor1", "Factor2", "Factor3", "Factor4", "Factor5", "Factor6",
                                                    "Factor7", ""))
nobatch.mofa.TypeP.corr$sig.label <- ifelse(nobatch.mofa.TypeP.corr$cor.p > 0.1,NA,
                                            ifelse(nobatch.mofa.TypeP.corr$cor.p > 0.05,".",
                                                   ifelse(nobatch.mofa.TypeP.corr$cor.p > 0.01,"*",
                                                          ifelse(nobatch.mofa.TypeP.corr$cor.p > 0.001,"**","***"))))
nobatch.mofa.plot.TypeP.corr <-
ggplot(nobatch.mofa.TypeP.corr, aes(Phenotype, Factor))+
  geom_tile(aes(fill = cor.r))+
  geom_hline(yintercept = 10.5, linewidth = 0.75, colour = "black", linetype = 1)+
  scale_fill_gradient2(low="#2166AC", mid="white", high="#B2182B", limits = c(-1, 1), na.value = "white")+
  geom_text(aes(label = sig.label))+
  labs(title = "", fill = "correlation")+
  theme_classic()+
  theme(plot.title = element_text(hjust = 0.5, face = "bold", size = 13),
        axis.text = element_text(color = "black", size = 12), axis.title = element_text(size = 13),
        axis.text.y = element_blank(), axis.title.y = element_blank(),
        legend.text = element_text(size = 12), legend.title = element_text(size = 13),
        legend.position = "top")
nobatch.mofa.plot.TypeP.corr
```

```
## Warning: Removed 13 rows containing missing values or values outside the scale range
## (`geom_text()`).
```

```
nobatch.mofa.TypeP.sample.weights <- read.csv("mofa_runs_20250211/mofa_results_250211_nobatch_TypeP_incl_lat_f7_v3_sample_weights.csv")
```

## Main figures

### Figure 6

Results summary:

```
cowplot::plot_grid(nobatch.mofa.plot.TypeC.variance, nobatch.mofa.plot.TypeC.corr,
                   nobatch.mofa.plot.TypeP.variance, nobatch.mofa.plot.TypeP.corr,
                   align = "h", axis = "tb", nrow = 2, labels = c("A","B","C","D"),
                   rel_widths = c(1,0.5,1,0.5))
```

```
## Warning: Removed 12 rows containing missing values or values outside the scale range
## (`geom_text()`).
```

```
## Warning: Removed 13 rows containing missing values or values outside the scale range
## (`geom_text()`).
```

```
ggsave("figures_2024/Figure_6_mofa_variance_correlation.png",units = "in", dpi = 800, width = 7.5, height = 10)
```

Two or three multi-panel figures:

1. Predator-exposed fish, factor one, showing feature weights and
   sample weights for 1 microbe and 1 behaviour (no correlation with fish
   metadata)
2. Predator-exposed fish, factor three, as above (coloured by
   population, as there’s a weak correlation)
3. Predator-exposed fish, factor four (coloured by parasite
   status)

```
nobatch.mofa.TypeC.Factor1 <- mofa_figure_ind_factor(
  "mofa_runs_20250211/mofa_results_250211_nobatch_TypeC_incl_lat_f7_v3_feature_weights.csv",
  "Factor1", feature.num.pos = 10, feature.num.neg = 10
)

nobatch.mofa.TypeC.Factor2 <- mofa_figure_ind_factor(
  "mofa_runs_20250211/mofa_results_250211_nobatch_TypeC_incl_lat_f7_v3_feature_weights.csv",
  "Factor2", feature.num.pos = 10, feature.num.neg = 10
)

nobatch.mofa.TypeP.Factor1 <- mofa_figure_ind_factor(
  "mofa_runs_20250211/mofa_results_250211_nobatch_TypeP_incl_lat_f7_v3_feature_weights.csv",
  "Factor1", feature.num.pos = 10, feature.num.neg = 10
)

nobatch.mofa.TypeP.Factor3 <- mofa_figure_ind_factor(
  "mofa_runs_20250211/mofa_results_250211_nobatch_TypeP_incl_lat_f7_v3_feature_weights.csv",
  "Factor3", feature.num.pos = 10, feature.num.neg = 10
)

nobatch.mofa.TypeP.Factor4 <- mofa_figure_ind_factor(
  "mofa_runs_20250211/mofa_results_250211_nobatch_TypeP_incl_lat_f7_v3_feature_weights.csv",
  "Factor4", feature.num.pos = 10, feature.num.neg = 10
)

# annotate microbes based on google search of genera (only for the factors of interest)
micro.genera <- unique(c(
  nobatch.mofa.TypeC.Factor1$micro.top.pos, nobatch.mofa.TypeC.Factor1$micro.top.neg,
  nobatch.mofa.TypeC.Factor2$micro.top.pos, nobatch.mofa.TypeC.Factor2$micro.top.neg,
  nobatch.mofa.TypeP.Factor1$micro.top.pos, nobatch.mofa.TypeP.Factor1$micro.top.neg,
  nobatch.mofa.TypeP.Factor3$micro.top.pos, nobatch.mofa.TypeP.Factor3$micro.top.neg,
  nobatch.mofa.TypeP.Factor4$micro.top.pos, nobatch.mofa.TypeP.Factor4$micro.top.neg))

micro.genera <- as.data.frame(read.delim("mofa_runs_20250211/mofa_results_250211_nobatch_GENUS_ANNOTATIONS_subset_250217.txt", header = T, sep = "\t"))

# annotate behaviour
beh.cats <- list(
  boldness=c("Center_Duration_AT","Center_Duration_BT"),
  activity=c("Dtot_AT","Dtot_BT","Vang_AT","Vang_BT","VelBL_AT","VelBL_BT"),
  predator_response=c("Dist_to_Pred_AT","Dist_to_Pred_BT","Pred_duration_AT","Pred_duration_BT","Latency.time_AT")
)

beh.cats.cols <- list(boldness="#D81B60", activity="#004D40", predator_response="#FFC107", other="grey75")
micro.cats.cols <- list(fish_associated="#E41A1C", animal_associated="#377EB8", environmental="#9cd435", not_assessed="grey75")
```

### Figure 7 TypeP F1

```
nobatch.mofa.TypeP.Factor1 <- mofa_figure_ind_factor(
  "mofa_runs_20250211/mofa_results_250211_nobatch_TypeP_incl_lat_f7_v3_feature_weights.csv",
  "Factor1", feature.num.pos = 5, feature.num.neg = 5
)

nobatch.mofa.TypeP.Factor1$micro <- merge(
  nobatch.mofa.TypeP.Factor1$micro, micro.genera,
  by.x = "feature", by.y = "Genus", all.x = T, all.y = F
)

nobatch.mofa.TypeP.Factor1$micro$Putative.source[is.na(nobatch.mofa.TypeP.Factor1$micro$Putative.source)] <- "not_assessed"
nobatch.mofa.TypeP.Factor1$micro$Potential.fish.pathogen[is.na(nobatch.mofa.TypeP.Factor1$micro$Potential.fish.pathogen)] <- "not_assessed"

nobatch.mofa.TypeP.Factor1$micro$Putative.source <- factor(
  nobatch.mofa.TypeP.Factor1$micro$Putative.source,
  levels = names(micro.cats.cols)
)
nobatch.mofa.TypeP.Factor1$micro$Potential.fish.pathogen <- factor(
  nobatch.mofa.TypeP.Factor1$micro$Potential.fish.pathogen,
  levels = c("documented","not_documented","not_assessed")
)

nobatch.mofa.TypeP.Factor1$beh$anno2 <- factor(
  sapply(nobatch.mofa.TypeP.Factor1$beh$feature, function(x){
    i <- grep(x,beh.cats)
    if(length(i) == 0){
      "other"
    } else {
      names(beh.cats[i])[[1]]
    }
  }),
  levels = names(beh.cats.cols)
)

nobatch.mofa.TypeP.Factor1$beh$anno3 <- factor(
  sapply(as.character(nobatch.mofa.TypeP.Factor1$beh$anno), function(x){
    if(x == ""){
      ""
    } else {
      x <- gsub("duration", "dur", x, ignore.case = T)
      x <- gsub("freq", "freq", x, ignore.case = T)
      x <- gsub("\\.time","",x)
    }
  })
)

nobatch.mofa.TypeP.specific.plots.Factor1 <- get_feature_abundance_meta(
  t(as.data.frame(otu_table(subset_samples(clean.ps.genus.relab, Type == "Predator")))), 
  levels(nobatch.mofa.TypeP.Factor1$micro$anno)[-length(levels(nobatch.mofa.TypeP.Factor1$micro$anno))],
  meta.typeP,
  sample.var = "SampleID", sample.ids = samples.TypeP)

nobatch.mofa.TypeP.specific.plots.Factor1 <- melt(
  nobatch.mofa.TypeP.specific.plots.Factor1[,c("SampleID","feature","abundance","Population","Parasite","Population2","Type",
                                                  levels(nobatch.mofa.TypeP.Factor1$beh$anno)[-length(levels(nobatch.mofa.TypeP.Factor1$beh$anno))])],
  id.vars = c("SampleID","feature","abundance","Population","Parasite","Population2","Type"),
  measure.vars = levels(nobatch.mofa.TypeP.Factor1$beh$anno)[-length(levels(nobatch.mofa.TypeP.Factor1$beh$anno))],
  value.name = "beh.value", variable.name = "beh.trait")

nobatch.mofa.TypeP.specific.plots.Factor1 <- merge(
  nobatch.mofa.TypeP.specific.plots.Factor1, 
  subset(nobatch.mofa.TypeP.sample.weights, factor == "Factor1"), by.x = "SampleID", by.y = "sample")

nobatch.mofa.TypeP.specific.plots.Factor1$feature <- factor(
  nobatch.mofa.TypeP.specific.plots.Factor1$feature,
  levels = c(nobatch.mofa.TypeP.Factor1$micro.top.pos,nobatch.mofa.TypeP.Factor1$micro.top.neg)
)
nobatch.mofa.TypeP.specific.plots.Factor1$beh.trait <- factor(
  nobatch.mofa.TypeP.specific.plots.Factor1$beh.trait,
  levels = c(nobatch.mofa.TypeP.Factor1$beh.top.pos,nobatch.mofa.TypeP.Factor1$beh.top.neg)
)
```

```
plot.TypeP.F1.A <-
ggplot(nobatch.mofa.TypeP.Factor1$beh, aes(rank, value))+
  geom_hline(yintercept = 0, color = "grey50", linetype = 2)+
  geom_point(color = "grey60", size = 1, alpha = 0.7)+
  geom_point(aes(color = anno2, size = anno))+
  scale_color_manual(values = beh.cats.cols)+
  scale_size_manual(values = c(rep(4,10),2), guide = "none")+
  ggrepel::geom_text_repel(aes(label = anno3), size = 3.5,
                             max.overlaps = 20, point.padding = 3)+
  labs(y = "Weight", x = "Behavioural trait", color = "Behaviour type")+ 
  theme_classic()+
  theme(axis.text.x = element_blank(), axis.ticks.x = element_blank(),
        axis.text = element_text(color = "black", size = 12), legend.position = c(0.2, 0.8),
        axis.title = element_text(size = 13), legend.text = element_text(size = 10), legend.title = element_text(size = 11),
        legend.box = "horizontal", #plot.title = element_text(hjust = 0.5, face = "bold", size = 13),
        strip.text.x = element_text(size = 13), strip.background = element_blank())
```

```
## Warning: A numeric `legend.position` argument in `theme()` was deprecated in ggplot2
## 3.5.0.
## ℹ Please use the `legend.position.inside` argument of `theme()` instead.
## This warning is displayed once every 8 hours.
## Call `lifecycle::last_lifecycle_warnings()` to see where this warning was
## generated.
```

```
plot.TypeP.F1.B <-
ggplot(nobatch.mofa.TypeP.Factor1$micro, aes(rank, value))+
  geom_hline(yintercept = 0, color = "grey50", linetype = 2)+
  geom_point(color = "grey60", size = 1, alpha = 0.7)+
  geom_point(aes(color = Putative.source, shape = Potential.fish.pathogen, size = anno))+
  scale_color_manual(values = micro.cats.cols)+
  scale_shape_manual(values = c(17,15,16))+
  scale_size_manual(values = c(rep(4,10),2), guide = "none")+
  ggrepel::geom_text_repel(aes(label = anno), size = 3.5,
                           max.overlaps = 20)+
  expand_limits(x = c(-2, 102))+
  labs(y = "Weight", x = "Genus", color = "Source", shape = "Pathogen")+
  theme_classic()+
  theme(axis.text.x = element_blank(), axis.ticks.x = element_blank(),
        axis.text = element_text(color = "black", size = 12), legend.position = c(0.3, 0.8),
        axis.title = element_text(size = 13), legend.text = element_text(size = 10), legend.title = element_text(size = 11),
        legend.box = "horizontal",
        strip.text.x = element_blank(), strip.background = element_blank())
```

```
cowplot::plot_grid(plot.TypeP.F1.A, plot.TypeP.F1.B, nrow = 2, labels = c("A","B"),
                   align = "hv", axis = "lr")
```

Example with top microbe & top behaviour

```
plot.TypeP.F1.C <-
ggplot(subset(
  nobatch.mofa.TypeP.specific.plots.Factor1, 
  beh.trait %in% c(nobatch.mofa.TypeP.Factor1$beh.top.neg[2])),
  aes(value, beh.value))+
  geom_point(color = "grey20", size = 3)+
  geom_smooth(method=lm , color="black", fill="grey70", se=TRUE)+
  labs(x = "Sample weight", y = "Mean velocity (AT)")+
  theme_classic()+
  theme(axis.text = element_text(color = "black", size = 12), legend.position = c(0.8,0.2),
        axis.title = element_text(size = 13), legend.text = element_text(size = 10), legend.title = element_text(size = 11))

plot.TypeP.F1.D <-
ggplot(subset(
  nobatch.mofa.TypeP.specific.plots.Factor1, 
  feature %in% c(nobatch.mofa.TypeP.Factor1$micro.top.neg[1])), 
  aes(value, abundance))+
  geom_point(color = "grey20", size = 3)+
  geom_smooth(method=lm , color="black", fill="grey70", se=TRUE)+
  labs(x = "Sample weight", y = "Clostridioides abundance")+
  theme_classic()+
  theme(axis.text = element_text(color = "black", size = 12), legend.position = "none",
        axis.title = element_text(size = 13), legend.text = element_text(size = 11), legend.title = element_text(size = 12))
```

```
cowplot::plot_grid(plot.TypeP.F1.C, plot.TypeP.F1.D, nrow = 2, labels = c("A","B"),
                   align = "hv", axis = "lr")
```

```
## `geom_smooth()` using formula = 'y ~ x'
## `geom_smooth()` using formula = 'y ~ x'
```

```
cowplot::plot_grid(plot.TypeP.F1.A, plot.TypeP.F1.C, 
                   plot.TypeP.F1.B, plot.TypeP.F1.D, 
                   nrow = 2, rel_widths = c(1,0.7),
                   align = "hv", axis = "lr",
                   labels = c("A","C","B","D"))
```

```
## `geom_smooth()` using formula = 'y ~ x'
## `geom_smooth()` using formula = 'y ~ x'
```

```
ggsave("figures_2024/Figure_7_MOFA_TypeP_Factor1.png",units = "in", dpi = 800, width = 12, height = 8)
```

### Figure 8 TypeP F3

```
nobatch.mofa.TypeP.Factor3 <- mofa_figure_ind_factor(
  "mofa_runs_20250211/mofa_results_250211_nobatch_TypeP_incl_lat_f7_v3_feature_weights.csv",
  "Factor3", feature.num.pos = 5, feature.num.neg = 5
)

nobatch.mofa.TypeP.Factor3$micro <- merge(
  nobatch.mofa.TypeP.Factor3$micro, micro.genera,
  by.x = "feature", by.y = "Genus", all.x = T, all.y = F
)

nobatch.mofa.TypeP.Factor3$micro$Putative.source[is.na(nobatch.mofa.TypeP.Factor3$micro$Putative.source)] <- "not_assessed"
nobatch.mofa.TypeP.Factor3$micro$Potential.fish.pathogen[is.na(nobatch.mofa.TypeP.Factor3$micro$Potential.fish.pathogen)] <- "not_assessed"

nobatch.mofa.TypeP.Factor3$micro$Putative.source <- factor(
  nobatch.mofa.TypeP.Factor3$micro$Putative.source,
  levels = names(micro.cats.cols)
)
nobatch.mofa.TypeP.Factor3$micro$Potential.fish.pathogen <- factor(
  nobatch.mofa.TypeP.Factor3$micro$Potential.fish.pathogen,
  levels = c("documented","not_documented","not_assessed")
)

nobatch.mofa.TypeP.Factor3$beh$anno2 <- factor(
  sapply(nobatch.mofa.TypeP.Factor3$beh$feature, function(x){
    i <- grep(x,beh.cats)
    if(length(i) == 0){
      "other"
    } else {
      names(beh.cats[i])[[1]]
    }
  }),
  levels = names(beh.cats.cols)
)

nobatch.mofa.TypeP.Factor3$beh$anno3 <- factor(
  sapply(as.character(nobatch.mofa.TypeP.Factor3$beh$anno), function(x){
    if(x == ""){
      ""
    } else {
      x <- gsub("duration", "dur", x, ignore.case = T)
      x <- gsub("freq", "freq", x, ignore.case = T)
      x <- gsub("\\.time","",x)
    }
  })
)

nobatch.mofa.TypeP.specific.plots.Factor3 <- get_feature_abundance_meta(
  t(as.data.frame(otu_table(subset_samples(clean.ps.genus.relab, Type == "Predator")))), 
  levels(nobatch.mofa.TypeP.Factor3$micro$anno)[-length(levels(nobatch.mofa.TypeP.Factor3$micro$anno))],
  meta.typeP,
  sample.var = "SampleID", sample.ids = samples.TypeP)

nobatch.mofa.TypeP.specific.plots.Factor3 <- melt(
  nobatch.mofa.TypeP.specific.plots.Factor3[,c("SampleID","feature","abundance","Population","Parasite","Population2","Type",
                                                  levels(nobatch.mofa.TypeP.Factor3$beh$anno)[-length(levels(nobatch.mofa.TypeP.Factor3$beh$anno))])],
  id.vars = c("SampleID","feature","abundance","Population","Parasite","Population2","Type"),
  measure.vars = levels(nobatch.mofa.TypeP.Factor3$beh$anno)[-length(levels(nobatch.mofa.TypeP.Factor3$beh$anno))],
  value.name = "beh.value", variable.name = "beh.trait")

nobatch.mofa.TypeP.specific.plots.Factor3 <- merge(
  nobatch.mofa.TypeP.specific.plots.Factor3, 
  subset(nobatch.mofa.TypeP.sample.weights, factor == "Factor3"), by.x = "SampleID", by.y = "sample")

nobatch.mofa.TypeP.specific.plots.Factor3$feature <- factor(
  nobatch.mofa.TypeP.specific.plots.Factor3$feature,
  levels = c(nobatch.mofa.TypeP.Factor3$micro.top.pos,nobatch.mofa.TypeP.Factor3$micro.top.neg)
)
nobatch.mofa.TypeP.specific.plots.Factor3$beh.trait <- factor(
  nobatch.mofa.TypeP.specific.plots.Factor3$beh.trait,
  levels = c(nobatch.mofa.TypeP.Factor3$beh.top.pos,nobatch.mofa.TypeP.Factor3$beh.top.neg)
)
```

```
plot.TypeP.F3.A <-
ggplot(nobatch.mofa.TypeP.Factor3$beh, aes(rank, value))+
  geom_hline(yintercept = 0, color = "grey50", linetype = 2)+
  geom_point(color = "grey60", size = 1, alpha = 0.7)+
  geom_point(aes(color = anno2, size = anno))+
  scale_color_manual(values = beh.cats.cols)+
  scale_size_manual(values = c(rep(4,10),2), guide = "none")+
  ggrepel::geom_text_repel(aes(label = anno3), size = 3.5,
                           max.overlaps = 20, point.padding = 3)+
  labs(y = "Weight", x = "Behavioural trait", color = "Behaviour type")+ 
  theme_classic()+
  theme(axis.text.x = element_blank(), axis.ticks.x = element_blank(),
        axis.text = element_text(color = "black", size = 12), legend.position = c(0.2, 0.8),
        axis.title = element_text(size = 13), legend.text = element_text(size = 10), legend.title = element_text(size = 11),
        legend.box = "horizontal", #plot.title = element_text(hjust = 0.5, face = "bold", size = 13),
        strip.text.x = element_text(size = 13), strip.background = element_blank())

plot.TypeP.F3.B <-
ggplot(nobatch.mofa.TypeP.Factor3$micro, aes(rank, value))+
  geom_hline(yintercept = 0, color = "grey50", linetype = 2)+
  geom_point(color = "grey60", size = 1, alpha = 0.7)+
  geom_point(aes(color = Putative.source, shape = Potential.fish.pathogen, size = anno))+
  scale_color_manual(values = micro.cats.cols)+
  scale_shape_manual(values = c(17,15,16))+
  scale_size_manual(values = c(rep(4,10),2), guide = "none")+
  ggrepel::geom_text_repel(aes(label = anno), size = 3.5,
                           max.overlaps = 20)+
  expand_limits(x = c(-2, 102))+
  labs(y = "Weight", x = "Genus", color = "Source", shape = "Pathogen")+
  theme_classic()+
  theme(axis.text.x = element_blank(), axis.ticks.x = element_blank(),
        axis.text = element_text(color = "black", size = 12), legend.position = c(0.3, 0.8),
        axis.title = element_text(size = 13), legend.text = element_text(size = 10), legend.title = element_text(size = 11),
        legend.box = "horizontal",
        strip.text.x = element_blank(), strip.background = element_blank())
```

```
cowplot::plot_grid(plot.TypeP.F3.A, plot.TypeP.F3.B, nrow = 2, labels = c("A","B"),
                   align = "hv", axis = "lr")
```

Example with top microbe & top behaviour

```
plot.TypeP.F3.Cv2 <-
ggplot(subset(
  nobatch.mofa.TypeP.specific.plots.Factor3, 
  beh.trait %in% c(nobatch.mofa.TypeP.Factor3$beh.top.pos[1])),
  aes(value, beh.value))+
  geom_point(aes(color = Population), size = 3)+
  scale_color_manual(values = gg_color_hue(4)[c(2,4)],
                     labels = c("Galtaból","Þristikla"))+
  geom_smooth(method=lm , color="black", fill="grey70", se=TRUE)+
  labs(x = "Sample weight", y = "Angular velocity (BT)")+
  theme_classic()+
  theme(axis.text = element_text(color = "black", size = 12), legend.position = c(0.15,0.85),
        axis.title = element_text(size = 13), legend.text = element_text(size = 11), legend.title = element_text(size = 12))

plot.TypeP.F3.Dv2 <-
ggplot(subset(
  nobatch.mofa.TypeP.specific.plots.Factor3, 
  feature %in% c(nobatch.mofa.TypeP.Factor3$micro.top.pos[1])), 
  aes(value, abundance))+
  geom_point(aes(color = Population), size = 3)+
  scale_color_manual(values = gg_color_hue(4)[c(2,4)],
                     labels = c("Galtaból","Þristikla"))+
  geom_smooth(method=lm , color="black", fill="grey70", se=TRUE)+
  labs(x = "Sample weight", y = "Paracoccus abundance")+
  theme_classic()+
  theme(axis.text = element_text(color = "black", size = 12), legend.position = "none",
        axis.title = element_text(size = 13), legend.text = element_text(size = 11), legend.title = element_text(size = 12))
```

```
cowplot::plot_grid(plot.TypeP.F3.Cv2, plot.TypeP.F3.Dv2, nrow = 2, labels = c("A","B"),
                   align = "hv", axis = "lr")
```

```
## `geom_smooth()` using formula = 'y ~ x'
## `geom_smooth()` using formula = 'y ~ x'
```

```
cowplot::plot_grid(plot.TypeP.F3.A, plot.TypeP.F3.Cv2, 
                   plot.TypeP.F3.B, plot.TypeP.F3.Dv2, 
                   nrow = 2, rel_widths = c(1,0.7),
                   align = "hv", axis = "lr",
                   labels = c("A","C","B","D"))
```

```
## `geom_smooth()` using formula = 'y ~ x'
## `geom_smooth()` using formula = 'y ~ x'
```

```
ggsave("figures_2024/Figure_8_MOFA_TypeP_Factor3.png",units = "in", dpi = 800, width = 12, height = 8)
```

### Figure 9 TypeP F4

```
nobatch.mofa.TypeP.Factor4 <- mofa_figure_ind_factor(
  "mofa_runs_20250211/mofa_results_250211_nobatch_TypeP_incl_lat_f7_v3_feature_weights.csv",
  "Factor4", feature.num.pos = 5, feature.num.neg = 5
)

nobatch.mofa.TypeP.Factor4$micro <- merge(
  nobatch.mofa.TypeP.Factor4$micro, micro.genera,
  by.x = "feature", by.y = "Genus", all.x = T, all.y = F
)

nobatch.mofa.TypeP.Factor4$micro$Putative.source[is.na(nobatch.mofa.TypeP.Factor4$micro$Putative.source)] <- "not_assessed"
nobatch.mofa.TypeP.Factor4$micro$Potential.fish.pathogen[is.na(nobatch.mofa.TypeP.Factor4$micro$Potential.fish.pathogen)] <- "not_assessed"

nobatch.mofa.TypeP.Factor4$micro$Putative.source <- factor(
  nobatch.mofa.TypeP.Factor4$micro$Putative.source,
  levels = names(micro.cats.cols)
)
nobatch.mofa.TypeP.Factor4$micro$Potential.fish.pathogen <- factor(
  nobatch.mofa.TypeP.Factor4$micro$Potential.fish.pathogen,
  levels = c("documented","not_documented","not_assessed")
)

nobatch.mofa.TypeP.Factor4$beh$anno2 <- factor(
  sapply(nobatch.mofa.TypeP.Factor4$beh$feature, function(x){
    i <- grep(x,beh.cats)
    if(length(i) == 0){
      "other"
    } else {
      names(beh.cats[i])[[1]]
    }
  }),
  levels = names(beh.cats.cols)
)

nobatch.mofa.TypeP.Factor4$beh$anno3 <- factor(
  sapply(as.character(nobatch.mofa.TypeP.Factor4$beh$anno), function(x){
    if(x == ""){
      ""
    } else {
      x <- gsub("duration", "dur", x, ignore.case = T)
      x <- gsub("freq", "freq", x, ignore.case = T)
      x <- gsub("\\.time","",x)
    }
  })
)

nobatch.mofa.TypeP.specific.plots.Factor4 <- get_feature_abundance_meta(
  t(as.data.frame(otu_table(subset_samples(clean.ps.genus.relab, Type == "Predator")))), 
  levels(nobatch.mofa.TypeP.Factor4$micro$anno)[-length(levels(nobatch.mofa.TypeP.Factor4$micro$anno))],
  meta.typeP,
  sample.var = "SampleID", sample.ids = samples.TypeP)

nobatch.mofa.TypeP.specific.plots.Factor4 <- melt(
  nobatch.mofa.TypeP.specific.plots.Factor4[,c("SampleID","feature","abundance","Population","Parasite","Population2","Type",
                                                  levels(nobatch.mofa.TypeP.Factor4$beh$anno)[-length(levels(nobatch.mofa.TypeP.Factor4$beh$anno))])],
  id.vars = c("SampleID","feature","abundance","Population","Parasite","Population2","Type"),
  measure.vars = levels(nobatch.mofa.TypeP.Factor4$beh$anno)[-length(levels(nobatch.mofa.TypeP.Factor4$beh$anno))],
  value.name = "beh.value", variable.name = "beh.trait")

nobatch.mofa.TypeP.specific.plots.Factor4 <- merge(
  nobatch.mofa.TypeP.specific.plots.Factor4, 
  subset(nobatch.mofa.TypeP.sample.weights, factor == "Factor4"), by.x = "SampleID", by.y = "sample")

nobatch.mofa.TypeP.specific.plots.Factor4$feature <- factor(
  nobatch.mofa.TypeP.specific.plots.Factor4$feature,
  levels = c(nobatch.mofa.TypeP.Factor4$micro.top.pos,nobatch.mofa.TypeP.Factor4$micro.top.neg)
)
nobatch.mofa.TypeP.specific.plots.Factor4$beh.trait <- factor(
  nobatch.mofa.TypeP.specific.plots.Factor4$beh.trait,
  levels = c(nobatch.mofa.TypeP.Factor4$beh.top.pos,nobatch.mofa.TypeP.Factor4$beh.top.neg)
)
```

```
plot.TypeP.F4.A <-
ggplot(nobatch.mofa.TypeP.Factor4$beh, aes(rank, value))+
  geom_hline(yintercept = 0, color = "grey50", linetype = 2)+
  geom_point(color = "grey60", size = 1, alpha = 0.7)+
  geom_point(aes(color = anno2, size = anno))+
  scale_color_manual(values = beh.cats.cols)+
  scale_size_manual(values = c(rep(4,10),2), guide = "none")+
  ggrepel::geom_text_repel(aes(label = anno3), size = 3.5,
                             max.overlaps = 20, point.padding = 3)+
  labs(y = "Weight", x = "Behavioural trait", color = "Behaviour type")+ 
  theme_classic()+
  theme(axis.text.x = element_blank(), axis.ticks.x = element_blank(),
        axis.text = element_text(color = "black", size = 12), legend.position = c(0.2, 0.8),
        axis.title = element_text(size = 13), legend.text = element_text(size = 10), legend.title = element_text(size = 11),
        legend.box = "horizontal", #plot.title = element_text(hjust = 0.5, face = "bold", size = 13),
        strip.text.x = element_text(size = 13), strip.background = element_blank())

plot.TypeP.F4.B <-
ggplot(nobatch.mofa.TypeP.Factor4$micro, aes(rank, value))+
  geom_hline(yintercept = 0, color = "grey50", linetype = 2)+
  geom_point(color = "grey60", size = 1, alpha = 0.7)+
  geom_point(aes(color = Putative.source, shape = Potential.fish.pathogen, size = anno))+
  scale_color_manual(values = micro.cats.cols)+
  scale_shape_manual(values = c(17,15,16))+
  scale_size_manual(values = c(rep(4,10),2), guide = "none")+
  ggrepel::geom_text_repel(aes(label = anno), size = 3.5,
                           max.overlaps = 20)+
  expand_limits(x = c(-2, 102))+
  labs(y = "Weight", x = "Genus", color = "Source", shape = "Pathogen")+
  theme_classic()+
  theme(axis.text.x = element_blank(), axis.ticks.x = element_blank(),
        axis.text = element_text(color = "black", size = 12), legend.position = c(0.3, 0.8),
        axis.title = element_text(size = 13), legend.text = element_text(size = 10), legend.title = element_text(size = 11),
        legend.box = "horizontal",
        strip.text.x = element_blank(), strip.background = element_blank())
```

```
cowplot::plot_grid(plot.TypeP.F4.A, plot.TypeP.F4.B, nrow = 2, labels = c("A","B"),
                   align = "hv", axis = "lr")
```

Example with top microbe & top behaviour

```
plot.TypeP.F4.C <-
ggplot(subset(
  nobatch.mofa.TypeP.specific.plots.Factor4, 
  beh.trait %in% c(nobatch.mofa.TypeP.Factor4$beh.top.pos[1])),
  aes(value, beh.value))+
  geom_point(aes(color = Parasite), size = 3)+
  scale_color_manual(values = gg_color_hue(4)[c(2,3)],
                     labels = c("Non-parasitized","Parasitized"))+
  geom_smooth(method=lm , color="black", fill="grey70", se=TRUE)+
  labs(x = "Sample weight", y = "Center duration (AT)")+
  theme_classic()+
  theme(axis.text = element_text(color = "black", size = 12), legend.position = c(0.2,0.8),
        axis.title = element_text(size = 13), legend.text = element_text(size = 10), legend.title = element_text(size = 11))

plot.TypeP.F4.D <-
ggplot(subset(
  nobatch.mofa.TypeP.specific.plots.Factor4, 
  feature %in% c(nobatch.mofa.TypeP.Factor4$micro.top.pos[3])), 
  aes(value, abundance))+
  geom_point(aes(color = Parasite), size = 3)+
  scale_color_manual(values = gg_color_hue(4)[c(2,3)],
                     labels = c("Non-parasitized","Parasitized"))+
  geom_smooth(method=lm , color="black", fill="grey70", se=TRUE)+
  labs(x = "Sample weight", y = "Deefgea abundance")+
  theme_classic()+
  theme(axis.text = element_text(color = "black", size = 12), legend.position = "none",
        axis.title = element_text(size = 13), legend.text = element_text(size = 11), legend.title = element_text(size = 12))
```

```
cowplot::plot_grid(plot.TypeP.F4.C, plot.TypeP.F4.D, nrow = 2, labels = c("A","B"),
                   align = "hv", axis = "lr")
```

```
## `geom_smooth()` using formula = 'y ~ x'
## `geom_smooth()` using formula = 'y ~ x'
```

```
cowplot::plot_grid(plot.TypeP.F4.A, plot.TypeP.F4.C, 
                   plot.TypeP.F4.B, plot.TypeP.F4.D, 
                   nrow = 2, rel_widths = c(1,0.7),
                   align = "hv", axis = "lr",
                   labels = c("A","C","B","D"))
```

```
## `geom_smooth()` using formula = 'y ~ x'
## `geom_smooth()` using formula = 'y ~ x'
```

```
ggsave("figures_2024/Figure_9_MOFA_TypeP_Factor4.png",units = "in", dpi = 800, width = 12, height = 8)
```

## Supplementary figures

### TypeC F1

```
# Run function to label top 5 in each direction
nobatch.mofa.TypeC.Factor1 <- mofa_figure_ind_factor(
  "mofa_runs_20250211/mofa_results_250211_nobatch_TypeC_incl_lat_f7_v3_feature_weights.csv",
  "Factor1", feature.num.pos = 5, feature.num.neg = 5
)

nobatch.mofa.TypeC.Factor1$micro <- merge(
  nobatch.mofa.TypeC.Factor1$micro, micro.genera,
  by.x = "feature", by.y = "Genus", all.x = T, all.y = F
)

nobatch.mofa.TypeC.Factor1$micro$Putative.source[is.na(nobatch.mofa.TypeC.Factor1$micro$Putative.source)] <- "not_assessed"
nobatch.mofa.TypeC.Factor1$micro$Potential.fish.pathogen[is.na(nobatch.mofa.TypeC.Factor1$micro$Potential.fish.pathogen)] <- "not_assessed"

nobatch.mofa.TypeC.Factor1$micro$Putative.source <- factor(
  nobatch.mofa.TypeC.Factor1$micro$Putative.source,
  levels = names(micro.cats.cols)
)
nobatch.mofa.TypeC.Factor1$micro$Potential.fish.pathogen <- factor(
  nobatch.mofa.TypeC.Factor1$micro$Potential.fish.pathogen,
  levels = c("documented","not_documented","not_assessed")
)

nobatch.mofa.TypeC.Factor1$beh$anno2 <- factor(
  sapply(nobatch.mofa.TypeC.Factor1$beh$feature, function(x){
    i <- grep(x,beh.cats)
    if(length(i) == 0){
      "other"
    } else {
      names(beh.cats[i])[[1]]
    }
  }),
  levels = names(beh.cats.cols)
)

nobatch.mofa.TypeC.Factor1$beh$anno3 <- factor(
  sapply(as.character(nobatch.mofa.TypeC.Factor1$beh$anno), function(x){
    if(x == ""){
      ""
    } else {
      x <- gsub("duration", "dur", x, ignore.case = T)
      x <- gsub("freq", "freq", x, ignore.case = T)
      x <- gsub("\\.time","",x)
    }
  })
)

nobatch.mofa.TypeC.specific.plots.Factor1 <- get_feature_abundance_meta(
  t(as.data.frame(otu_table(subset_samples(clean.ps.genus.relab, Type == "Control")))), 
  levels(nobatch.mofa.TypeC.Factor1$micro$anno)[-length(levels(nobatch.mofa.TypeC.Factor1$micro$anno))],
  meta.typeC,
  sample.var = "SampleID", sample.ids = samples.TypeC)

nobatch.mofa.TypeC.specific.plots.Factor1 <- melt(
  nobatch.mofa.TypeC.specific.plots.Factor1[,c("SampleID","feature","abundance","Population","Parasite","Population2","Type",
                                                  levels(nobatch.mofa.TypeC.Factor1$beh$anno)[-length(levels(nobatch.mofa.TypeC.Factor1$beh$anno))])],
  id.vars = c("SampleID","feature","abundance","Population","Parasite","Population2","Type"),
  measure.vars = levels(nobatch.mofa.TypeC.Factor1$beh$anno)[-length(levels(nobatch.mofa.TypeC.Factor1$beh$anno))],
  value.name = "beh.value", variable.name = "beh.trait")

nobatch.mofa.TypeC.specific.plots.Factor1 <- merge(
  nobatch.mofa.TypeC.specific.plots.Factor1, 
  subset(nobatch.mofa.TypeC.sample.weights, factor == "Factor1"), by.x = "SampleID", by.y = "sample")

nobatch.mofa.TypeC.specific.plots.Factor1$feature <- factor(
  nobatch.mofa.TypeC.specific.plots.Factor1$feature,
  levels = c(nobatch.mofa.TypeC.Factor1$micro.top.pos,nobatch.mofa.TypeC.Factor1$micro.top.neg)
)
nobatch.mofa.TypeC.specific.plots.Factor1$beh.trait <- factor(
  nobatch.mofa.TypeC.specific.plots.Factor1$beh.trait,
  levels = c(nobatch.mofa.TypeC.Factor1$beh.top.pos,nobatch.mofa.TypeC.Factor1$beh.top.neg)
)
```

```
plot.TypeC.F1.A <-
ggplot(nobatch.mofa.TypeC.Factor1$beh, aes(rank, value))+
  geom_hline(yintercept = 0, color = "grey50", linetype = 2)+
  geom_point(color = "grey60", size = 1, alpha = 0.7)+
  geom_point(aes(color = anno2, size = anno))+
  scale_color_manual(values = beh.cats.cols)+
  scale_size_manual(values = c(rep(4,10),2), guide = "none")+
  ggrepel::geom_text_repel(aes(label = anno3), size = 3.5,
                           max.overlaps = 20, point.padding = 3)+
  labs(y = "Weight", x = "Behavioural trait", color = "Behaviour type", 
       title = "Non-exposed fish - factor 1")+
  theme_classic()+
  theme(axis.text.x = element_blank(), axis.ticks.x = element_blank(),
        axis.text = element_text(color = "black", size = 12), legend.position = c(0.2, 0.8),
        axis.title = element_text(size = 13), legend.text = element_text(size = 10), legend.title = element_text(size = 11),
        legend.box = "horizontal", plot.title = element_text(hjust = 0.5, face = "bold", size = 13),
        strip.text.x = element_text(size = 13), strip.background = element_blank())

plot.TypeC.F1.B <-
ggplot(nobatch.mofa.TypeC.Factor1$micro, aes(rank, value))+
  geom_hline(yintercept = 0, color = "grey50", linetype = 2)+
  geom_point(color = "grey60", size = 1, alpha = 0.7)+
  geom_point(aes(color = Putative.source, shape = Potential.fish.pathogen, size = anno))+
  scale_color_manual(values = micro.cats.cols)+
  scale_shape_manual(values = c(17,15,16))+
  scale_size_manual(values = c(rep(4,10),2), guide = "none")+
  ggrepel::geom_text_repel(aes(label = anno), size = 3.5,
                           max.overlaps = 20)+
  expand_limits(x = c(-2, 102))+
  labs(y = "Weight", x = "Genus", color = "Source", shape = "Pathogen")+
  theme_classic()+
  theme(axis.text.x = element_blank(), axis.ticks.x = element_blank(),
        axis.text = element_text(color = "black", size = 12), legend.position = c(0.3, 0.8),
        axis.title = element_text(size = 13), legend.text = element_text(size = 10), legend.title = element_text(size = 11),
        legend.box = "horizontal",
        strip.text.x = element_blank(), strip.background = element_blank())
```

```
cowplot::plot_grid(plot.TypeC.F1.A, plot.TypeC.F1.B, nrow = 2, labels = c("A","B"),
                   align = "hv", axis = "lr")
```

```
ggsave("figures_2024/Supp_figure_S4_MOFA_TypeC_Factor1.png",units = "in", dpi = 600, width = 9.5, height = 8)
```

### TypeC F2

```
# Run function to label top 5 in each direction
nobatch.mofa.TypeC.Factor2 <- mofa_figure_ind_factor(
  "mofa_runs_20250211/mofa_results_250211_nobatch_TypeC_incl_lat_f7_v3_feature_weights.csv",
  "Factor2", feature.num.pos = 5, feature.num.neg = 5
)

nobatch.mofa.TypeC.Factor2$micro <- merge(
  nobatch.mofa.TypeC.Factor2$micro, micro.genera,
  by.x = "feature", by.y = "Genus", all.x = T, all.y = F
)

nobatch.mofa.TypeC.Factor2$micro$Putative.source[is.na(nobatch.mofa.TypeC.Factor2$micro$Putative.source)] <- "not_assessed"
nobatch.mofa.TypeC.Factor2$micro$Potential.fish.pathogen[is.na(nobatch.mofa.TypeC.Factor2$micro$Potential.fish.pathogen)] <- "not_assessed"

nobatch.mofa.TypeC.Factor2$micro$Putative.source <- factor(
  nobatch.mofa.TypeC.Factor2$micro$Putative.source,
  levels = names(micro.cats.cols)
)
nobatch.mofa.TypeC.Factor2$micro$Potential.fish.pathogen <- factor(
  nobatch.mofa.TypeC.Factor2$micro$Potential.fish.pathogen,
  levels = c("documented","not_documented","not_assessed")
)

nobatch.mofa.TypeC.Factor2$beh$anno2 <- factor(
  sapply(nobatch.mofa.TypeC.Factor2$beh$feature, function(x){
    i <- grep(x,beh.cats)
    if(length(i) == 0){
      "other"
    } else {
      names(beh.cats[i])[[1]]
    }
  }),
  levels = names(beh.cats.cols)
)

nobatch.mofa.TypeC.Factor2$beh$anno3 <- factor(
  sapply(as.character(nobatch.mofa.TypeC.Factor2$beh$anno), function(x){
    if(x == ""){
      ""
    } else {
      x <- gsub("duration", "dur", x, ignore.case = T)
      x <- gsub("freq", "freq", x, ignore.case = T)
      x <- gsub("\\.time","",x)
    }
  })
)

nobatch.mofa.TypeC.specific.plots.Factor2 <- get_feature_abundance_meta(
  t(as.data.frame(otu_table(subset_samples(clean.ps.genus.relab, Type == "Control")))), 
  levels(nobatch.mofa.TypeC.Factor2$micro$anno)[-length(levels(nobatch.mofa.TypeC.Factor2$micro$anno))],
  meta.typeC,
  sample.var = "SampleID", sample.ids = samples.TypeC)

nobatch.mofa.TypeC.specific.plots.Factor2 <- melt(
  nobatch.mofa.TypeC.specific.plots.Factor2[,c("SampleID","feature","abundance","Population","Parasite","Population2","Type",
                                                  levels(nobatch.mofa.TypeC.Factor2$beh$anno)[-length(levels(nobatch.mofa.TypeC.Factor2$beh$anno))])],
  id.vars = c("SampleID","feature","abundance","Population","Parasite","Population2","Type"),
  measure.vars = levels(nobatch.mofa.TypeC.Factor2$beh$anno)[-length(levels(nobatch.mofa.TypeC.Factor2$beh$anno))],
  value.name = "beh.value", variable.name = "beh.trait")

nobatch.mofa.TypeC.specific.plots.Factor2 <- merge(
  nobatch.mofa.TypeC.specific.plots.Factor2, 
  subset(nobatch.mofa.TypeC.sample.weights, factor == "Factor2"), by.x = "SampleID", by.y = "sample")

nobatch.mofa.TypeC.specific.plots.Factor2$feature <- factor(
  nobatch.mofa.TypeC.specific.plots.Factor2$feature,
  levels = c(nobatch.mofa.TypeC.Factor2$micro.top.pos,nobatch.mofa.TypeC.Factor2$micro.top.neg)
)
nobatch.mofa.TypeC.specific.plots.Factor2$beh.trait <- factor(
  nobatch.mofa.TypeC.specific.plots.Factor2$beh.trait,
  levels = c(nobatch.mofa.TypeC.Factor2$beh.top.pos,nobatch.mofa.TypeC.Factor2$beh.top.neg)
)
```

```
plot.TypeC.F2.A <-
ggplot(nobatch.mofa.TypeC.Factor2$beh, aes(rank, value))+
  geom_hline(yintercept = 0, color = "grey50", linetype = 2)+
  geom_point(color = "grey60", size = 1, alpha = 0.7)+
  geom_point(aes(color = anno2, size = anno))+
  scale_color_manual(values = beh.cats.cols)+
  scale_size_manual(values = c(rep(4,10),2), guide = "none")+
  ggrepel::geom_text_repel(aes(label = anno3), size = 3.5,
                           max.overlaps = 20, point.padding = 3)+
  labs(y = "Weight", x = "Behavioural trait", color = "Behaviour type", 
       title = "Non-exposed fish - factor 2")+
  theme_classic()+
  theme(axis.text.x = element_blank(), axis.ticks.x = element_blank(),
        axis.text = element_text(color = "black", size = 12), legend.position = c(0.2, 0.8),
        axis.title = element_text(size = 13), legend.text = element_text(size = 10), legend.title = element_text(size = 11),
        legend.box = "horizontal", plot.title = element_text(hjust = 0.5, face = "bold", size = 13),
        strip.text.x = element_text(size = 13), strip.background = element_blank())

plot.TypeC.F2.B <-
ggplot(nobatch.mofa.TypeC.Factor2$micro, aes(rank, value))+
  geom_hline(yintercept = 0, color = "grey50", linetype = 2)+
  geom_point(color = "grey60", size = 1, alpha = 0.7)+
  geom_point(aes(color = Putative.source, shape = Potential.fish.pathogen, size = anno))+
  scale_color_manual(values = micro.cats.cols)+
  scale_shape_manual(values = c(17,15,16))+
  scale_size_manual(values = c(rep(4,10),2), guide = "none")+
  ggrepel::geom_text_repel(aes(label = anno), size = 3.5,
                           max.overlaps = 20)+
  expand_limits(x = c(-2, 102))+
  labs(y = "Weight", x = "Genus", color = "Source", shape = "Pathogen")+
  theme_classic()+
  theme(axis.text.x = element_blank(), axis.ticks.x = element_blank(),
        axis.text = element_text(color = "black", size = 12), legend.position = c(0.7, 0.3),
        axis.title = element_text(size = 13), legend.text = element_text(size = 10), legend.title = element_text(size = 11),
        legend.box = "horizontal",
        strip.text.x = element_blank(), strip.background = element_blank())
```

```
cowplot::plot_grid(plot.TypeC.F2.A, plot.TypeC.F2.B, nrow = 2, labels = c("A","B"),
                   align = "hv", axis = "lr")
```

Example with top microbe & top behaviour

```
plot.TypeC.F2.C <-
ggplot(subset(
  nobatch.mofa.TypeC.specific.plots.Factor2, 
  beh.trait %in% c(nobatch.mofa.TypeC.Factor2$beh.top.neg[1])), 
  aes(value, beh.value))+
  geom_point(aes(color = Population), size = 3)+
  scale_color_manual(values = gg_color_hue(4)[c(2,4)],
                     labels = c("Galtaból","Þristikla"))+
  geom_smooth(method=lm , color="black", fill="grey70", se=TRUE)+
  labs(x = "Sample weight", y = "Center duration (BT)")+
       # title = "Control fish - Factor2")+
  theme_classic()+
  theme(axis.text = element_text(color = "black", size = 12), legend.position = c(0.2,0.2),
        axis.title = element_text(size = 13), legend.text = element_text(size = 10), legend.title = element_text(size = 11))

plot.TypeC.F2.D <-
ggplot(subset(
  nobatch.mofa.TypeC.specific.plots.Factor2, 
  feature %in% c(nobatch.mofa.TypeC.Factor2$micro.top.neg[1])), 
  aes(value, abundance))+
  geom_point(aes(color = Population), size = 3)+
  scale_color_manual(values = gg_color_hue(4)[c(2,4)],
                     labels = c("Galtaból","Þristikla"))+
  geom_smooth(method=lm , color="black", fill="grey70", se=TRUE)+
  labs(x = "Sample weight", y = "Massilia abundance")+
       # title = "Control fish - Factor2")+
  theme_classic()+
  theme(axis.text = element_text(color = "black", size = 12), legend.position = "none",
        axis.title = element_text(size = 13), legend.text = element_text(size = 10), legend.title = element_text(size = 11))
```

```
cowplot::plot_grid(plot.TypeC.F2.C, plot.TypeC.F2.D, nrow = 2, labels = c("A","B"),
                   align = "hv", axis = "lr")
```

```
## `geom_smooth()` using formula = 'y ~ x'
## `geom_smooth()` using formula = 'y ~ x'
```

```
cowplot::plot_grid(plot.TypeC.F2.A, plot.TypeC.F2.C, 
                   plot.TypeC.F2.B, plot.TypeC.F2.D, 
                   nrow = 2, rel_widths = c(1,0.7),
                   align = "hv", axis = "lr",
                   labels = c("A","C","B","D"))
```

```
## `geom_smooth()` using formula = 'y ~ x'
## `geom_smooth()` using formula = 'y ~ x'
```

```
ggsave("figures_2024/Supp_figure_S5_MOFA_TypeC_Factor2.png",units = "in", dpi = 800, width = 12, height = 8)
```

### TypeC F3

```
nobatch.mofa.TypeC.Factor3 <- mofa_figure_ind_factor(
  "mofa_runs_20250211/mofa_results_250211_nobatch_TypeC_incl_lat_f7_v3_feature_weights.csv",
  "Factor3", feature.num.pos = 5, feature.num.neg = 5
)

nobatch.mofa.TypeC.Factor3$micro <- merge(
  nobatch.mofa.TypeC.Factor3$micro, micro.genera,
  by.x = "feature", by.y = "Genus", all.x = T, all.y = F
)

nobatch.mofa.TypeC.Factor3$micro$Putative.source[is.na(nobatch.mofa.TypeC.Factor3$micro$Putative.source)] <- "not_assessed"
nobatch.mofa.TypeC.Factor3$micro$Potential.fish.pathogen[is.na(nobatch.mofa.TypeC.Factor3$micro$Potential.fish.pathogen)] <- "not_assessed"

nobatch.mofa.TypeC.Factor3$micro$Putative.source <- factor(
  nobatch.mofa.TypeC.Factor3$micro$Putative.source,
  levels = names(micro.cats.cols)
)
nobatch.mofa.TypeC.Factor3$micro$Potential.fish.pathogen <- factor(
  nobatch.mofa.TypeC.Factor3$micro$Potential.fish.pathogen,
  levels = c("documented","not_documented","not_assessed")
)

nobatch.mofa.TypeC.Factor3$beh$anno2 <- factor(
  sapply(nobatch.mofa.TypeC.Factor3$beh$feature, function(x){
    i <- grep(x,beh.cats)
    if(length(i) == 0){
      "other"
    } else {
      names(beh.cats[i])[[1]]
    }
  }),
  levels = names(beh.cats.cols)
)

nobatch.mofa.TypeC.Factor3$beh$anno3 <- factor(
  sapply(as.character(nobatch.mofa.TypeC.Factor3$beh$anno), function(x){
    if(x == ""){
      ""
    } else {
      x <- gsub("duration", "dur", x, ignore.case = T)
      x <- gsub("freq", "freq", x, ignore.case = T)
      x <- gsub("\\.time","",x)
    }
  })
)
```

```
plot.TypeC.F3.A <-
ggplot(nobatch.mofa.TypeC.Factor3$beh, aes(rank, value))+
  geom_hline(yintercept = 0, color = "grey50", linetype = 2)+
  geom_point(color = "grey60", size = 1, alpha = 0.7)+
  geom_point(aes(color = anno2, size = anno))+
  scale_color_manual(values = beh.cats.cols)+
  scale_size_manual(values = c(rep(4,10),2), guide = "none")+
  ggrepel::geom_text_repel(aes(label = anno3), size = 3.5,
                           max.overlaps = 20, point.padding = 3)+
  labs(y = "Weight", x = "Behavioural trait", color = "Behaviour type",
       title = "Non-exposed fish - Factor3")+
  theme_classic()+
  theme(axis.text.x = element_blank(), axis.ticks.x = element_blank(),
        axis.text = element_text(color = "black", size = 12), legend.position = c(0.2, 0.8),
        axis.title = element_text(size = 13), legend.text = element_text(size = 10), legend.title = element_text(size = 11),
        legend.box = "horizontal", plot.title = element_text(hjust = 0.5, face = "bold", size = 13),
        strip.text.x = element_text(size = 13), strip.background = element_blank())

plot.TypeC.F3.B <-
ggplot(nobatch.mofa.TypeC.Factor3$micro, aes(rank, value))+
  geom_hline(yintercept = 0, color = "grey50", linetype = 2)+
  geom_point(color = "grey60", size = 1, alpha = 0.7)+
  geom_point(aes(color = Putative.source, shape = Potential.fish.pathogen, size = anno))+
  scale_color_manual(values = micro.cats.cols)+
  scale_shape_manual(values = c(17,15,16))+
  scale_size_manual(values = c(rep(4,10),2), guide = "none")+
  ggrepel::geom_text_repel(aes(label = anno), size = 3.5,
                           max.overlaps = 20)+
  expand_limits(x = c(-2, 102))+
  labs(y = "Weight", x = "Genus", color = "Source", shape = "Pathogen")+
  theme_classic()+
  theme(axis.text.x = element_blank(), axis.ticks.x = element_blank(),
        axis.text = element_text(color = "black", size = 12), legend.position = c(0.3, 0.8),
        axis.title = element_text(size = 13), legend.text = element_text(size = 10), legend.title = element_text(size = 11),
        legend.box = "horizontal",
        strip.text.x = element_blank(), strip.background = element_blank())
```

```
cowplot::plot_grid(plot.TypeC.F3.A, plot.TypeC.F3.B, nrow = 2, labels = c("A","B"),
                   align = "hv", axis = "lr")
```

```
ggsave("figures_2024/Supp_figure_S6_MOFA_TypeC_Factor3.png",units = "in", dpi = 600, width = 9.5, height = 8)
```

### TypeC F4

```
nobatch.mofa.TypeC.Factor4 <- mofa_figure_ind_factor(
  "mofa_runs_20250211/mofa_results_250211_nobatch_TypeC_incl_lat_f7_v3_feature_weights.csv",
  "Factor4", feature.num.pos = 5, feature.num.neg = 5
)

nobatch.mofa.TypeC.Factor4$micro <- merge(
  nobatch.mofa.TypeC.Factor4$micro, micro.genera,
  by.x = "feature", by.y = "Genus", all.x = T, all.y = F
)

nobatch.mofa.TypeC.Factor4$micro$Putative.source[is.na(nobatch.mofa.TypeC.Factor4$micro$Putative.source)] <- "not_assessed"
nobatch.mofa.TypeC.Factor4$micro$Potential.fish.pathogen[is.na(nobatch.mofa.TypeC.Factor4$micro$Potential.fish.pathogen)] <- "not_assessed"

nobatch.mofa.TypeC.Factor4$micro$Putative.source <- factor(
  nobatch.mofa.TypeC.Factor4$micro$Putative.source,
  levels = names(micro.cats.cols)
)
nobatch.mofa.TypeC.Factor4$micro$Potential.fish.pathogen <- factor(
  nobatch.mofa.TypeC.Factor4$micro$Potential.fish.pathogen,
  levels = c("documented","not_documented","not_assessed")
)

nobatch.mofa.TypeC.Factor4$beh$anno2 <- factor(
  sapply(nobatch.mofa.TypeC.Factor4$beh$feature, function(x){
    i <- grep(x,beh.cats)
    if(length(i) == 0){
      "other"
    } else {
      names(beh.cats[i])[[1]]
    }
  }),
  levels = names(beh.cats.cols)
)

nobatch.mofa.TypeC.Factor4$beh$anno3 <- factor(
  sapply(as.character(nobatch.mofa.TypeC.Factor4$beh$anno), function(x){
    if(x == ""){
      ""
    } else {
      x <- gsub("duration", "dur", x, ignore.case = T)
      x <- gsub("freq", "freq", x, ignore.case = T)
      x <- gsub("\\.time","",x)
    }
  })
)
```

```
plot.TypeC.F4.A <-
ggplot(nobatch.mofa.TypeC.Factor4$beh, aes(rank, value))+
  geom_hline(yintercept = 0, color = "grey50", linetype = 2)+
  geom_point(color = "grey60", size = 1, alpha = 0.7)+
  geom_point(aes(color = anno2, size = anno))+
  scale_color_manual(values = beh.cats.cols)+
  scale_size_manual(values = c(rep(4,10),2), guide = "none")+
  ggrepel::geom_text_repel(aes(label = anno3), size = 3.5,
                           max.overlaps = 20, point.padding = 3)+
  labs(y = "Weight", x = "Behavioural trait", color = "Behaviour type",
       title = "Non-exposed fish - Factor4")+
  theme_classic()+
  theme(axis.text.x = element_blank(), axis.ticks.x = element_blank(),
        axis.text = element_text(color = "black", size = 12), legend.position = c(0.2, 0.8),
        axis.title = element_text(size = 13), legend.text = element_text(size = 10), legend.title = element_text(size = 11),
        legend.box = "horizontal", plot.title = element_text(hjust = 0.5, face = "bold", size = 13),
        strip.text.x = element_text(size = 13), strip.background = element_blank())

plot.TypeC.F4.B <-
ggplot(nobatch.mofa.TypeC.Factor4$micro, aes(rank, value))+
  geom_hline(yintercept = 0, color = "grey50", linetype = 2)+
  geom_point(color = "grey60", size = 1, alpha = 0.7)+
  geom_point(aes(color = Putative.source, shape = Potential.fish.pathogen, size = anno))+
  scale_color_manual(values = micro.cats.cols)+
  scale_shape_manual(values = c(17,15,16))+
  scale_size_manual(values = c(rep(4,10),2), guide = "none")+
  ggrepel::geom_text_repel(aes(label = anno), size = 3.5,
                           max.overlaps = 20)+
  expand_limits(x = c(-2, 102))+
  labs(y = "Weight", x = "Genus", color = "Source", shape = "Pathogen")+
  theme_classic()+
  theme(axis.text.x = element_blank(), axis.ticks.x = element_blank(),
        axis.text = element_text(color = "black", size = 12), legend.position = c(0.3, 0.8),
        axis.title = element_text(size = 13), legend.text = element_text(size = 10), legend.title = element_text(size = 11),
        legend.box = "horizontal",
        strip.text.x = element_blank(), strip.background = element_blank())
```

```
cowplot::plot_grid(plot.TypeC.F4.A, plot.TypeC.F4.B, nrow = 2, labels = c("A","B"),
                   align = "hv", axis = "lr")
```

```
ggsave("figures_2024/Supp_figure_S7_MOFA_TypeC_Factor4.png",units = "in", dpi = 600, width = 9.5, height = 8)
```

### TypeC F5

```
nobatch.mofa.TypeC.Factor5 <- mofa_figure_ind_factor(
  "mofa_runs_20250211/mofa_results_250211_nobatch_TypeC_incl_lat_f7_v3_feature_weights.csv",
  "Factor5", feature.num.pos = 5, feature.num.neg = 5
)

nobatch.mofa.TypeC.Factor5$micro <- merge(
  nobatch.mofa.TypeC.Factor5$micro, micro.genera,
  by.x = "feature", by.y = "Genus", all.x = T, all.y = F
)

nobatch.mofa.TypeC.Factor5$micro$Putative.source[is.na(nobatch.mofa.TypeC.Factor5$micro$Putative.source)] <- "not_assessed"
nobatch.mofa.TypeC.Factor5$micro$Potential.fish.pathogen[is.na(nobatch.mofa.TypeC.Factor5$micro$Potential.fish.pathogen)] <- "not_assessed"

nobatch.mofa.TypeC.Factor5$micro$Putative.source <- factor(
  nobatch.mofa.TypeC.Factor5$micro$Putative.source,
  levels = names(micro.cats.cols)
)
nobatch.mofa.TypeC.Factor5$micro$Potential.fish.pathogen <- factor(
  nobatch.mofa.TypeC.Factor5$micro$Potential.fish.pathogen,
  levels = c("documented","not_documented","not_assessed")
)

nobatch.mofa.TypeC.Factor5$beh$anno2 <- factor(
  sapply(nobatch.mofa.TypeC.Factor5$beh$feature, function(x){
    i <- grep(x,beh.cats)
    if(length(i) == 0){
      "other"
    } else {
      names(beh.cats[i])[[1]]
    }
  }),
  levels = names(beh.cats.cols)
)

nobatch.mofa.TypeC.Factor5$beh$anno3 <- factor(
  sapply(as.character(nobatch.mofa.TypeC.Factor5$beh$anno), function(x){
    if(x == ""){
      ""
    } else {
      x <- gsub("duration", "dur", x, ignore.case = T)
      x <- gsub("freq", "freq", x, ignore.case = T)
      x <- gsub("\\.time","",x)
    }
  })
)
```

```
plot.TypeC.F5.A <-
ggplot(nobatch.mofa.TypeC.Factor5$beh, aes(rank, value))+
  geom_hline(yintercept = 0, color = "grey50", linetype = 2)+
  geom_point(color = "grey60", size = 1, alpha = 0.7)+
  geom_point(aes(color = anno2, size = anno))+
  scale_color_manual(values = beh.cats.cols)+
  scale_size_manual(values = c(rep(4,10),2), guide = "none")+
  ggrepel::geom_text_repel(aes(label = anno3), size = 3.5,
                           max.overlaps = 20, point.padding = 3)+
  labs(y = "Weight", x = "Behavioural trait", color = "Behaviour type",
       title = "Non-exposed fish - Factor5")+
  theme_classic()+
  theme(axis.text.x = element_blank(), axis.ticks.x = element_blank(),
        axis.text = element_text(color = "black", size = 12), legend.position = c(0.2, 0.85),
        axis.title = element_text(size = 13), legend.text = element_text(size = 10), legend.title = element_text(size = 11),
        legend.box = "horizontal", plot.title = element_text(hjust = 0.5, face = "bold", size = 13),
        strip.text.x = element_text(size = 13), strip.background = element_blank())

plot.TypeC.F5.B <-
ggplot(nobatch.mofa.TypeC.Factor5$micro, aes(rank, value))+
  geom_hline(yintercept = 0, color = "grey50", linetype = 2)+
  geom_point(color = "grey60", size = 1, alpha = 0.7)+
  geom_point(aes(color = Putative.source, shape = Potential.fish.pathogen, size = anno))+
  scale_color_manual(values = micro.cats.cols)+
  scale_shape_manual(values = c(17,15,16))+
  scale_size_manual(values = c(rep(4,10),2), guide = "none")+
  ggrepel::geom_text_repel(aes(label = anno), size = 3.5,
                           max.overlaps = 20)+
  expand_limits(x = c(-2, 102))+
  labs(y = "Weight", x = "Genus", color = "Source", shape = "Pathogen")+
  theme_classic()+
  theme(axis.text.x = element_blank(), axis.ticks.x = element_blank(),
        axis.text = element_text(color = "black", size = 12), legend.position = c(0.3, 0.8),
        axis.title = element_text(size = 13), legend.text = element_text(size = 10), legend.title = element_text(size = 11),
        legend.box = "horizontal",
        strip.text.x = element_blank(), strip.background = element_blank())
```

```
cowplot::plot_grid(plot.TypeC.F5.A, plot.TypeC.F5.B, nrow = 2, labels = c("A","B"),
                   align = "hv", axis = "lr")
```

```
ggsave("figures_2024/Supp_figure_S8_MOFA_TypeC_Factor5.png",units = "in", dpi = 600, width = 9.5, height = 8)
```

### TypeC F6

```
nobatch.mofa.TypeC.Factor6 <- mofa_figure_ind_factor(
  "mofa_runs_20250211/mofa_results_250211_nobatch_TypeC_incl_lat_f7_v3_feature_weights.csv",
  "Factor6", feature.num.pos = 5, feature.num.neg = 5
)

nobatch.mofa.TypeC.Factor6$micro <- merge(
  nobatch.mofa.TypeC.Factor6$micro, micro.genera,
  by.x = "feature", by.y = "Genus", all.x = T, all.y = F
)

nobatch.mofa.TypeC.Factor6$micro$Putative.source[is.na(nobatch.mofa.TypeC.Factor6$micro$Putative.source)] <- "not_assessed"
nobatch.mofa.TypeC.Factor6$micro$Potential.fish.pathogen[is.na(nobatch.mofa.TypeC.Factor6$micro$Potential.fish.pathogen)] <- "not_assessed"

nobatch.mofa.TypeC.Factor6$micro$Putative.source <- factor(
  nobatch.mofa.TypeC.Factor6$micro$Putative.source,
  levels = names(micro.cats.cols)
)
nobatch.mofa.TypeC.Factor6$micro$Potential.fish.pathogen <- factor(
  nobatch.mofa.TypeC.Factor6$micro$Potential.fish.pathogen,
  levels = c("documented","not_documented","not_assessed")
)

nobatch.mofa.TypeC.Factor6$beh$anno2 <- factor(
  sapply(nobatch.mofa.TypeC.Factor6$beh$feature, function(x){
    i <- grep(x,beh.cats)
    if(length(i) == 0){
      "other"
    } else {
      names(beh.cats[i])[[1]]
    }
  }),
  levels = names(beh.cats.cols)
)

nobatch.mofa.TypeC.Factor6$beh$anno3 <- factor(
  sapply(as.character(nobatch.mofa.TypeC.Factor6$beh$anno), function(x){
    if(x == ""){
      ""
    } else {
      x <- gsub("duration", "dur", x, ignore.case = T)
      x <- gsub("freq", "freq", x, ignore.case = T)
      x <- gsub("\\.time","",x)
    }
  })
)
```

```
plot.TypeC.F6.A <-
ggplot(nobatch.mofa.TypeC.Factor6$beh, aes(rank, value))+
  geom_hline(yintercept = 0, color = "grey50", linetype = 2)+
  geom_point(color = "grey60", size = 1, alpha = 0.7)+
  geom_point(aes(color = anno2, size = anno))+
  scale_color_manual(values = beh.cats.cols)+
  scale_size_manual(values = c(rep(4,10),2), guide = "none")+
  ggrepel::geom_text_repel(aes(label = anno3), size = 3.5,
                           max.overlaps = 20, point.padding = 3)+
  labs(y = "Weight", x = "Behavioural trait", color = "Behaviour type",
       title = "Non-exposed fish - Factor6")+
  theme_classic()+
  theme(axis.text.x = element_blank(), axis.ticks.x = element_blank(),
        axis.text = element_text(color = "black", size = 12), legend.position = c(0.2, 0.8),
        axis.title = element_text(size = 13), legend.text = element_text(size = 10), legend.title = element_text(size = 11),
        legend.box = "horizontal", plot.title = element_text(hjust = 0.5, face = "bold", size = 13),
        strip.text.x = element_text(size = 13), strip.background = element_blank())

plot.TypeC.F6.B <-
ggplot(nobatch.mofa.TypeC.Factor6$micro, aes(rank, value))+
  geom_hline(yintercept = 0, color = "grey50", linetype = 2)+
  geom_point(color = "grey60", size = 1, alpha = 0.7)+
  geom_point(aes(color = Putative.source, shape = Potential.fish.pathogen, size = anno))+
  scale_color_manual(values = micro.cats.cols)+
  scale_shape_manual(values = c(17,15,16))+
  scale_size_manual(values = c(rep(4,10),2), guide = "none")+
  ggrepel::geom_text_repel(aes(label = anno), size = 3.5,
                           max.overlaps = 20)+
  expand_limits(x = c(-2, 102))+
  labs(y = "Weight", x = "Genus", color = "Source", shape = "Pathogen")+
  theme_classic()+
  theme(axis.text.x = element_blank(), axis.ticks.x = element_blank(),
        axis.text = element_text(color = "black", size = 12), legend.position = c(0.3, 0.8),
        axis.title = element_text(size = 13), legend.text = element_text(size = 10), legend.title = element_text(size = 11),
        legend.box = "horizontal",
        strip.text.x = element_blank(), strip.background = element_blank())
```

```
cowplot::plot_grid(plot.TypeC.F6.A, plot.TypeC.F6.B, nrow = 2, labels = c("A","B"),
                   align = "hv", axis = "lr")
```

```
ggsave("figures_2024/Supp_figure_S9_MOFA_TypeC_Factor6.png",units = "in", dpi = 600, width = 9.5, height = 8)
```

### TypeC F7

```
nobatch.mofa.TypeC.Factor7 <- mofa_figure_ind_factor(
  "mofa_runs_20250211/mofa_results_250211_nobatch_TypeC_incl_lat_f7_v3_feature_weights.csv",
  "Factor7", feature.num.pos = 5, feature.num.neg = 5
)

nobatch.mofa.TypeC.Factor7$micro <- merge(
  nobatch.mofa.TypeC.Factor7$micro, micro.genera,
  by.x = "feature", by.y = "Genus", all.x = T, all.y = F
)

nobatch.mofa.TypeC.Factor7$micro$Putative.source[is.na(nobatch.mofa.TypeC.Factor7$micro$Putative.source)] <- "not_assessed"
nobatch.mofa.TypeC.Factor7$micro$Potential.fish.pathogen[is.na(nobatch.mofa.TypeC.Factor7$micro$Potential.fish.pathogen)] <- "not_assessed"

nobatch.mofa.TypeC.Factor7$micro$Putative.source <- factor(
  nobatch.mofa.TypeC.Factor7$micro$Putative.source,
  levels = names(micro.cats.cols)
)
nobatch.mofa.TypeC.Factor7$micro$Potential.fish.pathogen <- factor(
  nobatch.mofa.TypeC.Factor7$micro$Potential.fish.pathogen,
  levels = c("documented","not_documented","not_assessed")
)

nobatch.mofa.TypeC.Factor7$beh$anno2 <- factor(
  sapply(nobatch.mofa.TypeC.Factor7$beh$feature, function(x){
    i <- grep(x,beh.cats)
    if(length(i) == 0){
      "other"
    } else {
      names(beh.cats[i])[[1]]
    }
  }),
  levels = names(beh.cats.cols)
)

nobatch.mofa.TypeC.Factor7$beh$anno3 <- factor(
  sapply(as.character(nobatch.mofa.TypeC.Factor7$beh$anno), function(x){
    if(x == ""){
      ""
    } else {
      x <- gsub("duration", "dur", x, ignore.case = T)
      x <- gsub("freq", "freq", x, ignore.case = T)
      x <- gsub("\\.time","",x)
    }
  })
)
```

```
plot.TypeC.F7.A <-
ggplot(nobatch.mofa.TypeC.Factor7$beh, aes(rank, value))+
  geom_hline(yintercept = 0, color = "grey50", linetype = 2)+
  geom_point(color = "grey60", size = 1, alpha = 0.7)+
  geom_point(aes(color = anno2, size = anno))+
  scale_color_manual(values = beh.cats.cols)+
  scale_size_manual(values = c(rep(4,10),2), guide = "none")+
  ggrepel::geom_text_repel(aes(label = anno3), size = 3.5,
                           max.overlaps = 20, point.padding = 3)+
  labs(y = "Weight", x = "Behavioural trait", color = "Behaviour type",
       title = "Non-exposed fish - Factor7")+
  theme_classic()+
  theme(axis.text.x = element_blank(), axis.ticks.x = element_blank(),
        axis.text = element_text(color = "black", size = 12), legend.position = c(0.2, 0.8),
        axis.title = element_text(size = 13), legend.text = element_text(size = 10), legend.title = element_text(size = 11),
        legend.box = "horizontal", plot.title = element_text(hjust = 0.5, face = "bold", size = 13),
        strip.text.x = element_text(size = 13), strip.background = element_blank())

plot.TypeC.F7.B <-
ggplot(nobatch.mofa.TypeC.Factor7$micro, aes(rank, value))+
  geom_hline(yintercept = 0, color = "grey50", linetype = 2)+
  geom_point(color = "grey60", size = 1, alpha = 0.7)+
  geom_point(aes(color = Putative.source, shape = Potential.fish.pathogen, size = anno))+
  scale_color_manual(values = micro.cats.cols)+
  scale_shape_manual(values = c(17,15,16))+
  scale_size_manual(values = c(rep(4,10),2), guide = "none")+
  ggrepel::geom_text_repel(aes(label = anno), size = 3.5,
                           max.overlaps = 20)+
  expand_limits(x = c(-2, 102))+
  labs(y = "Weight", x = "Genus", color = "Source", shape = "Pathogen")+
  theme_classic()+
  theme(axis.text.x = element_blank(), axis.ticks.x = element_blank(),
        axis.text = element_text(color = "black", size = 12), legend.position = c(0.5, 0.2),
        axis.title = element_text(size = 13), legend.text = element_text(size = 10), legend.title = element_text(size = 11),
        legend.box = "horizontal",
        strip.text.x = element_blank(), strip.background = element_blank())
```

```
cowplot::plot_grid(plot.TypeC.F7.A, plot.TypeC.F7.B, nrow = 2, labels = c("A","B"),
                   align = "hv", axis = "lr")
```

```
ggsave("figures_2024/Supp_figure_S10_MOFA_TypeC_Factor7.png",units = "in", dpi = 600, width = 9.5, height = 8)
```

### TypeP F2

```
nobatch.mofa.TypeP.Factor2 <- mofa_figure_ind_factor(
  "mofa_runs_20250211/mofa_results_250211_nobatch_TypeP_incl_lat_f7_v3_feature_weights.csv",
  "Factor2", feature.num.pos = 5, feature.num.neg = 5
)

nobatch.mofa.TypeP.Factor2$micro <- merge(
  nobatch.mofa.TypeP.Factor2$micro, micro.genera,
  by.x = "feature", by.y = "Genus", all.x = T, all.y = F
)

nobatch.mofa.TypeP.Factor2$micro$Putative.source[is.na(nobatch.mofa.TypeP.Factor2$micro$Putative.source)] <- "not_assessed"
nobatch.mofa.TypeP.Factor2$micro$Potential.fish.pathogen[is.na(nobatch.mofa.TypeP.Factor2$micro$Potential.fish.pathogen)] <- "not_assessed"

nobatch.mofa.TypeP.Factor2$micro$Putative.source <- factor(
  nobatch.mofa.TypeP.Factor2$micro$Putative.source,
  levels = names(micro.cats.cols)
)
nobatch.mofa.TypeP.Factor2$micro$Potential.fish.pathogen <- factor(
  nobatch.mofa.TypeP.Factor2$micro$Potential.fish.pathogen,
  levels = c("documented","not_documented","not_assessed")
)

nobatch.mofa.TypeP.Factor2$beh$anno2 <- factor(
  sapply(nobatch.mofa.TypeP.Factor2$beh$feature, function(x){
    i <- grep(x,beh.cats)
    if(length(i) == 0){
      "other"
    } else {
      names(beh.cats[i])[[1]]
    }
  }),
  levels = names(beh.cats.cols)
)

nobatch.mofa.TypeP.Factor2$beh$anno3 <- factor(
  sapply(as.character(nobatch.mofa.TypeP.Factor2$beh$anno), function(x){
    if(x == ""){
      ""
    } else {
      x <- gsub("duration", "dur", x, ignore.case = T)
      x <- gsub("freq", "freq", x, ignore.case = T)
      x <- gsub("\\.time","",x)
    }
  })
)
```

```
plot.TypeP.F2.A <-
ggplot(nobatch.mofa.TypeP.Factor2$beh, aes(rank, value))+
  geom_hline(yintercept = 0, color = "grey50", linetype = 2)+
  geom_point(color = "grey60", size = 1, alpha = 0.7)+
  geom_point(aes(color = anno2, size = anno))+
  scale_color_manual(values = beh.cats.cols)+
  scale_size_manual(values = c(rep(4,10),2), guide = "none")+
  ggrepel::geom_text_repel(aes(label = anno3), size = 3.5,
                           max.overlaps = 20, point.padding = 3)+
  labs(y = "Weight", x = "Behavioural trait", color = "Behaviour type",
       title = "Predator-exposed fish - Factor2")+
  theme_classic()+
  theme(axis.text.x = element_blank(), axis.ticks.x = element_blank(),
        axis.text = element_text(color = "black", size = 12), legend.position = c(0.8, 0.3),
        axis.title = element_text(size = 13), legend.text = element_text(size = 10), legend.title = element_text(size = 11),
        legend.box = "horizontal", plot.title = element_text(hjust = 0.5, face = "bold", size = 13),
        strip.text.x = element_text(size = 13), strip.background = element_blank())

plot.TypeP.F2.B <-
ggplot(nobatch.mofa.TypeP.Factor2$micro, aes(rank, value))+
  geom_hline(yintercept = 0, color = "grey50", linetype = 2)+
  geom_point(color = "grey60", size = 1, alpha = 0.7)+
  geom_point(aes(color = Putative.source, shape = Potential.fish.pathogen, size = anno))+
  scale_color_manual(values = micro.cats.cols)+
  scale_shape_manual(values = c(17,15,16))+
  scale_size_manual(values = c(rep(4,10),2), guide = "none")+
  ggrepel::geom_text_repel(aes(label = anno), size = 3.5,
                           max.overlaps = 20)+
  expand_limits(x = c(-2, 102))+
  labs(y = "Weight", x = "Genus", color = "Source", shape = "Pathogen")+
  theme_classic()+
  theme(axis.text.x = element_blank(), axis.ticks.x = element_blank(),
        axis.text = element_text(color = "black", size = 12), legend.position = c(0.3, 0.8),
        axis.title = element_text(size = 13), legend.text = element_text(size = 10), legend.title = element_text(size = 11),
        legend.box = "horizontal",
        strip.text.x = element_blank(), strip.background = element_blank())
```

```
cowplot::plot_grid(plot.TypeP.F2.A, plot.TypeP.F2.B, nrow = 2, labels = c("A","B"),
                   align = "hv", axis = "lr")
```

```
ggsave("figures_2024/Supp_figure_S11_MOFA_TypeP_Factor2.png",units = "in", dpi = 600, width = 9.5, height = 8)
```

### TypeP F5

```
nobatch.mofa.TypeP.Factor5 <- mofa_figure_ind_factor(
  "mofa_runs_20250211/mofa_results_250211_nobatch_TypeP_incl_lat_f7_v3_feature_weights.csv",
  "Factor5", feature.num.pos = 5, feature.num.neg = 5
)

nobatch.mofa.TypeP.Factor5$micro <- merge(
  nobatch.mofa.TypeP.Factor5$micro, micro.genera,
  by.x = "feature", by.y = "Genus", all.x = T, all.y = F
)

nobatch.mofa.TypeP.Factor5$micro$Putative.source[is.na(nobatch.mofa.TypeP.Factor5$micro$Putative.source)] <- "not_assessed"
nobatch.mofa.TypeP.Factor5$micro$Potential.fish.pathogen[is.na(nobatch.mofa.TypeP.Factor5$micro$Potential.fish.pathogen)] <- "not_assessed"

nobatch.mofa.TypeP.Factor5$micro$Putative.source <- factor(
  nobatch.mofa.TypeP.Factor5$micro$Putative.source,
  levels = names(micro.cats.cols)
)
nobatch.mofa.TypeP.Factor5$micro$Potential.fish.pathogen <- factor(
  nobatch.mofa.TypeP.Factor5$micro$Potential.fish.pathogen,
  levels = c("documented","not_documented","not_assessed")
)

nobatch.mofa.TypeP.Factor5$beh$anno2 <- factor(
  sapply(nobatch.mofa.TypeP.Factor5$beh$feature, function(x){
    i <- grep(x,beh.cats)
    if(length(i) == 0){
      "other"
    } else {
      names(beh.cats[i])[[1]]
    }
  }),
  levels = names(beh.cats.cols)
)

nobatch.mofa.TypeP.Factor5$beh$anno3 <- factor(
  sapply(as.character(nobatch.mofa.TypeP.Factor5$beh$anno), function(x){
    if(x == ""){
      ""
    } else {
      x <- gsub("duration", "dur", x, ignore.case = T)
      x <- gsub("freq", "freq", x, ignore.case = T)
      x <- gsub("\\.time","",x)
    }
  })
)
```

```
plot.TypeP.F5.A <-
ggplot(nobatch.mofa.TypeP.Factor5$beh, aes(rank, value))+
  geom_hline(yintercept = 0, color = "grey50", linetype = 2)+
  geom_point(color = "grey60", size = 1, alpha = 0.7)+
  geom_point(aes(color = anno2, size = anno))+
  scale_color_manual(values = beh.cats.cols)+
  scale_size_manual(values = c(rep(4,10),2), guide = "none")+
  ggrepel::geom_text_repel(aes(label = anno3), size = 3.5,
                           max.overlaps = 20, point.padding = 3)+
  labs(y = "Weight", x = "Behavioural trait", color = "Behaviour type",
       title = "Predator-exposed fish - Factor5")+
  theme_classic()+
  theme(axis.text.x = element_blank(), axis.ticks.x = element_blank(),
        axis.text = element_text(color = "black", size = 12), legend.position = c(0.2, 0.8),
        axis.title = element_text(size = 13), legend.text = element_text(size = 10), legend.title = element_text(size = 11),
        legend.box = "horizontal", plot.title = element_text(hjust = 0.5, face = "bold", size = 13),
        strip.text.x = element_text(size = 13), strip.background = element_blank())

plot.TypeP.F5.B <-
ggplot(nobatch.mofa.TypeP.Factor5$micro, aes(rank, value))+
  geom_hline(yintercept = 0, color = "grey50", linetype = 2)+
  geom_point(color = "grey60", size = 1, alpha = 0.7)+
  geom_point(aes(color = Putative.source, shape = Potential.fish.pathogen, size = anno))+
  scale_color_manual(values = micro.cats.cols)+
  scale_shape_manual(values = c(17,15,16))+
  scale_size_manual(values = c(rep(4,10),2), guide = "none")+
  ggrepel::geom_text_repel(aes(label = anno), size = 3.5,
                           max.overlaps = 20)+
  expand_limits(x = c(-2, 102))+
  labs(y = "Weight", x = "Genus", color = "Source", shape = "Pathogen")+
  theme_classic()+
  theme(axis.text.x = element_blank(), axis.ticks.x = element_blank(),
        axis.text = element_text(color = "black", size = 12), legend.position = c(0.3, 0.8),
        axis.title = element_text(size = 13), legend.text = element_text(size = 10), legend.title = element_text(size = 11),
        legend.box = "horizontal",
        strip.text.x = element_blank(), strip.background = element_blank())
```

```
cowplot::plot_grid(plot.TypeP.F5.A, plot.TypeP.F5.B, nrow = 2, labels = c("A","B"),
                   align = "hv", axis = "lr")
```

```
ggsave("figures_2024/Supp_figure_S12_MOFA_TypeP_Factor5.png",units = "in", dpi = 600, width = 9.5, height = 8)
```

### TypeP F6

```
nobatch.mofa.TypeP.Factor6 <- mofa_figure_ind_factor(
  "mofa_runs_20250211/mofa_results_250211_nobatch_TypeP_incl_lat_f7_v3_feature_weights.csv",
  "Factor6", feature.num.pos = 5, feature.num.neg = 5
)

nobatch.mofa.TypeP.Factor6$micro <- merge(
  nobatch.mofa.TypeP.Factor6$micro, micro.genera,
  by.x = "feature", by.y = "Genus", all.x = T, all.y = F
)

nobatch.mofa.TypeP.Factor6$micro$Putative.source[is.na(nobatch.mofa.TypeP.Factor6$micro$Putative.source)] <- "not_assessed"
nobatch.mofa.TypeP.Factor6$micro$Potential.fish.pathogen[is.na(nobatch.mofa.TypeP.Factor6$micro$Potential.fish.pathogen)] <- "not_assessed"

nobatch.mofa.TypeP.Factor6$micro$Putative.source <- factor(
  nobatch.mofa.TypeP.Factor6$micro$Putative.source,
  levels = names(micro.cats.cols)
)
nobatch.mofa.TypeP.Factor6$micro$Potential.fish.pathogen <- factor(
  nobatch.mofa.TypeP.Factor6$micro$Potential.fish.pathogen,
  levels = c("documented","not_documented","not_assessed")
)

nobatch.mofa.TypeP.Factor6$beh$anno2 <- factor(
  sapply(nobatch.mofa.TypeP.Factor6$beh$feature, function(x){
    i <- grep(x,beh.cats)
    if(length(i) == 0){
      "other"
    } else {
      names(beh.cats[i])[[1]]
    }
  }),
  levels = names(beh.cats.cols)
)

nobatch.mofa.TypeP.Factor6$beh$anno3 <- factor(
  sapply(as.character(nobatch.mofa.TypeP.Factor6$beh$anno), function(x){
    if(x == ""){
      ""
    } else {
      x <- gsub("duration", "dur", x, ignore.case = T)
      x <- gsub("freq", "freq", x, ignore.case = T)
      x <- gsub("\\.time","",x)
    }
  })
)
```

```
plot.TypeP.F6.A <-
ggplot(nobatch.mofa.TypeP.Factor6$beh, aes(rank, value))+
  geom_hline(yintercept = 0, color = "grey50", linetype = 2)+
  geom_point(color = "grey60", size = 1, alpha = 0.7)+
  geom_point(aes(color = anno2, size = anno))+
  scale_color_manual(values = beh.cats.cols)+
  scale_size_manual(values = c(rep(4,10),2), guide = "none")+
  ggrepel::geom_text_repel(aes(label = anno3), size = 3.5,
                           max.overlaps = 20, point.padding = 3)+
  expand_limits(y = c(0, 0.15))+
  labs(y = "Weight", x = "Behavioural trait", color = "Behaviour type",
       title = "Predator-exposed fish - Factor6")+
  theme_classic()+
  theme(axis.text.x = element_blank(), axis.ticks.x = element_blank(),
        axis.text = element_text(color = "black", size = 12), legend.position = c(0.2, 0.8),
        axis.title = element_text(size = 13), legend.text = element_text(size = 10), legend.title = element_text(size = 11),
        legend.box = "horizontal", plot.title = element_text(hjust = 0.5, face = "bold", size = 13),
        strip.text.x = element_text(size = 13), strip.background = element_blank())

plot.TypeP.F6.B <-
ggplot(nobatch.mofa.TypeP.Factor6$micro, aes(rank, value))+
  geom_hline(yintercept = 0, color = "grey50", linetype = 2)+
  geom_point(color = "grey60", size = 1, alpha = 0.7)+
  geom_point(aes(color = Putative.source, shape = Potential.fish.pathogen, size = anno))+
  scale_color_manual(values = micro.cats.cols)+
  scale_shape_manual(values = c(17,15,16))+
  scale_size_manual(values = c(rep(4,10),2), guide = "none")+
  ggrepel::geom_text_repel(aes(label = anno), size = 3.5,
                           max.overlaps = 20)+
  expand_limits(x = c(-2, 102))+
  labs(y = "Weight", x = "Genus", color = "Source", shape = "Pathogen")+
  theme_classic()+
  theme(axis.text.x = element_blank(), axis.ticks.x = element_blank(),
        axis.text = element_text(color = "black", size = 12), legend.position = c(0.3, 0.8),
        axis.title = element_text(size = 13), legend.text = element_text(size = 10), legend.title = element_text(size = 11),
        legend.box = "horizontal",
        strip.text.x = element_blank(), strip.background = element_blank())
```

```
cowplot::plot_grid(plot.TypeP.F6.A, plot.TypeP.F6.B, nrow = 2, labels = c("A","B"),
                   align = "hv", axis = "lr")
```

```
ggsave("figures_2024/Supp_figure_S13_MOFA_TypeP_Factor6.png",units = "in", dpi = 600, width = 9.5, height = 8)
```

### TypeP F7

```
nobatch.mofa.TypeP.Factor7 <- mofa_figure_ind_factor(
  "mofa_runs_20250211/mofa_results_250211_nobatch_TypeP_incl_lat_f7_v3_feature_weights.csv",
  "Factor7", feature.num.pos = 5, feature.num.neg = 5
)

nobatch.mofa.TypeP.Factor7$micro <- merge(
  nobatch.mofa.TypeP.Factor7$micro, micro.genera,
  by.x = "feature", by.y = "Genus", all.x = T, all.y = F
)

nobatch.mofa.TypeP.Factor7$micro$Putative.source[is.na(nobatch.mofa.TypeP.Factor7$micro$Putative.source)] <- "not_assessed"
nobatch.mofa.TypeP.Factor7$micro$Potential.fish.pathogen[is.na(nobatch.mofa.TypeP.Factor7$micro$Potential.fish.pathogen)] <- "not_assessed"

nobatch.mofa.TypeP.Factor7$micro$Putative.source <- factor(
  nobatch.mofa.TypeP.Factor7$micro$Putative.source,
  levels = names(micro.cats.cols)
)
nobatch.mofa.TypeP.Factor7$micro$Potential.fish.pathogen <- factor(
  nobatch.mofa.TypeP.Factor7$micro$Potential.fish.pathogen,
  levels = c("documented","not_documented","not_assessed")
)

nobatch.mofa.TypeP.Factor7$beh$anno2 <- factor(
  sapply(nobatch.mofa.TypeP.Factor7$beh$feature, function(x){
    i <- grep(x,beh.cats)
    if(length(i) == 0){
      "other"
    } else {
      names(beh.cats[i])[[1]]
    }
  }),
  levels = names(beh.cats.cols)
)

nobatch.mofa.TypeP.Factor7$beh$anno3 <- factor(
  sapply(as.character(nobatch.mofa.TypeP.Factor7$beh$anno), function(x){
    if(x == ""){
      ""
    } else {
      x <- gsub("duration", "dur", x, ignore.case = T)
      x <- gsub("freq", "freq", x, ignore.case = T)
      x <- gsub("\\.time","",x)
    }
  })
)
```

```
plot.TypeP.F7.A <-
ggplot(nobatch.mofa.TypeP.Factor7$beh, aes(rank, value))+
  geom_hline(yintercept = 0, color = "grey50", linetype = 2)+
  geom_point(color = "grey60", size = 1, alpha = 0.7)+
  geom_point(aes(color = anno2, size = anno))+
  scale_color_manual(values = beh.cats.cols)+
  scale_size_manual(values = c(rep(4,10),2), guide = "none")+
  ggrepel::geom_text_repel(aes(label = anno3), size = 3.5,
                           max.overlaps = 20, point.padding = 3)+
  expand_limits(y = c(0, 0.01))+
  labs(y = "Weight", x = "Behavioural trait", color = "Behaviour type",
       title = "Predator-exposed fish - Factor7")+
  theme_classic()+
  theme(axis.text.x = element_blank(), axis.ticks.x = element_blank(),
        axis.text = element_text(color = "black", size = 12), legend.position = c(0.2, 0.8),
        axis.title = element_text(size = 13), legend.text = element_text(size = 10), legend.title = element_text(size = 11),
        legend.box = "horizontal", plot.title = element_text(hjust = 0.5, face = "bold", size = 13),
        strip.text.x = element_text(size = 13), strip.background = element_blank())

plot.TypeP.F7.B <-
ggplot(nobatch.mofa.TypeP.Factor7$micro, aes(rank, value))+
  geom_hline(yintercept = 0, color = "grey50", linetype = 2)+
  geom_point(color = "grey60", size = 1, alpha = 0.7)+
  geom_point(aes(color = Putative.source, shape = Potential.fish.pathogen, size = anno))+
  scale_color_manual(values = micro.cats.cols)+
  scale_shape_manual(values = c(17,15,16))+
  scale_size_manual(values = c(rep(4,10),2), guide = "none")+
  ggrepel::geom_text_repel(aes(label = anno), size = 3.5,
                           max.overlaps = 20)+
  expand_limits(x = c(-2, 102))+
  labs(y = "Weight", x = "Genus", color = "Source", shape = "Pathogen")+
  theme_classic()+
  theme(axis.text.x = element_blank(), axis.ticks.x = element_blank(),
        axis.text = element_text(color = "black", size = 12), legend.position = c(0.3, 0.8),
        axis.title = element_text(size = 13), legend.text = element_text(size = 10), legend.title = element_text(size = 11),
        legend.box = "horizontal",
        strip.text.x = element_blank(), strip.background = element_blank())
```

```
cowplot::plot_grid(plot.TypeP.F7.A, plot.TypeP.F7.B, nrow = 2, labels = c("A","B"),
                   align = "hv", axis = "lr")
```

```
ggsave("figures_2024/Supp_figure_S14_MOFA_TypeP_Factor7.png",units = "in", dpi = 600, width = 9.5, height = 8)
```

# Taxa investigations

To look up species of the genera identified as interesting by the
above analyses.

```
taxa.tab <- as.data.frame(tax_table(clean.ps))

taxa.tab <- cbind.data.frame(
  taxa.tab,
  data.frame(
    Galta_NP=taxa_sums(subset_samples(clean.ps, Population2 == "Galta")),
    Galta_P=taxa_sums(subset_samples(clean.ps, Population2 == "Galta_P")),
    Pristi_NP=taxa_sums(subset_samples(clean.ps, Population2 == "Pristi")),
    Total=taxa_sums(subset_samples(clean.ps, !Blank))
))
taxa.tab$Galta_NP.prop <- ifelse(taxa.tab$Total == 0,0,taxa.tab$Galta_NP / taxa.tab$Total)
taxa.tab$Galta_P.prop <- ifelse(taxa.tab$Total == 0,0,taxa.tab$Galta_P / taxa.tab$Total)
taxa.tab$Pristi_NP.prop <- ifelse(taxa.tab$Total == 0,0,taxa.tab$Pristi_NP / taxa.tab$Total)

taxa.tab <- taxa.tab[which(taxa.tab$Total > 0),]
```

# Supp File S1

```
# write out metadata for microbiome-behavior analysis for supplementary
supp.file.s1 <- data.frame(clean.ps.genus.clr@sam_data)[,c(
  # sample IDs
  "Video_nb", "SampleID", "Seq_ID",
  # fish & experiment variables
  "Type", "Population", "Parasite", "Sex", "length_cm", "weight_g",
  # lab variables
  "Ext.date", "DNA.ng.ul", "Blank",
  # DNA sequencing variables
  "Reads.sequenced", "Reads.P5.unmapped", "Reads.P5.host", "Bracken.readct.total",
  # Behavior data BEFORE predator trigger
  "Vang_BT", "Middle_Duration_BT", "Start_Duration_BT", "Border_duration_BT", "Dtot_BT", 
  "Dist_to_Pred_BT", "Pred_duration_BT", "Vel_BT", "VelBL_BT", "Center_Duration_BT", 
  "OpStart_Duration_BT", "Middle_Freq_BT", "Start_Freq_BT", "Center_Freq_BT", 
  "Border_Freq_BT", "OpStart_freq_BT", 
  # Behavior data AFTER predator trigger
  "Vang_AT", "Middle_Duration_AT", "Start_Duration_AT", "Border_duration_AT", "Dtot_AT", 
  "Dist_to_Pred_AT", "Pred_duration_AT", "Vel_AT", "VelBL_AT", "Center_Duration_AT", 
  "OpStart_Duration_AT", "Middle_Freq_AT", "Start_Freq_AT", "Center_Freq_AT", 
  "Border_Freq_AT", "OpStart_freq_AT", "Latency.time_AT"
)]

# update names of type/treatment to match manuscript
names(supp.file.s1)[which(names(supp.file.s1) == "Type")] <- "Treatment"
supp.file.s1[which(supp.file.s1$Treatment == "Control"),"Treatment"] <- "Non-exposed"
supp.file.s1[which(supp.file.s1$Treatment == "Predator"),"Treatment"] <- "Predator-exposed"

# add ENA sample and run accessions of fastq files
ena.runs <- read.csv("../Sticklebacks_ENA/sticklebacks_ena_submission_receipt.csv", header = F)
names(ena.runs) <- c("sample.lane","Exp.accession","Run.accession")
ena.runs$sample.alias <- gsub("_L00[12]","",ena.runs$sample.lane)
ena.samples <- read.delim("../Sticklebacks_ENA/sticklebacks_ENA_sample_accessions.txt", sep = "\t")

supp.file.s1$ENA_sample_accession <- sapply(supp.file.s1$Seq_ID, function(x){
  if(x %in% ena.samples$ALIAS){
    ena.samples[which(ena.samples$ALIAS == x),"ACCESSION"]
  } else {
    NA
  }
})

supp.file.s1$ENA_run_accessions <- sapply(supp.file.s1$Seq_ID, function(x){
  if(x %in% ena.runs$sample.alias){
    # two lanes per sample
    paste0(ena.runs[which(ena.runs$sample.alias == x),"Run.accession"], collapse = "|")
  } else {
    NA
  }
})

write.table(supp.file.s1,
            "figures_2024/Supp_file_S1_metadata_for_microbiome_behavior_analysis.txt",
            sep = "\t", row.names = F, quote = F)
```

# Versions

List R version.

```
version
```

```
##                _                                               
## platform       x86_64-w64-mingw32                              
## arch           x86_64                                          
## os             mingw32                                         
## crt            ucrt                                            
## system         x86_64, mingw32                                 
## status         Patched                                         
## major          4                                               
## minor          4.1                                             
## year           2024                                            
## month          06                                              
## day            25                                              
## svn rev        86831                                           
## language       R                                               
## version.string R version 4.4.1 Patched (2024-06-25 r86831 ucrt)
## nickname       Race for Your Life
```

List packages with versions used for analysis.

```
subset(data.frame(sessioninfo::package_info()), attached==TRUE, c(package, loadedversion))
```

```
##                   package loadedversion
## ade4                 ade4        1.7-22
## decontam         decontam        1.24.0
## factoextra     factoextra         1.0.7
## FactoMineR     FactoMineR          2.11
## ggplot2           ggplot2         3.5.1
## ggsignif         ggsignif         0.6.4
## kableExtra     kableExtra         1.4.0
## lattice           lattice        0.22-6
## Maaslin2         Maaslin2        1.18.0
## MOFA2               MOFA2        1.14.0
## permute           permute         0.9-7
## pheatmap         pheatmap        1.0.12
## phyloseq         phyloseq        1.48.0
## RColorBrewer RColorBrewer         1.1-3
## reshape2         reshape2         1.4.4
## vegan               vegan       2.6-6.1
```
